# The draft genome of the pest tephritid fruit fly *Bactrocera tryoni*: resources for the genomic analysis of hybridising species

**DOI:** 10.1186/1471-2164-15-1153

**Published:** 2014-12-20

**Authors:** Anthony Stuart Gilchrist, Deborah CA Shearman, Marianne Frommer, Kathryn A Raphael, Nandan P Deshpande, Marc R Wilkins, William B Sherwin, John A Sved

**Affiliations:** Evolution and Ecology Research Centre, School of Biological, Earth and Environmental Sciences, The University of New South Wales, Sydney, NSW 2052 Australia; Sydney Grammar School, College Street, Darlinghurst, NSW 2010 Australia; Systems Biology Initiative, School of Biotechnology and Biomolecular Sciences, The University of New South Wales, Sydney, 2052 NSW Australia; School of Biotechnology and Biomolecular Sciences, The University of New South Wales, Sydney, 2052 NSW Australia; Ramaciotti Centre for Gene Function Analysis, The University of New South Wales, Sydney, 2052 NSW Australia

## Abstract

**Background:**

The tephritid fruit flies include a number of economically important pests of horticulture, with a large accumulated body of research on their biology and control. Amongst the Tephritidae, the genus *Bactrocera,* containing over 400 species, presents various species groups of potential utility for genetic studies of speciation, behaviour or pest control. In Australia, there exists a triad of closely-related, sympatric *Bactrocera* species which do not mate in the wild but which, despite distinct morphologies and behaviours, can be force-mated in the laboratory to produce fertile hybrid offspring. To exploit the opportunities offered by genomics, such as the efficient identification of genetic loci central to pest behaviour and to the earliest stages of speciation, investigators require genomic resources for future investigations.

**Results:**

We produced a draft *de novo* genome assembly of Australia’s major tephritid pest species, *Bactrocera tryoni*. The male genome (650 -700 Mbp) includes approximately 150Mb of interspersed repetitive DNA sequences and 60Mb of satellite DNA. Assessment using conserved core eukaryotic sequences indicated 98% completeness. Over 16,000 MAKER-derived gene models showed a large degree of overlap with other Dipteran reference genomes. The sequence of the ribosomal RNA transcribed unit was also determined. Unscaffolded assemblies of *B. neohumeralis* and *B. jarvisi* were then produced; comparison with *B. tryoni* showed that the species are more closely related than any *Drosophila* species pair. The similarity of the genomes was exploited to identify 4924 potentially diagnostic indels between the species*,* all of which occur in non-coding regions.

**Conclusions:**

This first draft *B. tryoni* genome resembles other dipteran genomes in terms of size and putative coding sequences. For all three species included in this study, we have identified a comprehensive set of non-redundant repetitive sequences, including the ribosomal RNA unit, and have quantified the major satellite DNA families. These genetic resources will facilitate the further investigations of genetic mechanisms responsible for the behavioural and morphological differences between these three species and other tephritids. We have also shown how whole genome sequence data can be used to generate simple diagnostic tests between very closely-related species where only one of the species is scaffolded.

**Electronic supplementary material:**

The online version of this article (doi:10.1186/1471-2164-15-1153) contains supplementary material, which is available to authorized users.

## Background

**Figure 1:**
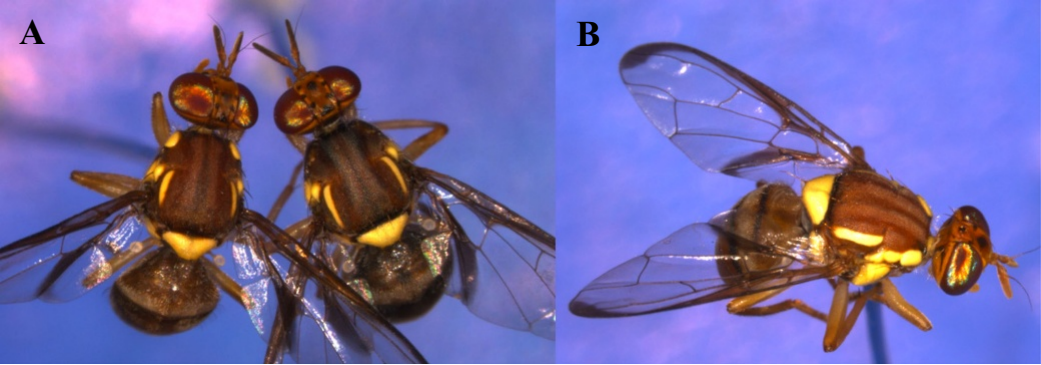
**The** ***Bactrocera*** **species used in the present study. Panel A** shows a *B. tryoni* male on the left and a male *B. neohumeralis* on the right. The species can be distinguished by the colour of the humeral calli (the “shoulder pads”) on the anterior of the thorax, which is yellow in *B. tryoni* and dark in *B. neohumeralis. B. neohumeralis* usually have a darker body colour. **Panel B** shows a male *B. jarvisi*, which is distinguished from the other two species by the extra yellow marking immediately posterior to the humeral callus, the lighter body colour, clear costal cells on the anterior wing margin and abdominal stripes.

The discovery of the genetic processes causing and accompanying speciation has been a long-standing challenge for evolutionary biologists. However, good study systems are rare. Model study systems would ideally include species that are firstly sympatric and secondly very recently diverged, with an effective mechanism of mating isolation. Additionally, the species should be amenable to forced mating in the lab to allow genetic analysis. In this paper, we present genomic resources to underpin the study of three sympatric tephritid fruit fly species that fulfil all these criteria. The three species are shown in Figure 1. For the major species, Queensland fruit fly, *B. tryoni*, we present a draft genome and annotation. For the two other sympatric species, *B. neohumeralis* and *B. jarvisi*, we present unscaffolded draft genomes to establish the genetic relations between the three species.

The economic importance of *B. tryoni* has prompted intense research interest for over 60 years. In Australia, *B. tryoni* is the most serious economic pest of horticulture and a major target for international and domestic quarantine efforts [1]. *B. neohumeralis* and *B. jarvisi* are also pests of economic importance and are sympatric with *B. tryoni* throughout most of tropical Australia [2]. Various *Bactrocera* species are major economic pests of fleshy fruit production in the Asia-Pacific region while ecologically comparable genera are major pests in other parts of the world: *Ceratitis* (including medfly) in Afrotropical regions, *Anastrepha* in Central and South America and *Rhagoletis* in Europe and North America [3].

*B. tryoni* and *B. neohumeralis* are identified by a morphological difference in the colour of the humeral calli (Figure 1) and a behavioural difference in time of mating: *B. tryoni* mates in a narrow window of falling light intensity at dusk, whereas *B. neohumeralis* mates in bright light during the middle of the day [4–6]. A DNA microsatellite-based survey found no evidence of hybridisation between the species in the wild [7]. However, interspecies hybrids can readily be produced in the laboratory by caging males of one species with females of the other (in both directions) [4]. These hybrids are viable and fertile, and can be maintained indefinitely. Not surprisingly, *B. tryoni* and *B. neohumeralis* appear extremely similar in DNA comparisons. Previous sequencing of nuclear and mitochondrial sequences found evidence of only one potential fixed difference in a ribosomal spacer region, ITS2, among a large range of shared polymorphisms [8, 9].

The third species is Jarvis’ fruit fly, *Bactrocera jarvisi*, which is an emergent pest of a number of cultivated fruits in northern Australia. It has a more restricted range of host-fruits [2] and appears to be limited in geographic distribution by the distribution of its main endemic host, *Planchonia careya* [10]. *B. jarvisi* is morphologically quite distinct from *B. tryoni* and *B. neohumeralis* (Figure 1) and has been placed in a different subgenus of the *Bactrocera* [2]. DNA studies have shown that this different subgeneric status may not be warranted, but *B. jarvisi* is sufficiently differentiated that it has formed a convenient outgroup for DNA sequence comparisons between *B. tryoni* and *B. neohumeralis* [8]. Surprisingly, *B. jarvisi* can also be forced to mate with both *B. tryoni* and *B. neohumeralis,* with a substantial proportion of viable, fertile hybrids [11, 12].

Therefore the three Australian pest tephritids, which can be hybridised and subjected to selection experiments, constitute a formidable model system that allows genetic and molecular analyses of a number of traits related to pest status – host fruit preferences, lure and odorant attractancy and invasive potential. Further, the apparent extreme similarity between *B. tryoni* and *B. neohumeralis* provides a model to investigate genome evolution and maintenance of separate species status, and the morphological differentiation of *B. jarvisi* allows investigation of the molecular mechanisms of morphological development and developmental canalisation.

## Results

### Sequencing and *de novo*assembly

The sequence data used for the *de novo* assembly of the male *B. tryoni* genome is summarized in Additional file 1. The *B. tryoni* data consisted of 58 Gbp of paired-end data, representing approximately 80 times coverage assuming a genome size of approximately 700 Mbp (as calculated below). For the related species *B. neohumeralis* and *B. jarvisi* we obtained 62 Gbp and 55 Gbp of 100 bp paired-end Illumina HiSeq data respectively. The assembly of the *B. tryoni* genome was performed using AbySS ver 1.3.4 [13] to construct contigs followed by scaffolding with SSPACE [14], as detailed in the Methods section. The statistics of the resulting assembly are shown in Table 1. Only five scaffolds containing bacterial sequences were detected and these were removed from the assembly. Due to a lack of mate pair data, *B. neohumeralis* and *B. jarvisi* remained unscaffolded.

**Table 1:** **Statistics of the genome assemblies**

	***B. tryoni***	***B. neohumeralis***	***B. jarvisi***
Number of scaffolds or contigs	31957	287119	189607
N50 length	69525	2859	3879
N50 number	1590	72665	36685
Longest	3181581	85617	908794
Total bp	518958810	710077868	582862706

Note that only the *B. tryoni* genome was scaffolded.

To assess the completeness of the coding regions in the genome assembly, we used the CEGMA set of conserved orthologs [15]. Complete sequences were found for 93.6% of the 482 genes that constitute the core gene set. Inclusion of partial matches increased that percentage to 98.8%, indicating that the assembly of coding regions was near-complete. Only 9.9% of matches had more than one ortholog within the assembly suggesting that the assembly contained only a low level of alternate coding assemblies. The mitochondrial genome of *B. tryoni* emerged from the assembly as a single scaffold with 99% identity with a complete *B. tryoni* mitochondrial genome [16], with our scaffold containing a possible 300 bp duplication in the A + T-rich region.

The same CEGMA-based approach was used to assess the completeness of the two unscaffolded assemblies. The *B. neohumeralis* assembly was 84.7% complete (96.4% including partial matches). The *B. jarvisi* assembly was 87.1% complete (95.6% including partial matches). However, for *B. neohumeralis* and *B. jarvisi* 23% and 21% respectively of loci had more than one ortholog. That increase from 9.9% in *B. tryoni* suggested that there were more redundant contigs in both those assemblies.

### Genome size

We first used the k-mer method to estimate genome size [17], using both Jellyfish [18] and DSK 1.6066 [19] to count 18-mers. For a male-only sample of *B. tryoni*, the calculated genome size was 797 Mbp. Recently, a sample of female-only DNA was sequenced (also Illumina Hi-Seq) giving an estimate of 829 Mbp. The larger female genome size was expected on the basis of cytological evidence [20]. However, since these estimates were larger than those expected from other *Bactrocera* genomes [21, 22], we sought alternate estimates of genome size.

**Figure 2:**
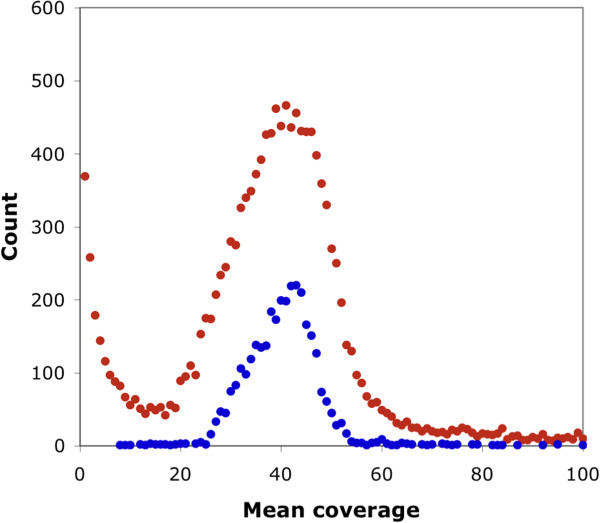
**Coverage of transcripts.** Sequencing reads were mapped to all putative transcripts (red points) and a median group of 3310 transcripts (blue points). The reduced set of transcripts was filtered to exclude repetitive sequences and incomplete sequences. The genome size was estimated from the peak of the lower distribution.

Our alternative approach to the estimation of genome size was based on the coverage of putative transcripts, with the assumption that many transcripts originate from single copy sequences [23]. That assumption was supported in the case of the *B. tryoni* assembly by the low percentage of orthologs (9.9%) in the CEGMA gene set. We first estimated coverage of all 16710 *B. tryoni* MAKER-derived transcripts (see below). We refined this measure to minimise the influence of repetitive sequences and erroneous gene models. Using a subset of 3310 filtered transcripts, we obtained a distinct peak coverage at 42.5 (Figure 2). Variation in stringency of mapping resulted in only a small variation in coverage estimates in the range 41-43. Given that the Illumina HiSeq data contained 29.8 GB of sequence, the estimated genome size was 701 Mbp. This was larger than previous estimates for other *Bactrocera* species of 445 and 619 Mbp, but similar to that of another tephritid *Ragoletis juglandis* [21]. However, if the 9.9% of the CEGMA genes that had more than one ortholog were misassemblies rather than true orthologs, then the method may over-estimate genome size by as much as 10%.

### Repetitive sequence analysis

Analyses of repeated sequences in *de novo* eukaryotic assemblies are sometimes limited to standard implementations of either homology-based searches (e.g. RepeatMasker) or *de novo* prediction (e.g. RepeatModeler [24]). However, we know that the genomes of *B. tryoni*, *B. neohumeralis* and *B. jarvisi* contain abundant insertions of *mariner* elements [25], and that partial sequences of other repeated elements have been found within sequenced introns (for example [26]). Also, satellite DNA sequences receive little attention despite their potential importance to many aspects of genome evolution and regulation [27]. Therefore, we undertook a detailed analysis of the tandem and dispersed repetitive sequences in *B. tryoni*.

We identified *B. tryoni* repetitive sequences from a combination of RepeatModeler *de novo* predictions, a k-mer extension analysis and manual curation as detailed in the Methods section. Initially, RepeatModeler produced 1236 sequences totalling 1.8 Mbp. The k-mer extension analysis was similar to the approach used in [28], in which the highest frequency k-mers found in the raw reads were extended one base pair at a time using the highest abundance k-mers that overlapped the original k-mer by (k-1) bases. The use of raw reads greatly increases the likelihood of finding repeats, such as satellite DNA, that are not easily incorporated into an assembly. Our k-mer extension produced hundreds of fragments in the range 50 – 300 bp. Among the 15 most abundant sequences were five potential satellite DNA sequences. In *B. tryoni*, these sequences ranged from 154 bp to 182 bp, typical of alphoid satellite DNA [27]. For both *B. neohumeralis* and *B. jarvisi*, the k-mer extension analysis identified variants of the same five satellite sequences (Table 2). The k-mer extension approach also refined the RepeatModeler output, which identified the same satellite sequences but in a variety of tandem arrays of 2-5 repeats. The remaining sequences produced by the k-mer analysis were mostly fragments of transposons. Table 3 summarises the classification and abundance of those elements.

**Table 2:** **Abundance of the five main classes of satellite DNA**

	***B. tryoni***			***B. neohumeralis***	***B. jarvisi***
Satellite	Canonical elements	Variable elements	Total Mbp	Variable elements	Variable elements
Btry_Sat1 (166 bp)	68350	250000	41.5	195000	225000
Btry_Sat263 (182 bp)	19682	50000	9.1	37500	111000
Btry_Sat274 (177 bp)	12974	47000	8.3	39800	9500
Btry_Sat2169 (154 bp)	3332	17500	2.7	13000	11000
Btry_Sat2877 (174 bp)	4374	5000	0.9	29000	30000

The Variable Elements count refers the total numbers of elements that vary from the canonical sequence by a single nucleotide substitution, insertion or deletion. Total Mbp is the total length of sequence of that satellite per genome calculated by multiplying the length of the element by the number of variable elements. For *B. neohumeralis* and *B. jarvisi* only the number of variable elements is shown since the genome size was not estimated.

**Table 3:** **Summary of the** ***B. tryoni*** **dispersed repetitive sequences**

	No. of elements	Total Mbp
LINEs	104159	49.18
LTR Retroviral	19836	8.44
DNA transposons	153861	46.29
Unclassified	150385	41.08
Low complexity and simple repeats	7827	0.81

The number of elements was estimated using RepeatMasker with the repeat library prepared in this study.

The genomic arrangement of the putative satellite sequences (tandem arrays, head-to-tail, head-to-head etc) was again investigated in the raw reads. The analysis described in the Methods section showed that these putative satellite sequences were predominantly in large head-to-tail tandem arrays, typical of satellite DNA. Analysis of higher order patterns in the tandem arrays is an objective of future work.

The final step in the identification of *B. tryoni*-specific repeats was a manual curation using an iterative alignment and extension of the remaining RepeatModeler *de novo* sequences. That process identified the consensus of mainly transposon-related sequences. The final output was a set of 153 *B. tryoni*-specific repeat sequences ranging between 52 and 7328 bp in length, with a total length of 249 kb. These sequences were classified on the basis of homology to Dipteran Repbase sequences [29] and are presented in Additional file 2. To estimate proportion of the genome consisting of those 153 repetitive sequences, we mapped the 298 million *B. tryoni* Illumina HiSeq reads to the *B. tryoni*-specific repeats. This suggested that approximately one third of the *B. tryoni* genome consists of repetitive DNA (~31.4% of reads mapped to the repeats with mapping quality *q* > 20, NM = 4.9 where NM is the SAM flag indicating the number of mismatches).

The utility of the *B. tryoni*-specific repeat library can be illustrated by comparing the masking ability of RepeatMasker [30] with and without the *B. tryoni*-specific repeat library. Using RepeatMasker with default parameters and the Repbase repeat library [29], 31 Mbp of the *B. tryoni* assembly was masked. Inclusion of our *B. tryoni*-specific repeat library increased the masking almost 5-fold to 146 MBp. The masking does not increase to 30% of the assembly since most satellite sequences and many transposon fragments were likely to be under-represented in the assembly.

Since *B. neohumeralis* and *B. jarvisi* both show very low sequence divergence from *B. tryoni* (<1%; see section in substitution rates below), homologs of the *B. tryoni* repeat sequences were constructed for the other two species by simply selecting the related repeats and substituting the most common single nucleotide polymorphisms or small indels from the second species. For each species, raw reads were then mapped to the appropriate set of repeats to estimate coverage for the repeated sequences. For *B. neohumeralis*, 33% of Illumina HiSeq reads mapped to the *B. neohumeralis* repeats (average NM = 5.2). For *B. jarvisi*, 37.8% of reads mapped to the *B. jarvisi* repeats (average NM = 4.9). Thus for each of the three species, approximately one third of the raw sequencing reads mapped to the 249 kb of repetitive sequences listed in Additional file 2.

**Figure 3:**
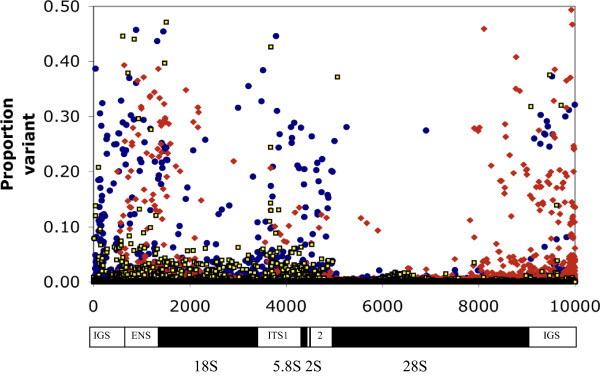
**The comparative frequency of variants across the rRNA locus.** The proportion of sites that vary from the consensus rRNA sequence is shown for *B. tryoni* (blue), *B. neohumeralis* (yellow) and *B. jarvisi* (red). Variation was measured from the consensus sequence for that species. The consensus sequence for *B. jarvisi* is only 9498 bp (rather than 10 kb) due to shorter assembled IGS sequences. The bar below the graph indicates the rRNA unit, with the four rRNA loci shown in black. The limits of the four rRNA loci are based on the *D. melanogaster* sequence. IGS = intergenic spacer (incomplete), ETS = external transcribed sequence, ITS1 and 2 = internal transcribed sequences.

Ribosomal RNA (rRNA) genes are expected to occur in large tandem arrays containing hundreds of copies of the loci. Sequence heterogeneity among the many loci affects assembly from short genomic sequencing reads [31]. Consequently, we manually assembled the consensus rRNA transcribed unit using similar methods to those used to assemble the other classes of dispersed repetitive DNA, an approach previously used with *Drosophila* [31]. The Intergenic Spacer regions (IGS) joining the transcribed units were not completely assembled due their highly repetitive nature. The rRNA consensus sequences for each of the three *Bactrocera* species showed the usual tandem arrangement of 18S, 5.8S, 2S and 28S genes. Transcriptomic assemblies confirmed our assembled sequence of the *B. tryoni* transcribed unit (DCAS, unpublished data). The alignment of the three sequences, along with the *D. melanogaster* rRNA sequence, is included in Additional file 3, which confirms the difference between the *B. tryoni*/*B. neohumeralis* ITS2 found in an earlier study [8]. Figure 3 shows the occurrence of sequence variants for the three *Bactrocera* species (we use “variant” to indicate sequence differences both within and between individuals of each species). In all three species, variation was reduced in the 28S and 18S regions with a greater reduction in the 28S locus. Our results parallel those for *Drosophila* [31], where higher frequency variants (>5%) tended to be concentrated in the Internal Transcribed Spacer (ITS) and IGS regions.

Comparisons between the three *Bactrocera* species showed that *B. tryoni* and *B. neohumeralis* have relatively few inter-species rRNA sequence differences. *B. jarvisi* shows greater differentiation from both *B. tryoni* and *B. neohumeralis*, particularly in the ITS and IGS. The differentiation of the *B. jarvisi* IGS was sufficient to prevent meaningful sequence alignment with the IGS of the other two species.

The amount of sequence variation also differed between the three species (Figure 3). The incidence of variants in the *B. tryoni* sequence reads was almost twice that of *B. neohumeralis*. Variation in the *B. jarvisi* data was as low as *B. neohumeralis* in the transcribed rRNA unit but higher than *B. tryoni* in the IGS and external transcribed spacer (ETS) region. The extra variation in *B. tryoni* was unlikely to have resulted from differences in levels of inbreeding because pooled sequencing data from 12 wild caught individuals showed no increase in heterogeneity over the sequencing data from our highly inbred strain of *B. tryoni* (data not shown), suggesting that the extra heterogeneity exists among the rRNA repeats of any one individual. The selective pressures operating on rRNA sequence variants both between and within individuals that may produce these varying patterns of heterozygosity are unclear.

### Annotation

Annotation of the *B. tryoni* genome was performed using the MAKER pipeline [32]. Evidence use to create gene models comprised *de novo* transcriptomes and gene models from *C. capitata* and *D. melanogaster*. For repeat masking, we used a combination of the Repbase Dipteran library [29] and the *B. tryoni*-specific repeats identified above. Default parameters were used except that potential intron length was extended to 40 kbp.

**Figure 4:**
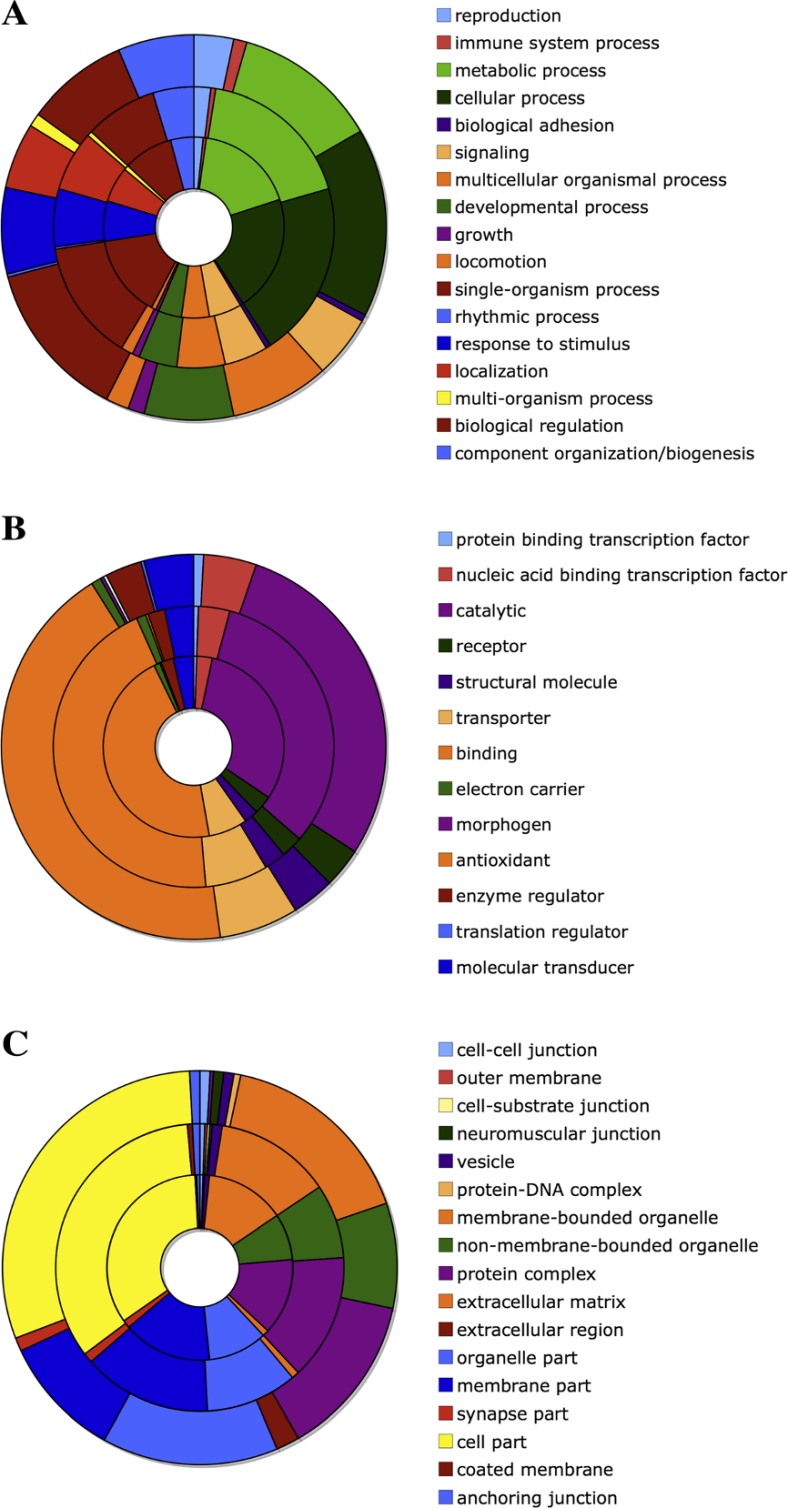
**Gene Ontology comparison.** Each graph compares the classification of the putative *B. tryoni* transcripts (outer ring) with the annotations of *C. capitata* (middle ring) and *D. melanogaster* (inner ring). **Panel A**: Biological processes. **Panel B**: Molecular Function. **Panel C**: Cellular components. The relative distributions support the conclusion that the assembly contains a near-complete assembly of coding DNA.

The set of 16710 gene predictions produced by MAKER were subsequently classified using a local installation of Blast2Go [33]. Of the 16710 input sequences, 14334 produced Blast alignments with an NCBI invertebrate reference sequence library. 9417 of those gene models were successfully annotated with Gene Ontology terms. 11333 sequences were annotated with InterProScan. That same annotation process was repeated for the *C. capitata* and *D. melanogaster* gene models and the comparison of the resulting classifications is shown in Figure 4. The similarity of these classifications suggests that our assembly and annotation are reasonably complete. However, differences in the number of gene models for each species and the greater representation of Drosophilids in the databases used by InterProScan mean that at this stage it would be difficult to reliably interpret the relative differences in the Gene Ontology terms.

### Comparison with other Diptera

To assess the overall composition of the *B. tryoni* gene model set, we compared it to gene model sets from two other Dipterans: *C. capitata* and *D. melanogaster*. Table 4 shows that the sizes (amino acid residues) of the 16710 *B. tryoni* gene models was comparable to *C. capitata* and *D. melanogaster*.

**Table 4:** **Comparison of the number and size range of the gene models for the three Dipteran species shown**

	***B. tryoni***	***C. capitata***	***D. melanogaster***
No. of gene models	16710	20674	26950
Mean length (aa)	593	673	659
Min. length (aa)	21	21	11
Max. length (aa)	17244	20191	22949

**Figure 5:**
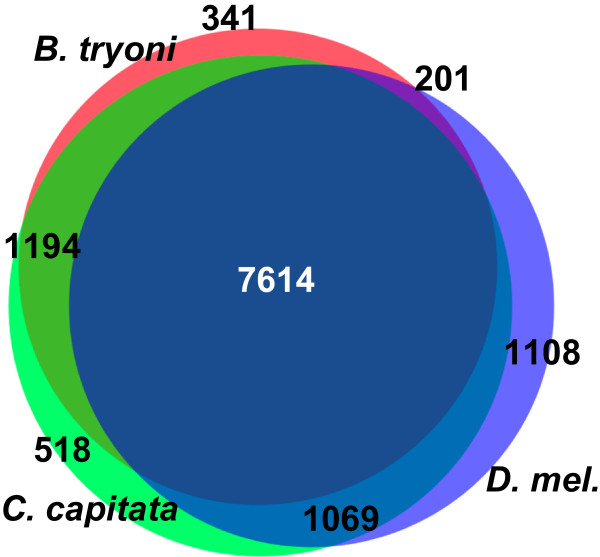
**Overlap of protein orthologous groups for** ***B. tryoni*** **,** ***C. capitata*** **and** ***D. melanogaster*** **.** The gene models for all three species were grouped according to similarity using OrthoMCL. The Venn diagram shows the number of groups that included gene models from either one, two or three of the species. Areas of overlap are proportional to the counts shown. For all three species, 2-4% of gene models occurred in species-specific groups.

To comparatively assess the overall composition of our *B. tryoni* gene models, we used OrthoMCL [34], which groups transcripts by sequence similarity. Although the three sets of gene models contain different numbers of gene models (Table 4) reflecting the different annotation history and stage of curation, this comparison provides an indication of the overall completeness of our assembly. OrthoMCL produced 11688 orthologous groups and Figure 5 shows the overlap of those groups between the three species. Of the orthologous groups, 65% contained representatives from all three species and only 3% of groups were *B. tryoni*-specific. While that high degree of overlap suggests that the *B. tryoni* assembly is reasonably complete, a caveat is that the *C. capitata* and *D. melanogaster* gene models were included in the *B. tryoni* MAKER annotation as part of the evidence used to create the *B. tryoni* gene predictions. Nevertheless, our *B. tryoni* gene models do not appear to be a simple subset of the other two species, since both *B. tryoni* and *C. capitata* are equally distinct from *D. melanogaster* and each other.

**Figure 6:**
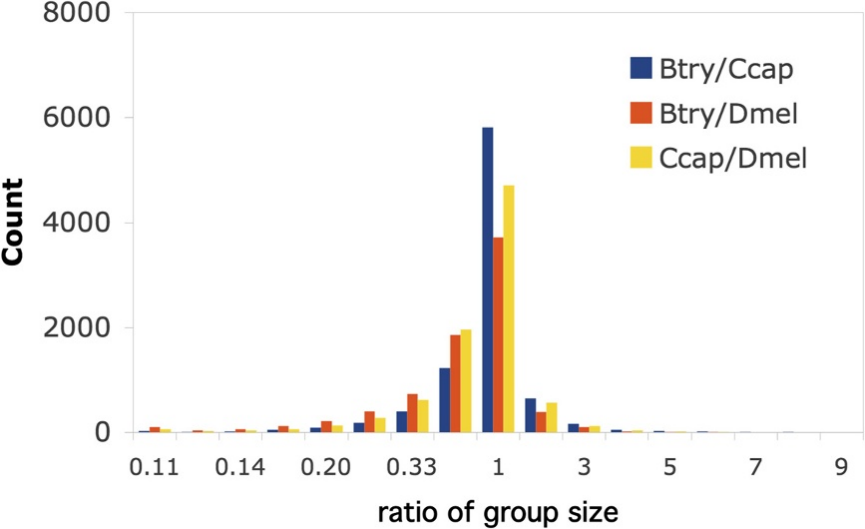
**Relative size of orthologous groups of** ***B. tryoni*** **,** ***C. capitata*** **and** ***D. melanogaster.*** The gene models within each orthologous group produced by OrthoMCL were classified according to species of origin. For each species pair, the ratio of the number of gene models was then calculated on a pairwise basis. The histogram shows the counts for the ratio indicated on the X-axis. For each species pair, the most common ratio was 1:1 indicating a strong correspondence of gene models between species. However, the ratios for *D. melanogaster* in particular are biased downward due to the greater number of gene models available for that species.

Within each ortholog group, closely-related species would be expected to have a similar numbers of gene models. As a result, the ratio of gene models within groups should be approximately 1:1. For each orthologous group, we calculated that ratio for each species pair. Figure 6 shows that, of the three pairwise comparisons, *B. tryoni* and *C. capitata* had the most groups with an approximate 1:1 ratio. Presumably that result is due not only to similarity of the species but also to the similar annotation methods used. Both pairwise comparisons involving *D. melanogaster* showed more groups with a 1:2 ratio rather than a 1:1 ratio. This must be due in part to the greater number of gene models available for *D. melanogaster*. Despite that bias, 80-84% of ratios were in the range 1:3 to 1:1, indicating that most ortholog groups contain similar number of gene models.

The possibility of gene enrichment was investigated in the 341 orthologous groups that contained only *B. tryoni* gene models. Those groups contained 1211 gene models (median sequence length = 150 amino acids), which were annotated with Gene Ontology and Pfam terms using Blast2Go. Of the longer sequences, less than 50% were either fragments of gene models in multi-species ortholog groups or transposon-related sequences. The possibility that the remaining gene models represent novel *B. tryoni* genes is a subject of future investigation.

### Substitution rates between *Bactrocera*species

The small genetic distance between *B. tryoni* and *B. neohumeralis* was reflected in the nucleotide substitution rates between the two species. We partitioned fixed inter-species nucleotide substitutions between exons, introns, UTRs and non-coding DNA, with exon substitution rates being further classified as synonymous or non-synonymous (Table 5). The observed substitution rates between *B. tryoni* and either *B. neohumeralis* or *B. jarvisi* were lower than that reported between, for example, *Drosophila* species (e.g. [35]). The non-synonymous versus synonymous ratio d_N_/d_S_ (or K_A_/K_S_) was approximately 0.1, which is consistent with some degree of purifying selection affecting each species.

**Table 5:** **Substitution rates in** ***B. neohumeralis*** **and** ***B. jarvisi*** **in comparison to the** ***B. tryoni*** **assembly**

Region	***B. neohumeralis***	***B. jarvisi***
Exon	0.0032	0.0197
Intron	0.0061	0.0295
5’	0.0036	0.0259
3’	0.0026	0.0221
Non-coding	0.0072	0.0233
Non-synonymous substitutions	0.0012	0.0065
Synonymous substitutions	0.0021	0.0121
Novel stop codons	190	1329

In the raw *B. neohumeralis* Illumina HiSeq data, 197 instances of apparently novel stop codons were found (Table 5; Additional file 4). The majority of these occurred either in alternate transcripts (45) or sequences that had no Blast results (70), while some stop codons were close to the end of the *B. tryoni*-version of the transcript. Differential expression of these is currently under investigation as part of a comparative transcriptomics project (K. Raphael pers. comm.). During investigation of these potential novel stop codons, we also found 30 instances where a substitution, if considered in isolation, would have produced a new stop codon. However, in each of those 30 potential transcripts, a second substitution in the same codon negated the effect of the first substitution.

The close similarity between the species at the sequence level allowed Illumina HiSeq data from both *B. neohumeralis* and *B. jarvisi* to be mapped against the *B. tryoni* assembly. For *B. neohumeralis*, 70.4% of reads mapped to the *B. tryoni* assembly with a mapping quality *q* > 20. By comparison, only 41.8% *B. jarvisi* reads mapped to the *B. tryoni* assembly with quality *q* > 20.

### Indel variation between *Bactrocera*species

Since we did not have scaffolded assemblies for either *B. neohumeralis* or *B. jarvisi*, we were unable to reliably investigate syntenic differences between the species. However, the sequence similarity was exploited to identify a large number of deletions in the *B. neohumeralis* and *B. jarvisi* sequence data relative to the *B. tryoni* scaffolds. Gapped alignment programs (e.g bwa-mem) were used to identify *B. tryoni* scaffold segments with zero coverage. These were then filtered to extract only those deletions with high, precisely aligned coverage on both sides of the deletion. For *B. neohumeralis*, of 59633 initial deletions over 10bp, only 4924 had sufficient coverage on both flanks to be investigated further. For *B. jarvisi*, 770662 initial deletions were reduced to 57285.

Since transposons may play a significant role in sequence evolution, species as closely related as *B. tryoni* and *B. neohumeralis* provide an opportunity to investigate the possible involvement of transposons in the earliest stages of genome divergence. Using the deletions identified above, we extracted the 1000 bp genomic segments adjacent to each of the deletions identified in *B. neohumeralis* and *B. jarvisi*. A Blastn search of these flanking sequences, using the species-specific repeat library (requiring 80% identity over 80 bp length for a match), showed that 32% of sequences contained repetitive elements. However, this level of association between repetitive sequences and the deletions was not significant. Using the same Blastn parameters, 1000 sets of the same number of random genomic sequences (separated by the same distance) produced an average of 30.1% repeat hits. While that result coincided with our estimate of approximately 30% repetitive DNA in the *B. tryoni* genome, that figure was only coincidental since the repetitive DNA is not well represented in the assembly. Similarly, the same comparison between *B. tryoni* and *B. jarvisi* also showed that repetitive DNA was not preferentially associated with deletions.

**Figure 7:**
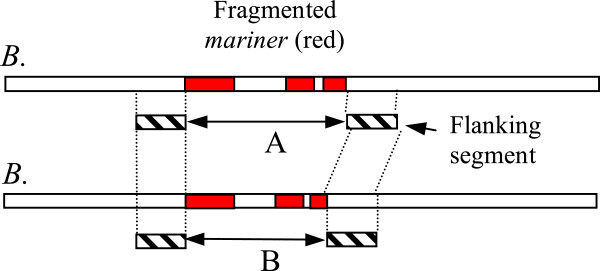
**The strategy used to detect variation between** ***Bactrocera*** **species in the size of** ***mariner*** **transposon sequences**. Fragments of *mariner* transposon sequences were counted as a single element if the fragments covered less than twice the length of the canonical transposon sequence and there were no other fragments for the same distance on either side. The two 1000 bp genomic flanking segments were extracted from *B. tryoni* and the homologous segments were identified in *B. neohumeralis* or *B. jarvisi*. The size of the element (**A** and **B** above) was then compared between species.

**Figure 8:**
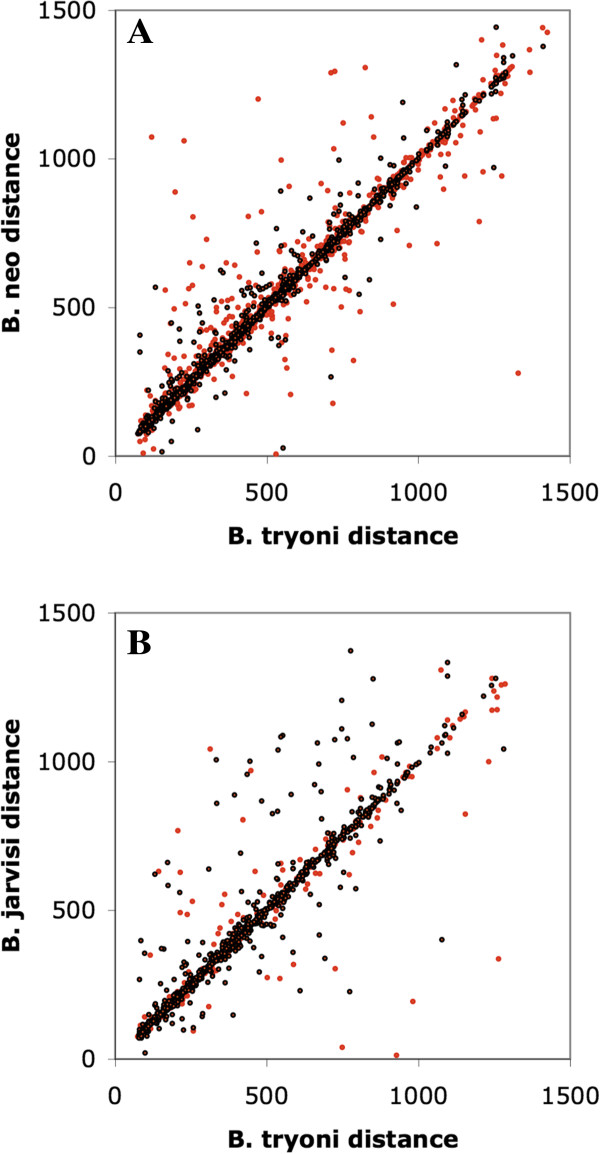
**The comparison of the overall size of homologous transposon.** Insertion sites in *B. tryoni* /*B. neohumeralis* **(A)** and *B. tryoni* /*B. jarvisi* **(B)**. For each species pair comparison, homologous DNA segment size varies more in the vicinity of transposon (*mariner*) sequences (red points) than at random sites (black points). The control group consisted of an equivalent number of sequence fragments drawn at random from the two relevant assemblies.

We also observed that many transposon sequences in the *B. tryoni* scaffolds were in homologous positions in both the *B. neohumeralis* and *B. jarvisi* contigs. We identified all *mariner* transposon sequences in the *B. tryoni* scaffolds, these were fragmented and often contained intervening non-transposon sequences. Any other *mariner* fragments within 1.5 kb (the average length of the *mariner* elements) were considered to be part of the same element. We then extracted the non-transposon sequences flanking each *B. tryoni* composite element and measured the length of the transposon insertion (i.e the distance between the two flanking segments). The distance between the homologous flanking sequences was then measured in *B. neohumeralis* or *B. jarvisi* (where the flanking sequences were on the same contig). This approach is illustrated in Figure 7. To assess the significance of any between-species differences, we extracted an equal number of random, paired *B. tryoni* sequences, with the insert sizes matching the gaps in the actual *B. tryoni* flanking sequence pairs. The results of this are shown in Figure 8A and 8B. For both species pairs, the mean insert size was larger for the *mariner* sequences than control sequences (*B. tryoni* /*B. neohumeralis* 17 bp vs. 9 bp: 2-tailed t-test, p < 0.001; *B. tryoni* /*B. jarvisi* 98bp vs. 29 bp: 2-tailed t-test, p < 0.001) and the variances were correspondingly higher. This suggests that *mariner* sequence insertions are associated with a small increase of intra-species sequence variation. Similar comparisons for other repetitive elements are currently underway.

## Discussion

The family Tephritidae consists of over 4000 species in over 400 genera [36], including major global economic pests [37]. Many of these species, including the *Bactrocera*, have arisen relatively recently in evolutionary terms [21]. Tephritids provide numerous promising study systems for speciation, behaviour, invasiveness and sex determination [38–40]. The present study presents a first set of *Bactrocera*-specific resources that will assist genomic and genetic studies in all these areas. In addition to the first draft of an annotated assembly of the *B. tryoni* genome, we have produced an extensive non-redundant library of *B. tryoni* repetitive DNA. Such a library not only assists annotation but also provides avenues for investigation of genome evolution. We have also delineated the majority of satellite sequences and have provided a complete assembly of the rRNA repeat unit, both of which are often lacking from *de novo* genome assemblies.

The close genetic similarity of *B. tryoni* and *B. neohumeralis* has attracted the attention of evolutionary biologists since the 1960s. This study quantified that similarity over the majority of the genome and confirmed previous first-generation sequencing studies of specific nuclear and mitochondrial regions [8, 9, 41]. The only differences identified in those earlier studies were two single-nucleotide substitutions and a trinucleotide indel within the ribosomal gene internal transcribed spacer (ITS2), a level of differentiation more commonly observed between populations than between reproductively isolated species. Those differences were confirmed in the present study.

Our results have highlighted the potential for speciation studies in the *Bactrocera* genus. The sequence difference between *B. tryoni* and *B. neohumeralis* is considerably less than that found between species of *Drosophila* [35]. The closest species pairs are *D. pseudoobscura* - *D. persimilis*, a cross which produces sterile males [42] and *D. simulans* - *D. mauritiana*. The estimated synonymous substitution rates for these two pairs are around 1.5% [43] and 4.7% [44], respectively, compared to our estimate of 0.21% for *B. tryoni* and *B. neohumeralis*. The estimate for *B. tryoni* and *B. neohumeralis* is sufficiently low as to be comparable with the extent of variation between strains of *D. melanogaster*. It is, however, difficult to measure genome-wide polymorphism levels with the currently available data. The *B. tryoni* strain used for sequencing was established from a mutant individual [45] and subsequently subjected to two further rounds of single-pair inbreeding to reduce polymorphism and facilitate assembly. The *B. neohumeralis* strain was not systematically inbred, but its establishment and maintenance as a laboratory strain for several years may have contributed to a loss of variability. Questions about the extent of polymorphism within and between the two species can only properly be answered by sequencing unrelated individuals from wild populations.

The close similarity between *B. tryoni* and *B. neohumeralis* is consistent with the fact that, despite the complete absence of wild hybrids [7], hybrids between the two species are viable and fertile, with only marginal reductions in fitness [4]. Somewhat surprisingly, laboratory hybrids between *B. jarvisi* and *B. tryoni* are also viable and fertile, despite the much greater genetic distance between the two (yet still comparable to the *D. pseudoobscura* - *D. persimilis* pair). In this case, the fertility of male hybrids is only slightly reduced (~80% of non-hybrids) and the sex ratio of hybrid crosses in both directions is slightly biased. Crosses between *B. jarvisi* and *B. tryoni* may produce up to 70% females in some crosses (Gilchrist, unpublished observation). Viable and fertile hybrids can also be produced by forced mating between *B. neohumeralis* and *B. jarvisi* (Gilchrist and Belanto, unpublished observation). The potential use of hybrids, together with the genome information, provides a powerful system for investigating the genetic basis for all identifiable phenotypic differences between the three species, including mating-time differences and morphological divergence. The degree of genetic similarity between *B. tryoni* and *B. neohumeralis* is unprecedented amongst the closely-related dipteran species studied to date, thereby providing a model for investigating the earliest stages of speciation. This is a field in which good study systems are extremely rare [46–51].

We also point the way to overcoming the problem of diagnosis: in the past, the similarity between *B. tryoni* and *B. neohumeralis* prevented the development of diagnostic genetic markers for use in hybridisation studies and quarantine testing. Here, we present over 4000 small indels that, subject to studies in outbred wild populations, could provide the basis for new genetic markers. These deletions are currently being investigated as a basis for a simple PCR-based test to allow quarantine authorities to differentiate *B. tryoni* and *B. neohumeralis* (since there is currently no reliable test to split the two species at the larval stage).

The methods we have used can be applied to any of the closely-related species groups which are challenging for traditional morphological taxonomy (e.g. the *B. dorsalis* species complex; [39]). Our method requires only a single assembled reference genome for comparison with raw sequencing reads from sibling species.

## Conclusions

This paper presents a comprehensive first draft genome of an important horticultural pest, the Queensland fruit fly or Q-fly, *B. tryoni*. Although endemic to Australia, *B. tryoni* is an insect pest of worldwide concern, particularly in these times of burgeoning movement of people and produce. Further, it represents the first annotated and published genome of a species in the genus *Bactrocera*, which contains the great bulk of tephritid pests in Asia and Oceania. Our data provides an important basis for comparison with the genome of *C. capitata* and with other tephritid genome projects, for functions related to host-fruit recognition, invasive potential and developmental regulation. Our data will also have direct application to the delineation of recent radiations such as the *B. dorsalis* species complex found in Pacific and south-east Asian countries [39].

The *B. tryoni* genome has also enabled analysis of the genomes of two related *Bactrocera* species, *B. neohumeralis* and *B. jarvisi*. These are of interest because they are clearly distinct species with different behaviours leading to strong pre-mating isolation. Yet, since hybrids between these species are fertile, they present an unusually powerful model for investigating the genetic bases of morphological development, the evolution of morphological change and the molecular aspects of pest status and behaviour.

## Methods

### Sample collection and DNA extraction

The *B. tryoni* strain used for sequencing was the *bent wings* (*bw*) strain [45]. The *bw* mutation was isolated from a single wild fly caught near Gosford, NSW, Australia, bred for homozygosity of the marker and maintained in the lab for 10 years (approximately 80 generations) before being further inbred by two rounds of single pair matings. The *B. neohumeralis* and *B. jarvisi* strains used for sequencing were caught near Cairns, Queensland, Australia in 2006 and maintained in lab culture since that time. Neither were deliberately inbred as was the *B. tryoni* strain. Genomic DNA for each sequencing run was extracted from 20 male fly heads using the method as described in [26].

### Genomic sequencing

For Illumina HiSeq runs, libraries were prepared with a commercial kit (Paired-End DNA Sample Prep Kit; Illumina Inc., San Diego, CA, USA) following the manufacturer’s protocol (Paired-End Library Construction). For *B. tryoni*, both Illumina paired-end sequencing (GAIIx, HiSeq and MiSeq) and 454 FLX Titanium pyrosequencing were performed at the Ramaciotti Centre at the University of NSW. For the mate-pair data, two Illumina GAII runs were performed at the Ramaciotti Centre at the University of NSW (3 kb insert), while 1 lane of Illumina HiSeq mate-pair data (10 kb insert) was generated at the Hawkesbury Institute for the Environment, University of Western Sydney. Additionally, a long jumping distance (LJD) mate-pair library (8 kb insert) was prepared by Eurofins MWG Operon, Ebersberg, Germany. The quality of paired-end data was assessed using FASTQ and subsequent quality trimming performed with the Trimmomatic software [52]. The Eurofins LJD library was quality trimmed by that company. Quake software [53] was used to remove and/or correct singleton 18-mers only in the Illumina HiSeq paired-end data (other data not having sufficient coverage).

For *B. neohumeralis* three lanes of 100 bp paired-end Illumina HiSeq data were obtained, totalling 62 Gbp. Two of the libraries were prepared with 300 bp insert size and one with 500 bp insert size. For *B. jarvisi*, two lanes of 100 bp paired-end Illumina HiSeq data were used, one with 300 bp inserts and the other with 500 bp inserts. The male-only data totalled 55 Gbp.

### Assembly

After Quake-correction [53] of the Illumina HiSeq paried-end data, the *B. tryoni* contigs were generated from the paired-end GAII, paired-end Illumina HiSeq, paired-end Illumina MiSeq and Roche 454 FLX Titanium reads using the AbySS assembly program [ver 1.3.4 ; 13] with overlap length *k* = 65. The *k* value was determined after testing values ranging from 40-70. Contigs greater than 210 bp were retained for scaffolding. Scaffolding was performed using the SSPACE scaffolder [14]. The three mate-pair libraries were added in order of increasing insert size (3 kb, 8 kb and 10 kb) as advised by the SSPACE authors. We screened for the presence of bacterial sequences in our assembly using a Blastn search against the NCBI NT database (e-value 1e-06). When any of the top three hits were bacterial, the scaffold was removed. CEGMA was run according to the authors’ instructions at http://korflab.ucdavis.edu/datasets/cegma/. The *B. tryoni* scaffolds have been submitted to NCBI, with accession number JHQJ00000000.

### Genome size estimation

We estimated genome size using two methods. First, we used the k-mer coverage methods (e.g. [17]), using both Jellyfish [18] and DSK [19] to count k-mers. Second, we used a variation of the approach of [23] to estimate genome size from the coverage of single copy sequences. The first step in that process was to map all Illumina HiSeq reads to the 16710 MAKER-derived transcripts using bwa-mem [54] and filtering out reads that mapped with low-quality (*q* < 30). The low coverage transcripts (coverage < 10) comprised 16% of transcripts and were likely to include many truncated or erroneous transcript predictions. High coverage transcripts (coverage > 60) comprised 14.6% of transcripts and were dominated by transcripts including highly repetitive sequences. The central peak of the distribution indicated a mean coverage of 41-43.

To refine this estimate, we re-analyzed the coverage of the transcripts for the median 50% of transcripts ranked by coverage. Genomic scaffold segments corresponding to each transcript were extracted along with the flanking 100bp regions. Genomic segments were used for read mapping as they had no intron/exon boundaries and thus provided longer contiguous regions for mapping. Genomic segments containing runs of five or more Ns were removed, as were transcripts with a MAKER-derived AED score > 0.2 (indicative of transcripts with lesser evidential support). This resulted in a final set of 3310 genomic segments corresponding to individual putative transcripts. Raw reads were again aligned to the set of 3310 transcripts with a mapping quality filter of *q* = 55 to ensure mappings were unique. Coverage was measured using BEDtools utility genomeCoverageBed [55], excluding the 100 bp flanking regions. Repetitive sequences in introns resulted in small regions of very high coverage, 10-1000 times above the median. Consequently, the median coverage rather than the mean coverage was used. Variation of the mapping quality filter (e.g. *q* as low as 10 or 20) resulted in only small changes in the coverage estimates.

### Repetitive sequence analysis

RepeatModeler was run on the final *B. tryoni* assembly following the authors’ instructions. The 18-mer extension method began with Jellyfish program [18], which was used to estimate 18-mer frequencies in the uncorrected Illumina HiSeq data. Using custom Perl scripts, we started with the most common 18-mer, which was extended by a single base pair after finding the next most common 18-mer that overlapped the starting 18-mer by 17 bp. This was repeated in both directions until an already-used 18-mer was met. No 18-mer was used more than once and only sequences that extended more than 50bp were retained for further analysis. The process was terminated after the 50000 most abundant 18-mers had been either been extended or eliminated due to inclusion in a previous extension sequence.

### Genomic arrangement of satellite sequences

Since satellite sequences do not assemble easily, their arrangement in the genome (i.e. as tandem repeats or dispersed elements) was assessed in the 100 bp Illumina reads rather than in the subsequent genome assembly. Although no single 100 bp read will cover a whole satellite monomer, if satellites are arranged in head-to-tail tandem arrays, then a predictable proportion of reads should cover the tail of one repeat unit followed by the head of the next repeat in the array. For example, two 12-mers at positions 1 and 50 in the satellite sequence should always appear 50 bp apart in the same 5’-to-3’ orientation. Conversely, two 12-mers that are more than 88 bp apart, should rarely co-occur. Additionally, 12-mers near the 3’ end of the satellite sequence should always occur in the negative direction (i.e. upstream) of the 1-position 12-mer. Other arrangements, such as head-to-head or dispersed, will not show that same pattern. While we used 18-mers in other sections of this study, we used 12-mers to speed counting. Additional file 5 shows that ~92% of 12-mers from Btry_Sat1 were at the distance and in the orientation expected if Btry_Sat1 occurred mainly in large tandem arrays. Similar evidence was found that the other four satellite sequences were also present mainly as head-to-tail tandem arrays.

### Satellite sequence copy number

To obtain a better estimate of satellite content of the *B. tryoni* genome, we estimated the frequency of satellite sub-sequences in the raw reads. We first counted the frequency of the 18-mers that comprise each of the six satellite sequences in the raw reads. Adjusting for coverage of the Illumina HiSeq reads, the mean frequency of all the non-overlapping 18-mers from each satellite gives an estimate of the number of genomic copies of the canonical sequence (Table 3). If searches allowed for variation by a single nucleotide substitution, more satellite sequences were detected. The data shown in Additional file 5, which includes single nucleotide substitutions, showed that the estimate of the number of Btry_Sat1 sequences (indicated by reads that had two distinct 12-mers at the correct spacing and in the correct orientation) increased from ~70,000 to over 250,000. Those variant sequences were also mainly contained within tandem arrays. For example, in Btry_Sat1, variants of the first and second 12-mers should co-occur on the same 100 bp read at a frequency of 0.87. This is the proportion of all the 100 bp reads that contain 12-mer 1 and will also extend sufficiently in the 3’ direction to include all of the adjacent 12-mer 2. For each million occurrences of Btry_Sat1 12-mer 1 in the Illumina HiSeq reads, we observed ~700,000 occurrences of 12-mer 2 on the same read.

### Dispersed repeats

Dispersed repetitive sequences commonly consist of many fragments of canonical transposon sequences, with various degrees of sequence heterogeneity amongst the fragments. This inherent heterogeneity prevents good assemblies of those sequences. However, canonical versions of repeated sequences can be reconstructed directly from the sequencing reads e.g. the construction of *Drosophila* rRNA sequences [31]. To identify as many as possible of the underlying canonical sequences, we undertook a manual curation of the remaining RepeatModeler *de novo* sequences and the 18-mer extension sequences. That process began by performing a Blastn alignment of all the RepeatModeler *de novo* sequences against the *B. tryoni* scaffolds (80% identity, e-value 1e-06). Starting with the fragment with the highest number of hits, we then performed an iterative process of alignment and consensus extension of the sequences. The process worked as follows. Each potential repeat sequence was aligned to the *B. tryoni* scaffolds using Blastn (80% identity, e-value 1e-06). For each alignment, a custom Perl script extracted the matching scaffold (genomic) sequence along with up to 200 bp of flanking sequence. Those extracted segments (often numbering several hundred) were aligned with Muscle [56] using the SeaView alignment viewer [57] allowing gapped alignments and 60% consensus threshold. The starting sequence could then be extended by up to 200 bp depending on the length of the valid consensus sequence. The process could then be iterated until no further consensus extension was possible. Terminal inverted repeat sequences produced a common sequence linked to two (or sometimes more) consensus sequences. In those cases, each consensus was extended separately and pairs would eventually overlap if the original terminal inverted repeat sequences came from a similar repetitive element. The final extended sequence was then aligned with the remaining RepeatModeler *de novo* sequences (Blastn, 80% identity). This resulted in the culling of numerous related RepeatModeler *de novo* sequences, which in turn reduced the number of sequences that had to be examined. Eventually, the manual curation process was no longer feasible due to a combination of too few aligned genomic segments and increasing heterogeneity among those segments. Also, if taken too far, the ‘align and extend’ process could eventually start identifying conserved protein motifs as repetitive elements.

To finalise the consensus sequence, the most common variant at each SNP was checked by mapping the Illumina HiSeq reads to the candidate repeats using bwa-mem [54], extracting variant frequencies from the Samtools mpileup file [58] using VarScan 2 [59]. Sequences with greater than 80% identity to an existing repeat sequence were removed. Lastly, the percentage of raw reads mapping to those repeats with mapping quality *q* > 20 was calculated from the alignment file. As a relative indicator of variation, the average number of mismatches for the mapped reads was calculated from the NM flag in the bwa-mem output.

### Species-specific repeat variants

We first mapped reads from each species to the *B. tryoni* repeats, retaining reads with mapping quality *q* > 20. SNPs with >50% frequency were extracted from the Samtools mpileup file [58] using VarScan 2 [59]. Those variants were then incorporated into the *B. tryoni* repeat sequences to produce a species-specific set of homologous repeats. Raw reads from each species were aligned to the appropriate set of repeats to estimate coverage for the repeats. Statistics were calculated only for reads with mapping quality *q* > 20.

### Ribosomal RNA sequences

The ‘align and extend’ process used to construct the canonical dispersed repeat sequences was also used to determine the sequence of the transcribed section of the rRNA repeat unit. The IGS sequence was incomplete and limited to the regions flanking the transcribed unit. The highly variable and repetitive nature of the IGS prevented the complete extension of the IGS sequence. All sequence variants for the transcribed rRNA unit and flanking IGS regions were extracted from the equivalent coverage of raw Illumina HiSeq data. Exact delineation of the rRNA genes was based on the *D. melanogaster* homologues [31].

### MAKER Annotation

Within the MAKER pipeline, we used three gene predictors: SNAP [60], AUGUSTUS 2.5.5 [61] and GENEMARK [62]. SNAP was trained using the set of conserved genes identified by the CEGMA pipeline. The training for AUGUSTUS was generated using the whole *B. tryoni* genome, now available as a web-based service (http://bioinf.uni-greifswald.de/bioinf). GeneMark was self-trained. EST evidence was provided in the form of five *B. tryoni de novo* transcriptome libraries produced by the Trinity software [63]. Four of the libraries were assembled using separate RNA preparations from whole embryonic, larval, late pupal and adult individuals (D. Shearman unpublished). The fifth library was prepared from brain tissues. Protein homology evidence for MAKER consisted of coding sequences from two other Dipterans: *D. melanogaster* (ftp://ftp.flybase.net/releases/FB2013_06/dmel_r5.54/fasta/dmel-all-CDS-r5.54.fasta.gz) and medfly, *Ceratitis capitata* (ftp://ftp.ncbi.nlm.nih.gov/genomes/Ceratitis_capitata). We installed a local database for use with Blast2Go. Using BLASTP (e-value = 1e-05), the sequences were aligned to the NCBI RefSeq invertebrate protein database (ftp://ftp.ncbi.nih.gov/refseq/release/invertebrate/). Additional annotations were sourced from the Pfam protein domain database using InterProScan 4.8 [64].

### Comparisons of gene models

For a comparative assessment of the overall composition of the *B. tryoni* gene models, we used OrthoMCL to compare the *B. tryoni* gene models to the gene models available for two other Dipterans: *C. capitata* and *D. melanogaster*. The genome of *D. melanogaster* has been thoroughly annotated and consequently has the largest set of gene models of the three species (Table 4). In contrast, the recent annotation of *C. capitata* is unpublished but the methods used to generate the underlying gene models were similar to those used in this study. Both used trained Augustus as well as SNAP and Genemark and similar multi-staged transcriptome evidence. A major difference was that our gene models were MAKER-based, while those of *C. capitata* were produced using JAMg (http://jamg.sourceforge.net). Nevertheless, the number of gene models for *C. capitata* was closer to that of *B. tryoni* (20674 versus 16710 gene models) and probably contained a similar proportion of splice variants. Nevertheless, each of the three sets of gene models are at different stages in their curation and so the present analysis was only intended to indicate the completeness if the *B. tryoni* genomic assembly, not the transcriptomes.

OrthoMCL compares gene models from different organisms, producing groups of protein orthlogs based on sequence similarity. OrthoMCL was run on the three sets of gene models using using default parameters, except for the Blastp all-against-all step, where a more stringent e-value of 1e-10 was used.

### Comparisons with *B. neohumeralis*and *B. jarvisi*

Substitution rates were measured by first aligning all *B. neohumeralis* or *B. jarvisi* reads to the *B. tryoni* assembly using the bwa-mem aligner. Homozygous SNPs for each species comparison (*B. tryoni*/*B. neohumeralis* and *B. tryoni*/*B. jarvisi*) were extracted from the Samtools mpileup file [58] using VarScan 2 [59] at sites with a minimum coverage of 10. Custom Perl scripts were used to classify each variant position as exon, intron, 5’ UTR, 3’ UTR or non-coding, using the scaffold-specific MAKER-derived gff3 files. For coding regions, synonymous versus non-synonymous substitution rates were also extracted from the MAKER-derived gff3 files using the phase information. Indels of 1 or 2 bp were counted as non-synonomous changes.

Deletions in the *B. neohumeralis* or *B. jarvisi* genomes (with respect to the *B. tryoni* assembly) were identified by first mapping reads from the two other species to the *B. tryoni* assembly using bwa-mem, which allows gapped alignments. The only alignments retained were those that both consisted of paired sequencing reads and had a mapping quality greater than 20. The BEDtools utility genomeCoverageBed [55] was used to identify all *B. tryoni* genomic intervals over 10 bp with zero coverage by either *B. neohumeralis* or *B. jarvisi* Illumina HiSeq data. To further reduce any false positives due to regions of low coverage, only those deletions with >20x coverage for all the 10 bases immediately bordering both sides of the deleted segment were used in further analysis.

A gapped aligner allows even short segments of *B. neohumeralis* or *B. jarvisi* reads (19bp with a seed length of 19) to be mapped to *B. tryoni* even if the remainder of the read does not match. That had the advantage of maximising the coverage of the homologous sequences on either side of any *B. neohumeralis* or *B. jarvisi* deletion, which in turn reduced the likelihood of mistaking low mapping coverage with a real deletion. Conversely, this limited our ability to identify short deletions as some reads will align over short gaps and/or mismatches. However, since we were primarily interested in identifying deletions in order to develop PCR-based species identification tests, we did not quantify deletions under 10 bp.

### Availability of supporting data

The data set supporting the results of this article is available in the NCBI Bioproject repository, project ID 241080 http://www.ncbi.nlm.nih.gov/bioproject/?term=PRJNA241080.

## Electronic supplementary material

Additional file 1: **Summary of the data used for the** ***B. tryoni*** **genome assembly.** (DOC 38 KB)

Additional file 2: **A fasta file of** ***B. tryoni*** **repetitive sequences.** (DOC 543 KB)

Additional file 3: **Alignment of rRNA sequences.** The alignments of the three *Bactrocera* rRNA sequences, along with the *D. melanogaster* rRNA sequence. The 18S, 5.8S, 2S and 28S regions are indicated on the *B. tryoni* sequence by blue highlighting. The red highlight indicates the *B. neohumeralis* insertion identified in an earlier study [8]. (DOC 131 KB)

Additional file 4: ***B. neohumeralis*** **transcripts with novel stop codons.** (DOC 359 KB)

Additional file 5: **The spacing of satellite DNA 12-mers in the 100bp reads.** For the satellite sequence Btry_Sat1 (166 bp in length), the histogram shows the frequency distribution of the spacing between the 12-mer beginning at position 1 of the canonical sequence and other 12-mers from the same satellite sequence that are close enough to co-occur on the same 100 bp read. The 12-mers in the 3’ direction start at positions 13, 25, 37 etc, while the 12-mers in the 5’ direction start at -11, -23 etc. The distance expected from the canonical sequence is shown above each distribution. In each case, the >92% of the 12-mers occur at or near the expected distance and in the expected direction, which is consistent with the existence of large tandem repeat arrays. The 13 separate frequency distributions are non-overlapping at this scale and are therefore presented as one histogram. The frequencies decrease with absolute distance from the 1-position 12-mer due to the decreasing number of 100 bp reads that span both 12-mers. Some decrease is also due to cumulative sequence variation. In all cases, 12-mers were scored as matching if they differed from the canonical sequence by no more than a single 1 bp substitution. (DOC 61 KB)

## References

[1] Drew RAI (1989). The tropical fruit flies diptera tephritidae dacinae of the australasian and oceanian regions. Memoir Queensl Mus.

[2] Osborne R, Meats A, Frommer M, Sved JA, Drew RAI, Robson MK (1997). Australian distribution of 17 species of fruit flies (Diptera: Tephritidae) caught in cue lure traps in February 1994. Aust J Entomol.

[3] White IM, Elsom-Harris MM (1992). Fruit flies of economic significance: their identification and bionomics.

[4] Lewontin RC, Birch LC (1966). Hybridization as a source of variation for adaptation to new environments. Evolution.

[5] Tyschen PH, Fletcher BS (1971). Studies on the rhythm of mating in the Queensland fruit fly, *Dacus* (*Bactrocera*) *tryoni*. J Insect Physiol.

[6] Smith PH (1979). Genetic manipulation of the circadian clock’s timing of sexual behaviour in the Queensland fruit flies, *Dacus tryoni* and *Dacus humeralis*. Physiol Entomol.

[7] Gilchrist AS, Ling AE (2006). DNA microsatellite analysis of naturally occurring colour intermediates between *Bactrocera tryoni* (Froggatt) and *Bactrocera neohumeralis* (Hardy) (Diptera : Tephritidae). Aust J Entomol.

[8] Morrow J, Scott L, Congdon B, Yeates D, Frommer M, Sved J (2000). Close genetic similarity between two sympatric species of tephritid fruit fly reproductively isolated by mating time. Evolution.

[9] Wang Y, Yu H, Raphael K, Gilchrist AS (2003). Genetic delineation of sibling species of the pest fruit fly *Bactocera* (Diptera: Tephritidae) in Australia using microsatellites. Bull Entomol Res.

[10] Fitt GP (1981). Responses by female dacinae to male lures and their relationship to patterns of mating behavior and pheromone response. Entomol Exp Appl.

[11] Cruickshank L, Jessup AJ, Cruickshank DJ (2001). Intersprecific crosses of *Bactrocera tryoni* (Froggatt) and *Bactrocera jarvisi* (Tryon) in the laboratory. Aust J Entomol.

[12] Shearman DCA, Frommer M, Morrow JL, Raphael KA, Gilchrist AS (2010). Interspecific hybridization as a source of novel genetic markers for the sterile insect technique in *Bactrocera tryoni* (Diptera: Tephritidae). J Econ Entomol.

[13] Simpson JT, Wong K, Jackman SD, Schein JE, Jones SJM, Birol I (2009). ABySS: A parallel assembler for short read sequence data. Genome Res.

[14] Boetzer M, Henkel CV, Jansen HJ, Butler D, Pirovano W (2011). Scaffolding pre-assembled contigs using SSPACE. Bioinformatics.

[15] Parra G, Bradnam K, Korf I (2007). CEGMA: a pipeline to accurately annotate core genes in eukaryotic genornes. Bioinformatics.

[16] Nardi F, Carapelli A, Boore JL, Roderick GK, Dallai R, Frati F (2010). Domestication of olive fly through a multi-regional host shift to cultivated olives: Comparative dating using complete mitochondrial genomes. Mol Phylogenet Evol.

[17] Wang X, Chen W, Huang Y, Sun J, Men J, Liu H, Luo F, Guo L, Lv X, Deng C, Zhou C, Fan Y, Li X, Huang L, Hu Y, Liang C, Hu X, Xu J, Yu X (2011). The draft genome of the carcinogenic human liver fluke *Clonorchis sinensis*. Genome Biol.

[18] Marcais G, Kingsford C (2011). A fast, lock-free approach for efficient parallel counting of occurrences of k-mers. Bioinformatics.

[19] Rizk G, Lavenier D, Chikhi R (2013). DSK: k-mer counting with very low memory usage. Bioinformatics.

[20] Zhao JT, Frommer M, Sved JA, Zacharopoulou A (1998). Mitotic and polytene chromosome analyses in the Queensland fruit fly, *Bactrocera tryoni* (Diptera: Tephritidae). Genome.

[21] Peterson BK, Hare EE, Iyer VN, Storage S, Conner L, Papaj DR, Kurashima R, Jang E, Eisen MB (2009). Big genomes facilitate the comparative identification of regulatory elements. PloS One.

[22] Tsoumani KT, Mathiopoulos KD (2012). Genome size estimation with quantitative real-time PCR in two Tephritidae species: *Ceratitis capitata* and *Bactrocera oleae*. J Appl Entomol.

[23] Hu H, Bandyopadhyay PK, Olivera BM, Yandell M (2011). Characterization of the *Conus bullatus* genome and its venom-duct transcriptome. BMC Genomics.

[24] Smit AFA, Hubley R: **RepeatModeler Open-1.0.** 2008–2010. http://www.repeatmasker.org. Accessed 20 December 2014

[25] Green CL, Frommer M (2001). The genome of the Queensland fruit fly *Bactrocera tryoni* contains multiple representatives of the mariner family of transposable elements. Insect Mol Biol.

[26] Bennett CL, Frommer M (1997). The white gene of the tephritid fruit fly *Bactrocera tryoni* is characterized by a long untranslated 5’ leader and a 12 kb first intron. Insect Mol Biol.

[27] Palomeque T, Lorite P (2008). Satellite DNA in insects: a review. Heredity.

[28] Macas J, Neumann P, Novak P, Jiang JM (2010). Global sequence characterization of rice centromeric satellite based on oligomer frequency analysis in large-scale sequencing data. Bioinformatics.

[29] Jurka J, Kapitonov VV, Pavlicek A, Klonowski P, Kohany O, Walichiewicz J (2005). Repbase update, a database of eukaryotic repetitive elements. Cytogenet Genome Res.

[30] Smit AFA, Hubley R, Green P: **RepeatMasker Open-3.0.** 1996–2010. http://www.repeatmasker.org. Accessed 20 December 2014

[31] Stage DE, Eickbush TH (2007). Sequence variation within the rRNA gene loci of 12 Drosophila species. Genome Res.

[32] Cantarel BL, Korf I, Robb SMC, Parra G, Ross E, Moore B, Holt C, Alvarado AS, Yandell M (2008). MAKER: An easy-to-use annotation pipeline designed for emerging model organism genomes. Genome Res.

[33] Conesa A, Gotz S, Garcia-Gomez JM, Terol J, Talon M, Robles M (2005). Blast2GO: a universal tool for annotation, visualization and analysis in functional genomics research. Bioinformatics.

[34] Li L, Stoeckert CJ, Roos DS (2003). OrthoMCL: identification of ortholog groups for eukaryotic genomes. Genome Res.

[35] Dunn KA, Bielawski JP, Yang Z (2001). Substitution rates in Drosophila nuclear genes: Implications for translational selection. Genetics.

[36] **Fruit Fly (Diptera: Tephritidae) Classification & Diversity** [http://www.sel.barc.usda.gov/diptera/tephriti/TephClas.htm]

[37] Malacrida AR, Gomulski LM, Bonizzoni M, Bertin S, Gasperi G, Gugliclmino CR (2007). Globalization and fruitfly invasion and expansion: the medfly paradigm. Genetica.

[38] Smith PT, Mcpheron BA, Kambhampati S (2002). Phylogenetic analysis of mitochondrial DNA supports the monophyly of Dacini Fruit Flies (Diptera: Tephritidae). Ann Entomol Soc Am.

[39] Clarke AR, Armstrong KF, Carmichael AE, Milne JR, Raghu S, Roderick GK, Yeates DK (2005). Invasive phytophagous pests arising through a recent tropical evolutionary radiation: The Bactrocera dorsalis complex of fruit flies. Annu Rev Entomol.

[40] Shearman DCA (2002). The evolution of sex determination systems in dipteran insects other than Drosophila. Genetica.

[41] An X, Wilkes K, Bastian Y, Morrow JL, Frommer M, Raphael K (2002). The period gene in two species of tephritid fruit fly differentiated by mating behaviour. Insect Mol Biol.

[42] Orr HA (1987). Genetics of male and female sterility in hybrids of *Drosophila pseudoobscura* and *D. persimilis*. Genetics.

[43] Kulathinal RJ, Stevison LS, Noor MA (2009). The genomics of speciation in Drosophila: diversity, divergence and introgression estimated using low-coverage genome sequencing. PloS Genetics.

[44] Garrigan D, Kingan SB, Geneva AJ, Andolfatto P, Clark AG, Thornton KR, Presgraves DC (2012). Genome sequencing reveals complex speciation in the *Drosophila simulans* clade. Genome Res.

[45] Meats A, Maheswaran P, Frommer M, Sved J (2002). Towards a male-only release system for SIT with the Queensland fruit fly, *Bactrocera tryoni*, using a genetic sexing strain with a temperature-sensitive lethal mutation. Genetica.

[46] Via S (2009). Natural selection in action during speciation. Proc Natl Acad Sci U S A.

[47] Nadeau NJ, Jiggins CD (2010). A golden age for evolutionary genetics? Genomic studies of adaptation in natural populations. Trends Genet.

[48] Renaut S, Bernatchez L L (2011). Transcriptome-wide signature of hybrid breakdown associated with intrinsic reproductive isolation in lake whitefish species pairs (*Coregonus* spp. Salmonidae). Heredity.

[49] Emelianov I, Hernandes-Lopez A, Torrence M, Watts N (2011). Fusion-fission experiments in Aphidius: evolutionary split without isolation in response to environmental bimodality. Heredity.

[50] Nosil P, Schluter D (2011). The genes underlying the process of speciation. Trends Ecol Evol.

[51] Streisfeld MA, Young WN, Sobel JM (2013). Divergent selection drives genetic differentiation in an R2R3-MYB transcription factor that contributes to incipient speciation in *Mimulus aurantiacus*. PloS Genetics.

[52] Bolger AM, Lohse M, Usadel B (2014). Trimmomatic: a flexible trimmer for Illumina sequence data. Bioinformatics.

[53] Kelley DR, Schatz MC, Salzberg SL (2010). Quake: quality-aware detection and correction of sequencing errors. Genome Biol.

[54] Li H (2013). Aligning sequence reads, clone sequences and assembly contigs with BWA-MEM. eprint ArXiv.

[55] Quinlan AR, Hall IM (2010). BEDTools: a flexible suite of utilities for comparing genomic features. Bioinformatics.

[56] Edgar RC (2004). MUSCLE: multiple sequence alignment with high accuracy and high throughput. Nucleic Acids Res.

[57] Gouy M, Guindon S, Gascuel O (2010). SeaView Version 4: A multiplatform graphical user interface for sequence alignment and phylogenetic tree building. Mol Biol Evol.

[58] Li H, Handsaker B, Wysoker A, Fennell T, Ruan J, Homer N, Marth G, Abecasis G, Durbin R, Genome Project Data P (2009). The Sequence Alignment/Map format and SAMtools. Bioinformatics.

[59] Koboldt DC, Zhang QY, Larson DE, Shen D, McLellan MD, Lin L, Miller CA, Mardis ER, Ding L, Wilson RK (2012). VarScan 2: Somatic mutation and copy number alteration discovery in cancer by exome sequencing. Genome Res.

[60] Korf I (2004). Gene finding in novel genomes. BMC Bioinformatics.

[61] Stanke M, Tzvetkova A, Morgenstern B (2006). AUGUSTUS at EGASP: using EST, protein and genomic alignments for improved gene prediction in the human genome. Genome Biol.

[62] Lukashin AV, Borodovsky M (1998). GeneMark.hmm: new solutions for gene finding. Nucleic Acids Res.

[63] Grabherr MG, Haas BJ, Yassour M, Levin JZ, Thompson DA, Amit I, Adiconis X, Fan L, Raychowdhury R, Zeng QD, Chen ZH, Mauceli E, Hacohen N, Gnirke A, Rhind N, di Palma F, Birren BW, Nusbaum C, Lindblad-Toh K, Friedman N, Regev A (2011). Full-length transcriptome assembly from RNA-Seq data without a reference genome. Nat Biotechnol.

[64] Hunter S, Jones P, Mitchell A, Apweiler R, Attwood TK, Bateman A, Bernard T, Binns D, Bork P, Burge S, de Castro E, Coggill P, Corbett M, Das U, Daugherty L, Duquenne L, Finn RD, Fraser M, Gough J, Haft D, Hulo N, Kahn D, Kelly E, Letunic I, Lonsdale D, Lopez R, Madera M, Maslen J, McAnulla C, McDowall J (2012). InterPro in 2011: new developments in the family and domain prediction database. Nucleic Acids Res.

