# Supplementary material for: The draft genome of the pest tephritid fruit fly Bactrocera tryoni: resources for the genomic analysis of hybridising species
Source: BMC Genomics. 2014 Dec 20;15(1):1153. doi: 10.1186/1471-2164-15-1153 (PMC4367827; doi:10.1186/1471-2164-15-1153)
Supplement: Supplementary file 1 — Additional file 1: Summary of the data used for the B. tryoni genome assembly. (DOC 38 KB) [file 12864_2014_6888_MOESM1_ESM.doc]

Additional File 1. Summary of the data used for the *B. tryoni* genome assembly.

|  | Insert size (bp) | Read length (bp) | Reads per sample (million) | Total bases (Gbp) |
| --- | --- | --- | --- | --- |
| 3 x GAII p.e. | 300 | 100 | 60, 54, 54 | 16.8 |
| HiSeq p.e. | 500 | 100 | 298 | 29.8 |
| 2 x MiSeq p.e. | 738 | 234* | 24, 26 | 12.5 |
| 454 FLX Titanium | - | 517* | 1.41 | 0.73 |
| 2 x GAII m.p. | 3000 | 36 | 68, 44 | 4.0 |
| HiSeq LJD m.p. | 8000 | 88 | 87 | 7.7 |
| HiSeq m.p. | 10,000 | 36 | 28, 20 | 1.7 |

Table S1. p.e. indicates paired-end sequence data and m.p. indicates mate-pair sequence data. *Mean read length.
