# Supplementary material for: The draft genome of the pest tephritid fruit fly Bactrocera tryoni: resources for the genomic analysis of hybridising species
Source: BMC Genomics. 2014 Dec 20;15(1):1153. doi: 10.1186/1471-2164-15-1153 (PMC4367827; doi:10.1186/1471-2164-15-1153)
Supplement: Supplementary file 2 — Additional file 2: A fasta file of B. tryoni repetitive sequences. (DOC 543 KB) [file 12864_2014_6888_MOESM2_ESM.doc]

>Btry_Sat1#Satellite

AACGGCAGTGCATTTGGAAGAATTCGACAATCTTATATTCAGGTATAAAGTCGATTTTCTTGTATATACACATTTAGCAGTCGATATTGAGTTGCTTCAAATCATTAACTTCACATATTTAAGTTAATTAACCTTCTTCATCTTACTGAAGTTGACATAAAGCCAT

>Btry_Sat263#Satellite

GTTTTGAGAAAAATCACATGTTTGTTTCAACTTTGTAAACGCTCTAGCGGCTAAAGTATTCGACGTAGGTGGGTCAAATAAATAGCATCGGTTAGATAATTTAAGGAGCTTTTAGAAAAGCCCTCAACCTATTCTCTATCCCTACCAGGAGCGTGGCAATAGTGTTCACAAACAAATGTAGT

>Btry_Sat274#Satellite

ATATGAACTTTACATATTTATGCTAATTAGCCGTTTTGATGTTATTGGAGTTGTCATAGAGTTGTCCTATAGCAATAGCGACAGTGTATTTGGAACAATTTTACAATCTTAAATTAAGGTGTAGAATTGATTTTTTGGTACATACATATTATGCAATCGATATCAAGATGCGTAAAA

>Btry_Sat2169#Satellite

GTTCAGTTTGTATGGCAGCTATATGCTATAGTTAACCGATCTGAACAATTTCTTCGGAGATTACATTGTTGCTTTAGAAAATAATCTATACCAAATTTCGTGAATATATCTTGTCAAATGCAAAAGTTTTCCATACAAGAACTTGATTCCGATC

>Btry_Sat2877#Satellite

ACGTCACTGCATTTGGAAAAACTGCAGAATCTTATGTTTTGGTTCAAAATCGATTTTCTTGTTGTTTGTATATACACATTGAGCAATCGATATGAGAATAGCGTGAAAAAACTAACTTCACAAATTCAAGCTAATTTAACTTATTCACGTTATTAGAGTATTCATAAAGCAATG

>Btry_Hel1#RC/Helitron

AAAAACAAGTAAGGAAGGGCTAAGTTCGGGTGTCACCGAACATTTTATACTCTCGCATGATAAAGTGATAATCGAGATTTCATTATACGTCATTTACATATTTTTCAAATACCGTATTTTTGTAAAGTTTTATTCCGCTATCATCATTGGTTCCTAATGTACATATATATTATACAGAGAAGGCATCAGATGGAATTCAAAATAGCGTTATATTGGAAGAAGGCGTGGTTGTGAACCGATTTCACCCATATTTCGTACATGTCATCAGGGTGTTAAGAAAATATTATATACCGAATTTCATTGAAATCGGTCTAGTAGTTCCTGAGATATGGTTTTTGGTCCATAAGTGGGCGACGCCACGCCCATTTTCAATTTTTAAAAAAAGCCTGGGTGCAGCTTCCTTCTGCCATTTCTTCCGTAAAATTTAGTGTTTCTGACGTTTTTTGTTAGTCGGTTAACGCACTTTTAGTGATTTTCAACATAACCTTTGTATGGGAGGTGGGCGTGGTTATTATCCGATTTCTTCCATTTTTGAACTGTATATGGAAATGCCTGAAGAAAACGACTCTGTAGAGTTTGGTTGACATAGCTATAGTAGTTTCCGAGATATGTACAAAAAACTTAGTAGGGGGCGGGGCCACGCCCACTTTTCCAAAAAAATTACGTCCAAATATGCCCCTCCCTAATGCGATCCTTTGTGCCAAATTTCACTTTAATATCTTTATTTATGGCTTAGTTATGACACTTTATAGGTTTTCGGTTTCCGCCATTTTGTGGGCGTGGCAGTGGGCCGATTTTGCCCATCTTCGAACTTAACCTTCTTATGGAGCCAAGGAATACGTGTACCAAGTTTCATCATGATATCTCAATTTTTACTCAAGTTACAGCTTGCACGGACGGACGGACGGACAGACAGACATCCGGATTTCAACTCTACTCGTCACCCTGATCACTTTGGTATATATAACCCTATATCTGACTCTTTTAGTTTTAGGACTTACAAACAACCGTTATGTGAACAAAACTATAATACTCTCCTTAGCAACTTTGTTGCGAGAGTATAAAAA

>Btry3509#DNA/TcMar-Tc1

TTTTTCGAGCATTTCGGCCGGAATATTTTATCGACAAATTTTGTTCAGCGATGAGGCTCATTTCTGGTTGAATGGCTACGTAAATAAGCAAAATTGCCGCATTTGGGGTGAAGAGCAACCAGAAGCCGTTCAAGAACTGCCCATGCATCCCGAAAAATGCACTGTTTGGTGTGGTTTGTACGCTGGTGGAATCATTGGACCGTATTTTTTCAAAGATGCTGTTGGACGCAACGTTACGGTGAATGGCGATCGCTATCGTTCGATGCTAACAAACTTTTTGTTGCCAAAAATGGAAGAACTGAACTTGGTTGACATGTGGTTTCAACAAGATGGCGCTACATGCCACACAGCTCGCGATTCTATGGCCATTTTGAGGGAAAACTTCGGAGAACAATTCATCTCAAGAAATGGACCCGTAAGTTGGCCACCAAGATCATGCGATTTAACGCCTTTAGACTATTTTTTGTGGGGCTACGTCAAGTCTAAAGTCTACAGAAATAAGCCAGCAACTATTCCAGCTTTGGAAGACAACATTTCCGAAGAAATTCGGGCTATTCCGGCCGAAATGCTCGAAAA

>Btry13508#DNA/TcMar-Mariner

ATAATAGGTTGTCAAAAAAGTCTTGCGGTATTTTCGCTAGTTGGCGCTGAAAGCGCGTAGTTCTAGTTTTATTCGTCGCATCGGGTCATGCTATACCTTTTTGGAAAGCTCATTTCACGCGCTAACACGTGTTTGATTGATTGTCGTTTCTTTTAAGTCGTTCGTGAGTTATAGCGTCGCAAACATGGAGCAAAATAAAGAGAAAATACGGCATATTTTACAGTACTACTACGATAAAGGCAAAAATGCATCTCAAGCCGCCAATAAAATTTGTGCAGTTTATGGACCCGATACAGTTTCCATTTCCACCGCACAACGATGGTTTCAACGTTTTCGTTCTGGTGTAGAGGTGGTCGAAGATGCGCCACGCTCCGGAAGGCCTGTCGTCGAAAATTGCGATAAAATCGCTGAATTGGTCGAAAGAGACCGGCATAGTAGCAGCCGTAGCATCGGTCAAGAGCTGGGCATGAGTCATCAAAAATTCATTTCAATTTCAATAAAAAAATTCAATAAAAATACCGCAAGACTTTTTTGACAACCCAATTAT

>Btry_1315#DNA/TcMar-Mariner

ATACAAGGTGCGTTCCAAAGTAAACAGGACTTAAAAAAAAACAGAACAAATGGTTTTTTCGGCAAAATCAATTTATTTTATTCAAAATAGTCTCCTTCTGCTTCAATACAGCTTTTTGCACGGTCCAAAAGCATGTCGAACGAGTGTTTTAGCTCGTTGGCCGGTATGGCCGCCAGTATGCCGGTGCAAGCCTTTTGAATGGCCTCTACGTCTGCATAACGCTTTCCTTTCATGGGCAAATGCATTTTTCCGAAAAGGAAGAAGTCGCACGGTGCCATATCAGGTGAATACGGGGAGTGGTTAATGGTTAAAATGTGATTTTTGGTCAAATAATCGTCCTTCGAATGTTGGATTCTGAGCAATTTTTGGTCGTCAGTCAATTTGTGCGGAACAAACCGTGCACACACCTTTCGTAAGCCCAAATGTTCGGTCAAAATGCGATAAATCGATGTTTTGGAGATGTTCAATTCCATTTCCATGAATTTCAATGATGATTTCGGCTGATTTTTGATGAATTCACGCACAGTTTCGATGGAATTTCCGGTGATCACGGATTTTGATTGGCCCACATGTTGATCGTCATTTATGTCCTCACGACCACTTTGAAAACGTTGAAACCACTCGTGCACTCTGCTACGGGATAGGCAATCATCGCCATAAACTTGTTTCATCAATTGAAACGTTTCGGTAAAAGTTTTACCAATTTTAAAACAAAATTTAATGTTGGCTCTTTGTTCGAAGCTCATTTTCGCACCGATAACACAAACATACTGACACTTAAAACGCAATAACTTCACTTCCAATCAATGAAATGTCATGAAATTCTCACTGGACAATCGATAAAGATAGCAGATTCTAACGCACCAGTCGACATATAGATGGCGCCACCAGGGGGCGCTAGATTCAAAAAGTCCTGTTTACTTTGGAAAGCACCTTGTATA

>BtryLINE1#LINE/RTE-BovB

GGAGGATGGAGTCATGTGTAGAAGTTCACGCAAGTGAGGAAAGTTCTCTGATCGCCATTCACTTGGGAGTGGCCAGAAACGATTCTTTTACATATGGCTCAAGCAGCTCACGACTTCCGGTCTTTGACCAAGTATCCTCTGGGTAGCCTAAGAACATCCGTTCGAAGGCGAGCTAAAGTGAGAAGGCGAAACATCCCCTACATAGGGTTGTGCGCTGGGTTTGGGACCCGCCACGTAAAAAACACCCCCAATGAAAAGGAACAACCAGCCTCGGATGAGAGACCCCCCTTTTGATGACGACCATGGCAAACGAAATAAGGACTACGATTTGAGGGCATGCACCTGGAATGTCCGGTCCCTTAATTGGGAAGGTGCCGCTGCCCAGCTGGTTGATGTCCTCGTGAAAATAAAGGCTGACATCACCGCCGTCCAAGAAATGCGATGGACGGGACAAGGACAGAGACGAGTAGGTCCTTGTGACATTTACTACAGTGGCCATATAAAGGAGCGCAAGTTTGGTGTGGGATTCGTGGTGGGAGAGAGACTCCGTCGCCGAGTACTATCATTCACTCCGGTGAATGAACGTCTAGCCACAATCCGCATCAAAGCGAGGTTCTTCAACATATCGCTGATTTGCGCCCACGCCCCGACGGAAGAGAAGGACGATGTGACCAAAGATGCCTTCTATGAGCGCTTGGAGCGCACTTATGAGAGCTGCCCCCGCCACGATGTCAAAATCGTGCTTGGCGACTTTAACGCCAGGGTGGGCAAAGAAGGTATCTTTGGCACTACGGTCGGTAAATTCAGCCTCCACGACGAAACATCCCCAAATGGGTTGAGGCTGATCGACTTCGCCGGGGCCCGAAATATGGTTATCTGTAGTACTAGATTCCAGCATAAGAAGATTCATCAAGCTACCTGGCTGTCTCCGGATCGAAAAACTACCAACCAGATCGATCATGTTGTGATAGACGGAAGACACGTCTCCAGTGTTTTAGATGTGCGTGCGCTCCGAGGTCCTAACATCGACTCGGACCACTATCTTGTTGCAGCCAAGATTCGCACCCGCCTCTGTGCAGCAAAAAACGCACGCCAACAAACACAAGGAAGGTTCGACGTCGAGAAGCTGCAATCACAACAGACAGCCGAACGATTTTCTACTCGGCTTGCACTCCTGCTCTCTGAGAGCACTCGTCAACAACTCGGTATAAGGGAACTGTGGGACGGCATTTCAAACTCCTTACGTACAGCTGCAACCGAAACCATTGGTTTTCGGAAAGTGCAAAAGAACAGCTGGTACGACGAGGAGTGCCGTGTCGCAGCGGAGAGAAAACAGGCTGCCTACCTCGCAACGTTACGATCGACCACAACACGTGCGGGATGGGATAGATACCGAGAGTTGAAGAGGGAAGCGAGACGCATTTGCAGACAGAAAAAGAAAGAGGCCGAAATGCGTGAGTACGAAGAGCTTGATAAGCTGGCCGACAGGGGTAATGCTCGAAAATTCTACGAAAAAATGCGGCGGCTTACAGAAGGTTTCAAGACCGGAGCATACTCTTGTAGAACCCCCAAAGGTGATCTAGTCACCGATGCCCAGAGCATACTTAAATTATGGAGGGAACACTTCTCCAGCCTGCTGAATGGCAGTGAACGCACAACACCAGGAGAAGGAGAACCCGATTCCCCAATCGATGACGATGGAGCAGACGTTCCATTACCCGACCATGAAGAAGTTCGAATAGCAATTGCCCGCCTGAAGAACAACAAAGCGGCAGGGGCCGACGGATTGCCGGCCGAGCTATTCAAACACGGCGGCGAAGAACTGATAAGGAGCATGCATCAGCTTCTTTGTAAAATATGGTCGGACGAAAGCATGCCCAACGATTGGAATTTAAGTGTGCTATGCCCAATCCATAAAAAAGGAGACCCCACAATCTGCGCCAACTACCGTGGGATTAGCCTCCTCAACATCGCATATAAGGTTCTATCGAGCGTATTGTGTGAAAGATTAAAGCCCACCGTCAACAAACTGATTGGACCTTATCAGTGTGGCTTTAGACCTGGAAAATCAACAACCGACCAGATATTCACCATGCGCCAAATCTTGGAAAAGACCCGTGAAAGGAGAATCGACACACACCACCTCTTCGTCGATTTCAAAGCTGCTTTCGACAGCACGAAAAGGAGCTGCCTTTATGCCGCGATGTCTGAATTTGGTATCCCCGCAAAACTAATACGGCTGTGTAAACTGACGTTGAGCAACACCAAAAGCTCCGTCAGGATCGGGAAGGACCTCTCCGAGCCGTTCGATACCAAACGAGGTTTCAGACAAGGCGACTCCCTATCGTGCGACTTCTTCAACCTGCTTCTGGAGAAAATAGTTCGAGCTGCAGAACTAAATAGAGAAGGTACCATCTTCTATAAGAGTGTACAGCTGCTGGCGTATGCCGATGATATTGATATCATCGGCCTCAACACCCGCGCCGTTAGTTCTGCTTTCTCCAGACTGGACAAGGAAGCAAAGCAAATGGGTCTGGCAGTGAACGAGGGCAAGACGAAATATCTCCTGTCATCAAACAAACAGTCGTCGCACTCGCGACTTGGCTCTCACGTCACTGTTGACAGTCATAACTTTGAAGTTGTAGATAATTTCGTCTATCTTGGAACCAGCGTAAACACCACCAACAATGTCAGCCTGGAAATCCAACGCAGGATAACTCTTGCCAACAGGTGCTACTTCGGACTGAGTAGGCAATTGAGAAGTAAAGTCCTCTCTCGACGAACAAAAACCAAACTCTATAAGTCACTCATAATTCCCGTCCTGCTATATGGTGCAGAGGCTTGGACGATGACAACAACTGATGAGTCGACGTTACGAGTTTTCGAGAGAAAAGTTCTGCGAAAGATTTATGGTCCTTTGCGCGTTGGCCACGGCGAATATCGCATTCGATGGAACGATGAGCTGTACGAGATATACGACGACATTGACATAGTTCAGCGAATTAAAAGACAGCGGCTACGCTGGCTAGGTCATGTTGTCCGAATGGACGAAAACACTCCAGCTCTGAAAGTATTCGACGCAGTACCCGCCGGGGGAAGCAGAGGAAGAGGAAGACCTCCACTCCGTTGGAAGGACCAAGTGGAGAAGGACCTGGCTTCGCTTGGAATATCCAATTGGCGCCACGTAGCGAAAAGAAGAAACGACTGGCGCGCTGTTGTTAACTCGGCTATAATCGCGTAAGCGGTGTCTACGCCAATTAAGAAGAAGAAGAA

>Btry37622#DNA/TcMar-Mariner

AAAAATAGTAAGACTTTGTTCATAATTTTAAAATTCTTAATTTATTCTTCAAAATCTATGTCGTTCACACTCAAAGTAATCCCCCTTGGCCCCAATACACTTGTGCCAACGTTTTTTCCAATCCTCGAAACAGTTGTTAAAGTCAATTTCCGGAATAGCCTTCAATGCGCGTAGCGATTCACGTTTAATGTCTTCAATTGACTCAAAACGGTTTCCCCGGAGCGGTCGTTTGAGTTTGCTGAATAGCCAGAAGTCACACGGAGCTAAATCAGGCGAATACGGTGGTTGCGGCACGATATTGGTTGAAAATTTGGCGAAAAACTCACGAAGAATCAATGCAGTATGCGACGGTGCATTATCGTGGTGCAAAAACCAAGAGTTGTCGGCCCATAATTCCGGCCTCTTTTTACGAATAGCTTCGCGCAAACGACGCATAACACTCAAATAGTATTCCTTGTTGACAGTTTGGCCGGTCGGAAGGAATTCGGAGTGCACCACACCTCGATAATCGAAGAAAACTGTCAACATAACCTTGATTTTTGACCTGCTTTGACGGTTTTTCGGCTTCGGCTCACCTTTGCCACGATATTCGGCCGATTGATCGTCTGTTTCCGGGTCGTAAGCATAGATCCAAGACTCATCGCCAGTAATAATACGTTTCATGACATCCTGGTAGTCGGAAAGCATTGTTTCACAGACGTTAACGCGACGCTGTTTTTCGAAAAAATTTAGTGATTTTGGAACCAATCGTGCTTTCACTTTTCTTAGGCCCAAATGATCTTTCAAAATGGTTTTCACTGATCCTTCCGATATTCCAACGATGCCAGTAAGATCTCTGACTGTTAATCGTCGATTCTCAAGCACCAATTCCTTTATTTTATTGACGTGTTGATCATCAGTTGATGTTGATGGCCGTCCTGGACGTGGTTCGTCGTCAACGCGTTCTCGACCCTCTTTGAATAATTTGTACCAATCAAAAACACTTGCTCGCGACAAACAATTATCACCGAAGGCCTTTTCCAACATTCTGAACGTTTCGGCACCAGAAATTTGATTCCGCACACAAAATTTAATGGAACTTCTTTGTTGAATAATTTCACTCATCGTAAAAATCGCCGAATGCACTTTATGTACTTCAGAAAGACAAGCGTATACTAAACACTAATGATTATTTTGATGTGACATTTGGCACAGATGTCACTGACAGTCATACCAACCTAGAAAAAAAATATTTCGACGAATGGGTTTTCGCGCGAAATTTAAATTAAAAAGTCTTACTATTTTTTGCCCACAGTAGTATAT

>Btry_Hel3#RC/Helitron

AAACAAGTAAGGAAGGGCTAAGTTCGGGTGCAACCGAACATTTTATACTCTTGCAACTTGCAAGAATCAAAGCCAGGGAAATACTTTAAGGTGTAAAACGTCAACCAGAGGATCGAAATCCAAGCAATTTTATATATACATATACTATATATAACTCATACACTGACCGACATATTCGGCATAAGATTTGTTAGAAAAACGAAAATCATTATATGTAGTATATGGGAGTTGGGGTAGTATCGACCCGATTTTATCTATTAACTATTATCATGCCACATACTAAAAAAATGTTCCCTGGGTTTCATTAAGGTACCTCACATACAATCACCAATCATATGGAGCAAAGTCAGCCGGATGTTCGAAAATCCTGATATTAGTTATATGGGGGCTAGGTCAAGTTTTCGCCCAATTTTATCTATTTTAGGCACAAAGATACACTGTTATGAGTAAAACACGTTCTGTAATTTTCATTGAGATAACTCAAATATTGGCCGATATATGCAGTATAAAGTCACCTGGAAGTTCGAAAATCTTTATATTAGGTATATGGGGGCTCAGGGAAGTATTGACCCGATTCAACCCATTTTTGATACACAGAAATACTATTGTCAGGAAAGGATTCTCTCTGAATTTCAATTGTATATCTCACACATTGACCGATATTTTCGGTCAAAAGTCAACTATAGATACTGGGGTCCACATATTCGGTACCTAGGGGCTTGAACAGTTTTGGTTGGATTTGGACAATTTTTGGTCATAAGGTGGCATACTTTAAAGGAATTATTAGTGCAAAATTTTATCCCGTTATATTAATTACTTCTTGATTTGTGTACTGGAAAGTGAAAGAATCAAATGGAATTTAAAATTGTGTTATATGGGAAGTAGGCGTGGTTGTAGTCCGATTTCGCCCATTTTCACACTGTGACATAGGAATATGAGAAGAATGCCACGTACTAAATTTTGTCGAAATCGGTTGGTCGGGTCCCGAGATATGGGATTTCACCAAAAAGTGGGCGGTGCCACGCCCATCGTCCAATTTTCACACCGGCTCCCATAAAGCTCTCTCATACCATCTCGGTGGTAAAATTTAATGTCTCTGGCGTATTTAGTTATTGATTTATCGCGCTTTTAGTAGTTTTTAACAGTACCGTTATATGGGGAGTGAGCGGGGTTATCATCCGATTTCATCCATTTTCACACTGTCGGTAGAAGTTCTTATAAGATTTGTACTCAGCAAATTTGGTTGTTGTAGCTTAAGTGGTTTAGGAGATATGTACATTAAACTTATTAGAGGGCGGGGCCACGCCCACTTTTTCAAAAATTTTTTCCCACAGGTGCCCCTTGCTACTGCGATCCCCTGTACCAAATTACAGTTTTATATCTTAATTTAGTGCTTAGTTATGGCACTTTATATGTTTTCGGTTAATGGCGATTTGTGGGCGTGGCAGTGGTCCGATTACGCCCATCTACGAACTCGAACTTTTTTTTGTACTAAGGAACCTGCATACCAAGTTTCATCAAGATATCTCAATTTTTACTCAAGTTACAGCTTGCACGGACGGACGGACAGACAGACAGTCAACCGGATTTCAACTTTTCTCGTCATCCTGATCATTTATATATATATATATAACCCTATATCTATCTCGATTAGTTTTAGGTGATACGTACAACCGTTAGGTGAACAAAACTATTATACTCTGTAGCAACATGTTGCAAGAGTATAAAAA

>Btry_39337#DNA/TcMar-Tc1

ACTAACGGGTGATCCAAGTAGAGGTACTTTTTTCAATAGCCTTTTTTTGACAGATCACGCGTGAGTCGTGTCAAGCTGTCATGTTATTTTTGTTCAGTATTGTTTGGCATTTCATCATGGAAAGACTTACGCCTGAACAACGTTTACAAATCGTTCAACTTTATTACGAAAATTCACGTTCTGTAAAGAATGTGTTTCGCGCGCTTCGCTCAACTTATGGTCAACATAATCGGCCTACTGAGCGTACTATTCGCAACACCATCACCCATCTTGAGACCCAGCATTCATTATTGGATAATATTCGACCGAATAGACCACGTCCAGCACGCAGTGAAGAAAATATAGCAGCCGTAGCTGAGAGTGTACACGAAGACCGTGGAGAGTCGATTCGGCGCCGTTCGCAGCAACTCGGACTGACGTATGGAACGACTTGGCGCATTTTACGTCGAGATCTTAAATTGAAAGCGTACAAAATACAGCTTGTGCAAGAACTGAAGCCGCTCGACCTTCCCAAGCGACATCGCTTCGCTCTATGGGCTCTTGAAAAGTTCCAAGAAGATCCGACGTTTTCGAGCCAAATTTTGTTCAGCGATGAGGCCCATTTCTGGCTCAATGGGTATGTAAACAAGCAAAATTGCCGCATTTGGGACGAAGAGCAACCTGAAGAGATTCAAGAGCTGCCATTTCATCCAGAAAAAACAACGGTTTGGTGTGGTTTGTGGGCCGGTGGAATCATCGGTCCATATTTCTTCAAAAATGATGCCGGTGAGAACGTAACCGTCAATGGCGACCGTTATCGCGCCATGATAACCGACTATTTGATGCCTGAAATTGAAGCTCGTGATCTCGGCGACATTTGGTTTCAACAAGACGGCGCCACTTCCCACACATCGCATCAATCAATGGATTTATTGAGAGAACACTTCGGTGAGCAGATAATTTCACGTTTTGGGCCGGTCGATTGGCCACCAAGATCGTGTGATATCACACCGTTAGACTTTTTCCTGTGGGGATATGTAAAGTCTAAAGTCTATGCGGACAATCCCGCTTCGATTCAGGCCTTGGAGCAAAACATCACGCGTGTCATTCGCCAGTTACCAGTCGAAATGCTCGAACGAGTCATCGAAAATTGGACTCAACGGATGGACCATCTGAGACGTAGCCGCGGCCAACATTTGAAAGAGATAATCTTCAAAAAATAAATGCCAAAGAATGTTCTTTCGAATGATAATAAACATTCCCCATTAAATTTGAAGTTTCTGTGTTTTTTCTTTAAAAAAGTAGGGAACCTCGAAATGGATCACCCTTTATA

>Btry_Mar43870#DNA/TcMar-Mariner

ATATATTGGGTAGTCGAAAAAGTCTTTTCGTATTTCTAATCAAACTTCAACTTATTTTTTTTATATTTATAATGAACTTTAATGAACCAAATATGTACCATTTTGGTCGACCACTTTTTGCCATTTTTCCGCTAGAGACATTATTCCATCAGTGTAAAACTTTTCTGGTTTCTCGGCGAAAAACTGCGACAAGTAATTTTCACAGGCTTCTCTTGAAGCCAACTTTACTCCATTAAGGGAGTTCTGCATTGACCGAAACAAATGGTAGTCCGATGGTGCAAGGTCAGGGCTATATGGTGGATGCATCAAAACTTCCCAGCCAAGCTCTCCCAGTTTTTGCCGAGTCATCAAAGATGTGTGTGGTCTAGCGTTGTCCTGATGGAAGACGAAGCCCTTTCTGTTGATCAGTTCTGGCCGTTTTTTTCGATTGCTTGCTTCAATCTCATCAGTTGTTGACAGTAAAATGTAGAATCAATCGTTCGACCAGGCTGGAGCAGCTCATAGTGGATGATTCCTTTCCAATCCCACCAAACACTCAGCATAACCTTTCGAGGCGTCAATCCTGGCTTTGCGACCATTTGTTGAGCTTCACCACGCTTGGACCATGATCTTTTTCGCACATTATTGTCGTATTTGATCCACTTTTCGTCTCCTGTTACCATTCGCTTCAGAAATGGTTCGATTTCATTTCGTTTCAGCAAAGAATCGCAGATGTTAATTCGGTCCATTAAATTTTTCACAGACAATTCATGTGGTACCCAAACATCGAGCTTCTTTTTGTAGCCAGCCTTTTTTAAATGGTTCAAAACCGTTTGATGATGAATGTTAAGTTCCTTAGCGATGTCATGGCTGCTTATGTGACGGTCCTGGTCAATCTTTTCCATAATTTCATCGACTTTTTCAACGATAGGTCGACCAGAGCGAGGTGCATCTTTCACATCGAAATTTCCAGAACGGAAGCGAGCGAACCATTGTTGTGCTACACGAACTGATACAGCATCGTCTCCGTAAACTTCACAAATTTCATTGGTGGCTTGCGTGGCATTCTTCCCTTTTTTATACAAAAATTTCAAAATATAGCGAATTTCTTCATTATTTTCACTCATTTTTGAACAGCTGTAACTTTTTTTCAACTTCCCCGAATTTAATTTTTTTTTGGTTAAATGAAGCTTAAAATCTCACCTTTCCAACACTATATGGTATGACACAATGTGATTGGTAGCACTGGAGATATACGACTGCAACGACATCTATTGACAAAATACGAAAAGACTTTTTCGACTACCCAATAT

>Btry56645_Hel5#RC/Helitron

ATACTAGCTGACCCCGCAGCCGTTGTCCTGCGTGAAATTATGTGTTTTGAAATGAAAAGAAAGTTGAATTTATCATTTCATTTTTTTTTCAATGTAACGCAGCTTGATAAACAACATTTTTTGGTTTCTGTTCCGGTGTACAGAAAAGATGGTTTTCCAACTCGTGAGCACGCCACGTATATCTGGCCGTGTGAAAAACATAGCATTTCCAAATTTATGCCACACACTATGCGACTATCCTTGTGTCCTGTTGATTGTCATCTCAAATGCAATTTTCACTGGAAACGGAATACGTTTGAACTGAAATGGCAAATCGTTAGAACTCAAAGGAATTCGTAGAATCAAGCATTCTGCCCCCTTGTATTCGATAGCTGCGTCACAATCAGTCGGGTTCCATTACACAGTTTCGGCGCATGAAGATTGCGAAACATAATAACGACAGATCCAACTTTGAGACGCAAATGATGCGGTGGCATACCAAGCCTCTCCAAAGAATTTAGAAATTCCACTGGATAATTCACGGCGTCGTCTTCATTGTCAAGACGATCGATCGATTTGTATGAGCGCAAGTCTCCCGGAATTTGACTTTGAATCTTCCAGTTTAAGTCAACAACATCGGTATTTTTGGCAGCTAACATTGCGCGTGCACTTAAGGAATCGTAGTTACGATAATTTGGCCAATGTCCGAACATTCGTGATGAGTTCATCTTTCGTGAATTGATAAAGGGAAGATGGAATTGATATCAAACCACCGGAAGTATCAACCGGAATTGACCCATTTCCAATTCTTAGCGAATACGATGTAAAATGTCTTCGGCAATGTCATTTTTGTTCGTATCCCATAATTGACGCGGATTTGAAGGCTGATATGTCGAAATGATTATCGCGAAAAGTGTGCGAACTTCATTTTCATTCCAGTGATTAACATGTTCCAGCAATTGTAATTGCTGGCTTACTTCTCCAAAAGTTGCACACACCGTACCATTCACAGTACGCAATGACTCAAATGAAGTTGAACCGCGTACATTAATCAATAGCAACCGAAGGTAGAAGCAATCGTCGTTTTTCGGGTGGATTGTGTAAATTCGTCCTAATGCATTAGTTGAGAACACACCTGGATGTCCATCTACTGGTTGGCCTTGCTTTCTGCGTAACTATTTCTTCGACGATGCATTCCATGTATAATATCAAGGTATTTCCGAATAGAGCAATGTTCTCGCAAACGGATCACTGACGAAAGTTGAGAAAAAACTCGTCAATGTTAATTTTGTTGCTGGCGGTGTTTCCACTGGCTGTACTGTATTCTCTGGGTTGAAATACACTCTTTGGCCATTCTCCAAATTAACTGCGAGACGCACAACAGTCGGAAAACGTTCATGAATTGCAAAAGTAAATATCCTACAAAGCGCTTTATTGCAGTTCACGTATCGACCGTATCGTTCAAGACCAATAACCGCCATGTTGCTTCCTTTGGTGACGTACTTACAAACGTATTTTATCGATTTCACCAAACTGCAATATTCAACATTGACATGAGTTTTGAATGTGAATTAGACAATAGTGGCGAATATGGTACGATCCATGTGTTGTCAACTTCGATATTCACTCTTCTAAATTGAATGCTGAATGTTCTGCCATTGTCGTCTGGTGAGCGACGCCGATAGAGTGGATATCCATCATTTCTAGTTTGTGTTTCCGAAAGAAAAGCACGTGGATAGTGTTTCGTGCATTTATTGTCAGACATACAAACCGAAGTGGGATTGTGGTGTCCGCAAGGTCCATGAACCATATTGGTTTTCATCACTTCGTATAATACTGGATCTTTCTCTGCATCAGGAATTTCCGCAGAAATGATTTCATCAATTTGATCTGGTGTAACCACCATCCAAAGAAGAATGTGTGCGTGCGGCAAACCTCTCTTTTGCCACTCAACAGAGTACATCCAGCATCTAACAGCACCACATATACGTTGCTTCAAGATAAAGTCCATCAAACATCGTAACTGTTGTTTGAAAAGTCGTGCTGTGACATCGTGACGATCGCTTGCTGATTGACCGCGATCCATAATTCCGCACGTATGTCATCGCATCCTGGGAGTACTCGTTCATGTGCCTTGGGCTGCCATACGTTGATGGCAAAAAGAACGCCGACCGATATTAGCGCGACCTCTTCGTGGCTTCGTAACAAATTTCAATCTGTAATTGATCACTTTGTGTGAAACACACATAAAGTTTTCACTGAATAATTTTCAATAATTTTTCTTTTGAATAGCCGGAATAAAAAAGCAAGCGACCGAAATTGAACGATTCAAATAATTTACAAATCAAAATATTGAAAAACAAAACAAACAAAAATTGAAAAAAAAATTGTATGGAAAAAAGTTACTTTTTGGAATTGTCCAATTTTCCGACTTTTCCTCATGAAAAGTATAAGGTATTTTTATTTCTAAACCTTCCCCGATCCACAACGAACAACCTCTGAAAATTTCATCAAGATTGGTTCAGCCGTTCTCTTAGCGTTACTAACAAACAAACATTCATTCGTTTGTATGGGAAAAAGAAAAGGGCTGTTTTTAGGGGTTTTCCGGCAATTATTCGAATTTTTCTCGCCGTAAAAACCATCCTTGAACTTCAACGAACATTTAAGAAAAAGAATTGGCCAAATTGGTCCAGGCGTTGTTGAGTTATGCGCTTAGCAACACATTTTGCGATTCATTTTTATATAT

>Btry_Mar71742#DNA/TcMar-Mariner

TACATTGTGACAAAAAAGCACCCGGAAATTGTAATTAAATTCCCCGGGTAAATGATATTTCAAAAAAATGTATTTTGCTAAGTTGGTAGGACTGTCCTTAATTACTATGCCAAATATGAGCGCGATCTGTCAACCAGTTTGTTTACAGCAGCTGCTTAAGTCGGTACACCTCAGTAGTGCGTTGCGATTTTTACAATGGATAAAAATATCGAACAAAGAATTTGTCTCAAATTTTGTATTTCTAACCAAATTTCGTGTGCGGAATCGTTGCGAATGTTGGAAAAGGCTTACGGTGATTCAGTTTTATCAAAAACACAAGCCTACGAGTGGTACAAAGCCTTCAAAGACGGTCGAGAGATCGTTGAAGACATGCCTCGTTCTGGACGACCTTCGACCTCTTCAACTGATGAAAATATTAAAAAAGTGAAGGATATGGTGCTTGAAAAAAGTGTTAGAGAGATGGCAAGAGAGCTCGACATCTCTCGCGAGTCCGTTCGAATGATTTTGGTGGATATTTTGGGTATGAAACGCGTTCTTGCTCGACTCGTCCCGATAAAGCTGAATTTTTTTCAAAAAGAGTACCGTAAACAGGTCTCTTTGGACATGCTTGATCGTGCGAATTCCGATCCCACATTCATGGAGAGCATTATAACTGCCGATGAGACATGGGTTTATGAGTTTGACATGCAAACAAGTCAACAATCATCGGAATGGAGAAAAAACGAGCCGAAACCAAAAAACCACGCCAAAGCCGCTCAAAAATCAAGGTGATGCTCATTGTTTTTTTCGATATTCGTGGTTTGGTGCATCATGAATTTGTTCCGGAGGGACAGACGGTCAATAAGGAGTTCTATTTGGCCGTATTGAGGCGTTTGCGTGAGAACATCCGTCGAAAACGGCCGGAATTGTGGAAGAACAATTCATGGATTTTACACGATGATAATGCACCATCGCATCGAGCCACGATTGTGACCGAATTTAAAGCCAAAAACGCAATGAATACCATCGATCAACCACCGTATTCACCAGATTTGGCTCCGTGTGATTTTTTCTTGTTCCCCAAACTGAAATTGCCGCTCCGTGGAACCCGTTTTCAGTCGATCGAAGAGATAAAACAAAATTCGCTGAAGGAGCTGAAGGCCATCCCAAAAAGTGCTTATGAAAAGTGTTTCGAGGACTGGAAAAATCGTTGGCATAAGTGTATTACATCTGGTGGGGATTACTTTGAAGGCGACAAAATAAATATTGATGAATAATTAAATATTTTGCGTTTTATTTACAATTTCCGGGTA

>Btry_Mar81574#DNA/TcMar-Tc1

GGGGTTTTCAATAGGGACGCTACAAAAGTAGACCGATAGGGACAGCAAACGACGCCATATTTTTTCCCGCTCTTTTGACATTTCTCTTCAGTAAGGTTTGCCATTTCATCATGGAAAGATATACGATCCAACAACGAGTCGAAATTATTAAAATTTACTACCGAAATTCGGAGTCAGTGGCCTCAACTTTAAGAGCGCTACGTCCAATTTATGGTCGTCATAATCGTCCTGTCAGATCAACAATTGAGCGTCTAGTGGAAAAATTTGAATCCACAGGCACAGTACAAAATGTTCCCGTGCCAGTGAGACAAAGAAGTGCCCGTAGTGTCGAGAATATTGCTGCCGCTAGCGCATCAATTGAGGAAGACCCAAATCAGTCTCTCACACGTCGTTCTCAAGCGTTGGGCATCTCTGTGACGTCGTTGTGGCGAATTTTGCGAAAAGATCTTGGCCTACATCCTTACAAGATCAAATTGACGCAAGAACTGAAGCCGCTTGACCACCAGAATCGTCGTATGTTCGTGAATTGGGCTGAGCAACAACTTGAAAATGATCCGGATTTTCATCGAAAAATCATCTTCAGCGATGAGGCTCATTTCTGGCTGAATGGCTTCGTCAATAAGCAAAATATGCGTTATTGGTCAGGCAGCAATCCACACGTACTCCATGAGTCACCATTGCATCCCGAAAAAATTACGGTTTGGTGCGGTTTATGGGCCGGCGGCGTCATTGGGCCGTACTTCTTCCGTGATGATCAAGACCGGCACGTTACTGTGAATGGGAATCGCTACCGCTCAATGATAACCGAATATTTTTGGCCCGAATTGGATGATATGGACTTGGACAATATGTGGTTCCAACAGGACGGCGCCACAAGCCACACAGCGAATGTCACAATCGATTTATTGAAAACCAAGTTTGGTGAACGTGTTATCTCACGAAATGGCCCAGTCAATTGGCCGCCTCGGTCGTGCGATTTGACGCCGTTAGACTATTTCCTGGGGCTACGTCAAGTCTATGGTCTATGCCAACAAGCCAGCGACGATTGATGAACTTCGTACGAATATCGAACGTGAAATTGCAGCAGTATCGGCCGATTTATGCTTGAAAACCGTCGAAAATTGGGTTCAGCGTCTGGACTTCTGCAAGCGT

>Btry_MarA#DNA/TcMar_Tc1

TACAGGGTCCGGCACTCGAAGTGTAACCAACTTCAGACCGTTCGCGCAGCTGTCGCACTGGAATCAGCTGTTTATAAATTTGCTGAATGACAGTTCGGAGTATTGTTCACAAGTGTACAAAAGCGTTTTGCCAAAAGTGAACGCAAAAAAAGTGATCAGCGATGGAATTCAAACGTAATAGTGTGATTGCTTTATATTTAGCTGGAAAATCACAGCCAGCAATTGTTCGTGAGCTCAAACACCTTAATGTGAATAAAGTTTTTGTTTATCGCACCATCACTCGTTACAATGATACTGGTAGCATCGCAAAACGCCATGGAGGTGGTCATCAAAAGACTGCAACGTCACGTGAGATGGTTCGGAAAGTGAAGAAGCGACTTGAGCGAAATCCACGACGAAGTGCCAATCAAATGGCTAAAGAACTGAAAATATCCGACCGCAGCATCCGACGCATATTGAAAAATGAGCTCAAGGTCAAGCCTTACAAGTTCCAAAAGGCCCATGATCTCACACCGAAGCAGCAACAAGTAAGACTTGAGAGAGCGAAGCAGTTGCTTCGCTTGGCCGAAAGCGGTCAAATTCCGAACATTGTGTTTTCTGACGAAAAAATTTTTCCAATTGAGCAATTCGTAAACTCTCAAAACGATAGGGTTTACTTGACCGACCGTTCATACGAGAATCTAAGTCATCGGTTGGCCACCAGGAGGCAGCACCCGCCACAAATAATGGTTTGGGCCGCTGTCACCGCAGATGGGCGCTCTCCAATTGTTTTCATCGAGCCTGGCGTCAAAGTAAATGCGACATATTATCGGGAAAGTGTTCTGGAGGCTGCTTTGAAGCCGTGGGCAAACAAACATTTCGGTCGCAAACCATGGACGTTTCAACAAGACTCAGCACCGTCTCACAAAGCGCGAGTGAACCAAGAATGGCTGAAAAACAACGTTCCGAACTTCATTACGACCACACAATGGCTCTCGAATTCACCAGATGCGAATCCAATGGATTATTCTCTCTGGGCCATTTTGGAGAGCAAGGTCCGAAGTAAAAAATACACCAGTCTCGAGATGCTGAAGAAAGCCATTGTCCGTGAGTGGGCCAAAATACCGGCAAGTCACATTCGGGCAGCTTGCGATTCGTTTTTTGACCGTCTCAAGGCCATAGTTAAGGCAAAAGGTGGTCATATCGAGCAAAAGTGAATTGGTCCTGAATTTATGATTATTTTCACACATTTTGTACTTTAAAATAAATAAAAATAATTTTCCAAACCGAATTTATGGCTTTTTTAATTGGTTACACTTCGAGTGCCGGACCCTGTATA

>Btry_MarD#DNA/TcMAr

ATACCAGGTTGTTCAATAAGTTTTGTCGTTTGATAAGAAAAACACAATTTTATGATTCAAAATACACTTTATTATTCAGTATAATCTCCCTGAACATTAATACACTTGCTCCAACGATCCTCCAATCTGTGGATCCCATCCCTGAAGTGAGAATCCGGAAGGTCTGCAAAATACGCTTCAACAGCCGTTATGACCTCTTCATTTGATGAAAAACGCTTGCCACGCATAAATTTTTTGAGTTCTGGAAACAAATGGAAGTCGCTGGGGGCCAAATCTGGTGAATACGGTGGATGCTCCAACAATTCGAACTTTAATTCATGGATTTTAGCCATTGTCAAAATGCTCTTGTGACACGGTGCATTGTCCTGATGAAAAAGGATTTTTTTCTTCTGCAAACCGGGTCTTTTTTCACGAATTTTTTCCTTCAACTGGTCCAAAAGGTTGCAATAATATTCAGAATTAATTGTTTTACCAGTTTGCAAGTAATCCACAAACAAAATTCCTCTCGCATCCCAAAAAACTGATGCCATAACCTTCTTGGCCGATTTCCGGACACGAACTCGTTTCGAAGCCGAACAACCAGGTTCACACCACTCTTTAGCCTCTTGTTTTGATTCAGGATCATGGTGATAGACCCAAGTCTCATCCATAGTGATGAATCGACGCACAAAATCCACTTTATCCTTTTTAAAACGCTCCAAATGTTGCTGAGAAAGTCGCATTCGAATGTGTTTTTGTTCCATTGTTAGCGAATGCGGCACCCATTGTGCACACAGCTTTCTAAAACCCAAAATTTCACTTAAAATATGGCTCACACTGCCTAATGAGATGTGTAGAGCCTCTACTAAATCTCTCTCAGTCAATCGACGATCTTCCAAAACCATATCCTGTATTTTGGCTATGATTTCTGGTGTTGATGCGCTTTTTGGACGTCCTTCACGTGGATCGTCTTCAAGGCTTGTACGACCACGTTTAAATTCAGCAACCCATCTTTCTACTGTTCTAATTGTAGGTGAACAATCATTATACACTTTTAACATTCGTTCGTGAATTTCTTTTGGTGCTACACCTTCCAAAAATAAGAATTTTATCACTGCACGATATTCAATTTTTTCCATTGTAAAAAAATACTGTGACACGGCGACACTAAATGGCTTGTAAACAAAGAATGAATTGACAGATTGAAATGAAACTTCACATACGTTCATATGAAGAGTGTACCAACAAAACAAAAAAAATNTTGGGCTAGTAGCAACGCCCTCTCTTATCGAACGACAAAACTTATTGAACAACCTGGTA

>Btry_MarG#DNA/TcMar

ATATTAGGTTGTCAAATATCTCCCTTCCGCCTTTTTGTCTTTTGAATTTCGCGGCTATGTATAAAGCGCTACAGAGCTCGTATCTGGCAATACTATACATCTTTGAAAGGTCTTGACATAACCTACAAAACGACGCTATGCATGATTAGTTTGGATATTGCGTTCAACAGTTATAGACGTGTAAACATGGAGTTCACTAACGCCGAAATTCGCGCTATTTTAAAGTTTTCCTTCGTTAAAGGCAAATCCGCTAGAGAAACGTTCCGTGAGATTAATGGTGTTTTGGGGGATGGTACTCTATCACTTCGAACTGCGGAGGAATGGTTTCGACGATTCAGAGCGGGTGAAAACGACACCATGGATAAGCCAGCCGGCGGAAGACCTGTGACGACGAATACCGATCAAATCATGGAAAACATCGAGTTAGACCGGCATGTGGCATCTCGTGACATCGCCCAGAAGATGGGAGTTAGTCACCAAACCATTTTAAACCATCTGCAGAAGGCTGGATACACAAAAAAGCTTGATGTTTGGGTGCCGCATGATTTGACGCAAAAAAACCTTCTGGACCGAATCAACGCCTGCGATATGCTGCTGAAACGGAACGAACTCGACCCATTTTTGAAGCGGATGGTGACTGGCGACGAAAAATGGATCACATACGACAATATCAAGCGAAAACGGTCGTGGTCGAAGGCCGGTGAATCGTCCCAAACAGTGGCCAAGCCGGGATTGACGGCCAGGAAGGTTTTGCTGTGTGTTTGGTGGGATTGGAAGGGAATCATCCACTATGAGCTGCTCCCATATGGCCAGACGCTTAATTCTACCATCTACTGCGAACAACTGGACCGCTTGAAGCAGGCGATCGACCAGAAGCGTCCAGAATTGGCCAACAGGAAGGGTGTAGTGTTCCACCAGGACAACGCCAGACCACACACTTCGTTGATGACTCGTCAGAAGCTACGGGAGCTCGGATGGGAGGTTTTATCGCATCCACCATATAGCCCGGACATAGCGCCAAGTGATTACCACCTGTTCCTGTCCATGGCGAACGCCCTTGTTGGTGTAAAGTTGAACTCAAAAGAGGCTTGTGAAAAGTGGCTGTCCGAGTTCTTCGCAAATAAGGAGGGGGGCTTCTACGAGGGGGGTATTATGAAGTTGCCGTCTAGATGGAAACAGATTATCGAACGAAACGGCGCATATTTGAACTAAATCCGATCACTGTAACACTTTTTATAAAGCATTGAATAAAGAGCAAAAAAGCGGAAGGGAGATATTTGACAACCTTATATAT

>Btry_958_partial#DNA/TcMar

TTATATATATACTAGAGGACCCGTCCACGCTTCACTGTGGCTGATACATAGATAATACTACTACTACCATCTATATGATTTCTATTAGTTGGATTATAACTATTAGTTAATGAGATATAGCATTTTTGTGAAGCAACTTGTGTTAGTTTACAAAAAAATGGATCATAAAGCATTTCGTGTGTTAATAAAGCATTGCTTTTTGGGGGAAAATACTGTTGAATTTGGGCTCAGGGAAATCCACCGTTGAAAAGATATTTGCTAAGCTGGAAACAGGCGAAATGAGCACCGAGGACAATGATGCTCTAAAGAGGCGAAAGCTCTGTGCAAAGTGGGTGCCTCGCAAGTTCATAATCCAACAAAAACAACGAATAACTGATTCTGAGAAGTGTTTGAGTGCTCTTTAATCGTAATAAAACCGAATTTTTGCGTCGGTGTGTGACAATAGAAACATAGCTCCATCATTTCCCACTGGAGTCCAATCGACAGTCATTCTAGTGGACTGCACATGATGAACCGGTTCTCAGGCGTGGAAAGACGAAAAAGTCGGCTGGAAAGGTTATGACATCAGTCTTTTGGGATGCACGTGGTATAATACATACTCAACGACTGATTACTGAGCCAGTTATAATCTATTTCACTGCGTATTTGGTTGCAGTTCTTTTCTACTATTCCAAATACTTAGCAATTGTATTATTTTTGAGGAAGAATCTGTTTAAAATTGTACGCATATATTCAAATGATTCCTACAAACATGAATTTTGAACAAATGCTTATGATTTGAAATATAATTTTTGTATTTATGAGTTGTATTAAATTTTGACATAAAGAGATAAAAGGTATCCTATGTCCTTACCCTGGTTCTAAGCTACCTCCCCACCAATTTTCAGCCAAATCGGTTCAGCCGTTCTTGAGTTATAAATGGTGTAACTAACACAACTTTCTTTTATATATATAGATT

>Btry_1307#DNA/TcMar-Tc1

TACAGGGTTTTCCAATAAGAGTGATATGATTTCTTAAAAAAAAACACACTAATTGTTAAGGAAATTCAAATGTTTTTTATTTAATGTGAAGTACAATCGATACAATTAAGTTCTGAATATAACATCATTCAAATGGCCTCCACGACTTCTTTTGCAGGAACGAATTCGATGAACCCAATTTTCGAGTACTTTTTCCACTAAATCAAGTCGTATGTCATGAATAGCACGTTCAATATTGACTTCTAAAGCTTGAAGAGAGTCTGGTTTATTGCTAAAGACCAATGACTTCAAATAACCCCACAAGAAGTAGTCTAATGGTGTTAAATCACAACTTCTTGGAGGCCATTCAATGTCACAATTTCTTGAAATAATCGAATCTCCAAACTTTTCTCGCAATAATTCGGTTGTTGCACGTGCTGTGTGGCACGTAGCCCCGTCTTGTTGGAACCAGATGTTGTCAAGATCAACTTCTTCCAATTGCGGCCATAAAAAGTTGGTTATCATCGATCTATACCGCTCTCCATTGACAGTAACGGTGTCACCAGCTTCATTTCGAAAGAAGTACGGACCGATGATTCCACCAGACCAAAAACCACACCAAACTGTCAATTTAGGTGAATGTAATGGTTGTTCATGAATAATTTGTGGATTTTCTTCGCACCAGAATCGACAGTTTTGTTTATTTACCGCACCACTCAGATGAAAGTGAGCCTCATCGGAGAAGATGATTTTCTTAAAAAATCGTTATCGTTTTCAAGTTGTTCCAAGGCAAAATCAGCAAATTCACGACGTTTACGGTGGTCCAATGGCTTCAGTTCTTGAGTGAGAACAATTTTATACGGATGCAAGCCCAAATCCTTTCTCAAAATCCGCCAGGTTGTGGTTTGAGACAGTCCCAATTCTTGAGAACGTCTTGGAATCGACAAATTGCGGTCTTCAGCAACACTTGCCTGAACGGCAGCAATATTTTCATTACTTCGAGCGGTTCTTTGTCTTATTGGCACTTGAACATTATGTAAAGAAAATGTACGCTCGAAATTTTCAACAATTCGACGAATAGCGAGCACAGGTGGACGATTATTACGACCATAAAATGGCAAAAGCGCACGAAAAGTTGCAATTGGAGAACGATTATTTTGATAATAAAATTGAATAATTTGTAAACGTTGTTCTTGCGTATATCTTTCCATGATGAAATTGTAAACATTACTGAATACAATGAAAAAAAATTACAGATCATGACATATTAAAAATGGCCGAAACGACACTTAGAAATCATATCATCCCTATTGGAAAACCCGGTATAT

>Btry_LINE2#LINE/LOA

GGGATGTAGTGCAGGTCGCCTCCAAGCTGGTGCATCCTGGGTAACGCTAAATGGCCTGCGGCCTTCACGGCCTTACAACTCCCTAGACGACTTTGGCATTGAACTGGTTTTTAGAGTAGGAAACTTATTTGGATTTGGTAGAACCCTGGGCTGGTGGTGGCGGCATTCTGGCACCTGAGTATGATGAGGTGACACTCATCAGAAATGGCTGTCGGGCTAATGTCGCAACTGCCTCCGTCCCGTTAAAACCCTGGCAGGCCTCAGAGTACGTTCCCCCCCGTCGTCGTTAAGTTGGCGTGGTAGGTGTAAGTTGAGAGGACTCCTTCTTTACGCTGGTCGGCTCTGAACGTATTGCCTCATAAGGGCGTACGTCAACCCTCGCCTTGGCCTCGTCACTGAGATAACCTTGGGACCATAAAAGGCGACTACCTTTCCGGGGGACGGATAGCGGCTCTGAGTGGGGACCCTTTTTGCATGGACAATTTAACAACATCGACGGAAAGATGACGAAGGGAGGTGAAGGGGTGTCGAAATCGACACCCAGATCTGTCCCAGGCGGGGCTGCTGCTCCTGTGGCGACGAAACCGTCGCCGAGTAACTGCAGCAACAGGACAAAGACTCCAGTAGGAGTCTCCTCATCAGGCGGCAGGATGGGGGAAGCCGGCGCAAACCCGCCGGCAAAAGAGGTAGTGAAGGGTGGAGCCTTCATACGCGGCGGAATCGCCAGCACTAGCAGAGGAACTTCTGCTAGAATTATGAAGGGATCCACAGGGGGCATTAGCAAGGGAGCTTTAGCTAGAAAGCTGAAGTCGGACCGTCGATTGGCGGCAAAAATCGTGGAACGCTACGGTGGCAAGCATGCTGGTCAGGTATCTGAGCAGCATGCTAATACACTTGAGTGGGCCAAGAAGGTGCTGAGCGAGGACGATAGGGCAGGACTGGAAACAAAGAGAACCGAACCAGCGGTTAAGCGGCAAAGGTCGCACGAGGGAGAAACCTCCCTTGGTAAGAAACCGCGAACGGGAAAGGCGCCAACCTTTAGCGAAATCGCTAAAGGCGCTGGTGCAAGAGTACTTGGGGTGCTCGACCGCAGCAGGGAGGACGGTGCCATCTCCCATGAAGAATGGAAGAGAGTAGCGGCGGCTATCTCTTCGGTCTTCCTGAAGGTGGTCAAGGAAAACCCGGGGCCACCCCCAAAATGTGTGAGTGCCGGTTGGCACCATGGGCTGCACAAGCTCACAAGTTGTGCTGACGAAAGATCGGCCATCTTATATAAGAAGGCAGTCTCTCAGGTGGGGGAGGTTTGGAAGGGAGCCAGACTGGAGGCCGTGGCCAAAGAGGATCTTCCTCTTCGCCCTAGGGCTCGCGTCTGGCTGCCGGCCGAACCTTCCACTGCGAATGAGATTGAGGAAATCCTCAGATATTGCAATCCCTCACTTCCAACGCAGGACTGGAGAGTTATAAGGCTGGAGAGAACCGATGAACCATATCGGCAAGCGCTGATAATGCTAAACGCGGAATCCATCGGTCCTCTCAGCAAAACAAAGGGAGCCATAAGCTATGGTTTCGAAATGGTAGTGCTGAAAGTACTCCCATCGGATGCCAGAGCTGATGGCACCCAAGCTGCAGATGCGGTGGATGTGACAGAGGGCGCTGACGGTCAAACGACCGTGGAAATCGACCCGAATCTCTCGGACGTGGCGAGTTCTGGGGGCGGTTCGTCGATCGGCGACTCCGTCTTCAGCCTCGAACAACTGTTTGAGGAGGTGCGGGTCGATCACGACTTGGGCGCTGAACTAGCGCTCCTGGAGGAGAGTCTGAAGGATGAAATTCCTCCAGATTAACCTCCAGCACGCGAAGGCGGCCTCCGCCAATCTACTGCTTCGTCTGGAACAAGACGGAGCTGATGTCGTCCTGATCCAGGAACCGTGGCTGACTGGTAACGGGATCTCTGGATTGAGGACAAAGAGCCATAAGCTGCTGGCGGCCAAGGATGCAGGTAGAAACAGAGCCTGTATGCTGGTCAGAAATGAATTAACGGTATTTCTTTTACCTAATTTCAGCAACGCTGACGTAGTGACAGCGAAACTGGAGTGTGACACCGGAGATTTCTGGCTTATCTCCGCGTACATGCCACACGATGATGTGGTGGAGCCACCTCCCTTATTGCTGAGGAGGACCTTGGCTGAAGCGTCCCGGAATGGTGCCGGCGTCATCATCGGTTCGGACGCCAACTCGCGACACTCGATCTGGGGGAGCTCGGATACCAACACTAGAGGTGAGTCGCTTTTTGATTTTATTGTTAGCGAAAATCTTCGCATTTGTAATAGAGGAAACTCTCCTACTTTTGTGACCGCAGGCAGGGAGGAGGTTCTCGACCTCACCCTAGCCTCACAAGGGATTGCCCCGCTGATCTCGAACTGGAGGGTCCTCGACGATCACTCCTTTTCTGATCACAGGTACATCGGGTTTAGTCTAGTCGGAGAAGGTCCGCCTAGGAAATCTTTCAGAAATCCTAGGAACACGGACTGGGAAGGTTACCGCAGGAGGCTTCGGCAAACTCTCCCACGCGCACCGGGGGTGGACTCGTTGGCCACCACCGATGCGGTGGAGGAGTGGGTTGAAGTCTTCAGCTCGGCGTGCAATGCTGCACTGGAGAGCTCCTGTCCACTGAAAACACCGAAGGGCAGAGGGAAACCTCAATGGTGGACTATGGAGCTCTCGGAAATTAGGGCGTCCTGTAGACGCTTATTTAACAGGGCCCGCAGGAGTGGGTTATCTGATGACTGGGCTTTGTATAAAACAGGACTTTCAATATACAAGTCCGAGTTAAGAAAGGCTAAGAGGATCTCGTGGAAGAGCTTCTGCGAAAAAGTGGAAGGCTGTCACGAGTCCTCTAGATTCAGGCGTATTCTCGCAAAAAACCCAGTGTCCCTGGGGTATCTCAAGGACGTAAATGGGAAGTGGGCCCTCAGCAGTGAAGAAACACTTCAGATGCTCCTTGATGCTCACTTCCCGAGCAACACGTCCTCTGTGAGGGGATCTCTCGGGGTGCCGCATGACGATTGCGCGGCCTTTGTCGACCTTCTGGATGAGCGACACTTGACGTGGGCGATTAATTCGTTCAAACCGTTCAAGTCCCCGGGACCGGACGGCATCATACCAGCGCAGCTGCAGCAGGCTGTTAAGGTGTCATGCAGCTGGTTGGCACCTATCTATGCGAACTGCATCAGATTAGGTTACATCCCGGGGGCCTGGAGACGAGTCAAAGTTACGTTCATCCCGAAGGCTGGCAGGGGCTCCCATGTCACGGCCAAGGATTTCAGGCCCATCAGCCTCTCCTCCTTTTTGCTAAAAACTTTGGAGAGACTGATGGACTGGTATATAAGGAGGCGCATTCCGAGAGACTATCTATCAGGAGCGCAACATGCTTATCGTAAAGGCAGGTCAGTGGACACTGCCCTGCATACTATCGTGTCATGGCTTGAGGAAGCCATTGAGGCTAAGGACTTCGCTGTTGGGACCTTCCTTGATATTGAGGGAGCTTTCAACAATGTCCTGCCTGAGGCAGTCGTAACTGCTCTTGACGGTCTTGGGGTTGAATCGAACCTCAAGCACCTCATTTTCAGTCTTCTGTGCGACAGAGTGATCGAAACAGAATGGGGCAGCGCGCGTGCGCGGCGAAGTGTGAGTAGGGGAACCCCACAAGGTGGGGTTCTCTCTCCTCTTCTATGGATCCTAGTCGTTAACGACCTGCTCAAAAAACTAGAGGATCGTGGCTGTCGGGTGATTGCCTATGCAGACGATGTAGCACTTATGGTCAAAGGAAAGTTCCTTAGCACCGTGTACGAGTTGACGCAGGGATATCTCAGCGTTGTAACAAAATGGGCGGTCGGAAGTGGACTCGCCATCAATCCAAATAAAACAGAAATGGTTTTATTTAGTAGAAGATATAAGATCCCGGTGGCACCGCTGCCGCAAATGGGAGGTATTCGCCTGCAACCGGCGGATACAGTGAAATATCTAGGGCTCATCCTGGATAGGAAGCTTTCATGGAAGCCGAATATCGAAGAGAGAGCAAAAAGGCATCGATTGCCTTATACTGCTGTAGAGGAGCCATTGGCAAAAGGTGGGGTCTCTCACCGAAAGTGGTCCTCTGGCTCTACGACACCATAGTCAAGCCCATTATGTTCTATGGAGTCTTTATGTGGTGGAGGGCTCTAGAAAAGACCACACTTGCAAAGAAACTCGAGAGCGTTCAACGGGCGGCGCTCATCAGCATGTGTGGTGCACTAAGGTCCACTCCAACCATGGCACTTAACGCCATCTTGAACAGTATGGCTGCGAAAGTTGCTATCAGACTCAGAGAATCTGGGTTCCTAAAGAACACGTGTCTGAACACTCGGATATCCTCACACACTTTGACTTCATACCGGATCACTTGGATCATGGAGTCGCCAAACCGAACTCTGGCGGCTCCTTCTCTGCCCATATACCGGCGAGGGAGTTGTGGGTGGGAAGAAGCCGTTGGAGGAGAGGGGCAGTGAGCTTCTTTACGGATGGGTCAAAGCTTGGGGGAAGGTTGGTGGAGGGGTTTACTGTCAGGAGCTCTCTATCAACTCCAGTTTCAGACTTCCTGACTATTGCAGTGTCTTCCAAGCCGAGGTTGCAGCCATTAAGGTAGCAGTAGATTTACTGCTCCGGAGTGCAGCCTCCTTCAGAGAAGTGACCATCCACTCAGATAGCAGAGCGGCGATACTAGCCTTGAACTCATTAACAGTGCGTTCAGGGTTGGTCGAAGAGTGCCTAACCTCGCTATCAATAGCATCGAGTGTCTTTGTGATAAGACTGGTATGGGTGCCAGGCCACAGCGGAATCGCAGGAAATTGTAAAGCTGATGAGCTAGCAAGGAAAGGCACCCTAGAACCGCTATCGGTAGAATGGGAGCGGGTCGGTGCTCCCGTATCCTCTTGTGTTCTACTACTAGATAGCTGGGCTTCGCGGAAGCTTGGCCAGCGCCGGTACATGCGCTGTCGCAAAAGCCTTTTGGCCCAAGATCGATCGCAAGAGATCTAGTGATCTCTTTGCCCTCAGTAAGGCTAGCCTCTCCCTAGTTGTGGGGCTTTTAACAGGCCATTGCCCTATTGGCGTCCACGCTGTAAGGCTGGGAATCTTACCAGACGCCATCTGCAGAAGCTGTATGGAAGAAGATGAGGTGGAAACAACTCAACACTTCCTTCTTGACTGTCCCGCGTTTGGGAGGTCAAGACTTAAACACTTGGGAGCGCATACCTTCAGACATCCCACCGAACTGGCGGGAATGGAAATTAAACGCCTGTGCAAATTTGTATTGGCTACTAAGCGTTTTGCTAATTTATAAGTTCGAACACAGGAGTTCTTCTTTTCTATGGCTTCACAAAGGACTACATATAGCAGTCCACGTGCGATCTTTGATCAGCCATCTAACCTAACCTAACCTAACCT

>Btry_4497#LINE/Jockey

TTTTTTTTTTTTCAATATTATTTTATTTATTGAATCTGCTTTTTGTTTTAAAAGCCTAACAATAAGTTATAAAATCTTAAATCTATTTAAAAACTAGACAGGCTCAAGCAAGTGATTGAGCTGTTTTGGTGATACGTTGCTCCTTAGTACGCAGGCATATCTCTTCTTTTAAGGCGTGTTTGATCGCAGGAATGTACCAAAGCATTAGCCAAAGGGTTTGGGTGATCTCGGAGTTTAGATATATATTTAATTCTGCTGTCTTCTACTTCTTTCTTTACCATAGGAATACCAAGGTCTTTATGGATATTTTCATTACGCATGTACCATGGTGAACACGTGATTGTTCTAAGCATTTTTGATTGGAACCTTTGTATTATATCAATATTAGTTGCACAGGTCGTACCCCACAGTTGAATGCCATACATCCAAATCGGCTTTATGACCGCATTATATAAAAGCACTTTGTTGTCTAGGCTAAGTTTGGAGTTTTTATTTAAAAGCCAATTTAAATTTGCAGCTCTAATCTTCATACATGTTATTTTACTAGAGATGTGTTTTCTCCACGTGAGCCTTCTATCTAGGTGAATACCAAGATATGTTACTTCGTTCGCTTGGGGTACTAAAATATTGTTTATTTTTACTGCCGGGCACATTTTTGGTCTTAGCGAAAATGTAACATGCTTACACTTCTGTTCATTCACATTTATACGCCAGTTCGCTAGCCATTCTTCGACAGAACTTAAATGCTCCGCTAATATTCTTGATGCTAAAATGTGGCATTTGTTACGGCTCACTATAGCTGTGTCATCCGCAAAAGTTGAAGTTAATACATTATTAGCTGTTGGAAGATCTGCTGTATATATGATGTATAGTGTTGGGCCTAAAACACTGCCCTGGGGTACACCAGCCCTTATCTGTCGTTCATCAGATATGAAATCTCCCACTTTTACCATAAATTGTCTATTTTTTAAATAAGACTCCAATGTTTTATACAATTCTAAAGGTAGAATTTTTTTAATCTTATATAAAAGGCCTTCATGCCACACCTTATCAAACGCCTGAGCTACATCTAGAAATATTGCTGAACAGTACTCCCTGTGCTCAAATGCTTTTCTTATTTCGTTAGTAATTCTATTTACTTGCTCTATCGTGCTATGTTTTTCACGAAACCCGAATTGGTGCGTTGGTATTATATTATTTTCGTGGAGGAAAGGAGACATCTTTGATAGTAACACTTTTTCAAATATTTTAGAAAGACAGGGTAGAAGACTGATTGGTCTGTATGAAGACGGCTGTGTTAAGTCTTTCCCAGGTTTATCTATCAAGATAATCTGCGACTTTTTCCATGAAATTGGATAGTATCCGAAACTAAGAATTGCATTGAAGAGCAAAGAGAGCACCTCTACAGCAATATTTGGTAACTCAATTAGCATTTTTGGGGTAATATTATCATGTCCCGGCGACTTTTTTGGATTAAGTTCTTTTATGATTCCAATAATTTCAGAAGGTGAAGTCTTAAAAGACTCGAGCGACTCTTTAGCTGTGTTGGGTAAGATTGACAGCTTAAAGTTGTTCTTTGGCAAATTGGGTTGAAATACCTTTTCTAGGTGATTTGCAAAACAATTAGCCTTTTCCTCGTCACTTCGAGCCCAATTTCCACCCAAGTCTCGTATAGGCATGTTGGAGTCAGTTGGTGGCTTCATGGACTTTTGGGCTTTCCAAAGGGAATTTTGCTTGTTTGAATTTGGACACAGCTTCTTTATATACATTTCGGTGTTGAATTCTTCCTCACGTTTAAGCGCTTTTTTTAATTTACGTACAGCAGATTTCAATTGAAGCTGAGTGGAAGGGGAGCGATTTATCTGCCATTCACGTCTTGCACGCCTTTTTTCATTTACAAGCTTTTCTATTTCTCTATTAGAGATTTTTCTGAAACCAAGCGGCTTATAGTTTTTGTTTGGTGTTGCTAAGACAGCTGCGCTAGTTATTATATCATTGAGTTCTCTTATGCTTTCGTCAATATCTCTTCCTGTATTTATTTTGTACTCAATATTAATGTGGCTACTCACGTATTTTTTATATTTTAGCCAATTCGTTTTATAAGAAGTTAGACCCACTTTTGGATTAACGAATATGGGTTGTTCACATAACTTTATTAGTACAGGAGAGTGATCAGAAGATAGATCAGTACATGTATCAGCTGTTATGTGCGATTTATCTATATTTTTGATTACAGCAAAATCAATTAAATCTGGTATTTTGTTACGATCACTAGGCCAATATGTCGGCTTACCAGGAGATATTATGTCAAGGTTATTGTGCCTATTTATAATAGTGTGATACAGCTGTCGTCCTTTCGGATTAATAAGACGTGAGCCCCAGTACGTGTGTTTTGCGTTGTAATCTCCACCTGCTAGAAATCTTTGACCTAGTGTTCCAAAAAAGTCTTTAAATTGGATGTCTGTAATTTTAAACCGAGGTGGGCAGTATATGGCCGTCAGGTTTAAGTCCCCGCAGCGATTTTTTATAGTTATTGTTGTAGCTTGTAATTGCGCTGTGGCATAAGATTCTAATGTGTGGTGATTTAACCGTTTTCTAACCAACACTGCTGTTCCTCCATGTGCCTTTCCATCTGGGTGGTTTGTAACATAGAGTCTAAATCCCGGTATAAAGAAATTGTTTTTGTTCGTAAGATGAGTTTCTGACAATAGCATTACATCAATATTATTTTCATCCAGAAATCTAATAAGTTCCAATTTATGTTGGTTAACACCGTTAGCATTCCATAAACAAATGTTCAATACACTCATTTTTTACTTAAAAAAGTTTGCAACATTTGGTTTTGTGATTTGATTATCTCTTGAATCATATTTTGCATTGAAGCCATAAATTGTGTCATACATTGGGTAAGGTTGAAAATCATAGTTTCAACGCTTCCATTTGGTTGGTTTTGGGGCAATTGCATTTGTGCAGTATTGCCTTTCACCACGTTTGCATAGCTCCCTTGTGTAGCATTGTCTTTAAGATTTACTGGTTTTGAAATATGGACGGGGATTTCAATATTTTCATTAGATGGAATATTCAACATTTGGTTACGACGTGCTTGAATGCCCTGTGACAACTTCGATTTCAAATCTTTATATACAGGGCAACCCCTGTAGTTAGCAGTGTGATTTCCTCCACAATTGCTGCATTTTTTATTTAAATCCTCTTTTTTAAGAGTACATTTTGAAGTAGGATGTAGATCACCACATACCACACAGACACTGCGTAGAGTGCAATATGCTTTCGTGTGTCCATACTCTTGGCAGTTTGTACATTGTACTGGACCATTTCTCTTATGTGGTTCCTCAACGGTGATTCTGCGATGGAGAAGATATTTTAAATTGTAAATCGGGTGTATTTCATTTTTCTTTAATTTGTTAGAATCTGGCATCAATTCTATTTTGAACATTGGTTGTGGGACTTTGTTCCTGTTAAAAATATTTACTACTGATTTAATGCTAAAACCACCTTCTTCCAGTGCTTCTTTAACATCGTTAGAGTCTACCGAAGACTCTATACCTTTTATGACAACAACTAGGCCTTTTGAGCTCTTCAGTTGGTAAGAGTAGTAATTCTTGTTATTATTTGACAAAAATTTTACAATTTCCATGAAACTTTTTTCAGTGTATGACTGAATTTTTGTTTCATCTATATTGCCTTTTTTCAAAGGCACTATATGAAAATTGTTTGTGCCTATTATATTGCTCATTTTTGAAACAAGCGCATTTGAGCTACGCTCACGCAAATATATTGGAGGGGGTTTGGCATTAACGACTGTGGTGGTACCCTTCGCATCGTCGTCTGAACCATTGCTTAGTAAGGCAAATCTGTTGCCATTTAGAATTTCTGGCTTTTTACTATTTGGCGTGCCTGCTTGAAATTTTTTGGTATTGACCGCTGATTTAATAGGACTCAATTTCCTTTTTACATTAATGTAGCGATCAATACCAGTCTGAACAGATGGCCCTTTTTTGCTTGGTACATTCTCACCTTGCATTTTGTTTTCCTTCGCTGTCCGGGTGCTTGCTGGTACCTGCATAGTGGCTGCTGTCAGTGCATTTGTTGTTGCCCGATTGCTGTTTTCTGCTGCTTCTGCTGACTGCGCTTGCTTAGTCTCTCTTTCAGCTGATCGCTGCGATGACTCATCATCGCCGTTACATTTGTTTTTGCCAGGAGTTGCTTTTGTTGGCTCGACAAAGCTGAAGTAGGCATTTAAGGAATGCCTACGGCCTTGCTGGTGAGGCTGCGAAGCACTCATTATTGTTTGTTTTTTGTTTTGTTTTTTGTTTTTATATATTTGTTTCGTGCACTATTTGTTTATGTTTATTATTAATAATTTGTGTGCACTGTTTTTTATTTGTTTGTTTGTTTTTATTTTATTATTATTGTTTGCTTACTTTTTGCAAAAATTGAATTCACGGAGCGAATTAAAAAACACGTCCGTACACCTTGAAC

>Btry_1671#DNA/TcMar-Tc1

ATATATACAGCTGTTGACAGCTAATTAGAAACGCTCCCTTTTTTGTAGGTGGCTGATGAGTATAATGATAAAAATTTTTGATATACCGTTATTTATATTCCTAAAAGTCTTGCATTGTTCGTGTTACTCTATTTACTACTAAATTCACTCTGTTTTGTAGTTGTGAAGTTAAAGTTCTTTTAATTTTTTCCCAAATAGTAAATGCGTTACGATATAGAAGGTGAATTAAGTGCGACAGCTAATTAGAAACAGTTGTTTAAAGAATTAAAATTCCTCAAGGTGTTAATTTTTATTAATAATATTATTGATTTCTGGTAAGGGTGTGTTTCAAAGTTGTGGTTTAAAATATATTAAAATGTATTATTCAAGTTTTTTAATGAGGAAAAGCTGCAGCTGTACACCTACCCTTCGCAGCTACATTTTTGACCTTCGTCAGTCTGGAAAGATGTGCAAAGAAATTTCGAATAAGGTCAAATGCTCAAAAAAAATTGTTTTGAATGTCCTTAAGCATATACAGAATTTTAAAACTATTTTCAATGTTGCACGCAAAGAAAAAACCCGAAAAACTTCAACAGAAATATACCGCAGAATTACTACTATCTCCCGAAGAGACCCAAAAAAGAGTTCCACTGACATTCTACATGAAATTCAAGACGAATATAATGTCCAAATATCCAAGAAAACAATTGCCCGGCGTTTGGTCGAATTTGGACTTCACGGCAGGGCAGCACGCAGGAACCCACTTCTGACTAAAATTCGTAGAAACGACGCGTAGACTTTGCCAACTCTTATTGGTCTAAGACAACAAATCAACGGAGGTATGTCGTTTGGAACGATGTAACAAAAATTAATCGCTTAGGTCCTGATGGTCGAAGGTATGTTCGCCGTCCAATAATTCAAGAATTTGATCCTCGCTATGTTTCACGCGTAGTTGAGTATGGTGGAGGATCAATCATGGTGTGGGAATTTTTTATCTGGAATGGTGTTGGTCCACTCCATAAGATTGATGGAATTTTAAACAAGGAGAAATACGTCGACATCCTAAAAAATGTCATGTTGCCATGGGCTGAGGAAAATTTGCCTGTTATAAGGAAGATAACAATTCAAAGCATACGTCTAAGTTGACACAAAAGTTTTTTAATGAAAATTCTATCAATGTTTTGGAGTGGGAATCGTCCAGCCCAGACTTAAACCCTATAGAACATCTCTGGGTGATGTTAAAAGAGCCATATGCACCAAAAATATCCCAAATATGGATGTTCTTTGTGATGAAATTAGAACGGCATGGCAAGCTACACCATTGGTGCGCTGTCAGAGTCTTATAACGTAAATTCGGAATCGATGTGAAGCAATATTAAAGTAAAAGAGTTAAGCAACAAAATACTAACTGAAAGTAAGCAAGTTTTTGTAATATCATTATTTTTTGTTGATAAAATATTTTCGTTTCTAATTAGTTGTCGCACTCAAATCGTTGATTCCACATGTTTTTGTTAAAATCTTCAAAACAAAAACGAATTTTAAACAAAATTTTTACTATTAGAGTTTAAGTTTAACATAGGTTTCAATAAAATAAGTGAAAATACAATAAATATACGTGATTTATTGGAAAATAACATCATTTACTTCAAAATAGGTGTCGTTTCTAATTAGCTGTCAACAGCTGTATAT

>Btry_3984#LINE/Jockey

CAATTCCCACGACAGCCGGTTCTACGCACCGGAATTGACTCGGATTTCATCCGACCAAGGGCTGTTTTTTCGGCGATCTAACCCCGTTTGGATCGGTAAGTGTTAATTTGTGTTTGTCGTCATTGGAGCAATGCCTTGCAGCAGGGTTGCTCCGTATCCTCCTGACTCGTGCTGGCATTGAGTCCAACCCGGGCCCGGAGGAATTTTTCTGCTGCGTTTGCGCAAAAAGGCTCCATCCAAACTCCACCTCGGTCAGGTGTAATACATGCAATGGATGGAGCCATCTTAAGACCTGCTCAGGCCTTAAGACTCATAGGGAGTGGACCACGAGTTACGTGGCCCCATGTTGCCTACGCAATACTGCGACACTAGCCTCTACGGCTGTGCAATCTGCACATAGTTCGCGCGCCGCGCCGCCACTCACCCCGAGTGGCCAACAGAGGACCTCCACGGTCCCCAGGAGCTCAAACAGGCAACATAGCTCCCACTCTGACTTGTCCCCCCCAACCTTCATCCCGCTCCAATCCTCGTGCGAGAACCAACCAGCAGCTCTTGGTCCCTCGCACAGTCTGTTCCGTGTGTCAGACCGTCATACGTCGGAACGTTACATCAGTCAAGTGCAATTCTTGTACTGGCTGGTGTCACTTTTCGGCGTGTTCCGGCCTGCGCACCACACGTGAGTGGAGTGAGTCGTACGTTGCCCCATGCTGCAGGAGTCTGCCCCCGCAACTCACAACAGTGGCGTCGCCAACCAACACCCCAGCGCGACAACCGCCCCTGCGGGTTCTACAGCTGCAACAAATTCCACCCACACGTCACTCCCTCACCCCCAGGATCACCCACGGACACCCGCGACAAACCCGGCTACTTCAATTTAACTGCAACGGACTCCAGAGTAAGATCGAGGAGATAGTTGCACTTATGAATCGGGAACGCGTATCGATAGCTGCGGTCCAAGAAACCAAGTTAAACAGCCGCTCAGATCTTCTGAGTTGTGCAGGTTTCAACGTTATACGTAAAGATCGCGAGCGAGATAATGGTGGTGGCCTAGCCTTCATATTGCACAACACCGTGCAGTATCGTCTAATCGATGAAGACATCGACCGCAGGGATACTACCCTAGAATGTCAGGGTATAGCTATCCGGTCAGGCGATGTCGAGCTCGAAATATTTAATATATACATCCCCCCAGTTACATGTTGCCCTACAGGATATCACCCGAATATAGGTGCGCTACTTCGTGGTGAAAACCGTTTGGTACTAGGCGACTTTAACGCGCACCACGATCTTTGGCATTCCTGCCTGTCAAATGATCGTAGGGGGATGGAGCTGGCGGAACAGATTGACGATTCGACATTCTGCACAATGAATGACGAAGCCCCCACCAGAGTTATGGGCACCTGTAATAGCTCGCCCGATATTACCATTGCTAGCGGTGGTCTGATAAATAGCATAACCTGGCGACCTATGCTAACTCTCGCATCAGACCATCTGCCCATAATTATCTCGATCGAGAAACCTCCCGATTTTGTTTCTGTGGACAACCGCACCTTCGTTAACTTTAACAAAGCTAACTGGGTCGGCTTCACAGAATTTACTGAGAGCACCTTCAACGCTCTACCCATTCCTACGGACGTATGCGTTGGCGAACGTCAATTCCGCAAGGTGATCGCAGCAGCTACCGCTCGCTTCATCCCGGCTGGAAGAATAGCGGAAATCCGTCGAAGAATAGCGGAAATCCGCCCCAATTTCCCAGCCGAAGCAGCCGTTTTAGCTAATGAGCGCGACACCTTACGCCATGCCGATCCCGGGGATCCCCGAATAAGGGATCTCAATTTGGAGATTCGGCGAATGGTAAACCAACATAAGCGGACGAAATGGATAGAGCACCTGAAGTCCTGCAACCTCTCCACCGGTGTGAGTAAGCTTTGGGCTACTGTCAAAGCTTTGTCTAACCCGAAGAGACATGACGACCGAGTTGAAGTTCGGACCCGAAGAAGTGCGCGAGCTATTTTAGCCGGCAGTTTACACTGCACCCTTCGGTAGACAAAGCCAAACGATGTGTTAACCGACGGCTGCGCAAAATGCCAAACGACTGCGCGCCACTTACTTTCACCGATGAGGAGGTTCAGAGTGTCATCAACAAAGCAAAATCATCTAAATCGGCGTCTTCCATCATTGGCCCTGATGGAATCAACATGCTAATGCTAAAGCATCTAGGCTCGACGGGAGTAAGCTATCTCACCAAGGTCCTCAACCTGTCGCTGACCACTCTTCAAATACCCGATGTGTGGAAAGTCGGAAGAGTGGTCCCACTACTGAAACCTGGGAAACCCGCCAACAAAGGGGAGTCCTATCGTCCGATAACTCTCCTCTCCCCAGTAGTGAAGACACTTGAGGCCTTGCTACTCCCGACCTTCACTCACCACCTGAGCCTAGCCAGCCACCAGCATGGATTCCGAAAAGTGCACAGCACCACCACAGCACTTAGCGTCATAAACGCCCAGATAGTTCGTGGCCTCAACCAGAAACCACCCTGTGAGAGAACGATCCTCGTAGCGTTGGACTTGTCAAAAGCTTTTGACACAGTCAATCACACAACGCTACTGCAGGACATCGAAAAAACTACGCTCCCTCCAGGACTGAAGAGGTGGACCATGAACTACCTGAGCGGTCGTCAATCATCCGTACTATTTCGAGGTGAAACATCTAAACTGAGAAGAATTAAACAGGGGGTTCCGCAGGGTGGTGTCCTCTCCCCGCTACTGTTTAACTTCTACATCTCGAAACTCCCACAGCCACCAGAGGGAGTTTCCATAACCTCGTACGCTGATGATTGCACGATATTGACGTCGGGCAATGGAATCGATGGCATGTGTTCGAAGGTAAACAGCTACCTCTCCGACCTTTCTCGTTTCCTCACTGCACGGAACCTAACACTTTCCCCCACCAAATCCACAGCGACCATATTTACGAACTGGACGAAGGAGTATAGACTTGACCTTAATATTGCAGTCGATGGCGTCAAAATTCCGACTGTCAATAATCCTAAGATTTTAGGTGTAACTTTCGACAGTCTATGCTCCTTCACTCCTCATACGACCGCGATTATCGCCAAAGTACAGAGCCGCAACAAAATCCTCAAGTCGCTAGCCGGCAGCACATGGGGAAAAGACAAAGAAACGTTGTTGGCAACATACAAGGCAATCGGCCGGCCGGTCCTCAACTACGCAGCACCAATATGGTCGCCTGGATGCAGTGGTACGCAGATGAGGAAACTCCAGACCTGCCAGAACACTGCACTCCGGACCACGACAGGATGCCTCTTGATGTCTCCCATCGAACACCTACATAGTGAGACGCTTATGCTCCCAGTTAAGGAGCATAATGAACTCCTCTCCAAGCAGTTCCTGCTGGGATGTTTTCGCAGAAATCACCCCTGCAGCCATCTGCTTGGAGCGGAACCGCCTCCTAGGAGCATCAAGAGGTCTTTCCTTGACTACGTCGACGACGTCGTACAGTACGCCGACCGGACTTCGGACGCAACTAACTTCCGACAGGTACTGACCGCCATTCACAGTGGAGCCATCAACACCTTCACCGACTCCCTTCCAGTGAATGGCGTTCTTGGAGTCAAACCACCACCCATCGCAGACGAAGAGCTCGAGTTGCCGCGAGAAACGCGAGTGACCCTTGCGCAGCTTCGTTCTGGATACTGTAGCAGGTTAAACTCCTACTTATCCAGAATAGACCCCGACATATCCAATGTATGTCCTGCATGCAACGAGTCTCCGCATGACACTGACCACCTCTTTGCATGCCCTACCAACCCCACTCATCTAACACCCTTTTCCCTTTGGTCCGACCCCGTCGAAACAGCACGTTTCCTGGGCCTCCCGTTAGATGACGTCGACGACAACANAGATAACCCTTACCATCCTAACGGGGATTGATAAACCGTTAAAACAACAACAACAACA

>Btry_6435#LTR/Pao

ATATATTTATATAAATAAACTAATAATTAAATATTACGAACAATTTAATTTACATGTTCAAAGTCCAAATTGGCACATTTTCGGCAATACCCCTTGTTCTTTGATAAGCAAAAACAAGCAGGATTGCGTATATACATATGTACAAAGTGCACTGATCTCTTATACGAGCACTCAGTTGCAAGTACATACAAACGGATTCGTCTATAGGTATACCCTATAAGACATACAAACACAAAACAAATTAATAAAATAAATATTGCATATTTTAACAATTAAATAATAATAAATAAATTCGGTAATACTTTTATACTTTTTTCGAGATTAATTAAAACGAAACGCGTTATTTAATTTAATCCGATCAACAAAATAAAATAAAACAAAACATTTTACAATTTTATTATTTACAAGAGACTTCGCTTTGTAATCAGCAATTTATTTAAAGTCAATCAGTTTACACTGATAGAAATTATTTCATAAAATTTATTTGTTCTAAATGAACGCAGATTCTAAAGAATCTAAATCTTCACAACTACTGAAAGTTCCAAAAATGATTTCGGACGAAAAAAGTCCATGTACACCTGCAGAAGCTACACGCTCAAAGCAAGGTGCTACAAAACAAAAAAGGGGGAAAGACATATATACAGTAAATTCATTGCTGAGAGTGACAGTTTTATAAGATACTGCACTCGATTTTCATCTTCACCGATTCAAGACAATTCTGAATCGGTATTAGAAATCAAAAAAGAAAATCTTGACAATTTCTGGACACGTCTCCAAGCTGCATATGACGCAATAGTAGAATCTGACGACTCAGATCTACCAGAAAATTTTAAATCCTCGGCTTACGCCAAATATGAAAACTGCTTAGACCAATACGAAGAGACAAAAGCTATGATCTCTGATCAATTAAAATTAATTAAAGCAATTGCACCTACTCCACAACCGAGAGTAGAGCTGCCACAAATACAAACCCAAGAGGCAAGTTCAGGCATTCACCTCAAAGTGCCAGCATGCGACACAGAAATATTTCATGGAGGTTATGAACAATGGCCGTCCTTCCGGGACATGTTTACAGCCGTGTACATAAACCATCCTAAATTATCACAAGCGCAAAAATTGTATCACCTCAGATACAAAACAAAAGGTCAAGCAGGCGTAATAGTCAAACAGTTCGCTCTTAATGACGAAAATTTCAATTTTGCTTGGGAAGCTCTAAAAGCACGATACGAAAACGAAAGAATATTGGTCGACAAACAAGTGACGATATTAATGAATTTACCAAAAATTCAGAAAGAAACAAGTGAAGAATTCATAAAACTTCAATCCACTGTTTCAAATTGTTTGTCGGTTCTATCGACACAGAATATTCCCACAGACAATTGGGACCCCATTCTGGTAAATATATGCACCGCGGCATTACCAGAAAAATCGTTACTTTTGTGGGAGCAATCGCTCTCATCACGAAGAAAGTGCCCAACGTGGCAACAAATGAAAGATTTCTTAACTACCCAATATGAAATTGCGGAAAGGGTAGATAAAAAAATAGTCAGAACTAAAAACGTTCAACACGACCTAAATCGAAGCTTCAATAGACCCCAAGCAAGTAGCAACAATAATTTAAATAGAAGCTTTTTTAAAAATCAATCGTTCACATCCGAACAATATAAACAAACGTCATGCGAACTATGTAGAGGAGGGCACAAGCTAAAATCTTGCGAAAAGTTTAAAAAAATTAATATTAACGAAAGGAACAACTTTGTCAGATCAAAAAGACTCTGTACAAACTGCTTGTCCCACTCACACAATCTTAATAATTGCGAAAGCAAATTCAATTGCGTATATTGCCACAAAAGGCATCATTCAATGTTACATATCAATAACTTTCCCAACACACCTCAAAGTAGCGCTTTCTCAAAAAAAGCCACGGGTTTAGTTGCAACAGCAACTCCCGAAACACAAAATCCCGAAATTTGCCAAGAGACACCATGCTGCTCAAAGGCATTAAAAACTCAAACGCTACACAGCGAAAATCAAAGTAGGGTACTACTACCCACAGCAGTTGTCTCCATCGAACACCGAGGAGAACTGTTTAAACTCAGAGCCTTAATAGACCAAGGATCACAACGATCATTTATAGCGTCTAGGGCACAAAATAGGCTAAAACTGCCAACAAAACAAGCCAACTTTGAAATTACGGGAATGGGCGGAAGAGTAGTTCAAAACTCAAATAAAATCTGCCCCATTACCCTAATTTCCCCCCAAGCGGATAAGCGCATTCAAGCAGAAGCTATTGTCTTACCGCAACTAACAAACATGCTTCCAAGCTATCATATAAATAGCAAGCATTGGCAAAAGGTTTCACACCTTAAGTTAGCAGACCCCAACTGCAACACCCCCGCTCAAATAGATCTTCTATTAGGCAGCGATCTCATACCACAAATAATACTCGAAGGTATTGAGAAAATTACAAAAACACTTCTGGCGCAAAATACCATTTTCGGATGGGTCCTAAGTGGACTAGTTGCGGAACCAGTCACAACAATGACAACTCAAGTTGAGGAAATCTCAAACGAGTACCTCAATTCACAATTGAGAAAATTTTGGGAGTTAGAAGAACTCCCCCATTTCAATCACAACCCCAGAAGATCAGTATTGTGAAGACTTTTACAAAGCCACAACTACTAGATCAGATAATGGTCGGTATGTCGTACGACTACCACTAAAACAACAATTTCCAGACACACTCGCCTTAGGTCACTCTCGCACCTCTGCAATACAGCAGTTTCTAAGTATGGAAAAAAACCTACTTAGAAAAGGTGAGCTCAAACAAGATTATGATGGTGTCTTAGAAGAATACCTCCATTTAGATCACATGGAGGAAGTAAGCCCATGCGAAAAAATCATAAAAGGCAAATATTACTCATTCTACTTGCCACATCATGCAGTAGTAAAGCCTGACAAAAAAACAACAAAAGTAAGAGTTGTCTTTAATGCCTCAAGATCCACTAGCTCGGGGAATTCCCTAAATGATATTCTATTTACGGGACCCACACTCCAACCAGATTTAATGCTCCTCATATTAAATTGGCGTATATACAAATACGTATTCAATGGGGATGTCGAAAAAATGTATCGACAAATAGTCGTACATAAAGAGGATCAAGATTTTCAGCGAATTATTTTCCGAAAATCTCCCAATAGTCCACTACGCGACTTTAAATTAAAAACAGTTACCTTTGGCGTAAACTGTGCCCCATACCTAGCCATTCGTACACTCCACGAACTGGCAGAAGACACAAAGTCAGAATTTCCTCTGGCAACACAAGTGTTAAAAACACAAACGTATGTAGACGATATTCTGTCTGGAAGTCACAGTCTTCCACAAGCATACGAGGCCTTATCACAGGTAATAAAAGCCCTCAATTCCGCAGGGTTTCCATTAAAAAAGATTACGGCAAATCACCCAAATATATTAAAAAATATTCCAAAAGAAAATTTGTTGGACACTAATTTCCTTATATTCGAAAAGGAAAGTACAACAAAAACACTGGGCATCCAATGGAATGCGATATCGGACCAGTTTTCATACACGACTGAGTCCATATCCGCATTATCCGCCATAACAAAAAGACAAATTTTATCCTCGGTGGCAAAACTTTTCGACCCCGCAGGATGGCTTTCGCCAATTATGATACAAGCGAAAATCCTGATACAAGAATTATGGCTAGATGGAACCGACTGGGACGAACAAGTGAAACCTCTTCGCTTAGAAAAATGGTCCCAGTTCGCTAATAATCTGAATGACATTTCGCAGATACAAATTCCACGGTGGGTAAACTATTCCCCCGAATACAAAGTGGAACTACACGGCTTCTGCGACGCCTCTGAAAAGGCATATTGTGCCACTATATATGTGCGCACACAAAGCGATATCGCAACCACAAGCCACCTACTAGTGGCAAAAGCAAAAGTGGCTCCTCTAAAAACGATAAGTCTACCACGACTTGAACTCTGTGGCGCTCTGCTACTAGCAAAGCTAGTTTCCATGGTGCAGACCCACTTAAACATGGCAAAATACAAATTATATCTATGGTCCGATTCCGAAATAGTTCTAGCCTGGTTAGAAAAACCACCACATGCGTGGAAAACGTATATTTCTAACCGAACGTCTCAAATACTTGACCTAGTGGGATCAGCCACTTGGCGACACGTAGCCAGTGCTGACAATCCTGCTGATCTAGGTACAAGAGGGTGCAAACCTCTGCACCTTGCCACCACCACCCTCTGGTGGAATGGCCCCCGATGGTTAACAGAATCTCCCGATTCTTGGCCACAATCGCCCATGCGCAACATAATTGCCCCAGAAAGTCGAAAAATCGACACCTATCACACAACATTGGATGATACTGACATCCTTGAACGATTTTCATCGTTCCCCCGAGCCCTCAGAGTAGTCGCTTATATGCTCAAATTCATAGAGCGACTCAAACTTAAAGTTAAAGGAGCAACTTACCCCCAATGCGATACATTGACGCACCAAGACTTACAAAAAGCAAAGGTCGCTCTTATCGCATCAACCCAAACGCGCTACTTCAGCCGCGACATATCATTACTAAGAGAATCGAAGCCGATTGATAAAAAGAGCTCACTCTTAGTTCTAAACCCATTTCTAGACACGAAAGGTCTGCTTCGTGCCAATGGTCGGCTTGCTAATTCAAGCCTACTAAAGAACGCACACGTATAACGAACGTCATCCCATTGTTATACCAGAGAGATCGCAACTTGCCACTACACGCCGAACATCGCCTCATGCAACATATGGTACGCCAAGAGTTTTATATCCCAAGACTCAAGCCAAAAATAAAGAAGTGCATTTTCATGTGCAAGATCTGCACTATGCATAAGCAGAAGATGCGAACGCAGATTATGGCAGCACTTCCACCGGAACGCTGCAATTTCGCTCTGCCTTTCACCACCACAGGTGTCGATTTTGCTGGGCCTTTTCAGATAAAGGCGTCCATGCTAAGGTCTCCCACCCTCATGAAAGGCTATGTGGCTGTCTTTGTCTGTTTCACGACAAAAGCAGTACACCTTGAGCTATGTACTAATCTGACGACGGAGGCTTTTCTCGCGGCATTTGCTCGCTTCGTCGGACGACGTGGCTTTCCATCAAAAATCATGAGCGATAATGGCAAAACATTTATCGGAGCTCAAAGAGCCACAGAAAAACAGTTTGTGGATTTCATTAAACAAGTCTCACCTGACATTGTACAAAAGTACGCTCCCCAAGGTATCAATTGGCAATTTATCCCCCCAAGCGCTCCTCATATGGGTGGTTTATGGGAATCAGCTGTAAAAAGCTTCAAATCCCATTTCAAAAAGGTAGCTGGAAACTACAAATTTAATTATGAAGAATTCACGACATTATTAACTCGAATTGAAGCCGTTCTCAATTCACGGCCACTCACAGCACTCTCGCAAGATCCCTCAGATTTCACAGCCCTAACTCCAGGGCATTTTCTTAAAGGAGCACCCATTCTGGCCACACCTGAGCCAGGCGTGGAGTCGCTATCCTTATTAAATAGATGGGAAAGAATTAAAATTCTCCATCATGATTTCAGTCGCCGATGGAAAGAAGATTATATAAAGGACCTCCACAAAAGGTACCGATGGAAAACTCCAGAAAAAGCGCCTAAGCTTGGAGATTGTGTCCTAATTCATGACGATTGTCTCCCCCCTACCGAATGGCGGCTTGGCCGCATAGAGAAGCTCTACTACGGCTCCGACGGTCATATTCGCGTAGTCGATCTCCGTACGCAAACCGGAACGCTAACCAGACCGCTCGTTAAACTATGCTTTTTGCCAACCGCCGATAAAATCGAAACGTAAACCGTAAACAAACGATAAAACTCTCNAANAATAAATCTACAAATAATATATTAAAAATAACAACCAAAAAAAAAATGGTCGATCCACATGACGACCCACTCATGCCACATCACGTGGCACCAATGCCCATATGACTATATGCAACCAAATTCTAATTGAAATAACACTCTTCCAAACAGATCAATATGGATGCAGACATGCCAGCAGCCCCGACACCAACCACTCGTTCGGCTGTCAATGTGCCACGCCAAGCGGCTGCCCCAGTTGCAGCGCCCCGAACAACCACCCCAACGGCGCCTCGGAGTGGAACGGTAGCATTGCCGTCAACACCCGCGCAGCAGCAGACCCACAACGCCGTAGATGTCCCCTATGTCGTCGACCCCACCGGCTGCCACAATGCGTCATCTTTAAGGGATTGCCACCTCAACAACGCCAGTTGGTCGTGCAGGCGCACGGCCATTGCCTCAATTGTCTGGCGCCTACCCACACTACCCAGGAGTGTACGTCGGGCAATCTGTGCCAAATTTGTATGCGGCCGCATCACAC

>Btry_5345#LINE/LOA

GGTCGGCAAAAATTCCTCTTAGCTGTATCCTCCGCGCGGGTGTCGACTGGGCAATGGGTGTCGCTGCTTACAATGTCATGATCTCAAGCTGGCTCCTGTCAGAGTCATTACATTGCAGGCAGGCGGCTCCTAGCGAAGGCTGACGTATTTTCCCAATCATCCCCTTATGTTGGACTGCTTTATATGCATCCTCAGAAGTAGGTGAATGGGTAATACAACGGCCATTGCTAATGAGGTACCTGGCGGTAGGTATAAAACGCTAGGGGTAGAGACCCCCGAATCTAAAACTCAANATGGAGGACAACAAAGGAACGAAGCGGACTGGGATATCAGTCCAAACGTCGGTGGGAGACGAGGCTGCTACCACCGGCGGGCACAGCGATCGTATGAGCTGTGTCGCCAGCGGCCACACCTCCACTGCAACGGGAGCAGGTTGCGGTGGTTGTAGTACTACTGCCAAACCAGCTTTACAGCGCACGGGTGCCACAGTCCATGAAGACTCGGACCAAGAAAGCGCTGTAAGCATAAACGCGGAAAGGGAAGAAGAACTTCTTCGCTCTCCTGTGCAGGCGACCGAGGGTTCCAACTCTGGCAACAAGAGAGGGCCCCGGAAGAAGCCAAGCAAGGAGGGTTGAAGGCAAAAGCGAGATACAAGGCGGCGGTCGGAATATGCAACCGCCTCGAAGGCAAGTCCAGCCTGAAGGAAGAAGAGGTGAAACGGCTGGCTTGGGCTAGAGAGGAGGTGAAGAACGGCCGGGCACACTTCGCCACTCGTAATTGGTGTAATGCCTCGGATCCAGCGTATGCTAATCGCATTGAGGAGCATATCGCTGGAAAGAGGCAGCGTTCGACGGAGAGCGAACCAAAGGCGCCCTCGAAAAAGAGGAAGTGCAAAGACGTAGCCGGGAAGCTAGACGTCAAGCACGCGGACGACAAACCGACAACGTCCAAGGCAGCGGCGGCAGCCAATGAGATTGCGAAACGGCATTTGACCGTCGCGCTCATAGACCGCAGTAACCCGCTGGGACAAATGTCACAGGAGCGGTGGAAAATAGTGGAAATGAAGCTATTGGAAGCCCTCTTCTCGAGAATGGACGCCGAACCGAGCGCACCCATGCCGTCATTTGATGGAGCAGGATGGCTTAGCGGAGTCAAAATCTTGAAATGCAACGACGACCCGACGCTAACATGGGTAAGGGAAGCGGTCAGAACGCTTCCAAACCTGTGGGAAGGAGCGAAACTGGAGGTGGTGGACCGCAGCAGTATTCCATCGGTGCCCAAAGCGAAGGTAACCATACCTCGCGTGGTGGACCCGGAACATGCTCTGCGGCTGCTACAACGACAGAATCCGGACGTGCCAACTAGCGACTGGAGGGTGTTAAGCGTGGCGAAATCGATCAACGAAGATGGAGGCCAAAGCTACATCCTCCAGATCAATAAGGCGGCAGAGGATATTCTGTACGCGAGGTTCGGGAAGATGGCGTGGGGCGTGGGAAGCGTGTTCCTTCGCCTCAAGAAGCGCCACCCCGCGGACGGCAACAAAAACACGTTGGAGATGGGTGAAGTAGAAAAGGATCTCGGCATAGAAGAGATCATGGCTACTTCACTTGTCCTACAGGAGGTGGAGGTGGAGAAAGAACCCAGTCAGCAGTCTGATGGTCCAAAACCAAGAGCTGAGGGTACTCCAAGTGAACCTCCACAAAAGTAAGATCGCCTCTGCCGAGCTTCTTCTCAATTTAGAGAAGGGCGGCTACGACGTGGCTTTAGTCCAGGAACCATGGATTGCATCGGGTAACGTGGTCGCTGGGCTGAAGTCACAAAACTACACAACATACACCCCGAGCGTAATTAACAAGGTAAGATCAGCGATATTGGTGAAGAAAAGTCTATTTTCCCATATTGATTTCAGTCTTTCCACAGATGATCTGACGGTTGTGGCAGTGAAGGGTGCCAAGGATGAGTCTCTACTCCTTGCATCCTGCTATATGCCACACGATTGCGAGGCGCCAACGGCAGAGCTCCAAAGGCTGGCGGCTACATCCAGCAGAAGGAAGCAGGCGTTGGTGGTGGGCGCTGATGCTAACGCCCATCATACGGTTTGGGGCAGCCCCGACATCAATCAACGAGGTGAGTCCCTACTCAATTATATTTTACAAAGTAGTCTAGTGGTAGCTAATCGAGGTGAGGAACCTACCTACATAGGACCCACTTCCAGAAACGTTCTAGACATAACACTATACACAAGTAGAGATGCATTGGTAGAAAACTGGAGAGTGCTAAGCACACCGTCGTTCTCTGACCATAGGTACATCTACTTCTCCATTAGATCGGAAGCAAAGATCAGAAACGAGGTTAGGAGAAATCCCAGGAATACAAACTGGGATCTCTACAGGCAGATACTATTAGGCAAGAGACTTAGACCTTGCGTGTATAACTCACATGAAGAGCTTGAAAAGGGCGTGACTGTATTTACGAATGCCCTAAATACAGCTCTTCACTACGCATGCCCTACGCTGCGACTCAAGCACAAAACGAAGCCCCCTTGGTGGAACAAGGAGCTTGGGTCTTGCAGACGCAAAGTACGGGAATGCTTCAACTGGGCCAAGCTAGCAGAAAGCGAGGACTGTTGGAACGAATATAAAGATCTTCTAAGGGACTACAAGAAGTTGGTGCGCAGGACAAAGCGAGAATCCTGGAAAAACTTTTGTAGTGGATTTGAAAAGACCCACGAGGTGGCGAGACTTAGGAAACTATTATCCAAAAGCCCCGCCTCGCCCGGTCTAATTCGCAAAAGTAGCGGAGAGTGGACGGACAACTGCCAGGACTCATTGGAAGACCTAGTCAGTGTGCACTTTCCAGGCTGTAAGGACGCGGTAAGTCCTAGCTACGAAGGGAGTAGTGCGGTAAACGTTCCCGAACACATCTTTACGGAGAATAAAATAGAATGGGCAATCCGCTCATTCGACCCATACAAATCTCCCGGTACGGACGGAATCATTCCAGCAATGCTACAGAATGCGCTCTGTCTATCCGTGCCATGGCTAAAGGCGATTTTTAGGGGATGCTTGAAGTTCAGCTATGTGCCACACTCATGGAGAGAGGCTAGGGTAGTTTTCATACCGAAGGCAGGTAGAAACAACCCAACCTACTCAAAAGAGTTTAGGCCTATAAGCTTAACTTCCTTCCTGCTGAAAACGCTAGAGAAAATCTTGGACGCGCACATTAGGGCCGCAGCGCCTCCTAGAAGCTTGTCCAAGGCGCAGCACGCATATACGAAGGGCAGATCAGTAGAGACTGCACTCCATACACTGGTATTTAATATCGAAAGGGCTCTTGAATATAAGGAATACGCTCTTGGAGCATTCCTGGACATTTCTGGAGCGTTCAACAACGTCTCTACGGAATCTATTCTGAATAGTATCCAGTCAATAGGTGTTGATCCTGCTATACAACGCTGGATCAGAAGCCTTTTGACCTCTAGACGGATAAGAGCAGAATGGAACGACGCTAAAATGACCAAGGAAGTCTGTAGAGGTACTCCCCAAGGTGGAGTGCTATCTCCGCTTCTATGGACATTAGTGGTGAATAAACTGCTAAGGAACTTGGAAGGCAAAGCCCCTAAGATAGTGGCATATGCGGACGACATTGCCATCTTAATAACGGGTAGGTGTCTACAGACCATTAGCAGTATTATGACCACCACTCTCAATACAGTCCAGAATTGGGCATCGCAAGCGGGTCTGGGGTTGAACCCGGAGAAAACGGATCTGCTTGTGTTCACAAGAAAGCACAAAATTCCTCTATGGAGGTTCCCTTCGATAAATGGGACACAACTCTCTCTCAAGGACCGCACCAAGTACCTTGGGGTAGTCTTGGACAGCAAACTGCTGTGGAAGCACAACGTGGAAGAAAGAGTGAGAAAAGCTAGCAATGCTTTATATGCGTGTAGGCAGATGCTCGGCACAACCTGGGGGCTCTCTCCCTCTCTCATGCACTGGTGCTACACAGCTATTGTAAGACCAATTCTGCTCTACGGTGCAGTAGTATGGTGGACAGGCGTACGGAAATCCACCTACCGGAAGCCAATGGAAAGGGTTCAGAGGCTCGCTGCGCTGTGCATAACGGGAGCTTTAAGAACAACTCCAACGGCGGCTCTTGAACTGGTACTAAACCTGCCACCTATAGACCTCTTCGCTGAAAACTGCGCAGCAAAATCGGCGGGACGACTACTGGCTGCAGGAGAATTTACATATAGAACCTTCGGGCATAGCTCGGTGGGTAATGGCGGTTGGGCAAACACCGACTACATGATCCCACTTTTCAACTGGGAAAGAAGGTTCAAAGTAAACATAGAAAAAGATGGCTGGCGTAAAGGTATGTTAGCTACGCGCAACACTCTAAACATCTATACGGATGGCTCGAAAATGGACGATGGAGTTGGTGCGGGAATATACTGTCCAGAGTTAGGCATAAGGCAACCTTTTAAGTTGCCGGACCACTGCAGTATATTTCAAGCTGAGGTCTTTGCTATTGCGAAAGCCGCGGAGCTGGCCTCTAATGCACCTGCAGGCAATTCCAGAGTCAACATCTACGTAGACAGCCAAGCAGCAATCAAGGCAGTAACCTCGTATCGCATATCGGCCAGAAGTGTCTTGGGAAGCAGGGCAGCAGTGGAAAGTGTTGCCAGAAGCAAGCAACTTCACTTCTACTGGGTGCCAGGCCACAAAGGCATCGAGGGCAATGAAATAGTGGACGAGATTGCCAAGAATGGTGTACGGCTAACATCCGAAAACGTGATCAACATTGGGAAACCCATGCATTGTCTATACGACGATCTCGACAGAAGCATGGTAAAGAAAATCAAAACCCGATGGAACGAGCTACCTGGGTGCAAAACTGCAAAAGTCATGTGCAAAACGGTAGATCGGAAGTACACAAAATTCCTATTGGCACTCGATAGAAGAGACTGTAGGAACATGATGGGAATACTAACTGGTCACTGTCTGGTGGCGACACACGCCCGCAGAATGGGGCTGACAGATCGAGAAGACTGCAGGAAATGTCTAGAGCAAGGCACCAGGGAAACAATGGAGCATCTCTTGTGCACTTGTCCCGCATTGGCAAGACTACGCTGTAAGCATCTGGGGTCCCCACGGTATGATACACTGGAGGAGGTATCGATAGTGAGGCCGCAGAGTCTGTTAAAATTCGCGTCAAGCGCAGGCATCCTAAAGGATGACTACTCCTCTTGGACCTAGCAACTGAACTCCATCTGGTATCGCAAAGGACCAAAACTGGTCTATGCGTGGCTTATTGGCCTACCAGATTAACCTAACCTAACCTAT

>Btry_MarH#DNA/TcMarMariner

ATAACAGGTCAATTGAAAAGTCCCCGGCCTACCATAGTAAAACACATTTTTTTGGCAAAATTCGTTTTTTTTATTCAACATAGTTCCCTTCAAGGGTGATACACTGATTATAGCGACCCTCCAACTTTTCGATACCATTTTTGTAGTACGATTTGTCCTTTGCTTCAAAATAGGCCTCAGTTTCGGCGATCACCTCTTCATTCGACGAAAATTTCTTCCCAGCGAGCATTCTTTTGAGATCTGAGAACAGGAAATAGTCGCTGGGGGCCAGATCTGGAGAATACGGTGGATGCGGAAGCAATTCGAAGCCCAATTCATGGATTTTTGCCATCGTTTTCACTGACTTGTGACACGGTGCATTGTCTTGGTGAAACAGCACTTTCTTTTTCTTCAAATGCGGCCGTTTTTCGGCGATTTCGTCCTTCAAACGGTCCAATAACGCTATGTAATAGTCGCTGTTGATGGTCCTTCCTTTTTCAAGGTAGTCAATAAAAATTATTCCATGCGCATCCCAAAATACAGACGCCATAACCTTGCCAGCCGACTGTTGCGTTTTTCCACGCTTTGGAGCGGGTTCATCGTGTGCAGTCCACTCGGATGACTGTCGATTGGACTTCGGAGTGAAATGATGGAGCCATGTTTCATCCATTGTCACATATCGACGCAAAAACTCGGGTTTATTACGCTTGAACATCTCCAAACACTGCTCCGAATCATCAACTCGTCGTTGTTTTTGGTCAAAAGTGAGCTCGCGCGGCACCCACTTTGCACAGAGCTTTCTCATACCCAAATATTCGTGAATGATATGATGTACACGTTCAGTTGATATCTTTAGAGTGCCTGCTATCTCGAACAACTTCACTTTACGGTCATCCAAAATTATTTTGTGGACTTTTTTGATGTTTTCGTCGGTAACAACCTCTTTTGGGCGTCCACTGCGTTCACCGTCTTCGGTGCTCATTTCACCACGTCTAAACTTAGCATACCAATCCTTGATGGTTGATTTTCCTGGGGCAGTGTCCGGAAACTCGTCATCAAGCCAAGTTTTTGCTTCAACTGTATTTTTTCCCTTCAAAAAGCAATATTTTATCAACACGCGAAATTCCTTTTTATCCATTTTTTTCACAATAACAAAAGTTGCTTCACTCAAAATGATATAATTCACAAACTAATGATCCGACAGCTGTCAAATTTATACACGCGCCTTTTGAAGGTTAGGGCTAACTAAAAATCATATGGATTTAATTCTAGTAGCGCCATCTATGTGTCAGGCCGGGGACTTTTCAATTGACCTGTTAT

>Btry_MarI#DNA/TcMarMariner

ATACAGGGTTTGTCCGGAAAGTAATAGGACTGAGTCGATTTAAAAAAATTTATTGAACCAATCGTTACAATTCTTTAAAAACTTTCAAAATAGGCTCCTTCTGCGTCGATGCAGCGCTGCCAGCGCGATTTCCAAGCATTGAAGGCGTCACGGAAGGCATTCTCCGGAATAGCCTTGAGAGCCGAGGTGCATGCTGCTTGGATCCCCTCTGTCGTCTCAAAATGCTTGCCTTTCATCGGCCTTTTCAGGCAAGGAAACAAAAAAAGTCCGGGGGCCACATCTGGGCTGTAGGGCGGCTGCGGAAGCGTTGGGATGCCGGCCTTGGTTAGGTAGCTGTTCACAAGAAAGGCGGTGTGAGCCGGGGCGTTGTCGTGGTGCAACTTCCAATCGGCTGCGATGTCTTGTCGGACCCGATTGACCCTTCGTTTGAGTCTCTTGAGGACTTCCACGTAAAACTTGGCGTTGACGGTTTGTCCAGGAGGAACAAATTCATGGTGGACGATGCCTTTGATGTCAAAAAAGACAATGAGCATCGTTTTCACTTTGGATTTGCTCATTCTTCCCTTTTTTGGGCGAGGAGACGCCGGCGTGTGCCACTCGGAAGATTGCCTCTTTGTCTCGGGATCATACTCAAAGATCCATGACTCGTCACCTTTGATCATATCAAACGTCTCTGTCGCAGATTTACCGAGTTTCACACAGAATTTAATCGCGTACCTCTGCTCTAACGAACGCTGCATTTTCGGCTTGCACCACTCACAGAAACACGTCGCGCGAAAATGTTTGTCCTGACTCTCCAGGTGCTCGGAGACAACTGACCAGCCGCTCGTTCGTTAGCTAGGAACGCCCTCTACCGAATCCAGTCGGTGCGCGCACGCTCCGAAGTACAGTCGCGGCGGAAGAAAATCAGTCCTATTACTTTCCGGACAAACCCTGTATA

>Btry_5324#LINE/LOA

TAGGTAGGTAGGTAGGTAGGAGTGCAGCCCTATCGGGCTCAATTAGCACTTGATGTGCCATTTTGATACACTATTCGTAGAACCTCCTGTATCTGCTATTACGTTTCACCTTCTCTCTCAAACCATTTTGAGGTTTCGATGAAACTACTTATGTCCGATATGCTTTTAGTGGAAATCCATTCAAGGTTTGGTGCTTGATGGATTCCAAGTGTTTTGTGTCGGATCTGTGCTAGAGCTGGGCATTCGCAGAGAAAGTGGAAGATTGTTTCCTTGTTCCCTGCTACTCCGCAGCCACGGCAGATGTCGTTGTAGGGCATACCCATTTTGGACGCATGTTCCCCAAATGGCCAGTGCCCTGTTGTGGTAGCTGTTATTCTGTAAATACTTTTTCTTGACCTATTTAATAAGTCGTTGGTTCTTTTCCTGTCATATGTTGGCCATAATTGTTTAGTTATGACGCATTTTGTAATGTTTTTCCACCTGTTGTTTGCAATTTGTTGAAATTGTCTATTGATCTCTCCTTTGATCGATCCTAGCGGGCTTGGTATTAGCTCCGCCTCGGATTCGTTGAGGAAAGCTCCTGCCTTTGCCAACTCGTCTGCTTTTTCGTTACCGAAAATGTTCCTGTGGCCGGGAACCCAGATCAAGTTTAGTTGGAGCTTGTCTTTTATTGAGCTTAGTGCTCTCTTGCAGTTGTGAACTATACTGGAATTTGTCATGGGTGAAGACAATGCCAGGAGGGCCGCCTGGCTATCCGTAAATATAGTTGCTTTATGTATATTCCCGTGGTTTTCTAATAGAACTTCGCAGGCTTTTTCTATGCCTAGTACTTCTGCTTGGAAGATGCTAGCCGAGTTCGGTAGACGTATAGAGAGAGATATGCCTAGATCAGCGGAGAATACCCCCGTGCCTACTCCGCAGTCCATTTTGGACCCGTCGGTATAGACTGAGATGGTATCTTCCTCTACTATTGAACCTCTTCTCCATTCTCGTCTAGATGGTATCCTCACCTGGAAGTGTCTATTGAAATCCAGCTTTGGGAGAATGTAGTCTGATTTATTAATGGTGAGCTTGTTTAGAATTACACTATGTCCGTAGTTCTGCTGATTCCAACCTCCCAATTCTTTTATTCTTATCGCCGCAATAGCTGCTGTTTTGTGAATAAAAATGTCCATTGGTAGTTGATGCAGGATCACATTGAGCGCATCAGTCGGACATGACCTGATTGCACCCGTAACACCCGCACATGCTGCTCTTTGTATTCCATTTAATTTTCTAATGTTGTATTGTTTGTTTAGCGCTGGCCACCATACCAGAGCTCCATATGTAAGTATAGGTCTTATGACAGCCGTATACATCCACATGATTATACTTGGTTTAAGGCCCCAATTCTTGTTGACGATTTTCTTACAAGTATAGTAGGCCATGTATGCTTTGTTTATTCTTTTTTCTATATTTATTTTCCAGGACAGTTTGGTGTCAATCTCCACCCCCAAGTATTTAGCTGTGGGGGATAGAGTGAGTGTTGTACCGTTTAGTACCGGAAGTTGAAACTGAGGTATTTTGGTTCTGCTTGTGAATAGCATCAGTTCTGTTTTGTTCGGGTTAACTCCTAGACCATTGTTTTTAGCCCATAGGTTTAACCTACGAAGTGCTCTTTCCATAATCTCGCTGACTGTTGAAGGAAACATGCCTGATACCAACAACACTATATCGTCTGCATACGCTACTGCTTTCACTCCTCCACCGTTTAGTTGGAGTAGTATTTCGTTCAGCGCTGCAACCCATAGAAGCGGAGAGAGGACCCCCCCTTGAGGCGTCCCTCTACTCACATAGCTTGTTTGTGTTGCAGCGCCGTTTGAAGCTATAATCTTCCTATTCTCAAGCATCGATAGTATCCATCTGCACACAGTGTCGTCTATGCCACCATGTGCCAGAGAATTAATTATCGCATTTACTTCTATGTTGTTGAAGGCGCCCTCTATGTCTAGAAATGCAGCCATGGTGTATTGTTTGTGGTGTAGTGATTTTTCAATTACACTTATCACCTCGTGAAGGGCGGTTTCGGTTGATCGGCCCTTTATGTACGCATGTTGGGACTTCGATAACTTCTCCGCCATTATTGTTCTTACGTGTAGATCTATTAGCCTTTCTAGTACCTTCAGCATAAAGGAGGTAAGGCTTATAGGTCTAAAATCCTTGGCATTCTCGTGTGTTCTTCTGCCTGGTTTTGGAAGGAACACAACTTTACTTCTTTTCCAACTTCTTGGGATGTAAGATAGTTGAATGCTAGCTTTGTATATATTTTCAAGCCTTAGAACCATTGGTTCTACCAGTTTTTGCAGCATTATGGGCATAATCCCGTCAGGACCTGCCGCTTTAAATGGTGCAAAACTATTTATGGCATATTTGATTTTGCTTTTGTCTACTATTTGGCTTCGATAGTCAGCTGCGTTCAGCGGTGGTTCTGGTTGAACCCTCGTTGAAGGTGTTTGCTGACTCCCGGGGAAGTGCGTTGTGATTAGTACCTCCAGAGACTCTTTTGCCGAGGAGGTCCAATCACTTCCCTCCGCCCTAATGTAGGCAGTATTAATATGATCTTTGGATAGCATTTTACTCAGCCTAGCGGCTTCTCTGCAACTTTCTATCGATGTGCAGAAAGATCGCCATGAGTCGTTTTTAGCTTTCCTGACTGCTTTTTTGTATTCTTTCATAATGTCTTTATATGGCTGCCAGATTTTGGTTCTAAAACTCTCATTGAAAGCTTTCCTTAGTTGTGTTCTCAAATTCTTGAGTTCACTGTTCCACCAAGGAAGAGACTTTCTCTTTTTCAAAACTGTTAGCGGAGTAGATTTATTAAAAGTTGTGGTTAAGATTTTTTCCAACTTCTCTACTTCTGAATCTAATTCCTCAGGATTTCTGATACTCTTATTGCCTCCCTTTTCAAGGCATTTTGTTGCTATACTGGTAAATTTTCCCCATGCCGTTCTTCTAGGGTTCCGGTATGTGAGAGGCGCACTGTACTTCTCGCGGATGCTGAAGAGTATCCAGGAGTGATCCGACATGGAAGGCTCATCGGATACCCTCCAGTTATCCACAACGAGACTGTCTGTGTCTGTTGATAGCGTGAGGTCAAGCACTTCCTCCCACCCCGGAAACCTATCCGAGCTTGGGAAAATAAAAGTTGGCTTATCGCCCTTGTTGCAAATACTTAGATTACTATTCAAAATATACTGCAAGAGTGACTCACCTCTGGTGTTTATACTGGAGCTACCCCATACGGTGTGCCTTGCGTTTGCATCACACCCTATTAGGACATCTTCCTTCCTGTTCTCCTCTACTAGTCTGGTGAGCGGGGCCGGTGGTATGTCTTCGTCATGGGCCAAGTAGGCCGAGACCAAGAAGAAGGGCTTTTCCTTCTGTTCCACCTTTACCACTGTGATATCTGCTGTGCTGTAATTAGGACAAAGAAATGCATTTATACTTTTATTGATAAGAATGCATGCCCTAGGTTTACCTCTGTTCCGTGTGTAAAACAGGTTATAGTTGCTGCTGTTTAGCCCTTTAATGGTATCTTCATTGACCCAGGGCTCCTGTACTAGGCTGATGTCGATGCCCCCCTCGTTCACGAGGGTTGTCAGATTCGCTGTAGCACTCTTCGAGTGCTGCAGGTTTATCTGTACGCATCTTATGTTATTGGGCATCTCGGGTTTCTTCGATGCCCACATTCCTTAGCAACTGGCTGGCGTCGTCGACCTCTTCCGTGTCGTCTTCGTCAGCCGCTTTGTCGCCTTGGAAGATTTTTAGTCTGGCCTTGCGGATTCCGAAGCTGATTTTGTAGTCAGTCTTCTTCAGAGCCTCAATGGACTCATCACAAATGGCCACTAGTATTGGCTTGCTTGCTTTATTTGGTTTTTCCTCCTTGATTAGCTGCCACTCATCTATACCAGGTATAGATTTGTTCTGCAGCTTAATGCACCTCAGGAGTTTTTCGCCTGGCTCCTCTAGAGGGGGTAACCAAATGCGAGCACGAGGTCTCTTCGGAATGTCCCTGGCGGGTATAAGCCTCAATTGGAGGCCTACAAAGGCGTTGCTAATTTTAGCAACACTACCCGTCAGGAAATCTAGCGAGGCCTGGTCCGCGCACTTGATGACCCTGTAGCCCCTGAGGGTCTCAGAGGAGTCAAACTCCGGGTGAGGACCCGCAGGATTGTCGAGTACATAGCTTAGCACCATACTCGACAATCTGACGTCGATCTCCACCCATCTCTCCTGTATTGTGCTCGGAGAGGAGGAATTTCCATCTATGATGGCCACCTGCAGGCTATCTCTGGCTACATCGCAGAAATGCCTTGCCGTTGTTGTGCTGCTGTGTTCACCTTCACTCCGCCTCGGTTTTTTGGGCTGAGTTCCGGGTACTTCTGTGATGGAGCGATTTCTCTTCTGAGAAGTGCTCTCTTGTTTGCTCTCTTCTTGAGGTTGTGATTGCTTCCTTTTCAGGTAGCCTTCATATTCCTCTACGATTGATTTGAGGCGCTTTGTTTCTGCCTCATCAACTGGGGTACCGGCTGCCTGGTCTTTGGCTATCTTCCCTAGTATGAAGACTGCCCTCTGGTACCGGTTTTTCCTCAGCTGATTCTGGGATTTCTTGCGCTTCTTTTTGTTTTCTCCTTTAGCCGCCAGTGCACTTTGGTTGGTGCTCATGGCAGCCTTGGAGGTGGACGCTTCCTCCCTTAAATCTGCGGTTTTCACTGCTGTATCTACCGGTATACTTTGGTTGGTATGTCCGGTAGTACTCTTACTCGTCTCCTGGCTGGAGGCAAGTAGTTCGTCTTCTTCGGATTCCGTAATAGGATTCCGATAGTTCATGTCCTTAGTCGTTGCTGTTGTCGTCAATTTGGTTGTTGTTTGTTGTGTTATTGTTTTCGTTCATTTTTGGTCCCACGAGTAATCCGGAAAGGGTTGGTCACTCGTCCGCAGAGCCGGTATGCGGAGAGAAGGCTTTTATACCTCCGACCTCGCCCGGGCATCGGAGGGGACCGTTCGCGATCAGCTATTTATTACCCCCCGCTGACCATTCAGCCCTCGGCACGGGTACCTCGACACCTTGGCTTAGGGTGGTGTTGGTTCGGGTATCCTCATACTTCCACAATAGGAGATGGGCGTAAAACCTAGGGTTTATTCATCCATATCACTATTGTGGGAATTATGAAGTGTCCCTCTTAGTTCGTAAGATTAGAGTGCGTTTCCTTTGGGATGCACACTTTACTCTTACTTAAGCCCCTTCGCCACGACAAGGCGACCAACTTCAAAGAGG

>Btry_1326#DNA/TcMar-Tc1

TATACAAGGTGGCGCAAAAATAACCTTCCGATGTTTTTTGGCTGTAATTTTTTTAAAAAATGAAAAACTTTGATTCTGCTTGTGGATTATTTTTATTTGGTCTTTTAAATTTTTTTACATCAAGTCGAGAATATGATATCATGTAAATGGCCGCCGCCACAGTTGATTGTCATTTGAGCCCTTTTTATGGCATTTTCCATCACTTTGGCCAGAACTTCCGGCGATAGGTCCTCACATTCTTGACGGATATTGTCTTTAAGAGCTGCAAGAGTCTGAGGCTTGTTGACATAAACCCGCGACTTCAAAAAGCCCCACAAAAAGAAGTCTGGAGCGGTCAAATCAGGCGATCTTGCTGGCCAGTGCAAATCGCCAAAACGGGAGATTAGGCGCCCGGGAAATGCATCCTTCAGCATATCGGTTGTGGCACGTGCAGTGTGTGCCGTTGCACCGTCCTGTTGGAACCACATGTTTTCCAATCCCAATTCATCAAGTTGCGGCAAAAAGAACTCGTTGATCATTGCTCTGTAGCGCTCACCACTCACAGTAACCGTTTGGCCCGCGACGTCTTCGAAGAAAAAAGGTCCGATGACTCCTCCAGCGAAAACAGCACACCATACAGTGACTTTGAGCGGGTGTAATGGCTCTTCGTGGGTTACACGCGGATTTTCAGTGCCCCAGAAGCGTAAATTTTGCTTATTTACGTACCCGCTAAGATGGAAATGGGCCTCATCACTCATGATTATTTTTGATGAAAAATCATCTTCCTCTTGGTGGTGATTAAGGATGGCTTGAGCGTATGTTAGACGCGATTGGCGGTCAGCAGCTAACAGCTGATGCACCGTCTGGACTTTGTACGGAAACATCTTCAAATCTTGTACCAAAATTCGCTGTAAACACCGTCGACTGATACCCATTTGCGTGGCACGTCGTCTGGTCGATGTCGACGGCGCTTCCATGACATCCTCGGCAACAGCAGCAATATTCTCTGCAGAACGGCTACTCCGGGGTCTGCCACGCCTGGCAGCATCTCGTGTTGTGCCAGTCTCTTCGAGGCGAGCGGCTAAACGTCGCAGTGTTTCACCAGTAGGCGTCGGACGGCCAGGAAATCTGCGACGAAATTCACGTTGTGCCAAAGTCACCGACCGATTATTGGTCAAATAAATAGTCACCAATAACCCGCGTTCTGGAGCAGTGTAGCGTACCATTGTTTATTTACGTGTATTTCGCATGTGTTTACTACACAAATGTCAAAACAGAACTGACATTAGAGGTCAATTGCAAAATTTATAGCATCTTCTGATAGGAGGATTACTTTTGCGCCACCCTGTA

>Btry_MarJ#DNA/TcMar-Mariner

ATATACTACTGTGATCAAATTGAAAGGTGAATTTTGTCCATTTCATCTCCTTCAAAGTAATCCCCTCCCGCTGCAATACACTTATGCCAACGAATTTTCCAGTCATCATAGCACTTGGAAAAATCCTCCGTCGTGATGGCCATCAGAGCCTTCTTCGATTCAGCTTTTATATCCTCAATTGAGTCAAAACGGTGTCCTCGGAGTGGTCATGTGAATTTGCTGAATAGCCAGAAGTTACACGAAGGTAAATCAGGCGAATGCGGTGGTTGCGGCACGATATTAGTTGAAAATGTGGCGAAATGATCACGAATAACCAATGCAGTATGAGATGGTGCATTATCGCACTTCTTTGTTCAATAAATTCAGACATAGTAAAAATCGAAGAATTCACTTTTAGAGCCTCACAAAACGACGCGTATCTCAAATACTAATGAATATTTTGACGTGAAATTTAGCATAGATGTCACTAACAGTACTACCAACTTACAAAAAAAATTTACCGATTCGAAAAACACGCGAAGTATAAATTAAAAATTCACCTTTCAATTTGATCACAGTAGTATATA

>Btry_3105_LTIR#Unknown

CGGCCTTGAAATTTCGGAAATTCGCCTAAAATCGTGAAATTGTCTACCTAGCGCCTGAAATACGCATCTCATGGCAAAATGTATAAAACAAAAGTTATTTGTCACGTCATTTGCTACCGAAATGGTATATGTGATCATGGCCGTAGGACGAACCGTTCCCGAGATACGAGCGAAAAGGCGGCGCGCCACAGCGCAAGGTGAAAATTGTTGCAGCTCTCAGCTAACCTGTATTGGTCACCCAGAACCCACCGCGTGGAGGTGGCTGCTCTTCGGGTTCCTCCCAAGCCCAACCCAACCAACCAACCATATGTCAGGCTTGTTTTAGTCTGAATCTGTGTCAAATTTATTGATAAAATCAGATTTTGTACTAAACTTTGGCACTTGTTGCCTAGATTTCAGTGTAGAGCGTATAAAACGATCACGAGATTGGGGGCCAATAACTTTAGAAGATGCCTCTGTCACCAGTTTGACGGTTCTCTCGACTGCCACGGTGTGAGAGGGGAATTCACTAAATTTCCATACCTCTGCAGTGTCTCCCGACAGCCCTTTTATGAGTTCTTCGTTAGAAACTGAACAAAGAACTGGTGGAACAGTCAAGGTAATAGTCTTCCAGTTAATCATATCGTAATAATTATTAGCTTTGAAATTCAGCTTTGGCACTTTATTACATCTAACCCTTCCATTTACAGTGTCAGACTCTGCTTCTCTGGCTGCCAGAAGACGGTCAAGGGCCAACTTTCTGACTTCTATCCGTTCGTCAGTCATCATACTAAGGAGAATGTTTTCTGGAAGAGCAAAGAAAGCATTCTGTTGAATGGATGAATCGACAACCTTGCGCAGTTTAGCAGGTAAGTATCTCGACCTTTGAATTGCAGCATAGAGATGGCGCGAGCCATCTTTGATTGAACTGTTGAATCTTATTGAGAACCACAAAGGTGAGTAAACTGTAAGTATGTACTTGACCAAAATCTTTATTTCCTTTGTTGGTTTGTCAGTAGAAATGTATAATCTCAGAATTCTATTTGCACAGGTGAGCCAGCGAGATTTGCACATGTTACCTGGATGCATCGACGCTAAATCTGAGGAACATTGACCGGAAATAACAGCTTCTGATATTTTATAGAGATAAGCTTGGTCTGTGCTAAGTTGGGTCGGATTAATAACCTCAGAAGGTAATTGGCAGGATGGAAAACTTTCAAATTTGACAACAGGCAACTTCTCACAATCAGGGAGCAGTTTACCTATGTCGCCAGAGAATGTATTTGGACTCTTTGAGACACCATCTATGTGTTCGAAAAGAGCACGAAATGGAAGTTCGTTGAAATGTAGAAGACAAACAACCCACTGTAATGGCCTACCTATTCTTTCTTCTAGTTTCCGAATGATTCCACCTTTCCAACCTGTGTTCGTGTTGGTGCCATCAGCACCGACAACTTCTAGATGTTCCACATCAACTGAATTGTCTTGTAAATGTTGCCATATAGCTTGCGTCTCATCCTCAGCTGACCCGGAAGGAGAGGTGACGTGGCCAAAGTAATGACAACCAGGTTCCGCTATAAGAGAAATATGTTCCTCTTTGACAGTGTCGCGGTAGTACTTTTCACCTTCGCTGACTTGTGTAAGTGTATTGTCTTTGCGGCCGTCAAAATACAGCCCATATACAGAGTCAATACTTTCGGTGTTTTGGAGCTTAACTCCAACACTTTTTTTTGCCCGACTAATCTTGCATTTGTCAGTGACAAAGCTAGCCTGATCCTCTGTTATCAAACCTGCTTTTTTGGCATCCAGAAAAGCAGATGAAACAATCAAGGCCGCTGCTCTATCACTTACTCCAAATCTTTGGGCAGCTAAAGCAGTGTGTTCTAATACTAGTGTGTTTTGTTTGGTTTTAGAGGATGGAAGAGAAAATTCATCATCAGCATCGTTTTTGGAAGAATCCATGTGCGACGCACCTACATTGTTGTCGTCTGTATGAGAGTCTGGCTGATCTGAATGGTCACGTGTTCTAGCTGATACTTTTCTGGTAAGTGTTGATGTTGAATGACGTTTTGAAAGCTTTTCTTTACGTTCATTTTTCTTTGTAATCTTTTTTGTTTCAGGTAGATCAACACTTCCAATGCGACCAATTCTTCTGGTTCTCTGGTCCAAGAGAAATGGTTGTTCATTGATAGGAACTTTCCTTTCCTTTGGACAAGTGCAAGAAGAAAAATAAATGCATTTACAAGCAGCGATGTCAAATAATTTTCCGGCAGAAGAGACAAAGTCGTCTCTTTTAGAGCTCAAACCTATTGGGTTCCTTAAAAACATTTGTTTAAGAGTCAGAAATTTCTTATGGTATGTGGTGAGCATTTGTACAACTCTGGTGTGTGAAACTATCGGAATAGATGACTTTTTGAAAGTATTTTCAACCTTTTTTGCAACTATTTCTGTAACCTCTTTACTCCTAGGTTCATAGTTGTCGCCTCGGAGTCGCCCTATACGAAATCTTTCAAACTGATAACACAAAAGAACATCCTGGTAAGTAGGTAACTGGGACTCAGAGAGATTGTACGGATATATGCCAAACACAGGACATTTCTGTCTAAATTTAAATTTAGATACAGACATTTTGAGTTCTGTGTTCTGTTGAGAGAATATAAGTGATAGCACTCGGCGAAATCAACGACAAGAGTGAAGGAACTCGATGAAAAAATATTTATGTGGAGACCAGTCCGAACGTGTCGTATGGCGCAGGGAAATGAATGTAAGTGATAGAACTCGGCGAAATCACCGACAAGAGTGAAGGAACTCGATGAAAAAATATTTGTGTGGAGACCTATCCGAACGTGTCCTTTGGCGTAGGTGAATGAATGTGAGTGATAGCACTCGGCGAAATCAACGTCAAGAAGTGTGGCCGCAAGCCGATTTTCACCTTGCGCTGTGGCGCGCCGCATTTTCGCTCGTATCTCGGGAACGGTTCGTCCTACGGCCATGATCACATATACCATTTCGGTAGCAAATGACGTGACAAATAACTTTTGTTTTATACATTTTGCCATGAGATGCGTATTTCAGGCGCTAGGTAGACAATTTCACGATTTTAGGCGAATTTCCGAAATTTCAAGGCCG

>Btry_1501_hAT#DNA/hAT

AAATAAACATTTACTTGAAACATGAAGATTATTATATTAAATATAGTAGTCTTTATACAGTTTAATATCATGGTGGAAAAATTTCAAATCGACCTTCTAATATCAAATCCTACTAAATTGAAAAAAACACATTATAAATCGTAGACGTGCGGGTGACATACTTTATTGCAATGTATAATACTGTAGTCAGTTCGTGTATTGAATAGAAAAATTTCACAAAACAAAACGATGTGGGCCAAGTTTTGAGAATCTTTCGAGAACTTCTTCTGCAGTGACTTCAATGTCATGATGCGTGTGCAGCAACGCCAGGCCGATCAATCTATCTTGAAGCATGTTCGTCCTCAGCCACGTTTTCATGCGGCGAAGTGTCGAAAAAGAGCGTTCAGAGCTCGCATTCGTCACAGGAAGTGTGCAGAATATGCGAAGAAAACTATGAATTATGGGGTATAGGTCTACATCGCAGTTCTTCAACGTTTCCAATGCAGTAGTCGGTAACTTGTTGTCTTTTGACTTTTGCTTCTGCCCCCAATGGCACTGCCAGTGATCAAGTTCACCTTTGAGCTTCAAACGTCGCACGACGTTGTCATTATCCAAAAGGCCTTTGAATCTATCTATCAAGCAGTCAATAAGATTTTCCATTTCTTTGGTTTTCAAAGCACATACTTTTTCTGGAAGCAGCAAACTCAGTTGAAATCCATCCAATACTTCTCTAGAAAAGCGCGTCTTCAAATCTTCGCCGATTGTATCCAACAGAGGAATGTACACTGAGACCCTGTAGTATTCTTCGCAAGTTCTCACGCAGAAATTTTCGCGTTTTGTTTGTCGGCCGCAGATTCTTGGTTTTTCTTCGTCAACTTTCAGTTCTGCCGCCATTTTTTTCGTGTCTGAATAAATGGATCGAAAATGTTCCTCAGCTTTTTGTCTTCGATTTCGGAACGTTACAAGCAGCGTATCTATCATATTTGATGCCTTTGCCAGATCGATCGATTCTTTCTGAAGAATCACGCTGAGTGAATGAGTTAGAGATAGAATATCACATAGGCAAAAAATCCCAATTATAAATTGAAATTTGCACACGGCTGCGATGAGTATCGTTGCTTTTCCCGCTGTTTCCTTATTTTTCCAGTTACTAATTTTTCTATGTTGGAAAACTTGGAGGATAAACCGCGGAGAATGTATCAATCGAACATATCACAGATGGAGCTGAATGCAGCATCTATTTCAAGTTCTCTATCAAGCCGCGTCGCTTCAAATTTAGCACTTTCAGTTACGGATCTGATTGGATTTATTAACAATTTAATCTGCATACGTTGCTACACTTTGTAACTTCAGTGGTATCTTAGCATTATTTATTATATTCAAAAATGGAGACAGTCTTTAATTTTGTTTCTGAAATAAAATGGCAAATTTCTGGATTATATTTCTGTTACTTTATATTTTTTCAGAGTAGTAAAATTACTAATAAGCCATTTAGCCCTTGGGGGTAAATTTCCCCCATTTC

>Btry_MarK#DNA/TcMar-Mariner

TTAGGTCTACAACTTTGCTTCCGCCGTTTTCCAATAGATGTCTCTAGGGTCAAGCACTGGTCGATTAAATCGTTTTATATCGATCTTGGACATTTGTGTCAACACTAACCCAACAAAATATTTGTAGAGATCTGTTTGCATCCNAAACTTATTCTCAACTGAAAATGTCACTTTTTGAGCCGAATTCTCGACATTTGCGGGAAATTTTGCTTTTCTTTTTTAATTCCAAGAAAAGTGCGGCTGAGGCTCATCGAATGCTTTCGGATACGTACGGTGAGGCTGTCCTAAGTGAAAGAACATGTCGCGAATGGTTTCAACGTTTTAAGAATGGTGATTATGAAGTCGAAGACCGGCATGGTGGTGGAAGGGAGAAGATTTTCGAAGATGCTGAATTGGAAGCATTACTCGACCAAGATGCGTTTCAAACCCAAGAGGAATTGGCCGAATCGTTGCAAGTGACACAGCAAGCCGTATCAAAACGCCTCAAAGCCATGGGGATGATTCAGAAACAAGGAAACTGGGTTCCTTACGACTTGAAACCAAGAGATGTCGAACGGCGCTTCTTTGCATGTGAACAGCTGCTTCAAAGGCACAACCGAAAGGGATTTTTACATCGCATTGTAACTGGCGGCGAAAAATGGGTCCACTACGATAATCCTAAGCGAAGAAAGTCATGGGGAAAGCCTGGACATGCCTCCACGTCGACGGCAAAACCGAACATTCACGGCGCCAAGGTCATGCTCTGCATTTGGTGGGACCAGCTGGGGGTGATATATTATGAGCTGCTAAAGCCAAGTGAAACCATCACAGGAGATCGATACCGAACGCAATTGATGCGTTTGAGCCGAGCACTAAAAGAAAAACGGCCACAGTACGAGGAAAGACACGATAAAGTCATTCTCCAGCATGACAATGCTCGGCCTCACGTCGCAAAGGTGGTCAAAAAATATTTGGAGACGCTGAAATGGGAGATCTTACCCCACCCGCCGTATTCTCCAGACGTTGCTCCATCTGACTACCACTTGTTCCGATCGATGGCACACGGTCTAGCTAACGAGCACTTCAGTTCTTATGAAGAAGTCAAAAATTGGATTGATACTTGGATCGACTCGAAAGATGAGGAGTTCTTTCGTCACGGAATACGCATGCTGCCAGAAAGATGGTCAAAAGTAGTAGCTAGCGACGGCCAATATTTTGAATAACATTTTTGTAACCGTTTTTATACAATAAAGCCTTAAATTTACAAAAAAAAACGGCGGAAGCAAAGTTGTAGACCTAAT

>Btry_possibleHoana1#DNA

TACCAGAGAGCTGCAACGAGTATGATAAAATCAGAACAAACAGTCGTGCCAAAAACACAATCAAATCAATTCAGTTCAGCATAGACAACATTGTTAATCAATTCGAGTAGCCGCCAGCGTCAACTGACCTGATGCAACACGAACAATCTGTTCTTCGGCAATCACGATCGTGTAAACGGTATGGCTGGCTTCGGTTGCAATCGTATCATGTCAGTTCATAGCGTTGCGACGATTGTTTGAATTGAAGTGAAACTTTGGGTAAACTATAAGTAAATCTACGAGTAAATCTATGAGTACAGCAAACAACAACAATTTTCTGCTGGACTGATTTGAATCATGTGTGGCAAAAAATGTATCAGCTTTGAAAATGTGCGCATGTTACAAATTTTCGAAGCGTAAACAATGGAAATTCCATCGAGTACGTACATACATATATACATACATTTGTTGTATGTATGTACTCGATGGAATTTCCATTATACATATAACATATGTATATACATACATACATGTATATACAAACATATGTATTTGAAATAGAACAGAAGTGCACTAGTAAATCAGTGTTTAGGGGATGATATACAAACCGAACGCTGTCGATCATTTCAATCATTGAAGCATGAGTTTGCATCACTTCGGGTATTTTCTGCTGATACGAACGTTCATAAACAAATCAGGTATAAGCTGATCGCTAGTGATTCAAAGTTGTTCTCTATCGCTGATTTGAAGACAAACAGTTCGCTTCGTGTACATGAACTGAACTGACAGAATCAGACAGAGTAACGGATCAGTACTTAAGTATAAACAAAAATTTATTACTCGTTGCAGTTCTCTGGTA

>Btry_Baggins#LINE/LOA

AGATAGATAGATAGATAGATTATTATGAGGTAATGCACCGCGACCTAGGGTCTATTGTGCCCTCTCCTATATCACATGTCTTCCCAGAGTCCCAGCAGCCTGAATAGTTCCAGGAGTTTGCCGGGCGCTACTGAGGTGATGTGATCCCTGCTGATATAGATGGAATCCAGGGCCTTAAGCCTTTTTCCACAGATTGCTGTGCAATCCAGGATTAGGTGTGCTGGTGTTTCCTGTTCCAGGTCGCAGAACCGGCAGTTAGCACAGGAGACTATGCCCATGTTGGACAAGTGCTTCCTGAGCCTGCAGTGCCCTGTGTAGATTGCGACGAGGAGACGGAATTTGTCACGGGGGAGGTTAATTAGTTCCTTGAACCTGGAGAGGTTGTACCCTCCTAGAAGCAGCTTGGCGTGGCGCATACCTGCTGCCTGCTGCCAATGCCGTTCTCTCTCCACTCTCTCCTCCGTGCGGAGCAGCTCCTTAAGAGTGTGGGATCCTACCGCTATGCATGGTTCTGGTCCCATCATCTTGGACGCTGCCGCAGAGCGGGCCAGTTCGTCGGCCTTCTCGTTTCCTTCTATTCCTTTATGCCCTGGCACCCAAATTAGGTGCACCCGGTTGCACAAGGACAGGCGGTTTAGCCTTTCTATGCATTCCTCTACTAAAAGCGATTTGATCTCATATGCTGAGATCGCTTTTAGTGCCGCTTGACTATCGCTGAGAATAGCTATACGCTGGTTGCGATAGTTGCGGCGGAGGTTGATTTCTGCGCACTGACTTATAGCAAAAACTTCTGCTTGAAAGATGCTCGGAAAACGTCCCATTGGTATGGATAGCTTGGTACGCGGTCCCGCAATGCCTGCTCCAATGCCTTCCGGTGTTTTCGAGCCGTCAGTGTACCACTGGATGGTACTGTCTTCCAGCAGTCTTTCCAGCGTGGAATCGCTCCACTCCGTCTTGCTGCCGAGAGTAACTCTAAAGTTCTTTGTGAAGTTAATCCTCTTCGTAACGCCGTCCCTTGGAAGAAGGGCCAGCGGTGTGCTTTCCGCTAGTGCCTTCATCTGTTGAGACGACATTATCTTCCCTTTGCCGAAACCCTCTGCTGACATTAGCAGCATGGTGTGTTTTGCTGCTTGTTTTATCACCTGATGCAGCGGTGTGAGCTCCAGTATGACTTCCAGTGCCACCGTGGGGCATGTGCGCATTGCCCCCGAAGCACAGACACAGGCAAGTCTTTGCAGCTTTGACAGTCTCCGGATCACCGAAGACTGCGCTGCTTTGGTGGCCCAGGCAACCGCTCCATAGGTGACAATAGGCCTTACTATCATGGTATACAGCCATCTAATGATACCTGGCTTGCATCCCCATGATCTGCCGGCCAGGCGTCTGCATATCATAAGTGCTCTAGTTGCTTTGGACAGCGTTAAGTCCAAATGCCTACTCCACCGTAGTGATGAGTCTAAAGTGAGGCCAAGGAATTTGACCTCCTTCGACATTTCCACCTCTGTGCCTCCAATTGTTAGGGACCTCAGGCCCGGAAGAGATCTCCGCCTAGTGAATGGGATCACGGTAGTTTTTGCTGGGTTGATGTTTAGCCCTACCGTGTTGCACCATCCCTTCGCCAGGTTTAAGCCCCTTTGAACAATGTCACAGAGAGTGTTCTCAAATCTACCTCTCGCCATTATGACAATGTCGTCCGCGTACCCTTGACAGCGGATTCCATTGCTGGTGAGCAACTCGAGGAGTTCGTCTACTACCAGGCTCCATAGCAGAGGGGATAGTACTCCACCCTGTGGGCAGCCCCTTGTGGTGCCAAGACGAATCTTGCTTTCCCCCACCGTTGTTTCCGCTACCCTAGTACGCAGAAGGGCTTCTATCCATCTGCATATTGGAGTGGTTACGTTCCTTCTCTCAAGTGCCTTGATCACGCTAGTGTGAGACGCATTATCAAAGGCACCCTCAATGTCTAGGAAGGCGCAGATCGCAACCTCGCCGTTATCCAGCGAATCCTGCAGCTCAGACGTGAGTTGGTACAGAGCAGTGTTTGTGGATCTACCCGCTCTGTACGCGTGTTGCCTGGCATGTAGGGGTGCGATCTTCAGCGCTTCCGATCTAATGTGGTTATCTATTACTTTCTCCATCCCCTTTAGCATGAAGGATGTGAGACTTATAGGCCGGAATGATTTGGCTAACGAATAGTCTCTCCTCCCTACCTTGGGGATGAAGATTACTTTTGCAATTCTCCATGGCTCTGGGATATACGACATCGCCAGGCTGTCTCTCATCAGCCCTAGCAGGTGTGGCAGGAGAATATCCATACCTTGCTGCAAAAGGGCCGGGAAGACGCCGTCCACTCCCGGTGACTTATAGCTCGAGAAGGAGGCCAGTGCCCATCTGACTGAGTCTGCAGTGAACAGATTCCTTGCAGTCAGCCAGTCTGAGCGATCCGGCCTGCGTGTGACCGATGCTGGAGTTAGGTCAGCTTGAACCGTCTCCGGGAAGTGCGCTTGTAGGAGTGTTTCTGCCCTCTCCTCCGCGCTAGTCGTATAGGTCCCGTCCTGTCTCTTTAGGGATAATACTGTGTCCGTCTTACCTCTCGACAACGCCTTGTGCAGTCGAGCTGCCTCCGGGGTCGAGGAGACGCTGTCACAGAATTTCCTGAAACTGTTTGTCTTGGCAAGCCTAATCTCCTTGTTGTAGGTTGTTAGGTGCCGTTTGTATTCCTCCCAGCTGCCTGTGCGCTTGGCTTTATTGAACAGCCTTCGCACCTTATGTCTGAGAGCTGAGAGTTTACTAGACCACCAGGGGCAGATTTGTTTACTGGTGGTCGCTTTCAGTGGACAGGCACAATGATAGGCGTCCATAATGGACTGGTTTATGGCACTCAGTCTGCTCTCCAGTCCAACTGTGGTGGATCTACCATCTGCCTGCCTCACTCTGCTTATGTTTTTGCTTAGGGTCTCTTTGAAAGTGCCCCAATTTGTATTTCTAGGGATACGTGTCGGAGGTCGGTCCTCGACTTCCACCCTCAGAGCAAAACGAATGATCCTGTGATCTGACAGTGAGGGCTCTTTTGATACCCGCCACTGCGAGATCAACCCGATCATGTTGTCATTGCTTAGGGTAATGTCCAGGACCTCCCTGCGAACACTAGTTACGAATGTGGGCTCACAGCCCACATTCTCTATGTTAATATTATTACTAATTATAAATTCAAGCAGTGACTCACCCCTCATGTTGCAGTTCGTGCTACCCCATTCCGTGTGATGAGCATTGGCATCGCATCCCAGGGTCAGGGGAAGTTTGTTGATCCTGCAATACTCCACCAGATTAAGTATCGATGGGGGGGGTGCTGTGGCTGTCTCTCCCGGAAAGTATGCGGATGCAAGGACGAAGTCCGAACCTTCCTTGTCCTTCACCTGTACCGCCACCAGATCTTGCGTTAGAAACTCTGAAATACAGAAAAAGTCTATATCGGCTCTAACGACTACACAAGAACGGGGTCTCTCGCTAGAGAGATCCCAGATTACCTTGCTTCTTCTCGTGTTGAGGCCTCTTACCTCTCCTTTGAACACCCAAGGCTCTTGGATTAAGAGGATGCCCAGGTTATCCGAGATGAACCTCTTCACGATGACAGCCGAGGCTGCCGATGCGTGATGTAGGTTCACCTGGGCAACTTCTATGCCGGCGCTGGAGCTTATAATATGGGTTCGATGAGAGACAGGTCCTCGTCCCCGATCTCTTCATCAACCTGCATTTCCAGCCCTTCCAGGAGCTCTTGGGTGGATGGCAGCGTGCCTTCTTGCTCGTTGGGGAGCTCGTTGCTCCTGCTTTCATCCGCGGCGGTGGTCACAGCCTGCCCAGCCGAGATGGGGTCGTTGGGGGACTCGTGCCGCTCTCCCGCAACCACAGCGGCAGTCGCCTCCTGCCCTGCTGAGCTGGCTGCGGATGAAGCCGGAGCTGCGGTCATCCCCGTCGCAGGTTCTTTGTTCGCCGTTGGCGTTGCCTTGGGCCTCCATGGCCTCATCACTACCCTGCCGAAGCGGAAGTTCAGCTTGAATCCCTTCTGCCTTATGTATTTGTAGGATTCATCATCTACTGTTATGTTGAGGTTCCACCCTGAACCCACAACACTGCTCGATACGACCTTCCACGCCGTGGTTCTTAAGCCCACGTTTTGGTTTTTCACTAAACCAAGCGCGAAGTCGTAATTTTCGTCCGCACTTCTAGGGAAGAATACCGCCATACTGTGCAGTTGTGGTATATCCTCGCCTCTCTTAACACACAGGGCCGGGCCATTCCAACCCACTAGCCTTGGCGTGGTCTCCGTTAGCCAGGTGGCCGACTTCTCATCCTGGCAGTCTACCAGCAGCATACCGCCCTTGAAGAAGATGCCATTAAAGGCGCCGGTGTACTCGTCGCCCACGAACAAAGCTTTCACTAAGCAGTTCTGGAGAGACGTCTGCTGTTCCGACCCTAGGGCCTCCGCCGGGTAGTTGCGGGGCAACACTGCCATCCGGATGCTCCTCACGGCCTCCGAGTACTTCCGGCTTACGGCTCTCGTCGGCTGCTGGTTGGGGTTGGAGCTTTTTCCCCTGGCCTCCTGTGGTTTGTCCTCTCGGATTCTCTTGGACCTAGGTGGCTCCTGAGGAGTTATTTGGCCGCTTTTCCTCTTCTCCGTTCGATTGGGTGCCTCTTGGGGTGCCGGCCTAACTTCGCTTCGTCCCTCTGACAAGGCTGCTTTGGCTCGGCGCCGCCTCTGATTCCTGCGTCTTGTTGCAGACGAACCGTTGGCACCTGCGCGTTGTGCATTTTTAGGAGAGGGGTTGCTCCCCCTGCTCCTACTCAGAGCCCGCTTTTCAGCTGTCTCCGGTGTCATGCCGTCCTGAAGGAATCTGAGGTACCACTTGAGAGTGGCCCCACTCATTCCCGCTCTTCGTGGGTCGTCGTGACCTCCTGGGCGTCCTGGCTCCGGGGTACATGGGATGTTCCCTCGTTGCCGTCTCCTTTTACGTTCGCCCCTTGATTCCACTTTCGTGAAACCACCCTCCTCTCCGCAGTCGATCCTGCTCGAGGGGGTTGTGGGGGCGGCGTTCGCGCGGTTCGCAGTGGTCGCGATGGACGCACTACGTTGAGTGCCGCTGCAAGAGGGCATGTCGTCGTCGTCACGCGCGTGCTCCTCAAAGAGTGCTCGTTCTTCCGGCGACAGAGCATCCAGGAAGCTAAACTTTCGTCTAGCCCCTGGGCCACTCTTGCTTGCGGTACTTGTGCAGCCGTGCACTGTTGTTGTTGATGCTGCTGTTTCGGTTGTTGTCGTTGTTGTTGTGTTTTGTTTGTTGTTGTTTTTGTTGTTCATCTTGTTCGTACGACCGTTTGGCCCGTGTTGTGGGCCTCCCGGTCTATTCTTTAGAAGGGGAATCTGAAAGGTCCGCCGCGCCAGAGCCCCTTGACGCGGTAAGGCCACCGTTACTTCCCAAGGCGGCCCGGTGTTGGGAAGGCTCCGTTCGAATACAGCCGAATTTACCTCCTGGCTGCAAATCATCCAATGGGCACGGGAGTCGCATAACACCCTGGATTAGGAGGTGGTAGCTCTTGGTCGCCGCACACATGCAGCTTCTGCCAACCCCCAGGAGAGATGCTATGCAGCATCACCCCTGATGGTTGGGAGATGCCGTACTGTGTTGCAGTTTAAGCCCCTGCACCGCGACAAGGTGCCTACCATTCGCAGAGG

>Btry_5979#LINE/R1

CATTCGTTAGTTGACTGTCGACGCGGATAGACGTGCGTGCGCATCGTTGCGATATTCAAAGTGTTTTTTCGGTGATTTTTTGCGATTCTCTTTTGTGCGGTTATTTGTTTACGCGATTTAGTTGGCACCGTTATTGCCTGGCGCGTGTGCGTAGCGGTTCGGTGGAATTGTTTGTGCGATCGCGAGTAGTGAGGGTCGGTGACCGCGAGTGTTTTTGTGCGGGTACACATTTTTGTGCAATTTTGTGCATTTTTGTGTGCCAAATCGTCGTGAGTAGCGGTAAGTAATTTAATTATTACTACTGTATATACGGACAACGTGGTCATCCACGTTGTTGTTATTGGCGGAAGCTTCGCACGGTTCCAGTGTAGCTCCGTGTTGTTGGCATTCTTGCTGACTGGACTGCCTGCCAGTAGTTCTGTGCTGAGCATCTGGCGGTTAGTTGGCTCAGTGTCAGGAGGATCTGGACAGGATTGCCCAAAGGGTGCAAGCGTCTGTCCAGCGTTGGAGATTGCGACTCCTGTGAGGACCAACGGTCGTAACAGCGCTACCGTTTAGCCCACGGTGCGATTCGGCTCAGCGCCACATCGGTCGACTCCGGACCGTTACGGGGACTTTGTCCGGCCGGCGGCCAATTGAGTGGCCTTAATCCGACCACTGGTTGGGGTTGTGGGCTGTGCCACCGCGAGCACGCTATAATCGACCGCTTGGGTGGTCGCTGTTACTGAGCGGGCCCTTGCGTGAGTCGCACTTTTCAGCGCGTTCAGACGCACGCACCTGGAGAGCATCTGCTCCAGAATCTTTCTGTCCGTGAGCCTTCTACGCTAGTGGTACGCCAGCGGATCAGTTCGACTGAAGGGTGAGTTGGGAAGCGAGATGCCTCCTGGACGGAAGAGGAGGACGAAGGCCCTCGCTGGAAGCGGAGAATCCGCCGCTTCCACAGTCGTGGATGACTCCACGTCGGGTGGCGAGGGGGAAGCGACGGGAACGACGGTAAGGTCTCCACCGCGGAGTAAGGCGCGTTTGGGGTCGCCGCGTAATGAGGGCGGAAGCGAGGGAGGGAGCGGGAGTTGTGGCGGGAGTGCCAACGTGGCCTCCGCTGAGAAGGTGGTTACGCGTGCTGGGTCGCCGCGTAACGTAGGGGGTGTCGAGGGAGGGAGCGGGTGTTGTGGTGGGAGTGCCATCGTGGCCACCACCGAGAAAGCGGTTGCGCGTACGGGGTCGTCGCGTAACGAGGGAGGTAGCGGTAGTGGGAGCGTGGCCGCCGCTGTGAAAGCTCATCCGTCTAGTACGGTGGGCAAAGGAGTGGGCGTCGCGAAGGAGGGCGCTGCGAAAGTTGTCACTCGTGTCGCGAGTGGCAATAAAGAGGGAACGCGGGCTCTTCCAAGGGGAGCTGTTGTTGTTGGGGGTGGGGCAGTCAGCCATGTTGCGGCTGCCAAACGAATAACGGGGGAGCTAAACGAGCTGGTATTCGAGGCCAAAAATTTCGATGCAGGGACCGCGAAGGGGCTGATGGAGCTTGCCTCAAAGTATGAGGCGCTCCTGATGACGGTCATTACCGAGAATGCCCATCTTCGCGGTCAGGTAGATGCCCTTAGGGGAAGTTGTGGGGGACACTCCTCGACGCCGAGCAGGTCAATGCCGGCTCCGTCGGCACCGATGCCTGCGCCTCCAGCACCTGTGCTGGATGCGATCACACCGGTGACGCCGAAGCCTGTGGAGACCTGGTCGGTCGTGGTAAGGAGTAAGGGGCCTGCAACTTCCTCTAAGGAAGTTATAAAGAAGGTGGTCAAGGAGGTGGGTCCCTCACTTGGTGTGAGGGTCCACGAAGTTAGACCAATTAAGGGTGGTGGGGCGGTTATTCGCACTCCCTCTGTTCTTGAGAGGGAGAGAGTTGCGAATAACAAGAAATTCGAGGAGGTGGGGTTGGACGTCTCTGTCAACCGGAAGTTGGGTCGTCGGGTTGTGGTCCAGGGGGTTCATACGGAGATCCCCCATGAGGAATTCATGGAGGATCTACTCCGGCTGAACCTCAAGGACTTCAGCCCGGCGTCCCAGAGGACTGACGTGAGGATGGTCAGTCGCCCCTGGAAGGTGGCCGCTGACGGTAGCACCAATGTCGTCCTGGAGGGTACGGACAAGTTGATGTCCGCCCTCTTGGAGACAGGTCGGTGCTACATAAAGTGGTTTTCCTTCCGGGTGCGACCGGATAGCCCCGTCGCTGGCTGCTTCCGGTGTATGGGTTTTGACCATAGAGTGGCTGAGTGTAGGGCCAAAGCAGATGTCTGTCGGAGGTGCGGTCAAGAAGGCCACAAAGCTGCCAGTTGCGTCAATGCACCACATTGTCGCAACTGTGAGTTTAAGGGTAGACCAGCTGGGCATCTTATGATGTCAGCTGTCTGCCCTATATATTGTGGCATTGTTGAGCGCGCCCTCGCCAGACACTGATGGGTGGGATTATCCAGCTCAACTGTCAGGGCTCTTATGCCGTAATGTGTGAGTTGGGGGGTTGCATGGTTGAGGGTGGGTGCAGTATTGCTCTACTCCAGGAGCCCTACGCCACCAATGGTGTGGTTCGGGGTCTTCCTGGGGGTTTTAAGGTCTTCACTGACCTTAGAGCTAACGCTGCAATAGTTGTGAATAATCCACGCTACGATTGCGTAGTTGTGGATTCTTCACAACTGGGTATATGTGTAGCTATAGAAGGGGAGTTCGGTAGGATGATTGTCGCTAGTTTGTATTGTAAATATAGCGAGCCCCTAGAGCCCTACCTGGGCTACATGGATAAGCTGCTACTACTTGCGAGTAGTAGTCCATTTATCCTAGGGCTGGATGCGAATGCCTCGTCCTCGATGTGGTTTAGTAAGGTATCCCGGCATTCGTCTGGATACCAAAGCCACAGTCGAGGCGAGGCATTAAGCGAATGGGTGGTGGCTAAAAGCCTCCACATTTTGAATGAGCCGAGTGAATGGTATACGTTTGATGGGCCTGGGGGCGTAAGTGACATTGACGTGACGCTTATGAATGAGGCAGCAAGTAGGGCTTTTAGCGTTAGATGGGAGGTTAAGGGAGGGTGGGGATTGAGTGATCATAATTTGATTCAAATTATGGTTACCCCTCGATCCCCTCCCTCGCCTTATGAGAGTCCATTGCGGCGATGGCGTACCACTGGTACTGACTGGAACCAATATGGACTCTCGGTTAGGGAAGCGGTATCTTGTATACCGCTTAGTGAGTTTGAGGAGTTGGGGGTGGACGAGCAAATTGCTCGTCTCTATGAGGTAGTTTGGGGGGTAAATGATAGGGTTTTTCGTAGGTATGACTGTTCTAAGGTTAGTAAAATTAAATGGTGGACGCGTGAGCTAACCTTGAAGAGGAGGACTGTCAGGCGATTGAGGCGAAAGTTTCAACGCGCCCGACGGTCCAATTCTGATAGGCTGTCCCAGCTTAGGTATGAATTTAGTTGTGCGGATAGGGAATATAAGGAAATGTTAGTTAAAATTAAAGAGGAAGAGTGGAGGAGTTTTGTTAGGGAAAATAGGAACGACCCCTGGGGTCAGGTCTATAGGATCTGCCGGGGTCGTAGGAGGGAGGATATCACCTCTCTTCGCGTCGGTGACACTCTGTTATCGTCGTGGAGAGAGTGTGCGGGGGTTCTGTTAGGTGCGTTCTTTCCCAGGTCGGAGGTGCAGGTACCTCAAGCACAAGAGGTGCCTGTCCCTCCATTAGATGATGGGGAGTTGGGGTACGCCTTTGGCCTGGTTAGGTCTAAACGGTCCCCAGGTTTTGATGGTTTGAACGGAGAGATGTGTAAAAGCTTATGGAAGTTCATTCCGGAATACCTGGAGGCCATTTATGATAAGTGCGTCTGGGAGGGATATTTTCCACGCGAGTGGAAAAGTGCTAGGGTTGTTCCTCTCCTGAAGTCCCCTGATAAGATCAGGAGCGATCCTCGATCTTATCGGGGCATCAGTCTCCTTCCAGTACTTGGAAAAGTGCTGGAAAGAGTCATGGTGGAACGGCTTCAGGAGCTAACGCGGGGTATGTGGTCGGATAGGCAGTTTGGGTTCAGGAAAGGACGCAGTATAGAGGATGCGTGGTTTTATGTTCAGAATGTTGTTAGGGAGAATGTCAACAAATATGTCCTTGGCATATTTGTTGACTTCAAGGGGGCGTTTGATTACCTAAGCTGGGATCGAGTGGTGCAACGGCTGGAGGAGCTTGGCTGTCCGGAAATTACTCTTTGGCGGAGTTATTTTTCGGACAGAAAGGCCTCTCTTGTCGGCATGAATGGGAGTGTGGAGATCGGAGTTGTTCGTGGCTGCCCGCAGGGTTCCATCTGTGGTCCATATATTTGGAACCTTATGATGGACACTCTGCTTGGGCAGCTCGAGCCACTCTGTAAGTGTTGTGCGTATGCGGACGACCTTCTTCTTATGGTTGAAGGTCGCTCGAGGTCTGAGTTAGAACGGGCGGGAGGAGATCTCTTAGAGATCGTTAATTCTTGGGGTTTAGGTGTGGGGGTTGACATATCGATGGAAAAAACGGTGGCAATGCTGCTGAAAGGTAGATTGTCACCGGTTCGTCCACCGATTGTCCGTGTGAATGGGGTCAGCATCAGATATGTGACGCAAGTCAAATATCTGGGACTGACCATGAGTGAAAGGATGTGTTTTACTCCACACTTGGCGAATGTGAAGGAGCGACTGCAGGCAACTGTAGGCAAAATACGACGGATTTTGAGGAGCGATTGGGGTCTCGGACGTCGTGCTGTCCGCACCATATATCGTGGCTTGTTTGTGGCCTGTGCCACTTATGGAGCCCCTGTATGGTGGGAGACAGCCACGACTGTCTCGAGGTACTGTATGATGCTTGCATGTTTGCCTGTATGTCGCACCGTCTCAACGGATGCGATGCAGGTTTTGTTAGGTGCACCCCCTCTTGACCTGATTGTCATACAGCGTGCTGTTGCATTCAGGTTAAGAAGGGGCTTGAGTGTGTCATTGCTGCGGAATGACTGGATTTCCGACGATGATGTGGAGAGGGAGGGATATCTAGGGAGCAAGAGGCTNCTAGATGATAGGGTTAGGTGTAGGTGGCAAGAACGTTGGGACAATAGTCCTAACGGCCGAGTGACGTACGAGTACATTCGGGACGTTGGGTTCGTTGAGGATAACCCAGACTTCAGATTTTGTCTGAGTCTGGGTTTTCTGCTTACGGGGCATGGGCCTCTGAATGCATTTTTGCATCAGAGGCACCTATCGGATACGTCGGGGTGTTTTTGTGGGGCGGGGTTAGAAAATTGGTCGCATTTAATTGCGGAGTGCCCGATGTACTCGGACATTAGGGATCTGGGTGGTATGGGGATTAGCTGGACTGATGGACGATTAGATGTTAGTGGTGTGATCTCCACTAGTCGGAATACCGAACAGTTAGGGTCGTTTGCGCGTGAATTATTTAAGCGGCGAAGGCGGTTAGCTGGAAATTAGGGTTAGTTTCTGTATGATTGGTGTGTGTAAGTGATGTGTGTATGGGGTCCAACCCCTGGCCACCAGTCCAGAGCCGTATGGAAGCTCAACTGGTAGTAGTTTCGGCTACGAGGTCTGACCGGAGACTTAATTCTGGTACCACGGGGAGCAGGAGCCCTTGGAGTTCGCTCCAACCTGCTCATGCGGACTGGCCCCTCGGGGAGTATCGTGGTGGTTGTGGTTAAAACCCAAATGCGGGAAGAACTGACTTTGTCAGTTGAATGTGGAGTTGCATTGCAACCGGGTGCCGGACCCAAAGCACGGCAGAGGTTTTAGATGGGCCTCGAACCCTACCAAGGTGGTTAGTGTGTCCATTCCACACTGCCAATTGGTACTGAAAATGCTTAGCATTCTCAGGGCGCTGATCAACGCATTGATTTGATCCGCTGTGCTTTTGAGCGCCGTGGAGTAAGGCTGTCGTCAGCAGGTACCCACGTTAAACACACCGGACGTTG

>Btry_MarL#TcMar-Mariner

AATATACAAGGTCTGTCGCAAAAGAAACAGAACTTTTTAAATATAACTGTTTCTGGTGGCGCCACCTATTGGTGGGTATATGAAATAAAAAGTTTGATCTCTTGTTGACATTTCGTAAAAATTTTAAGACAATTGGATAACTACAATCGATGTTATCGATCAAAAAGTGACAGCAGCTTTTGGTCATCGGTCGTAAAATGCAAAGAGCAAATATTAAATTTTGTTTTAAACTTGGGAAAACGTTTACTGAAACATTTCAAATGATGAAAAAAGTTTATGGTGATCAGTGCCTATCCCGTAGTAATGTGCATGAGTGGTTTAAGCGATTCCAAGAAGGTCGTGAGGACCTCTGTGACGATCAGAAGTCGGTCGTCTTCAGAAGTCAAAAATAAAGACAATGCTGATTTGTTTTTACGATTCCGAGGGTATTGTACACCGAGAGTTCGTCCCACCTGGCCAAACGATTAATGCTGTGTTTTACCTTGGTGTTATGAAGCGTCTTTTGTCACGCATTCGTCGTGCTCGACCACAATACCGTGAGGCAGGGTCCTGGCGCCTGTTGCACGATAATGCGCCGTGTCATCGGTCGACGCTTGTCACTGATTTTTTGACAAAAAACTCCATATTAACCATTAATCACTCACCCTACTCACCTGATCTGGCACCCTGTGATTTTTACCTATTTGGAAAACTTCATTTGCCCATGAAAGGACACTGGTTTCAGGACATTTCAGCTATCCAAAAGGCGACGACCGATATTCTCAAGAGCATTCCGAAAAATGACCTTAAACACTCATTTGAAATGCTAATTGACCGGGCTAAACGCTGTATCGAAGCACAAGGAAACTACTTTGAATAAAAAAATATAACTTTTGAAAAATATTAATTTTTTGTTGTTTTTTTAACAGTCCTGTTTCTTTTGCGACAGACCTTGTAT

>Btry_4956#LINE/R1

TTTTTTTTTTATAGTAGGGGGAAGCATCGAAAGCCGGAGTGCGGAACTTTAATCCGCTAAACCTAACCTACTCCCAACTCAAGACTCTCCCACGGAACCACCGATTAAGTATTACTTCGTGGGAGGGACAGGACATTTAAGTCTCCTCTATTGCGCGGACTGACGGACGCCTAATCTGTTGAAAAAGTCTCAGTTTTGTCATTATGAATGCTGACGCTTCGCTGACAGCTTTCCATTTAACAGCTTTCCATTTATCAGTCGACTGACACATGAGTGTCGTTAAATTATCGACCGTAAAACTACCACCGAGTGTGGTTTTTAGTTTTTCCCTTGTATTTGAAAATCGAGGGCAATAAAATAATACATGTTCAGAGTCTTCTATATACTCTGTACACGTCGGACAGTTCGGACTAATGTCATTGTGGAATCTGAAAAGGTAGCTTCTAAAGCACCCATGTCCACTAAGTATTTGGGTCAGATGGAAATCTAGGTCCCCATGTCGTCTGTCTATCCACGAGTGGATGTCCGGTATAAGTCGATGGGTCCAGCGCCCTTTGGATGAGGAATTCCACCGCTCCTGCCACATTTTAGTGCTCTTGTTTCTCTCCTCTGTCCTTGCCGATACTGTTTTAGGCGCTGATAGCTGATATAAACGCTCGAGTTCATCTCCCTGGATGTCAATGGGCGGCATGCTGGCTAATACTTCAGCAGCGTCGGTTGATATAGTCCGGAAAGCACTTATTACTCGAATTGCAGAAAGCCTATGCACTGCAATTATTTGTTTAGCATACGCTTTTATATCCATTGCCTGTATCCATACTGGTGCTGCATACAGTATTACGGAACTCATTGCTTTCGCTATTAAGAATCGCCGGCTGGAACGCACACAGCCTCTATTGGTCATCATTCTCGATAGAGCATTATAAATTTTGTTTGCTTTACCGGAAGAATACTCCAGGTGTTCTTTAAATTTTAATTTGGAATCCAATATTACTCCCAGATACTTCAGTTGCGGCTGCGAAGTAATCTCGCACTCCCCTATGGTGAGCTCTATTCGTTCTTCTATCTTCCTAGTGCTTATGAGTAAAACTTCTGTTTTTTCCTCCGCCAGTTTCAGACTCACAGAAGAGAACCATTGGCGTAGACCATTTAAGCACTCATTACATTTATTTCTCAGGTCGGCTAATTGTTTGGCCACTGCTACCACCATGAGATCGTCCGCATAAGCTACTAGTTTCACGTCTGTTGGTTGCTGTCTTCTTAGCACCCCGTCATACATGAGGTTCCATAATAGTGGGCCTAACACTGACCCTTGGGGTACTCCACTCGAGATAGAGTAGCTCTTGGTGCCTTCGTCCGTATCAAAAATCAGCCTTCTGTTTTTAAAATAACTCATTATAATGTTTATGAGGTATTCAGGGGCCCGTATTTCATCCAGGGCTTTGATTATGTGTGCCCATTTTGCCGAGTTGAACGCATTCTTGACATCCAGGGTAATCAGCGCGCAGTACTTTTTTGTGCCACCTTTCCATCTTTTGCCACTTACTGCACATTTCGCAGTATCGACGACTTCTCGTAGTGCGTCAATAGTGGATCTCTTTTTTATAAAGCCGTATTGTCTCTCTGATAATCCGCCGGCTTTCTGGATTGCTAACTCCAAGCGGTTTCTTATTATACTTTCGTACACTTTACCCATTGTGTCGAGCATACACAGAGGTCGGTACGATGAAGGTTCTTCAGGTGGTTTCTTTGGTTTTGGGAGCAGAACCAAACGCTGTACTTTCCACGAGTCGGGGAATACCTCTTCCTTTATACAAGCGTTGTACATTTTAGCGAATAGTTGAGGTTTTGAGCTTATAGCTTCCTTCAGGGCTCTGTTTGGTATGCCATCCAATCCAGGCGCTTTGGTGTTTTTGATTTTCTTTGCTATGGCCAATAGTTCGTCTTCCGTGACTAACGGGGGTGACTCTGCAGCTTCGTTTCGTCGCTTGGCATATGAAATCCGGTCGTGTTTCGGGAATAATGTTTCGACGACTTTTCCCATAAAAGAGGCATCTTTAGGTTGCTGCTGCTTATTTTTAAATCTCGACATACAGATTTTGTAAGCAGTTCCCCAAGGGTCAATGTTTGCTTCCTCACAGAGCTTCTCAAAACATGATTTTTTGCTCCGGGTAATTGCCGACTTCAGGAGCTTCTTGTGGCTTTTAAATTCTTCTCTAAGGCGATTTTCGTTCGTTTCACCCCGGTTACGCTGTAGACGCCGTCTAGCTGAGTGACAAAGCTTTCTCAGCGTGGAAATTTCCTCATTCCACCAATATACAGGCCGCCGCTTGTTGTGATGTGATGTTCTACGCATTGTTGCATCGCATGCTGCGACCAGTTCCTTTCGCACTGTCGATACCAAGTGGGCGGCGTCGTCACCTGATGCTTTTGATGCACTCCAGACTAGCTCAAACAGGTCTGGGTCGAACTCCTGTTGCTTCCAGCTTCGCTTTCGGGAAGTGTTTCTGCTAAGAAGTTCCGGCTCTCGGGAGTACGAGATTTGTGCTATAATTGCCATATGATCGCTGTTTGTGTATATGTTTGACACCGCCCACTTAATGTGCCTACTAAGCGAATCATTTGCAAACATAATGTCAATTATGGATCCTTTATTCCCTTTTTGGTACGTGTTTTGTGTTCCGGTGTTCATTATTGTTAAGTGCGTTTGACTCAGATATTCTTGAATTAGCCGCCCGCGATGGTTTGTACATCTGCTGCCCCATGCAGTTGACCAAGCATTAAAATCACCCGCTATTATAATAGGTGTTTTGCCTCTGGTTTCTAAAGACAGCCTTAAGAGGAAATCTTCGAATTCTGTGATTGCTGCGCTGGGACGAGCATAGCAACTTACAAAATATATCCCCTGTATATTCGCCCATGTGAAGCAGTTATGAGCTTCTCTGGAGCAGGTCTTAAAAGGTTGACCTTTGCAGGACCATATTGCTGCCTTGCCAGATTTGTCTGCAATCCATGTGCTTTCCGGACGCTGGGAGTATTGTTCACTTAGTATGGCGACATCAATGTTCTCTTCTATGATTGTCTGATCTAGCAAATCCTGTGCTGCTGCGCAATGGTTTAGGTTTAACTGTAGTATCCTCATTTACTTAGAGACTTCATCATCTCGAGGTACTTTGGGCAAGTCGTGCTCAATACTGAGTGCTCCCCTTTGCAGAGCATGCAGCTCGGAACGTTTGTGCATACCTTTGCTAAATGTCCCGCGGTTCCACAACGTGTGCATTGGGAAGATCTGTCTGTCAAACTGCAGCATTTACGCGCCATGTGACCCAGGTGGAAACACTTATAACATCTGGGAATAGGCGAGTATTCTCGAAGGCGACAGATCGACCACCCAATCTTTATTTTTCCCAATTTGAGTGCGGCCTTAGCATCTTGAGCACGCATGCATACAAGTGCTATTTGTGTGCCACTACGCGTTTTTCGCAGACTTTTGACACTCGATATCTCCAGATTTTGGATTCCACACTCCCTTTTCAGTGCTGCGCAGATTTCTTCGGGAGTTGTTATTTCGTCCAGGTCTTTGCACATAATCGTGACGTTGTGGGTTTTGGGCTGTATCATAGCCATTTCTCCAACCACTCCCTCCAGGGCGCTTTGGAAAGATTCGGCTTTCGTGTCTGCTCGATTTTTTAGTTCAATAAGTAAATCTCCTTTTAGCGTTTTACGAATATGGGTTACATTTGAACCCAGTACTTCAAGCCCGCTGTCACTTTTAATTTTTCGCAGAATATCTGCATACGACGCACCTTCCTTCTTTGTTATAATGATGGCATCCGGTTTTGTTCTTATTTTCTTTTCCATCGGTTTCCTCTTTTTAACCACGTCTACCCATCTTTCACTTGTGTTTAGACTAGTCGTATTTTCCTCTTGGGTTAATTTTGCTGCTCCACTGTACAACAATTGCGGCTTTTCGTTTACATTTTTTGGCTTTTTTGTGGGTGGTAAAAAGCCTGATGCCTCTCGCATTCTTTTTGGGGTATTATCATCTCTACCAATAGTGGGTACTTGCGATGGTTTCCCTATCAAAACTCTTCTCGCTTGATTTCCCAATTTTTCCGTTTTGGCATGTAATATCACTATGCTGCTTACTAATTCGCGCATAGCTTGGTTAATATGCCTTTGGCCGGCCATCATCATTTCTAGTTCTTTAATTTTTTTACTCAGGCTGCAAAAGATGCTAACCGTTTCATTGTTTTGCGATTCCTCGGGTTTAGCGCTTTTGCTTACATGATCGGCAGTATCACATTGCTTATTTGGTCCAGCAGCGGCGGACTCCTCATTTTTACCATTAGGCGGCGTTCTCATTATTTTGGAAATCCTTCGGAAAGGATTATCCGGTGTTCGCCCAATACTATTTTCAGAGGCCATATCCTCTGCGAAAGAATAGTTTGGGAACACCCGTCGCTGTGTATGTTCAGTCCCTAGCCTTACTGCTTAACTAAAAACAAGACGGTTGGCAATCCCAGAACAGAACAGAAAAAGGGAACAGCTGATACTTGTGTTTTCGATGGTTGGCAATGCGCAAACCAAAAGGAAAACGAAAACACGAGGAGGGGAACAGCTGATCGATCAAAATAACAACGGCAGAAAAAGAAAAATACAATTAACTGCACGCTGTTATAAGGGGAAAAATTAAATTTATTTTGCAAATTGGCGCGTAAAAAACAAAAGGTATGTGCGTATGATTATATATAAGTATAAATCAAAGTGTACTTATCTAATCCCGTAGGTCTGGTGTCCACTTAGGTGGTCAATCCTGCCGGAACGTCCCACACAAAAACCCACTTCCAATATAAAAAAAAACTAGAAATTTACTAAGAAATTTCGGGAACACAATAAAAACACGTCTACTTCACACGTATGCAGTCGTATCGTCGCTCTCCACCACACATATGTAC

>Btry_MarM#DNA/TcMar-Mariner

TATACGAGGGCTGCTATATATATTCTGGCCTAGGGCAACACTAAGTGTTGCCAGGTGCAATCTGACATTTCCATTGGAAAGTTTGACATTTTTTAGCATAACATCACTCAGAACGTTTTGTCATTTAATCGTGAATTGTTTTATTTACAGGGAATTAAAAAATTCATCTCGGCCAAAAAATGGAATTAACTCGTGAACATTTTCGTGCGATCATTTTTCACAACTTTCGACGTGGATTATCACGACAAGAGTGCATCGATGAACTAAAATCTTTGTATGGCTATGAAGCACCATCCTATAGCACTGTGAAAAACTGGTACAACGAATTCAATCGTGGCCGACGCTCGCTCAAAGACGAATTCCGTGAAGGTCGTCCAAAAACAGCCGTTGTGCCAGAAAACATCGATGCCGTACGTGAACTGATAATGCAAGACCGTCATGTAACATACCTTCAGATAGAGGCATGCCTATGCATTTCTCCCACCAGCATACATTCGATATTGCATGAACACCTGGCCGTAAAAAAGGTTTGTTCTCGTTGGATCCCGCACAATTTGACAATCGCTCAAAAAAAGGCTCGTGTGGATTGGTGTAAAGAAATGCTGAAAAAATACGATCGCGGTGCTTCAAAAGACGTTTATAAGATCGTCACAGGTGACGAATCATGGATCTATGCGTATGAGCCCGAAACAAAACAGCAATCGACAAAAAAAAATAAAAAAAAAACATTTTCGTTGATAAATATGCCTATTTACATTATTAGGCCAGAAATATATATAGCAACCTACGT

>Btry_5785#LINE/LOA

ACCTCTGCGAATGGTAGGCACCTTGCCGCGGTGCAGGGGCTTAGGCGCAAGGCATACTCGGCATCCCCCAACCGGTGTGGGTGGTGCTGACAGGCACTGCCCAGGCAGGATGTCGGGTGCCGCATGCGTGCGCCAACCAAGAGCTACCACCTCCTAATCCAGGGTGTTATGCGACTCCCGTGCCCATTGGATGATTTGCAGCCAGGAGGTAAATTCGGCTGTATTCTAACGGAGCCTCCCCAACACCGGGCCGAATTGGGGAGTAACTGTGGCCTTACCGCGTCAAGGGGCTCTGGCGTGGCGGACCTCCTAGTTCCCCATAAAAATAATAGACCCGACAGCTCACCTTGTTGAGCCAAGGGTCGTAAGAACAAGATGAGCAAAAAACAACAACAACAACCACAACAAACAACCCAACTACAAGGCAACGACCCAGGACAACGACCACGGACAATGAGAAGGAAACCGGTGCAGCAGCGAGCAAGGGGGGTCAAGGAGCTAAGCGGAAGTTTAGCTTCCTTGACGCTCTCTCGCCAGAGGAGCGGGCGCTGTTTGAGGAGCATGCCAGGGAGGATGATGACGACATGCCCTCATGCAGCGGCGCTCGCCTGGCGAAAGTGCCTGCTGCCGCCGCAGCAGCTACGAGCGCCGCCAAGACCCCCTTAAGTAGGCCTAGCGGTTCAGACTCGGGGGAGTCGCATCGGGCAGAGACCGATGGCGCTCGTAGCAGGAGGAGGAGGCGCAGAAGAGGTGGGCAACGCCCTCTCCCAGCAGCTGGAAAACCCGGCGGGTGTGACGACCCGCACAGGAAGGGTATGAGTGGTGCCAGCATGAAGTGGTACCTGCGTTACCTCCGGGATGGGAAGACTCCCGAGGAGGCGGAAGCTTCAGTAAGAGGACGGAAGGAAGGGAGCAATGCATCCCCAGCAAAGAAGCGCAACAGGGCCGTGTCAGCTCGCACGCAAGGCAGCGGCACTAGAAACCGAGGCCCGGTCAGCACAACTAGGACCGCAAGGCGTGGCGACGGAAGGGCAGCGCCCACGGACACCTACGCCCAAACTGCGAAGCGGAAGAGCGGCCAGATCACGCCGCAGGAGCCCCCCAGCTCCAAAAGGATGAGGGGCGGCAACACAAGGGCAGCCGGAAGCCAACCATCCGAACCGGCACCCCACCGGGTGGAGGAGGGGCGGCGCAGATATGCGGATGCTGTCAAAGGCATCCGCATGGCTGTACTGCCTCAAAACTACCCGGCGGAGTTGCTAACATCGGAGGAGCTTACCGTGCTCCAGGACCTCCTCATGGAAGAGGTATTCAGAGGAGATGAATACGCGGCGTCCTTCCTTGGGGTGGACTTCAAAGGAGGTATGATACAGGTGGACTGCAAGGACGAGAGGTCCGCCAACTGGCTGCGGGAGTTCGCCCCAAAGCTGGAGGGCTGGAAGGGTCCTGTCCTCTGCGCGAAGAGGGCGGAAGATGTGCCAATCATGCACAGCATGACAATGTTCCTCCCCCGTTGCGGGGACAAGCCCTATGAGTTCGCCCTAGGTCTGGTGAAGAACCAGAACCTGGGCCTCAGTATCTCGGCCTGGCGCGTCGTCAGCAGCAAGGTGGAAAAAATAGGCGATATGATAGGTTGGAGATTGTACTTGTACATAGACGACGAGTCGTACAAGTACGTCCGGGCAGCGAGCTTCCGCCTGTTTTACAGGTTCAGCACGGTGGTCATGCGGCCCCACAAGCCCGCAGCCACCGGGAGCAAGGAAGCAGACAAGGCTGCGACTCAACAAAGCGGGGGTATCGAAAAACAGGCAGAGACTGTGGCGGCCCCGGAGGCAGAAAGAATGCAGGTGGACGAGGTTGCGAATCAGCCCAGCGAGAGCAGGGCTGGGCAGGCTATGCCTGCGGCGACCATCGACGTCCCGGATGTTAGTTCCTGCGGTCAGGGAGCAGAGCTACCCTCCACGCAGGAACTCCTTGAGGGGCTAGGAGACCCACTGGACGGCAGCGCGATTGACGGCGGGGATGAGGATTTGCCCCTCCTCGAACCACTATTATAGTGGCCGTCAACACGGAAATTGCCCAGGTGAACCTGCATCACGCGGCGGCAGCTTCGGCTGTCATAGCGAGCAGGTTCACAGCGGATAAACTGGGCATACTGCTGATCCAAGAGCCTTGGGTCCATAGAGGAGAGGTTAAAGGCCTCAAGACGGAGTCCAATAAGGTAATCTGGGATCTCTCTAGCGAGAGACCTAGAACTTGCATAGTGGTCAGGAATAACATTGAATTCTTCTGTATTTCAGAGTTCCTGACGCAGGATCTGGTGGCGGTGCAAGCCAGGGACTTCGAAGGCAAGGACTTCGTCCTGGCTTCAGCCTATTTTCCAGGGGAGGCATCCACGGCACCCCCGGAGGTGGTGGAAAGGCTGGTGGAGCATTGCAGAAGACACAGGCTTCCCCTAATCATTGGCTGTGATGCAAACGCCCACCACTTGGAATGGGGCAGTACGAACTGCAACGCAAGAGGTGAGTCTCTTTTAGAGTACATATTAGGTAATAGCTTAGCGATAGAGAATGTGGGGTGTGAACCCACATTCATAACTATAAGCAGGAGGGAGGTGCTGGACATCACCTTGAGCAATGAGCCTGCAAACGGATTGGTCTCACAATGGAGAGTCTCAACGGAGCCCTCCTTGTCAGACCACAGGATTGTAAGGTTCACGCTAAAGGTAGAGGTCAGACAACTCCCCCCCAGACGCAACCCTCGGAAGGCGAACTGGGATACCTTCAGGGAGATACTAAGTGAGGAATTGAGCAGGGGGGGGCAGACTGATAATAGCGTTTCAGGCCCAGGAATTGAGAGTAGGCTATCTGAGCTGAACGGGTCTGTTATTAACGCTTATCATGGCAGCTGCCCCCTAAAAGTGCCCACAGACAGACGAAACTGTCCCTGGTGGTCAAGGAAACTTGCAGACCTCCGGAAAAAAGTACGTAGCCTATTTAACAAAGCGAAACGTACGGGGGTCTGGGAAGAATATAGGAGCTACCTCACCTTATATAATAAGGAGATTAGGTCTGCAAAGCAGGCTAGCTTCAGGAGATTCTGCGAAAATGTCACCTCCACACCAGAAGCGGCTCGGCTGCATAAAGCTCTGGCTAGAGGCACGACTGACGCAGTACTAGCAATTAAAAGAGGTGACGGGACATTTACGACCAGCGCAGAAGACAGAGCTACGGAGCTTCTGCGGGCGCACTTCCCGGAGACTATTCGGGAAGACCAGTGTCTACCCGTGATCGAACACAGGCCATCCCGCGAGGATTGGAGTATCGCCAAACAGCTATTTACGGCGGACTCGATCAGGTGGGCAATGGCCTCGTTTGAGAGATTCAAGTCTCCGGGAGTGGATGGCATCTTTCCAGCGCTGTTACAACAGGGGGAGCAGTTTTTACTGCCACACCTGGTTCGGCTGCTGAGGGAGAGCCTCGCAATGGCGTACATCCCTGAAGTATGGAGGACAGCGAAGGTGATCTTCATACCCAAAGTAGGAAGGAAGGACTACTCATTGGCGAAATCCTTCAGGCCAATCAGTCTAACTTCTTTCCTACTAAAAACCATGGAGAAGATCGTGGACCATGAAATAAGGTCGAAAGCGCTGAAGAGTGCACCCCTGCATGCGGCTCAGCATGCATACAGAGCGGGCAGATCTACCAATACTGCTCTGTACCAGTTAACCTCTGAGATAGAGAGTTCACTGGAGAATGGGGAGGTGATGCTTTGCGCGTTCTTGGACATCGAAGGTGCCTTTGACAACACGTCTCACAGGAGTGTAGCCAGGGCACTGGAGAAAAGGAATGTGGCAGCACCGGTGCGTAGATGGATAGAAGCCGTACTGCGCACCAGAATTGCTGAGACCACAGTAGGAGATAAAAGGATTCGTCTTGGCACAACAAGAGGCTGCCCACAGGGAGGTGTGTTATCACCCCTGCTATGGAGTCTTGTCGTAGACGACCTGTTAGCACTGCTAACGGACAATGGGATCCGCTGCCAAGGGTATGCGGACGACATTGTAATTATAGCCAGAGGCAAATATGAGGACACTCTCTGTGACCTAATACAAAGAGGTCTAAACCTGGCAAAGGGGTGGTGTAGAGAGGTTGAATTGAACATCAATCCCTCCAAGACGACCGTAGTCCCGTTTACGAGACGCAGGTCCCTACCCGGTCTCAGGAATCTCACACTTGGGGGCAGAGAGATAGAGATGACCAACAAGGTCAAATATCTTGGTCTCACGCTGGATTCAACTTTGCGGTGGAAGCAGCATGTGAACCTGACCTTGGTCAAAGCAACAAAAGCATTAATGGTGTGCAATCGGCTGGCGGGAAAGTCCTGGGGCTGTAAGCCAAGGATCGTTAGGTGGTTGTACACCATGATAGTGCGACCGATAATCACGTACGGGGCGGTGGCCTGGGCCTCAATAGCGTCGCAGACTTCGGTAGGGATCAAACTATCGAAGCTGCAGCGGCTAGCCTGTGTCTGCATGACGGGCGCAATGCGCACCTGTCCAACGGCAGCACTGGAGGTCCTGCTTGAACTCACACCGCTTCATTTGGTTATCGGTCAAGTAGCCAAGCACACACTTCTGCAAATAACGGCAGAGGGGTGTGGCAAAGGTAAGGTAATCTCGTCCCAGAGGATGGAAAAGATAAGTGGCAATATACCATTAGCCCTCCTCCCGAGGGACAGCACTACCAAAACAGTTAACTTCACGAAGAAGTTTAAGGTCACCCTCGGCAGCAAGGATGAGTGGAACGACTCCACTCTCGAACTGATGCTGAGGAATAGTACGTTGAGGTGGTACACCGACGGCTCGAAAACGTCGGAGGGAATTGGTGCAGGGATCGCGGGTCCACGCACCAAGCTCTCCATACCTATGGGAAGTTTTCCGAGCATTTTCCAGGCAGAAGTCTTTGCCATAAGTCGGTGCATAGAGATAAACCTCCATCGCAACTATCGCAATGAGCGTATAACCATACTTAGCGATAGTCAAGCGGCACTAAAAGCGATCTCCTCCTACGAGATCAAATCGCTACTAGTGCAGGAGTGTAGAGAACGGCTGAACAGCCTAGCGGAACGTAACCAGGTGCACCTAATCTGGGTGCCCGGTCACAAAGGTATAGCCGGGAATGAACTGGCTGATGAGCTCGCCCGCTCCGCAGCATCCACCAACATGGTAGGACCCGAACCCTTCATTGCGGTGGGTCCCCATACCATAAAGGAGCTGCTCCGCAAGGAAGAAGGAGTAGGCAGAGAGAGGTATTGGCAACAACTCCAAGGCATGCGCCATGCCAAGTTGCTAATGGGTGGTTACAACCTCAGCAGGTTTAAATATGTAATCAACCTCCCNAGGGACAAACTCAGGAGGGATAAACTCAGGCTACTTGTCGCATTTTACACTGGCCACTGCAAGCTGAATAAGCACTTATATAACATGGGCCTATCTTCTTGCGCAAATTGCCGGTTCTGCGACAGGGAGCCTGAAACACCAGAACACCTGCTAATTGACTGCACAGCAGTCTGTAGACGCAGGATAAAGGCCCTTGGATCCATGTTTCCAAATAGGGATCACATCGCCTCACTAGCACCTAGCAGTATATTGGACTTTATCAATATGCTGGGGCTAAGTGAGTCGTTGTGATTTAGGAGAGGGCACAATAGACCTAAGGTCGCGGTGCAATCCTCATTTATTTATCTAATCTATCT

>Btry_4395#LINE/CR1

ATAATAATAATACCCAACGATTACCCTCCTCCCGCCCAACCGACGCGAGGGGCGCAATCCGCGAACGAGCGGAATTAGCCGAGTCATAAAAGGCAAGAGAGTTTTAAGCGTTGCGATCTTAAGCTGCTCAGCTACAGAAACAAAAATTTCAACAGCCGAAATTAAGAATTAGATTAAAGTTTATAATTTTTAAATGTTACTTGTTAAGAGTAGATAAAATAATTTTTTTAATGGTAAAGAGTGAGTCTGTTAGATCAAATGTGCTGGAATGAGAATTGAAATCATGACATATACGGCGGAATGGTTCGTTTAACTCAAAATTTGTTCTAGAAAATTTTAAAAGTAAGGGTCTAAACTGCCTGGTAGAGCGAGCGGGAACGTTAAAATTAATATCAGATAAGAGAGATGGGCTACAGACTGACCCATTCATGAGTTTTACAAGAAATATTATACCAAGCATCTCCCTACGACTAGCGAGTGTCGGGAGATTTATAAGTTTTAAACGGTTAATGTAAGGGGGAAGATTTAAACTGGAATCCCAATGCAAATGATTTAAGGCAAAAAGTAAAAATTGTTTTTGAACGGACTCTAACTTATCCGAATGGACTTGGTAACTAGGGTTCCAAACCACAGAAGCATACTCCAATATAGGCCTCACCAGAGATGTAAAAAGAATTTTTGTGGTAAGCGGATCTCTAAATTCTTTAGACCAACGTTTAACAAAACTAAGAGCGCCTTTAGCTTTAAAAACCGTGGAAGATGCGTGAAGATTAAAATTGAGTTTGGGGTCCATAGTTACTCCCAGATCAACAAAACTGCATACTTGCTCCAACCTAAAGTTTTGTATTGCGTATGAGGTAGGGTCTACAGCTCTACGTGAAAAGCACATCGTTTTACATTTTTTAAGGTTCAATGGCATGTCATTTCTATCACACCAAGAAACTAGGTTGTTTAAGTCTGTTTGCAACTGACATCTTTCGCTGTTTGAAGTATATGTTTTAAAAAGTTTTACATCGTCAGCATATAATAAAACTTTTGAAAACTTAACAACAGATGATATGTCGTTAATAAATAACAAGAACAGAATTGGACCAAGATGGCTACCTTGAGGAACGCCTGAAGGAACATTGATTATGTCAGAAAAAGTATCTTTAAATATGACTTGTTGAATTCTATTACTAAGATAAGAAGCAACCCATTTTAGAAATATTGGTTGAAAACCAAGAAGATCAAGTTTATTCAAAAGAATTGAATGGTTTACTTTATCAAAAGCTTTACTAAAATCTGTGTATATAACATCAGTATTCTTATTCTCCCTAAAGCCCGTTGATACATGTGATACAAATTCAAGCAAATTAGTTATGGTAGATTTCCCTTTACGAAAACCGTGCTGAGAACAAGAAATTAGTGGAGAGATCCGGAAGGTTATGTCGTCTGTTATAATTGCTTCAAAAAGTTTAGGTATTGCAGACAACTTTGCTATTCCCCTATAGTTTTCAATAGATGACCTAAATCCACTCTTATGCAAAGGAATTATAAATGACTTTTTCCATATGGATGGAAATAAGCCTTGCATAAGAGATGAGTTAAATAGTTTTGTTAGGGGTTGGTATATATATTTGGCACATTTCTTAAGAAAGCATGAGGGAATCATATCTGGGCCATAATTGTATGATTGTTCTAACATATTTAACTGATTTAAGACGTCTGCTTCGGAAATGGTAGGTGCATTAATGACAGAAATCGGACATAACTTGTGCTGATGCGGAACATTTTGTGGAGATTTTGGAGAGTAATTGGACCTAAAGAATTCAGCGAACATATTAGAAATGGTGTGATTGTCACTGGACATACTAGACTTATATTTCATAGCGGACGGAAAATTAGAAATCCTGCGTTTGGAGTTGACGAAACCATAAAACAATTTTGGATTACGTATAATATTATTTTTTACTTTATTTATATAATTATTATAACATATTTTGTTAAGTTCAGCAAATTGTCGACGCAATTTAGAATATTTTGAATAGTTAGCAACTAAACCAGTTTTTTTAAAAAGTTTAAAAGCACGAGATTTTCTATTTTTTAATTTACATAATTCTTTCGAAAACCATAAATTAGAACTAATTTTTTGAGTAACAACACACTTCGGAACATATTTTTCAAATAAGTTTACAATAGTTTCATTAAAGTGGGAAGCACTAAATTCAATATTGCCACTGTAATCTGGCCAAGTTATTTTAGAAAGTTCGTAATTAATCTTCTTAAAATTAGCTTTCGCAAAGTTGAACCGAAAACAAAGGTCTTGTGTATTATTATAAGCAAAGTTGGCTATGATTTCGATGTTAATTTCCAAAGCAGGATGATACGTGTCCTCTGGCCGAACCAAAGGATCACATCGGACAACAGAGAACATTGAAGCGTTATCGACGTAAACTAGATCTAAGAGCTTACCAAACTCGTTTGAAATTAAATTAATTTGGTTAAGACCCAACTCCGACATTTCATCTAAGAACTCGTTAAAACATAAACGAGTACTAATAGGGATGGTGTGATCATCAATAGATTTCCATGATACGCACGGTAAGTTGAAATCTCCCAAAACAATCATGGAATCAGTATCATTAACCATTGAGAAAACATTACTTATTAATGAAGCATGCTGCATATATACAGATATGTCCGAGTGAGGCGGTATATAAGACAATGTTAAGTAGATATGGCCACTATTAGCGCAAATACGAATGCACTTAAATTCAATGAAGTCTGCATGCGGAACATCGACTTCTTCAGATGGAATTGAAGAATGAACAGCAAACAAGACACCACCTCCTTTTCTGTTCAGCCGGTCATATCTAAAGATTTCGAATTCGCTGTTGAATATTTCGTTATCAAAAATGTGGGGCTTTAGCCAAGTTTCTGTGAAAGCAATGATTTTAAAGTTACAATGAATGCTATTTAGATACAAATCAGTAAGTTTTGTATTAAGCCCTCTAACATTTTGATAATAAATAATCAGCGAATTATTGTTAATTAGTTTTTTGTATCAACGGGATTTCTTGGCATTTTAGTGATAACTACAGGGGGCTTATCACGTTTGTGCTCGAATTCGCGCACAAAGACCCCAGATGGCCAGAATGAGTTGTCAAGTATCAATTGAAAATTGTCAGCGGATACGTCGATCTTAAACGAGGAAATATCCCTGTTGTATTTGAAATTAAATTTACGGATGTCTGTATCTACCGTTTTTAATTTCGACGAGATGTAACTTTTTATATCCTCGACTGTGGTATCCGCAGCAAGTCTCGAGATAAAAATTGCTTTTTTCCGCGGTATTACCACTAAGTTTTTATTAGCTGACTTGGAGGTATTTGGAGGCGGATCTTGAGAAGTTTTCCGCTTACCGTATTCAGTAACAGTTTTCTCTGTGTCCTGAGCAGACGGAACCTTCTCTCCTTGAGGACAAGGGGATGCAAGATTTATGAGGTCAATAGCGGGTGGGGGCATTCTTTTTAAGGAAGATACAGTGGTATCTTCATCAGACGGACGTTTTCGTTTAGATGAAGGTTTTGGATTCTCTTCCCGAATATTTAGGCATTGAAATGATTGAAACAGCGTTTCATAATGCCTAAATTTTTCAGTAAGGGTTTCAAGCTCACGACCAATCTCCTTGAAACCATTGCGCGCCTGCTTAAAAACTTTAAACAATTCAATTTCAGTTGGTCTGCATTTCAAGCAAGACCAGCGCAAGCCCTTACTGTCAAGAATGGAATCCAAAATCCTACCTGTCAGTCCAGCACATTTAAGATGGGCAAGCCCATCACAAAGCCAACAAGAAACAAAGCGTTCCGACTCGCCTTTCACAGAGCAGTTCGGAAAGTTACAAGTCATAATGAACAAAAACAGTATTAAACAAAAATATAAAAAATGTTAAATAATAAAAGTAAAAAGCGCGATGACAAAAATATTTATAACAAATGAATGCAATAAAAATAGTTGGTTATCAAAGATAACAACCAATGTAGCGAGCAGTTTGAGATGCACTGTAACACACGAAAAGTAAGAAACTCGCGAACAGCTGTGGACAAAGATGGAAATGAATAAAAACAAGAAAGAAATTTAAGTAAGCAATATAAATTAACACTAACAATTGCACTATTTAAAATGTAGCAACAAAAACAAATTGAAAATGACTCGGCACAATAAATTAAAATTAGCCAGAACAGAATTAAAGTAAATTTCAATTGCTTCACTAGCACAATATAACACTGATACTTATCTTAAAAGATCACTTAATTCAGATAATTTCACCACAAAGTTTTAAATATTATATAAATTAAACAAATTTCTAAGAGCACGAAAGCACAGCTGTACACGAGAAAAGTTGCCAGAT

>Btry_MarNcomp#DNA/TcMar-Mariner

TATACTCTGTGCCCAAAAAATAAGGTGACATTGTATTTATTTTGAAAATTCTTTATTTATTCTTCCAAATCAATTTCATCCCCTTCAAAGTAATCCCCTCCCGATGCAATGCACTTATGCCAACGGATTTTCCAATCTTCGAAACACTGGTTGTAGTCACTTTCCGGGATGGCCTTCAGTTCCGTCTTCGCTGCGGCTTGAATCTCCTCTATCGTATAAAAACGGTGTCCCCGGAGCGGTCTTTTGAGCTTGGTGAACAGCCAGAAGTCGCACGGCGCCAGATCAGGTGAATACGGTGGTTGCGGAACGATATGGGTTGAGTTTTTGGCGAAATGATCACGAATTACGAGGGCAGTATGGGATGGTGCATTATCGTGGTGTAAAAACCAAGAATTGTCTTTCCACAATTCCGGCCTTTTTAGGCGGATAGCGTTACGCAAACGACGCATAACGTTCAAATAATATTCCTTGTTGACTGTTTGACCGGTTGGAAGGAATTCATAATGCACAACACCACGATAATCGAAGAAAACAGTCAACATGACCTTGATTTTTGAGCGACTTTGGCGCGATTTTTTTGGTCTCGGCTCTCTTTTGGCACGATATTCGCTCGATTGATCGGTTGTTTCGGGGTCGTAAGCATAGATCCAAGTCTCATCGCCAGTTATAATGCGTTTCATGACACCCTGGTAGTCGGAAAGCATCGTTTCACACACTTCAACGCGACGCCTTTTTTCCAAAAAATTCAATGTTTTCGGTACCAGTCGAGATTTGACGCGCTTGAGGCCCAAAACATCCTTCAAAATGGTATTGGCTGAGCCTTTCGATATGCCAACTTCATCAGCAAGGTCTCTAATTGTTAATCGACGGTTTTTCAACACCAAATCTTTGATTTGTTGAACGTGAGCTTCGTCGGTTGAGGTTGATGGTCGCCCTGGACGCTCTTCGTCTTCGACACGTTCACGGCCGGCTTGGAACTCACTATACCACTTGTAAACATTTTTTTTAGACATAGCCTCATCACCAAAGGCTTTCTGCACCATCCTCAACGTATCCGCAGCAGAAAATTGATTCCGTAAACAAAATTTAATGCAAATTCTTTGCTCAACAAATTTCGACATCGCAAAAAACGAAAAACTCACTTTTAGCAGCTCACAAAACGACACGTATCTCAAACACTAATGAATATTTTGACATGAAATTTGACATAAATGTGACTGACAGTACTACCAACCTAAAAAAAAAATAATTCTCCAAATCCTTCGACGCGCGCAGTTTAAATTCAAATGTCACCTTATTTTTTGGGCACAGTAG

>Btry_516#DNA/TcMar-Tc1

GTTGTGGCAGATCGTTCGGCTTCAGTTCTTGCACGAGCTGTATTTTATACGGTTTTACACCAAGATCTTTGCGTAAAATCTTCCATGTGGTCGAATAACACAAACCCAATTGCTGCGAACGGCGACGAATCGACATTTCACGGTCTTCAGCCACACTCTCAGAAACAGACGCAATATTCTCTTCTGTACGCACTGTACGCATTCGTGTGGTTGGTTTAATGTCCAATAAAGTAAACTGAGTGCGAAACTTGGTCACAATCGCATTAATTGTTTGCTCACTTGGTCGATTATGTAGACCATAAATCGGACGTAAAGCGCGAAACACATTTCGAACCGAACACTGATTTTGGTAATAAAATTCAATGATTTGCAAGCGTTGCTCGTTAGTAAGTCTATTCATGATGAAATGTCAAAGCATACTGAGCATCTTTCTCTTTGACACCATGTCTGAAATCCCACGTGATCTGTCAAATACTAATGCATGAAAATCCTAACCTCAAAAAAATCACCCGATAT

>Btry_5167_Waldo#LINE/R1

GGTAAGGCTGAGCTCAGGCTACTACCGGGGTCACAGCTGGCGGATAGTGGACGGCCTACGCTAAGTAGGTTAGCTGCGAATAGTAATACGCAGCCCCCGACCAAGAGCTGCAGGAGAGGAGGGCTTGGCTCAAAACGCCTCACGCGCCCAATGAACCTCCCGCGAAGAGGCGAGAAGATGCCGCCTTTAAATAAGCGTCCACAACCCCCACCGGGGTTGCACCGAGGGAGAACCTACCCACGAGAGGGAGGCAACAAAGCGACGAGGGAACAGAAGACGACGAAAAACCCTGGGCAAGAGCCCATCAGAATTGAGACCAGTAAGATTCTGATTATGGTGTACTGGAAAACCGAAACGGATCAGATTGACAAAGGGCTGGGGAGGGCAGTATTCTGTCACCTCAGTAGATAAGAGTGACATCTTACCGAAATGGCTGGCAGGCTAATACTGCTCTCACCTCCGTCTCGTTAAAACCATGGCAGGTCTTCAAGTACGTTCAATGCACGTCGCCATAATTTCTTTGGCCGTACAGGTTCAGTTGAGACGAGCTCCTGATTCGTGCCCGAACCTGGCTCTGAACACAAGCTCCCATAAGGACTTGTGTCAACCCTCGCGTGGCCTCCCTGCTTGCATAGATAACCACGGGGTTCATAAAGGGCAACTGTTACAACGTGCGGAGATAGCGGCTTGAAGCGGAGACCCACTAGAAAAAAATGAATACACCGCAACAACAACAAAAAACCCAACAGAAGGAACAACAAAACAAAGATGGCCAAGAAGAGGAATACGGGACAGTCTTCCGTAGAAGCAGTAGGGTACTCAGATCCCCCGTACTCATCGCCACTCCAAAACAGACAGACAAAACTGGAGAAAAGACAGACTCAAGGGAGACTCCGGCCACACAAGCAGCACCGGCAAAACAAGGAGAAGGGAAGAGCACCCCAGCTGCCACTAAAGAAGGAACTCAAAACTCGCCGAAACAAGAGTACATCACCCAAATGAGACGAGCAGAAGAGGAATCCCTAGAAAAGTGCAAGGGTATAGTGCAAAAAATGAAGTTGGCGATGGCAAAGCAAAAGAACGTCAGCATGGACGTCAAGAACGGAGCGGCACAGCTAGACGAACTCTTCGACGTCATAAAGAGCTACCGCAGAAACTGGCTCACCGCAGAAAACGAGAAAAGGCGAGCACACCTAAGCAGACGCATCACGACCCCCGAGTCAGCAACCTCGAAGCGGCGTGCGACAAGCCCTGTGGAGCCGCGAGAAACAGTGCGACCACGACACGAAAACGACGGCCAGTGGCAAAAAGTTTTGCCTAAAAAAGCCAGGAAGACCCACGTAAACGAAGGAACGAGTGAAGAAGCGCCCAAAAACGCAAACAAGAGGCAACAAGTCAACAACATGGCCGCAAGCCAGAAAGGAAATAATAGGCCTAAACGACAAACAGAGGCCGTTATAATAAAACCAGCAGAAGGAAATAGCTACGCCGAAGTCCTCAAGAATATCCGTAGCAAGGTAGATCTTAAAGAAGCAGAGGTCAAAATCAAAGGGATCCGCAAAACAAGAGCCGGGGCCCTATTACTAGAGCTAGAGAAGGGACAGTCCACAAAGGCCAGCTTCTGTGAGGCACTTAAGTCCACTCTTAAAGAGACTGCAACTGTGGCCGACCTTAAAACTAAGGCTACAATAGAAATCAGAGACCTCGACTCGCTCACAACAAAAGAGGAAGTAGCAGGGGCTGTCAAAGAAGCGCTACAGGACCCCACCGAAGACTTGACGATTAACATAACAGCTCCTAACACAAGAGAGCAGGTAAGGGCATATATAACGCTGGACCAGGATAGGGCTGACACACTGATGCGACAGGCACGCATCAAAATCGGCTGGGTTAACTGCAGGCTGCGGCTGAAAGAAACCCCCAAAAGGTGCTTCCGCTGCTTTGGACCAGGGCACTTAACCTGGGACTGCAAAGGGCCCGATAGAAGAGGACAAGGTGTTTGCATAAAATGCGGCCAACCAGGTCACAAATTGAAAGAGTGCACAAAGCCTCCTTCATGCTGCCTTTGCGAAGAGGCTAAACACGAAAAAGTCGATCACATCCCGGGCTCCGCAAAATGCACGGTATATAGGAATTACCGAAACAAATGAATATCCTACAGGCCAACATGCATAGATGCAAGGCTGCTGACGCACTACTCTCGCAAATGGTGATGGAGAACGACTACGAGATAATCATCATAAGCGAGCAATACAAAAAGAAAGAGAGAGGTACCTGGCTAGAAGATAGCAGCTCAACCGCCGCTATATGGCTACCACCAGGGAGTAACGCCACCACTGTAAACAATGGGAACGGCAACGGTTTCGTCTGGGCCAAATGCGACCTTTTTACAGTAATTAGCTGCTACTTGACGCCAAGCGATAGTATACAGGAATTCCAAGAAAAGCTCGACAATATAGAGGACACAGCCAGGTCTATAGAAGGTCAATTGATTATCGCCGGAGACCTAAATTCCAGAGCCGTAGAGTGGGGTATGCCAAACACGGACTCGAGAGGAAAGCGCATTCTAGAAATGGCTGCGCGGCTAGGTCTAATAGTGCTCAACACGGGAAATGCAACCACGTTTAGAAGACCGGGATGCGAGGAAACCACACCAGACATCACCCTATCAACAGAACGCATGGCAGGCTCAATAAAGAATTGGAAAGTCCTGGAGGATTACACTGGCAGCGATCATCACTACATCTCTTTCATGATCGACACTGGAACAAACGCTAACCAGCACAGGAAAAATACTGGAACACGAAAGTGGAACATTTCGAAGCTAGATACTTCGAAATTAATCTCCGCAATAGACGACCAAAACCCATCTAGCAACTCCCCCATAATTGCAAGAAAAACTGTCGAGCACACAATGCTTAATATAACAAGAGCCTGTCAAAAATCAATGCCCAAGGCTTCCCACAGCAAACATAAAGCAGCAGTTTACTGGTGGACTGAAAATATCGCCCAACTCAGACGCACATGCCTCCGCCTTCGCAGATCCTACACGAGATCCAGGCGCAGAGGAGCCGCACCCCAAGAAGCCGAGCAATACAAAGCAGCAAAAAAGGAGCTGAAGCACGCCATCGACGACAGCAAAAAGAAAAAATGGGAGGAACTACGCGAGGATATAAACAGAAACCCCTGGGGACTCGGGTATAAAATAGTGATGAAGAAACTCGGCGCTCGAGCAAAGCCACCTGACTTAACCGCTGCCAAAATGGATCACATCGTGAACGCACTATTCCCCACACACGAAAAAAGGGCTAGTGATCCAGAACAAACTCTAAGATCTGCGCAAATCCCCCAGTTTACCCTTGAAGAGCTGCAACTAGCCACTGGCAAGCTCAAGGCCAACAAATCACCCGGCCCGGACGGGATCCCAGCAGAAATCCTCAAAATAATCGCTGCAGAGCGCCCGGCAGTACTACTGAATATGTACAACGCCTGCCTTGAAGCCGGAATATTCCCTGAACCCTGGAAAAAGCAACGGCTGGTGCTAATCAGTAAGGGAAAAGGAGACCCCAATTCGCCATCAGCATACCGTCCACTATGCATGCTTGACACAGCGGGAAAGCTATACGAAAGCCTACTTAAACCCAGACTCGAAGCGGCTATCAACGAAGCTGGAGGACTGTCCCCTAGACAACATGGCTTTAGACCCGGCAGATCAACTATAGGAGCTATAAAATGCGTCATTGAAAGCGTAGAAGCCGCACAGCGTAGATGGCATAAATACAAAAGAATAGTGTTGCTGGCGACTCTAGATGTCCGAAACGCCTTCAACAGCGCTAGATGGGTGGATATGATCGACGCTCTCGAAAAAAGCTTTAAGATACCCGACTACCTCAGGGCTGTGGTGCGGAGCTACCTTAGCAACAGAAAACTGCTGTACGAAACTAAAGAGGGATCACGACAGATAGCGGTCACGTCAGGAGCAGCACAAGGATCCATTCTAGGCCCAGACTTATGGAACATTAGCTATGACGCTATATTAAAACTAGAAATGCCAGATGAATCGTATTTAATTGGCTACGCGGACGACATTGCAGCAGTAATCACAGCAAGGGACACAGAAGAAGCGCGAAGAAAGCTGAATCAGGTCATGATACGGACGCAAGCATGGCTCGACTCACACAACCTCCAGCTCGCTACGGAGAAAACAGAGCTACTGCTGCTAACAAACAAGCACATACCTCTCGAGATAAGCATGCACACGACTACGGATATTCTTAGGACAAAAAAGCAGTAAACTACCTAGGCGTAAGACTGGACCCCAGACTAACCTTCTGGGTACAAATCCAGCACGCCGCAGGAAAGGCAGCGAAGATCACCTCTCAACTGAGCCGATTAATGGCCAACATAGGAGGCCCCAGCCAAGAAAAGAGAAAGCTCCTAATGTCGACGACAATCAGCGTCTTATTATACGGAGCCGAGATCTGGGCAGACGCGCTTAAAAAGGAAAACCGGCGTAAGGTAGTGGCCAGAGTGCACCGCACCGCAGCCCTCAGAGTTGCATCAGCCTACCGAACAGTGTCAGGCGATGCAATATTAGTTATAAGCGGTAATGCCCCAATTGACCTTCTAGCATATGAAAGGAAAAAGCTGTGGGAGCTAAAGAAATCATCCGATAATAACAAGAGCGCAATACAACAAATAAGGAAAGATACAATAACAACATGGCAACAAAGATGGGAGAATGAAAGTCGCGGCAGATGGACGGCCAGGCTAATAAAAGATCTAGACTTATGGACAAGCCGTAAATTCGGAGAAGTAGATTTTTACACAACCCAGCTGTTATCCGGTCACGGATACTTCAAAAAGTACCTCCACAGAATGGGAAAAGTCGAAGAGCCGTCATGCCTATACAACGACGCGACAGAAGACGACGCTGAACACACATTCTTTGAATGTGTGCGCTGGCAAGGAGAACGCACCGCCGTAGAAGACCTGGTTGGCCCAATAAACGCGGACAATTTAGTCAGCGTCATGTTGGAGAGTGAAGCAAACTGGGGGATTATCAAGAAATTCGCAGCAATCTTGCTACGCAGCAAAAAGCGGGACCTGGACGCAGGTGCGCAGATGTAAATCTCTCAATGAGAAAAATGGAACGCCACCCTGAAGT

>Btry_MarO#DNA/TcMar-Mariner

ATACCATCTGATCAAATTTGACCCGGACTGGTCCATTTAAACTGCGCGCGCAAACCGAATCGATAAAAATTTTTTTCCTAGATTGGTACAACCTTTTTTATGTTCGTGTGAAGTTTGATCTCCATATGTCAATTAGTGTGTGTTTGGCAGCTGTTTGTTTACGACGCGAAAAGTTTGTCTGGCGATTTTTACCATGAAAAAAATTAACAACGAGTTTGCTTGAAATTTTGTGTTTCCAATGGAATCACGGCTACGGAATCGTTGAAAATGTTGCAGAAGTGTTTTGGGGAGTCTACTTTATCACGAACACAAGTATTTGAGTGGCACAAAGCATTCAGTGAAGGTCGTGAAGTCATCGAAAACTTGCCTCATGCGAGTCGTCCATCCACCTCTGTTAATGACGATAACATCGAAAAAGTTAAAGAAACAGTGCTTGAAAATCGTCGTGTTGGCATCAGAGAGATAGCAGAGGATCTCAACATCTCTTATGGATCGACTCAACACATTTTGGTTAATGTTTTGGGTATGAAGCGTGTCAATGCTAGACTCGTACCAAAAGACCTGAATCTTTTGCAAAAACGACGTCGAGTAGAGGTCGCAAAAGAGATGCTTGACAACGTAGCTGAGGACCCTACATTCATCAAACGCATCATTACTGGTGACGAGACGTGGGTTTATGAATATGACGTCGAAACTGTCCAACAATCTAGCGAATGGCGCTCCAAAAATGAGCCGAAACCGAAAAAACCAAAATTCGCTGAAGGCACTGAAGGCCATCCCAGCCGAGGCTTATAACAAGTGTATGGAAAATTGGATTAAGCGTTGGCATGCTTGTATTGGCTCAGGAGCCTATTTTGAAGGCGATAATAAAGATTTGTATTAAAATACGTGAAAATATTTTTTTTTGTCAGTCCGGGTCAAATTTGATCATATGGTATA

>Btry_1831#DNA/Transib

CCACTGCACACTGGTCGATTTCATAGGCCAAAAATAAAAAAATAAAAGTTTGAAATTTACCACTGTTTGTATCAAAGCTATTTCTTAAAACACCAATAAAACTAACGGTAAATACATTTTTGCTCTATCCCATTCGTAGGGGCTACGAAAAGCGAGAGAAGTGAGACAATAGAAACACGCGGACTGTGCAGTGAAAATAGCTTAAAAATTATCTCTTATAATAAATTAAATTGCAATATAAAATCGTGTTTAATCCCCATAAATCGAAATGAGTATTGATAATAAAGGTATATGTTATATAACAAAGTAGTTTTTATGTAATAATATGTTGTTTTATTATTTTAAAACAATTGTTTTATAAGTGCCCTTTTGAGTGGTATGTTTTTCTTTATACATCGATTTAAGGTATACAAAAAGTTGTGATCCCTGTCTCGCCCTCAAATGAATCAAGTTTGTTTATATTAGTTAATGTTTTCTAAGTCTATGCTTTTGATTTCAGCTGCAACTTTTTGCAACTTTCTTTGTTATTCATGTTATTCTACCAGGCATGCGATTTTTTGTGTGTTATTTTTTATAATTTTTTGTATTTACGTGTACGTATATTCGTGCATATTTTATTGTTTTTTATGTTTTTTTTCATCATACATATATACATATTTGAGCTTTGGTTTTCATTTTATCATTTTCTCACTTTTATCAAATGTTTGTTTTATACGTACTTTTATTTAGGCACATCATATAACTTTCACGCCTATATATTTTTGAATTTTGGTGTTTAGAAAATGTGTGAAGTAATATCACTTATCACCGAAGCAAATTTAATAAAGCTTCAGTATCTACTTATTAGAAGCTTCGTTAACAAAAAACTTGATATTAACTTTTTACCCTGCTATGATAACATTTTAAAGGAAAAACAGTTATCCTGAGCAAATAAAGGTCTCAGAATCTGATGTTGAAGTTGAGATAAAAAAATTATTGGACCACATTGCGTCACAAATGTAATTAATGAACGGAATGAGTCCTCTAATTACCTATTATCAATAGGAATATGTGGCTTTGATGGCAATTCTGGTCAGAGCGAATATAAGCAGAAATTTGAAAATAATAATTTGGAAGACAATAATTTATTTGTCACGGCATATGTCCCTGTATAACTGATAATAAAATCGCAAAATGATGAAGAGCACAAACAAATTTGGAAAAACTGCAGACCGACCTTCGTCCACCCGCTATTGCCGTCCTGTTCGAATTCAATTTCAAAAGGAATTAGTTTAATAAACCCTCAATGCACTAGATTTGCGCTAGATCCTGCAAAGGAATTAACTTCAAAATATTCCTGGTATTATCTTCCTTCGTCAGTCCGTAAAATTTTAATTCACGCTCCCGATGTTATTCAATATGCACCAGTTTCAATTGGAGAGCTCTCATAAGAATCTGCTGAGTCAAGCAATAAGGACGTTAAGATGTATCGACTCCATCATACACGAAAGAACTAACGGATCGCTACAAATCAGGACTTGCTAAACCGATTGCTTTTGAGCTTAGATCCTTTCATCACCAGTCAAAGAAACTATCATGCAAAAGTAAATCAATTTTATTAAAATCAGTTTTTGATTTAGTTGAATAATATTCAAAAAATTCCTTTTTTCGTTTATCAACTTTTTTAATTTAATATAATTAATTAAAATTTGTTACCATCGGGGGTGGCGGAGTGGGAAACGGGAGAGGGAGATTTTAAAAAGCTTTGGCAAAAGTTTCACTTTTATATAGGCAGTGGAAGTATTTTTGGCCGCTGATTCCATACAAGACCGACCACTGTGCAGTGGTTCGA

>Btry_1059bp_dispersed_32TIR#DNA

ATCAGGGGTCGAATCGTTACATCCGTGAACGTATCACTCGTCACGTAAAAGCCTGTATTTCGTATTATGACATACGTGATCGTAACGCTTCTATCGTAATCTGGCATGATGACATACGTGAACGAGTATATAAACGTATCCGTTCGTTCGAATCGTCATTCGAACGCAAACTACCAATCGTAATAGACAAATTTATGGAAATGTGAGCAAATTTTTTTAAATTTTATGTTTTTTATAATTTTTAGTAATAAAGAACATAAACAAGAAACCAATGAATGGAATATCTCATTAAAAATTAAAAAAAAAATTATTTATATGAACAAAATTTTTGTATTTTAGTTGACATTAAAAATAAAATTATTTACAAGGTTAAGGACATATACATTTTTGAATTAAATTTATTAAAGAATACTTTATTTGGTCTCTTATCGTTTGGTCGATTGCGGTGAGGCGGTTCGCTACCCCGGTGTCCATTTCTTGTGTGTGATAAGAATCGCAATTTTGAGGGTCGTAACTTATTGTAAATTTTATGCAGATGTTATGTAATGCCTCACAAACGTTAGCAAATCTTCCACTTTTGTGGGGTGATACCTTCCTCTCTTGCCATATCCTAAAATTCTCCACCGTCTTTTTAAGATTCCAATGCTTTGTTCAACGATACATCTACAAAGAAAATTTTGTTAATGAGGATTCATAATTTTTTTTTTATACCTACGCTATTGCTACGATCTCTTAGCTATCTCCGCACTATTTTTTTATCCCCATCACTTTCACTAGAGCTCAAATAAAAAAACAAAGTTGGATCCATTTTGATATTTAATTAGCTTTTGTTAGTTTATTGAAAATTTCGCAACAGTTTTGACAGTTCCACATCTATTGTGACCTAAAAATACGATCCAAAGCAATGTGGAATTTAAAAACTATTGTGAATTGAAAATACATTACGATCCGTTTGCTATCGTATGTCATAATGCCAATCACCGCATACGATTGACGTATCACGTAATTTTCTTTCGTATGTTTGCCGATCCGTGCACGGATGTAACGATTCGACCCCTG

>Btry_1183#DNA/hAThobo

CAGTGTTTCCCAAAGTGGGCGATAACGCCCCCTTGTGGGCGCTGCAGGTGTCAAGGGGGGCGGTAAGAGACCCAGAAAGAAAATGGGGGCGTTGTGTAGAGGCCTGGGGGGTTATTTGTAATTTTATTTAGCTAGGAGGCCTCTTAGACAACGACTACATGAGCTCTCGACTCTCAACAACAACTTGTGAACATCAACTAATATGAATTAAAAGATTGCTCAAACTCGGTTCTATATTTCTCTCCGCGTAGTTCATATATCTGTCCTATTTTATTGAATATAGAAAAAATGAAAATCATGTCAATCGGTCGCTCCGTTTCATAACGGGGCATCTCGACAGGATAGAATTTATAGAATACTAACTGTCAAACGTATTGCTATTGCCATTGCCAAATCTGTATGTGGGCATTTGCTATCATAATATAATCATTTGCGCAAAGGCGTAGGGTAGGTAGGTTCGAGCTGATGTAGCGTATGCTTTGAGCTCTTGAAAAATTTATTTTATCATTTGCGAAATGCCGTCGGGTAGGTAGCTGATGTAGGGCACGTTTTGAGCTCTTGAACTCCTTAAATCTTTATATGGATGAAAAAAATTTATGTGGAAAATAAAAAAAGGATAAAGATCCTTTTGTTTACAAATGTATTTCTGGGAAAAATAAGAATTCATATTTTTGGACTACTATATAATAATTGTGGCATAACTTTTATATACCAATTAAAATAATAAATTTAAAATGAGAAATGATATTTTGATTTATGCTTGTATAAGTTTATTAAATTTAAGACTTCGCTTATTCCCAACCCAATTTGCATAATATTTCTATAAATTAACAATATTAAACTAAATATTAATTTTTTGGAAAAATGTTATTTTGAAAATGTACTTTATTTTTATAATATAACACAACCGTCTTAACTCGCTCTTCTAATTTTCATATTACCGAAATTAAAATGCCGTCGGCATATGTGGAAATGGGATATGTTGCCTCTTCGAAATTTTCATATAAATGAAAACTGCGCTGTTAAACTGCTGAAGAGACAGCTTCTAGCTTACGATATATGGCAAACTATGTAGTATTCTATATCTAGTAAGCAATGAGCAATGTGGAGTCTGCACAGGCAATAGGCACGGCAATACAGTTAGTAAGACATAGATTTTATCCTGTCGAGATGCCCCGTAA

>Btry_2369#DNA/hAT-Charlie

TAACAAGTTTTTGTTAAATAACTCAAATTGTGCATTCCTGTATCAATCGGCTGGTTCATTGAATACTTGTTCGGACTTGTTGTGTACGATTGAGCGTCGAATGAGATAAACGAGTTTATGCATGTTTGTTTTATTCCTGTCGGTGCGTGCTGCTTAAGCATTCCTATATAAACAATGTAATATTTATTTTATATTCATTTGTGGTTTTGCGCGCAATAGATGAGCATTACTTACGCCTCCTTTACACTACTCGCGAAGTTAAACGTATTTCTCAAAGCAAAACAATGTCGGAAGTAAAAAAGAAGAGACGGCAATACTGTGCGGAATACATTAAATTCGGATTCATTGAAAATCCCACAAACCCATCCTCGCCTTTGTGTCTTCTATGTCTAAAAACATTTTCGAATGAAGCAATGAAGCCTTCAAGACTGCAAGATCATTTGAATAAAATGCATCCAGATAAGAGAGACAAGAATGTAGCATATTTTCAAGACCTAGAGAAGAAGCATAATACTCAGCCAAGTGTAGCAAAACTATTTTCGGCGGCTGCTAAGCAAGATGACGACGGACTTCGAGCTTCGTACAATATCTCTTTGTTAATTGCTCAAAAAGGCAAACCACATAACATCGGAGAAGAGTTAATATTGCCAGCAATAAATGAAGTAATAACTACCGTGCTTCATAAACCGGCCGCAGATATTATCCGAAAAATTCCTTTGAGTAATAATTCTGTGCAAAGACGAATTGATGAAATGGCTGAAAACATTGAAGAATCATTGTGCGATCACCTGAGGTCAAGTCAATTTTCAATTCAGCTGGATGAGTCCACTTTACCAACTAATGAAGCATTGTTGTTGTCTTACGTGAGTTTTATTAAAGATGAGAAAATATGTGAAGAACTATTATTTGATAGAAATTTAGAGACCGATACAAAAGGCGAAACCATATTCAATATATTGGAAAAGTTTTGCGATGAGAAAGAAATTCCTTTGAAAAATATTATTTCAGTTGCTACGGATGGCGCTCCGGCTATGACAGGGTGCCATAAAGGCTTCATAGCGTACTTAAAAAATAAAATTCCAGATGTCCTTGGTGTACATTGCGTTATTCATAGACAACATTTAGTTGCTAGAAACTTAAGTGAAAAACTATTTCAATCACTGCAATATGTCATCAAAGCAGTCAATAAAATCCGCAACAGCTCTTTGAATGATAGATTATTTCATCAGCTTTGTGTTACTAATGACGAGGATTTCAATCGTTTGTTGTTTCATACTGAAGTTCGTTGGCTATCAAGAGGCAATTGTCTGACTCGATTCTACAACCTGTTTGACTCTGTTATAGAGTTCTTGGAAACTAAAGATACAGAATTTCAAGACAAGCTCATAACATCAAAGAATGATATAGCTTACATGACAGACCTGTATAAATTGTTCAACGACATGAATCTCCAACTGCAAGGCGATAAACTAAATTTAATTAAAACAAAAAACGTCGTTGCTGCTTTCGCAGCCAAACTGCTTCTACACAAAAAGAATCTGGGCAGACGTGAGTTTCACAATTTTCCGAATTTATCAGTATCATGCAGTAACGACGAATTGTTTGCCTACTGCCAACATTTGGAAAACCTCCACATTGACTTCACCGAACGATACCAGGACATTTTGAACTTGGAAATACCAGACTGGGTGTTGGATCCGTTTTCAAATGTCAACACAGCAATGTCACCTCAGCTGGAAGAAGAACTTATAGAATTGACAACAAACGAGGAAATAAAAATCAAGTTCAAAAATGGTTACCAAGAATTTTGGCTACAAAAACCGATCTCGCAATTGTACCCTGGATTGTGGTCAATCGTTCAACGATTCTTGATAGCATTCCCATCGTCATATTTGTGTGAACGTGGATTTAGTGCTGTGACAACACTGCTTACTAAAAATAGAAATCGTTTGCTTGTTACCGAACGTGGCGACTTGCGACTGTTCTTGAGTAAATTGGAACCTGATATTAACAAACTTATTAAACAGCATCAGATTCATCCTTCACATTAGTTTTTATATATGGATCGATTATTTTAGTTTTATTTTTAATAAAATATTCTCTGTTTTAATACTATAAAGTTGTGTTTCAATAATCATTCTTATTGGCAGGTGGAGCCCGTAAGAGTTAACTTGAGACCTAAGCGCCGTTGTATGTTACCTACTGCGCTCAGGTTTCAAGTTGCTGGTTATAGTAAAAGTTTCGTTTAGAATTCTCCGTTAATATACATATATAGGTCACAATAATTAATTTGAAGTTATAGTGGTTTTTAAATAGGTGAAAAAATGGGGGCGCTAAAAAAATATTTATTCTCAAAGTGGGCGGTAGACAAAATAAGTTTGGGAACCACTGGTCTA

>Btry_837#unknown

TGTTCAAAAGTATGGGACTCTGATCCCATGAACAGCAAAAAAAATGCAAAGTTGTCGCTATACGGCGCAGTCTGTTCGCTTAGAAGGTTGTGTGAAGGAGGTAAACATGTAAATTGTATTCTTAGACTTGAATTAATGAAGAAATCAGTGTCTTCATTCATAAAAATATGCGCCGTATAGCGACAACTTTGCATTTTTTTTGCTGTTCATGGGATCAGAGTCCCATACTTTTGAACACTGATTCTTATGTAAAATTTAACGAGGAATCGAATGGGAAAGTCAATTTTGAAAAAAAGTTGGTGGAAAAGCACAAAAAATGGGATTTTTGGAAAAAAATTTTTTTTTTTTGGATTTTGAGCGACAACTTTGAATTTTTTTTGCTGTTCATGGGATCAGAGTCCCATATTTTTGAACACTGATTCTTATGTAAAATTGAACAAGGAATCGAATGAAAAAGTCAATTTTGAAAAAAAATTGGGGGAAAAGCACAAAAAACGGGATTTTTGGAAAAAAAAATGTTTTTTTTGGATTTTGAAAATTTTTTTGGATTTTGAAAATATTTTTATCAGAAAGCTGAGATTTTTTTACATAAAATTGGATAACTGCAAAATTTTTTTCACTCAATTTTGAGGTCGTTAAAAAATAAAAGAAATAGTCATTTTTTTGTTTGATCTCAATTTGAATTGCGCGCCAAAAAACAATGGAGCAACTTTGACCACTAAAAAGTTTACAGATATTCTTATTTTGGTCTATATTATGATATTCTAAAGTTTCGTCAAAATCGGAGAACCACGGGTACAAAACCATGTCGCCAATTGATGGAATGGCCCATATATA

>Btry_1325_partial#LINE/LOA

CCTATACTAACGTATGGTGCGGTGGTATGGTGGATGACATTATCGAAAAAGTGCCGTATTAAATCCCTGGAACAGGTGCAACAGGTTTGCTTGCTGGGGATTACGGGCGCAATGAAAACTACCTCAACAAAGGCTCTGAAGGTTATCCTCGACATCCGGCCAATACACATACAAGTGAAGTATGAGGCAATGCTTACGGCCAGCCGGCTCATGATTTTAGGCCATTGGAGTCAGACTCGGCGTCACTACGGGCATGGTATCATACTCGACCTTGTTAATGAACTCGGGCTAAATTGGCAATGCGACCGAGAAAAGGAGATCCATGAGGAATTAAACACAACGTTTTCATTCCCCAGTAGAGAAGAATGGACCAATGGTTCTGTGATCGACAGTTCCAACGGCGAAATCTACATATTCACCGACGGATCAAAGGGTGAAATAGGAACAGGAGCGGGCTTCTTCTGTAGTAATCCATACACAGAAGGTAGCTTCAAACTAAATGACTACAATTCAGTCTTTCAGGCCGAAATTGTGGGCATAACAGAAGGTGCCAAGTGGGCTTCTGCTCTAACCAGCAAGTCCATAAACCTTCTAGTGGATAGCCAAGCGGCAATTAAGGCTATCCGTAGCGCCAATACTAAGTCTCACTGCGTTAGGTTATGCAAGCAGGCAATAACTAGACTCTCAGTAGGTAACTCGGTTAATATCATCTGGGTACCCGGTAATAGGAAGGAAATGAAAAGGCAGACGAGCTGGCCCGCTCGGGGGCAGCTGGAAACACAATTCCCCTAAGCTGCCCCAGACCATTCAGCTCCTTCAAGGGTTGTTTGAAGCAATGGACCCTAGAGGAACACCTCAGGTCTTGGAACACAAGCAACACAGGGCATACCACAAAGTTACTTTGGGGTAGTGTTGATGGTGACAGACCAAAAAGCTCCTACTTCTAGGCAAAAGGAAGAACTTAGTAATATTGTAGGGGTCATCACAGGGCATACCCTTAAGATCCCACCTTCAGAAAATGGGAAAAGTGGATTCGGCGGAATGCAGAGCATGCGCGGAGGATGATGAGACGCTGGAACATTATCTATGCGAATGCCCAGCCTTCAGCCGGATGAGAAGGGACATATTCCACGGCTCCCAATACTCCAACTTAAGAGACATTGGGCAGCTGGGGTGGAAAAGAATCAGGTTTTTTACCAAATCCACTGAGTTGCTGGATAAGGCTTAATTTGGTTACCACCCGGGAGCACAATGGGCCTAATGATGGCCTAAGTGCGGCCGCCTGCGGTCTGGCGCTCATGCGCCTCCCTTTTCAACCAACCAAC

>Btry_729sTIR#DNA

TTAAGGGGTCCCGGTGGTCTAGAACCCTCAAATTAAGGGTGTTTTCTGGATTTTTTTTAAGGTTTAAAAAAAGAGCAATCGATTTAAAATTTTTACAGTTTATTTATACACTTATGAAAAGTACAAAAATATTTTTTTGTTAAATAAAAAAAATTTTTAACAATATTAAAAATGGCGTCCAAAAAAAGCTATCTTAAAAAATGACTCCCGGTGCCGTTGTTGATTTTGACTCTTCAGAACATCTGAAACATAAAAACCAAATAAATTATTAATCAGTAGGAGTGCAGCTATCGCTGGGACTACAACCAAAAAAAAATTGAAAATTAAAAAAATTTCGCGCTTTTGAAGTTCAGATTCTGAAAACAGCTATTTTTGGACCAGTTTTAGATCGAAAAAAATCGAAGTTCTTAAAATTAAAATTTGATCGTAGTCCCAGCGATAGATATTGATCTTATAAACACCATATTAAAATTTCAAGTGATTCGGATGGATAGTTTTTTTTTCATCAACGGCACGCCGAAAAAAGTAGTTTTGAGATAAATGACGTACAAAGCATCCATTCATTGAGCCCCTCGACCGCGCGCTACCTGCTAAAACGGCTGTAGAAGCTAAAATAATGTGAATATCCTTCCAAAAATTTTACAGTATATTCTTAAAGGACTATACTTTCGAACTATCAAACAAAAAAAATCGATTTTTTGAAAATTCTAGACCACCGGGACCCCTTAA

>Btry_3610#LTR/Gypsy

AAGTAGCTCAGTACATGTAAAATTAGATGACGAAGCACTTAAAGCAGTTGAAACACTAAAAAATAAACTAAAAGAACAAATAGAATTATTCCAACCAGATTTTGGAAAAGGATTTGATTTGACTACAGACGCAAGCAATCATGCGATTGGAGCTGTATTGTCACAAGGCAAGAAACCTATAGCATTCATTTCACGTACATTAACGCGAACTGAACAGAATTACTCAACAAACGAAAAAGAAATGTTAGCAATCGTTTGGGCACTGAAGAAACTTAGGAATTATCTGTATGGTCTCACTAGTAATACTACTATTTACACCGATCATCAATCCCTAATTTTTTCAATATCAGAGGACAACCCAAATTCAAAACTAAAACGATGGAAAAATTTTATAGAAGAATACGGAGCAAAAATTACATATATTAAAGGAGATTTAAATGTAGTAGCCGACGCACTCAGCCGTCAAGTAAACGTAACAACGAACGACTCAATACACTCGCAGGAATCATCCTCGTCAATCAGAATTAAAGATGTCAGCGCTCCACTGAACCAATTTAAAACACAAATTGTGATAAAAAAATGTGAACAAAATGCATTAGAAAGAGCAACAATTTTTGCAAACCACGAGAGATTTTATGTTAATTACATCACATCACAAGACTTGATAGATACACTAACGAAAATTATCAAACCCAATATCACGACCGCATTTCACACTACACTAGAAACCTGGTATGAAATTCATGATATTATAAACTCTTCATTTAATATAGAGAAAAAGGTTTTTTGTCAAAATCTATTAAAGGACATTACGAGTGAAGAGGATAGAGAAAATATAATTAAACATACGCATGAAAGAGCACATAGAAACGCAAAAAATAATACATTAGAGATACTAGAAACTTGCTATTGGCCAAATATAAAAAATGATGTAATCCGACTAACTAGAAATTGTAGCATTTGTAAAGAGAACAAATACGAACGCCACCCGACAAAACAAGTTTATGCAAAAACACCCATACCTGAAGGTATAGGCACACAAATACAAATGGACATATTTGAAATCGATAAAACAATGTATCTCTCATGTATAGATAGATTCTCAAAATACGCATATATGAGAAAATTAAACGACAAAAGAGAATTCTACAAAATATTAGAGGAAGTACTCATACAAATATTCCCATTTTGTAATAAACTAATGACTGATAACGAGACTATATTTAATAGCATAGCAGCAAAAGCTCTATTCCAACGGCTATATATTGAACATTCAATGACCGCAGCAAACCATTCAACATCGAACGCTCAAGTTGAACGGCTGCATTCAACCATTCTGGAAATTACTAGAATGCGCATTAAACAAAATAAATCAACTGCGACTGAAGAACTATTCAACGCAATCAAGGAATACAATAGGACAATACATTCCGTCACAAAAAGGAAACCTATCGAATTATTTTTTGAAAGGAGCAAGACTGAAGGAATAAAAGAACTGATCGAAAATAAGCAGGAGTATATGCTATCATACCACAATAAAAAACGAACACAAAAGGAATATAAAACAGGACAAACAGTTTATGTGAAACAAAACCGACGAAATAAAACAAATCCACGCTATTTGAAGAAAACCGTACAAGAGAATCGTGAGGACACAATTTTAACAACGGAGGGGAAGATCGTACACAAGGACAACCTCAGAAACAATGATGATGATAACGCTAACATTAATCCTAGCAACATCCTTAGCTCAGGAAATAACCGACTTACAAGATCGCAAGTACGTGTTAACCGAGACTGACCCATCATTTTTGTATCAAGAACATGAGTACCTATTCCATATGACGAACTTAAGCAAAATACTTAAACCATACTACAAAATCGCCGAACATAAAGGAGAGATGCCTAGTGAAGACGCAACAGAAACAGCATTGTTCAACAGAATAGAAATCCTCAGAAAACAACTACTGAATAATGGACGAGCCAGACGCTCAAGATCTCTTGATTTCTTAGGATCGATTTTGAAATTTATCACAGGAGTCCCAGACCACGTCGACCTGATTGAAATTAAAACAATACTCAACGACATAATTGAAAATAATAACAAACAAAGACAAATTAATTCCAACTTTGAAATAATGATGAAAACATTTAATATTAAGACAATTAAACAAAAAGTTGTATTGCAGGAATTATACAAGGAGTTATTTGACTTAACTATGACAATTAATTTCGCGAACAACGGAAATTTTTATTCAGCAGCAATAAATACGGACGAGATTGAAAGGATATTAGGAAAGGGACAAACTAGTATACCGGTTATTGACATACTCGAATATGCAGACACGCATGTAGGTAGATTAGACGACACTTTCATTGTAATTTATAAATATCCAGTCATTGAAAAGAAATGCACAACATACAAAGTTACTCCATTATCCTATCGTCAAGGCATTTTGGATATGAATAAAATCGTAAGTAAATGTGGAGATAAAATAATATTAGCATCTAAATGTAAAAGTAATTTAAGTAAATATATTTGTAAACAAGAACCTAATGACAATTGTACAATACCAATGATCAAAGGTTACGAAGCCCATTGTAATGTTATCCAAGAAAATAACGCACCTATTATAGATTTAGGACATGGTAATGTCGTATTACAAGGAAAACATATTTTAAACGGAAAATCAATTGAAGGATCGAATTTAATACAATTTAATGAAACAATTATTATTGACTCAGAAGTATTCCACAACCAGGAACAAGAAATTAAAGATTATATTATAGCTAATGGAAACGAAAGAATTCACATTCTTGAAATATTGAAATCAGAAAACGAATATCAATTTGACAACATTAAAAAATTAAACAAATTTTAATACCATTTGAAGAACACCCAATCAAGAATATGTTCATAACTATAGGAGTACTTGTAGTCCTAGGATTAAGTATATTTGTAATATTCAAACTATATATAATTTATCAAACAACTGTATATCTAAGAAACTACAACGCTACCATAGAACGTGTAGGAATGACTCATCTACTCAAGACTCAAGTATAGATCAGGAGATCTATTCTTCACAAGAGAGGGGAGAGTTAATACTTTAACCTTTAACCACAACCTTATCTTTCAGTAAACATTAAAGAAAAATCATCAAAACAACTTTATCTTAGAACAATAGAAGCGCACTATCAGTACAACAAACAATAACAACCCAAATAGTTATTTACTAGTAAACAACTAGCATCTCAATAGTATGAGCAGCGATAAGCATAGCCTATATAAGCAACTGGAACAATAGCCTCGAGGTCACTTTATCATTATCGACTGCCGAGTAACAAGTTCTGTATACCACTTGTAACACTTGTACAGTTTAAGTTTAATATATTCAATACTTAAGTACATGTGTATACTGTGAATTATTAAACGAATAAAGGATCAGTCAGTACCAATCTCATTGGCTGACTTAAAATTATATACATACATATATATTTATTTTATTCCTAACCCGAGTGGTTTTCATCGCATTTACGTCAAAATAAATTAACTCGCGCGA

>Btry_MarP#DNA/TcMar-Mariner

TATTGGGTCGTTCACTAAGTAATTTCGTTTGATGACAACAGCTGATCTTATTGAATTTTTTCGCAGCCAACTTCACACTTAACCGCTTTACCGTTATATATTTGAACAGCTGATATAGTAAGGCTTGTTTGTAAAAAAATTTCTTAAATTTTTTGGAATAGTTTTTGTTTGGTGCCCTATCGAAGATGGAAGATCGAAAACAGCATTTTCGGCATATTTTACTGTTTTACTATCAAAAGGGTAAAAATGCAGTTCAAGCAAGGCGAAAATTATGTGATGTGTACGGAGAAGATGTGTTGACCGAACGCCAATGCCAAAATTGGTTTGCAAAATTTCGTTCCGGCAATTTCGATCTTGAGGATGCACCACGTCCTGGAAGACTCTTGAAGCCGATGTGGATAAAATAAAGTCGTTGGTCGATGCAAATCGTCGAATAACAACTCGAGAGATTGCTGAAAGATTAAATTTGTCTAACGCGACCGTTCACAAGCACATGAAACGTCTAGGATTAATTTCGAAGCTTGACATATGGGTTCCTCATGTTCTTACAGAACGAAATTTGCTTCGTCGCATTAACGACTGTGATTTGCTTATCAAACGTCAAAGAAGTGATCCATTTTTGAAGCGCATTGTTACTGGCGACGAAAAGTGGGTTGTTTACAAGAATGTAAAGCGCAAGAGATCATGGTCTAAAAAGGATGAACCAGCTCAAACCACTTCGAAGGCCGATAGTCACCAAAAAAAGGTTATGTTGTCTGTTTGGTGGGATTTCAAAGGGATCGTTTATTTTGAGCTTTTGCCCGACAATACCACGATTAATTCTGAAGTGTATTGTGATCAGCTGGACAAATTGAGTGATGCACTCAAACAGAAGAGGCCAGAATTGATCAATAGAAAAGGTGTAGTGTTCCACCAGGATAATGCGAGACCTCATACAAGTTTGATGACTCGCCAAAAGCTTTTGCAGCTTGAATGGGATGTGCTACCACACCCACCATATTCTCCAGACTTGGCACCTTCGGACTATTACTTGTTCCGGTCTCTGCAAATTTTTTGGATGGGAGAACCTTCACCTCAAATCAGGACGTCAAAAACCACTTGGACCAGTTTTTTGCCTGCAAGGATCAAAAATTTTATGAGCGTGGAATCAATCTCTTGCCCGAAAGATGGCAAAAGGTATTGGATCAAAATGGAGAATATATAATTTCATAAAATGTTTTACACAATAAAAAATCGTGTTTAAATTGCACTAAAAAACGAAATTACTTAGTGAACGACCCAATATA

>Btry_5404#LINE/R1

CCCGGTGGGACTTTAAATTTCCAAGGGGAATAGTCACCGTCGCTGAGCTTTAAGCTACCGGGGGGGTCGGCAGGTCGCGGATAGCCGGACGGCCTGTAGTAGCAGGTTAGTTGCGAAATAAGTTTAAACGAATAAGCAATCCCCGACGAGGAGCGGCAGGAGTGGAGGATGCGGTATAAAAGCCAGCAAGACCGTTGAAACTCCTGCGACAGTGGGCGAATCGACACCGCCCCTTTGAAATATGTGTCCCCCGCAACATGCAAGGGCATGTTGTGTAATAAAAAAATCCAGGCGCGACGGACCCATAATGGGCGGCTTCCTGGTCAGCGTCTGTTCACCATGTTAGGTGCGGCTGATATTACAACTTGACCGGTACACAGCGCGGCGCACTGGGAGTCCCTGGATATCTTGACAGCGATGTCTCCGCTGGTAGGATGGAAAGGGTGATGTGAGCTTGCTCTGCCGAGGTGTCAGCTGCGTGGTGTATCAGTAAACAGCCACTTCGGTCCCTGGTCAGGGAGTGTGCATTGGGCGCAGGAGTAGTAGCCTGCGTTGCCCCGGGCGAGCGCCGGTCCGTCCGTGTTTTCCGGGACTGGTAAAGCGAAGGACAGGCCTCAGGGAACTGGGGTAGGAGCATNGTCTCCGAGGGTACACCCGTTCCTGTCTATTTCGGCCCGCCCCCCTACACACGATGCGCTGTAAAAAACAAACAAATGGAGAATAATTTGAGAAAATACACCAAAACCAATAGCAGCAGTGTACCTGACTGCATGATGAGCAACGGCACTCAAAGCCCTGGCAGCATGTTTTTAGAAACTGATTTATCCAGGGAAACGCCGACGATTAGCGGATCTAGCGAAGCGGATCCATTCAAACGCGCACCAAGATTGATGCGATCACCAAAACAGGCGCTAGTTGTTGAAAAACCTGTAAGAGCGAGATCGTCACCACCNGCGCTGCAAGACAGCACGCCTCGAGCGGAACCAACGCAACCGAAGGGTGACATTATGTCTGAATTGGGAGATATGATCAAGAGGCTAACNGAAGCAATGAAACTGCCTCAACGATCGATCAACAATCAGATCCGTGATCTGCTAACTTCTGTCACAAAGCTCCACGAACGCGCCCAAAGCGAGTACAACAAAATCAAGGAAAGCAGACGAAATGTCCTTTTAAGACACGCAGGAGTGGATTCAACGCCAAAAAGGCCCCGCGAGGATGAGCAGCTGACGCGAAAGACCCCGCCAAAAAAGAAAGCCAGCCAAGAGGAAGTCCAAAAGAGGACCACTGCGTGCAAGAGGAATGAAGGAAACATTCCTACAAAAGAACGGAAGGCAGAGGAACAGGNAAATGAATGGACACAGGTGAAACGAAAAACACAAAACCCGATTAAAAAGGCTGTTTTTGCTCCACGTCCGAGGCCCGATGCAATTATCATTGCGCGCACTGGAAACATGTCGTACAGCGATATGCTTAGAGCCGTCAAAAAGGAAGATGCTCTGAAAGAGCTTGGAGAAAACGTTTCGCGGATCCGGAAGACGGCAAAAGGCGAAATACTTCTAGAAATGAAGAAGGCCCAGATGAAAAACACGGGAGCATATGAAAAGGAGATATGCAAGGTTCTGGGCGACCAGGCGCAAATAAGAGCCCTAACCCACGAAGTATCAGTGGAGATCCGAGACTTGGACGAAATAACCACTAAAGAAGACATAGCGGTGGCAATACGATCACAAATCAAAGAGCTAAATTCCTTTGACGAAAATGCGATCAAAACCATGAGACAGGCGTACGCGGGCACGCAAACGGCAGTCATCAGCCTTCCCGCAGCGGACTCAAAACGGCTGCTTGACGCCCATAAGATCAAGATTGGATGGGTGGTCTGCAGAATAAGGGAAAAGCCGAACCTCAGAAAGTGTTTCAGGTGCCTGGAATACGGCCATCTGGCGAGGGCATGTACGAGCGAGGATGATCGAAGCAAATGTTGTGCTAAGTGTGGTGAAAAGGGGCATTTCGCAAAGGAATGTGATAAAAGCCCGTCGTGTGCAGCATGCAAAAGCAGTGGGAAAAAGGAGACAGGTCACCGGATCGGAAGTTGGAAGTGTCCTCTATATCAAGCGGCGTGTAAAAAGACAAAATGAGAGTTGTCCAAATCAACCTAAATCATTGTGAAGCTGCTCAAGAGCTACTGAAGCAAACAATCTTCGAGGAAAAGATAGATGTGGCGATAGTGTGTGAGCAGTATAAAAACATAAACGCTGGCACGTGGATCTCCGACAAGGAAAACAAAGCTGCCATATGGGCCTGTGGGGGCAACGCCTTCCAAGATAAGCCGCTCCTGGGNAGATCTTACTATACGCGAGCGAAAATAGGTGGCATTTATTTTTACAGCTGCTATATTCCCCCGAGTGTGCTGCAAGGAGATTACGAAAGAATTCTCGATGAACTAGTCCGAGACGCCCTCACTACTACGCCAAACGTGATAGCAGGCGATTTCAACGCATGGGCAATTGAATGGGGTAGTAGCTACACAAACAGGCGGGGTAATGCTCTACTCAAAGCGTTTTCTATGTTAGACGCAGTGCTGCTCAATACCGGCGGTAGAAATACTTTTGAAAAGAATGGACGCGGATCGATAATAGATATTACATTCGCCAGCAGCACGCTGGTTCGATCTGCAAATTGGAAAGTGTGCGACTTTTACACACATAGCGATCACCTGGCCATCTTGCTGGAAATCCGCAAGCAAACACAAACCCGATCCAGCGTAAAAAAATTGCAGAAAAAAGGATGGAAGGTGGAAACGTTCGATGAAGAAATTTTCAAACTTATGTTGGATGGGAACCTAGATGATGCGATCAATGTTGACCGACAGGCTGAAAAACTAGTGAAGCACATAAGTAAAGCCTGTGATGCTGCTATGTGTAGAAAAAGGCACAACATAACCCAAAGGCCCGCATACTGGTGGAACGAGGAGATCAGCACATTAAGAAGCGAATGCCATAAAGCGAGACGCCTCTGCCAAAGAAGCCAAGGTACGCCACACCATACAAGACTGCGAGAAAGTTTCAAAAAAAAACGTAAGGAACTCAAGAAAGCTATCAAAAGAAGTAAGTCCTCAAGTTTTAAAGAACTATGTGAAAAGGTGGACGAGAATCCGTGGGGTGACGCGTATAAAATAGTGATGGCGAAAATCAGAGGGGGTAAAAGTCAAGCACCAACCTGTCCGACCCTCCTAGAAAAGGTGGTGAAAACGCTTTTCCCAACGCAAGAACCCCAATCGAGTAGAACGATACCAACCGTGATGGAATCTCCAATTGTCGACGAGAAGGAGGTAATGGAAGTGGCGGAGAGATTTGGCAACGCAAAGGCACCAGGACTTGATGGCATCCCGAACAAGGCACTGAAAATTGCCATCAACCATAGCAAGAGGGCCTTCGCCGAACTCTACTCACGATGCCTAAAAGAAGGAGTTTTCCCAAAAATTTGGAAAAAACAGCGGCTGGTTCTTCTTCAGAAGCCAAACAAACCAGCGGGAGAGCCGAGTGCGTATCGCCCACTCTGCATGATCGACACGATGGCCAAAATACTGGAAAGACTGATTTGCACACGTCTAGAACGTCACCTAGAAGAAGAGTCTCCTGGCTTATCTGAAAACCAATTTGGATTCAGAAGACATCGGTCCACTTTGGACGCAGTTGGAAAAGTGACGGACATTGCGGCCAAGGCCATAGAGGGCACCAGATGGATGCATGGNGATAAAAAATACTGCGCAGTTGTCACGCTTGACGTGAAAAACGCTTTTAACTCTGCATCCTGGCCACATATACTGAGGGCATTAGAGGCAAGAAACACCCCGGGGTATCTTCTGAGGACAATTAGCAGCTATCTGTCTGACAGACTACTGCTGTATGATACCGATGCAGGTCCAAAAACCTACATGGTGACAGGTGGAGTACCGCAGGGCTCCGTATTGGGTCCGTTACTTTGGAATGTGCTATACGACGGGGTGTTACGCCTACCGCTGCCAAAAGAGGCACAAATAATTGGCTATGCGGACGACATTGCTGTGACGATAGTTGCTAAAGAGCTCTCCCAAATTCAAAACTTCTGCAATGCGACCACAGCAAAAATTAAGGACTGGCTCACGGAAACTAAGCTGCAGCTGGCCGGAGAAAAGACGGAAGCGGTGCTTATCACGAGCAGGAAAAAACTAGAAACGGTCACCTTAAATATCGATGGATACAACATTACAACGCAAAACTCCCTGAGATACCTTGGCGTTATGATTGATACGAGATTGAATTTCAAAGCGCATGTTGAAAAAGCCTGTAGCAAGGCAGCTACTGTTACAGCGGCCCTGTCAAGAATAATGGCTAACACTGGTGGCCCTAGTCAAAGTAGGAGAGCCCTACTAGCTAAAGTAAGTCAGTCAACACTGATGTATGCGGCCCCTATATGGGGAAAAGCACTGCGCCAAAAAACAAATGCCAATAAGGCAAAGGCAGTGACCAGGCTATGCGCCATCAGAGTGGCCTGTGCATTCAGAACGGTCTCACATGAAGCGGCCAGTATCATATCTGGTCTTATGCCTCCGGACTTAATGGCCTCAGAGTTACGTAGAGTCTACCTAAAATCTAAAGCTATGAATAGGCGTCTAACAGCTGACGAGCGAAAAAAGGAGAGACAGGTTAGCCTCGAAGAGTGGCAAAGACAATGGAACGCGGCAGAAAAAGGAAGATGGACGCACAAGCTCATTAAGAACGTATCAGCTTGGATTGAGAGACAACATGGGGAGACAGACTTTTATCTGACGCAATTCTTGACAGGCCATGGCTGTTTCAGGGAATACCTTTACAGATTCGGCCACGATGATGACACAAAATGTTCATTCTGCAGCAGTGCCAANGAAAACGCCGAGCATATCTTTTTCTTCTGTTCGAGATACGNAGTGGAGAGAACTACCATCGAAGGAATAATAAACCAAAGGATGACGCCTGACAACATTATAAGCCACATGCTGCAATCAAAGTTGGTATGGGCGAAAATGAAAGAGTGGGCCGCAATAGCGATCCAAGAACTCAGGATAAGAGAACGACAGCGAAGAAGCTTGAGACCAGCTGAATAACCACGAAAGGAGTTTCTGAAGAGGCATTCTTGCCCAGCTTGGTCCTGCGAAGTAATGCCTAACGGCGGTCCCGCAGGTCCAAATACAAGCTAAAGCAGCCGGAGTTAAGGGTTTAGTACGTAGGCGTACTGTTTCGCAAGTCCAGTAAAGTTAGATAATACTAACTGGGCGTGATCCCGAAAGAGGTGTATCTTTAGCGGGCAATATGAATCGTGCATGCTACCTGTCTAAGGCAGTATCTTTAGAAGATTCCCCCTCCGGAAACAAAAAAAAAAAAAA

>Btry_2674_TIR#unknown

ATATGTACATTAGGGTGGAGCGATTTTATTAATTTTTGATTTCTGTTTCGGTATACCTGTAGAAAGTTGCTGTTTTAGGTAAATATTAAACACAAGAAAAATCAGTAATCTCATGCTATGTTTAGAGGTGCCCCCATCGACTTGAAATTTTGAAAATTTATGCCAAATACTCATAAATTCGCAAACATTTTATAAACGTTATTTGATGTTCTAAGCGATGCTATGTTAGAGTAAGATAGAAATTAATGCTCTTAAAACATATATATCACTATCTAACCATATTATAATAACATAGCTACGTTCCGCACTTTAATTTCACATTCCTAATTTCACACTCCAAAACTTCCCCCATTCTATATGTTATGCTAATTTTCCCAGGCACTCACTTTCCACAATACCAGGCCGACAATATCCAAATAACGGTAGATTTAAGTAAGCAACTGACTGCGATCTGTCAATTCTGTACAGTCTTAAGTTTGAAGTCGAATAAAGTGGTTTGATTTAAAAAAAAAAAAAAACCTAAGTTGATACCTATACTCTTTAGTCGCGCGCTTACAGCAGTTCACTTGTTCGCCTTTCATTTATTTGTTACATCAAATTTTACAGCTGGAATCGGAATATTTACTGTAAATCGTATTTTTCATGTATATTTGATGCTTGTAGCTTAGCTTATAACTGTAATTAGTACTTATTACTTGTCTTGATTGTAGAAATATGTCTCAAGTTAAAGCACGACAAACGACGAGGTGTCCTGTCTCTGGTTTGCCGGAGAAGTTACCTGTTTCTCACTTACCTACGTATAATGATCTGATGAAATGTTATCTGTTCATAAAACATGAATTGAAGCCCGATAAAACAACAATGGAGCCAACAATTCTTGAAATATCTGAAAGATTAGCGGGTGAAGTTCTAAAAATATGGCAAAAATCTTCACTGCCAACTGTAAGTGTAAAAAGAATTTTACAATTGATTCGTTCCTACCACGACATATACAGGAGCTTAATGAAACCATATCAAGGCCGTCAAACCAACATTAAATACCAAGAAAATATTAGAACATTTATAACAGAGTCCCATAGATTATTTAATATCTGCAGCTGCAAGTGCAAAGATATCAGGAATTATTGCTGTTTAAAAGGGTGTCGTGTTGCCAAGGAGGAAGTAAGTTTCTTACTCGACCAAAGATCAACCAGAAAAATGATGATAGGCGGTGTCGATATAGTTTCAACTGTGAAGAATAAGAAAAAACATCGGAACCAGGAAACAACTATTTTGGTCATGTCACCTGTGAGTCGGGGACTGCTAAAAATATAAAAGACACTATATTATTATATTTGAAATCTAAGTCGGTAAATGTGAGTGAATTAGCTTTGTTGGGTGCGTTGGAACTGTAGTGGATACAGGTTTCAAAGGTGGTGTTATTCGTCTGATGGAGGAGGAATTCAACAGGTCATTAACAGTGGTTTATCTGTCAATTGCATTCAAATGAGTTACCACTACGTCATCTTTTATTACATTTAGACGGAAAAACTATCGGACCTAAATGCTTTTAAGGCGCCATCGGATCTCAATTATAAACTTGTCAAACAATGCCGATAGTCGAATTTACCGTTATACCTACATTATTACCTGAAATAACTAGGGAAGAGCTTAATACCGACCAAAAGTATTTATACGACATAATGACCGCTATCAGTACTGGTATATTTTCTCCTGTTTAGCTAATATGGAAGCTGGAGCCCTTAACCATTCTAGATGGCTAACAACAGCTAATCGAATTCTAAAATTGTATGCCACTAAAACTGACCCAGAAGAAAAGCTAGTTGTATTAGCCACGTACATATGAAAGTTTATGGACCAATGTGGTTTACAATAAAATGTAATTCTTCATACATTAACGGAGCCAAAAACATATGGCAAACCATTAACCTAAGTCGTTATTTGAATTCCGATATGAAAGCAATCGTTGACAAAGTAATCCAACGAAATGGATACTTTGGTCACCAAAATCTTCTCATGCATGCTTGGTGATGACAGAGAGATTATAAGAGATCTTGCATATCGAAGAATTGTAAAAGCAAGATCAGAGAATGCTCGAAGACTTCGAACGTTTAAAGTACCTACATTCAACTTCGATGCCAAAGATTACACTGAGATTATTATGTGGCAAGACTGTGATATAACTGATCCACCTCTAACTTTAAATATGTCTGATGAAACCTTGAAAGACATTGTCAACCGTTCTTGGTACATGTAAGGATATTACAGATTTTCTATGCCACACCCAAGCTGTGGAGCGCTGCATCAAGTTGGTAACAGAAGCATCGAGTGCAGTTTGTGGGGAAAATAAACGAGATGGTTTCATTAGGGCAAGACTGCTATCACGTCAACGAATGCCTCATTTCAATACAAAAAAACAGTTTGATGTTTAAATAGTATTTTAAGTAATGTAAGAGAATATTTTTCGTAATAAATGCTTAGAATGCCATTTTTTGTTTTATTTAAATGTTTGACTGTACTGGGGCACCTCAAAACATTAAGCCAGCTGTTTGATATTTTGCATGTTTGTTTTATATATTAAAAGGCAACTTTTCATAGCTATGCCGTTAAAAAACAGAAAAAAAATTTTCTCCATACATTATCGCTCCACCTTAATATA

>Btry_4124#LTR/Gypsy

TGTAATGTGACAATAAACGCCACATTATGGCAACCCTGTAATGGTTGTTGGCTGTCACATGAATTCAATTCAGCGTTAACTGCTTAATAAGACACACGTGTATAGAGACTTAAAAATATAGCGTCGATTATTCATTTCTACAAAATACATTGTTTTAATTCAAGTCCCGGCATATTACATGGTGTCAGAAGTAAAAAGATCTATTTTATTCTAAAATAAAAAAATAAACATAATTTGAACGCGGAATTTGGGAATAATTCTAAACTTTATGAATTTCATCATCGGGCCATTGTATTGCGGCGTGCATTGGAATATACAGTTAACGTTGTCAAGCAAACAACAATTTCATCGGAAAAAAATTGATAATGGCGGATACACGAAAACCATTGCGAGAATTGGATTGTGCGAATATATCATCGGATTGGAAGAACTGGAAACGTGATTTCCAAGTCTACATGATGGCAAACGACAAGGACAGCGTAGCTGAACAGAAAAAATATCAATTTTTCTGTGGTTGATTGGGACCAAAGGTGCTAACATTTACAACACTTTGTTTCCAAATGATGGGTCTCAAAATGCATTGTTGGGTACAATCACGACAAAAAGACATGTACCAGCAGCAGGCAATGTAGCAGCGCATGATGTGGATGAAACAAAACAACGCACATTGTGTGAAGTGATGAAAGCATTCGATGAACATTGTCTGCCGCAAAAGAATTTGACAATGGAATCGTACAAATTTAACAACATGGTGCAAAAAGAACATCAACCGTTCAGTGAATTCTTAACCGAACTGCGAACTCAAATCGATCGTTGTGAATTCAATTGCACTTGCAAATTATCGTACGAAGATCGAATGTTACGTGATCGTATCATCACTGGGGTTTTTGACAAGAAACTTCAGCTAAAATTACTCGATGGACGGGATGAAAAATTGGATCGTGTAATTGATGTGTGCAAGACATTTGAAGCAGCGAAAGTGAACAAGGGTATTTTAACGTCAAAGCATTCGATTTCAGCTGTGGTAGATGGTGAGACTGACTCAGTCAACGCAGTCAATTACAATCGGCGATTTTGTTTCAACTGTGGTGGTGATTGGAAGCCGGATCACAAAAGTAAATGTCCAGCCAAGGATTTCACATGCCAATGCGGTAAAAAGGGTCATTTTCGCAAGATGTGTCGTAGAAAGGGAACCAGATCGGACGCAAGTACATCGGAATCCAAATCAAAGGAAAAACAACGGGTTGACAATGTTAGCTGGGCTGATTTGTCAGGTAAGGCGTCGACAATACACCGAAATATAAAAAATAAGTTACTTAGTATTAAAAATACAAATTTTAGGATAAATTCATTCGGACGAAATAAAAAAAATTGGACTAAGACATACCAAATAGGGAGTAGACTAGTTAAATTTAAAATTGATACAGGAGCGGATGTCAACTGCGTTCCATTAAGTATTGTCAAGGAAATGAAAATCAAAATGAGCAATGAACAAAATGACTTCCCTGTATTCGATTATAGCAATAATAAAGTTAAAATTTTTGGTATTGTCGAAATTAAACTTATGGATGTAAAATCTCATTTTGAGCGAATAGTAGATTTTGTTGTTGTTGATAGTACATTTGAACCGATACTGGGTCTTGAAACATGCATTGAACTTGGATTAATTAAAAGACTGGATATCGAATCGATTCGATTCTCGGATGAAAGGGACCAGTTTGTGAGTAAACATAACGATGTTTTCACTGGACTTGGAAAAATTCCTGGGCAAATAACGATACATTTGCAGGAAAATTCAAAACCTGTTGTCCATTATCGAAAGCGTTTTCCGGATACAATAGTTAAAAAACTTAAACCAAAGCTTGATGAGATGGTTGATAAAGGAATCATTTCACCGGTATCTCACCCAACTGACTGGGTCAATAATTTGCAAGTGGTAGAAAAATCAAACGGTGATTTGCGGATTTGTTTAGATCCAAAACCACTTAATAAGTGCCTTAGGCGCGAACATTTTTTGATTCCAACTATTGACAATCTCACTTCGGAATTAGCAAATAAAAAAATATTCACTGTTTTGGATTGCTCAAACGGGTTTTGGCAATTAGAGTTGGATGAAAGGAGTTCCGAGTTAACAACATTCATGACACCTTTTGGTCGATACAAATTCAAAAGACTTCCTTTTGGGTTGAACATTTCTCCGGAAATTTTCCAAAGACGCATGATTCAAATTTTTGGTGATATTCCTGGAGTCTTTGTTTATTTTGATGATATTGGAATCATGGCGTCAGATGAGGGGGAACATGATAAAATCTTGGCAATTGTTCTTGAGCGTGCACGTATACATAATGTTAAATTCAACCCGGAAAAAATTCAGTATCGTAAATCAGAAGTAAAATTTATGGGCCACATTTTATCGAGCGGTAAGATTCAACCGGTTCACAAGCACATTGAAGCTATACTTAAAATGAAGAAACCAACTGATAAACATGGTGTAATGCGTTTGTTGGGTCTATGCAAGTATCTGGCAAAGTTTATTCCGAATCTATCAAAACTCACGGCAGAATTGAGAAAATTAACCGGAAAAGATGTTTGTTTGAGTGGACTAAAAAACACGACGCTGAACTCGATAAGTTATTATTGATTATCACATCAGACCCGGTTTTAAAAACATTTGATTCAGAGAAACCGATTGTCATTCAGACAGATGCNTCAAAAGATGGACTTGGATCGTCCCTATTGCAAGACAGTCACCCAATTGCTTTTGCATCTAGGACCCTAAGCAAAAGTGAACAGAGGTGGGCACAAATTGAAAAGGAACTTTTGGCCGTGGTTTTTGCATGCACACGTTTTAAAAATTTCATTTATGGACGCGAGTTCATAGTTGAAAGTGATCACAAACCACTTGAAACATTAGACTTGCGTGATATCGATGGTGTCACACCACGATTGCAAAGAATGTTCATGGTATTATTAAGATATCCGAAAATGAAAATCATTTATAAACCCGGAAAAAGTATGTTGTTGGCTGATTGTCTTTCTCGTGCACAGTTGGACGTGATCTCAGGAGGGGAGGAGGATGATGAGATTAGAGATGAAATGAGTGCAGTTATTCACCGGATTACAAAATCGGCTTGCGTGTCAAAAGACAATCTCGAATGCTTAAAGCGGTCATTAAATTCGGATGAAAACCTCAAACATGTGTGTAAATACGTAACAAACGGATGGCCAGAATATCGAATCCTGAATGATTTTTGCCGAAAGTTTCATAAGGTGAAATCAGAACTACACTTTGAAAATGGTTTATTGTTTAAGGACCATAAATTGGTGATTCCAACTGGGTTGCGGGGAAAAATGGTCAAGTGGTTGCATGAACCACATCTCGGGATTGAAAAAACACTTGCGAGGGCAAGAATGTTATACTTTTGGCCAGGAATGAGCACTCAAATTAAAGAAATGATTGCATCATGCAACATTTGTGAGAAATTCAAACGGAACAATCAAAAAGAACCATTGGTTCAGGAAATAAATCCCAAATATCCGTATCATATTGCATCCGTGGATTTATTTGAACATGCCGGTCGTGACTATATCTCAATATTGGACGCATATTCCAATTTTTTGGTAGCGACCAGGGTCAACAACAAAACTTCTGGACATTTAATTGAAGTATTTTGTACCATTTTTAACACGATCGGATATCCATCGATCATTAAAAGTGACAATGTTCCGTTCAACTCGTCGGAATTTGAAAAATTTTCACGGGATTTTAATGTCAAATTTAAATACTCAAGTCCAAGATATCCACAAAGCAATGGTTTGGCTGAGAAGGGAGTGGCAATTGCCAAGAATATTTTGAAACGTTGCTATGAGGCAAATGAAGTAAAACAATTTCAATACAGAATCATGGAATATAATACTACGCCGGTCGCAAGCATGCATTTTACTCCATCGGAATTATTCTTTGGTAGGCTGATTAAAACGAAACTGCCAGTGTCGGAACAATTGCTTGTCAGAAACAATGTCAAAGAGGAGGCTATTCAAGAAAAAATCGATAAAAAAAAAAAAAACCAAAAAAATTACTATGATCGAAATGTGAAGTCACTGCCATTATTGAAGGTGGGA

>Btry_1290#LINE/LOA

CTAAAAAGCTTGAACGTGTCCAAAGAGCGGCTCTCATCGGCATCTCCGGTGCACTACGGACTACACCAACAATAGCACTTAATGCCATTTTAAACATAGCACCCGTGGACATTGCCGGCAGGTGTATTGCTGCGAAGTGTGCTCTAAGGCTCAGGGAAGCTGGGTATATGAAGGGGTCCGAACAAGGACACGCCGAAATTCTCTCCTCCTTCAACTGCATTCCTGAAAACTTGGATCACTGCGTCGCAGAGGCCACTCGTGGCGGCTTTTTCTCTGCTCACATACCGACGAGGGATATGTGGATGGGGAGAAGTCGCTGGAGAAGAGGCGCAGTGAGCTTTTTCACGGATGGGTCGAAGCTAGGAGGGAAGGTTGGAGGGGGAGTTTTCTGTAAGGAGCTCTCCGTCAATCTTAGCTTTAGGCTACCGGACCACTGTAGTGTTTTTCAGGCAGAAGTGGCTGCTATTAAGGTGGCGGTAGACGTACTGCTACGAAGTGCAGCCTCTTTCAGGGAGGTGAGCATTCACTCCGACAGCAGAGCGGCAATACTAGCGCTGAGCTCGCTAACTGTGCGCTCAAAGCTAGTCAAGGAGTGTCTGTCCTCACTAGAAATAGCATCAAGCTACTTCATCATCAGACTCGTTTGGGTGCCTGGTCATAGCGGAATCGCTGGCAACTGCAAAGCCGATGAGCTAGCCAGAGGGGGCACCCTTGCCCCGCTCACATCGGAATGGGACCGAGTTGGATCTCCATTGGCGTCTTGCGTTCTAGCACTGGACCAATGGACCTCGCGTGCGCTTAGCAGGCGCTGGTCGACGACCAGCTTCTGTGCGACTGCGAGATCCTTTTGGCCTAGAGTAGAACGCAGGAGATCTACTGAACTACTTGCCCTTAGCAAAGTTCATCTTGCCGCAATCGTAGGAGTTCTTACTGGACATTGTCCAATAGGCATTCATGCGGTAAGGCTAAAAATCTTGTCGGACGCAAGTTGTCAAAGTTGTATGGAGGAAGGTGAGGTGGAAACATCCAGGCACTTTCTCCTCCATTGTCCAGCCTTTGCAAGACTGAGGTTGAAACATCTCGGCAGTCTTACCTTCGGCGAACCAGAAGACATAGCCGAAACTGACATTAGCCGTCTCAACAAATTTGTGATAGGCTCAAAGCGCTTTGTCGATTTGTAAGGGTCTTATGATCTATTATATAGGAGTTTTAGGTACCACAACGGACCGGCGTTCTAAGCTGTCCAAGTGAGATCCCTGTTAGGATCGACCTCTTAACCTAACCTAACCT

>Btry_6376#LTR/Gyspy

TATATATAACATAAAATAAAACAATATGTTTTTACTTGGCAGCACTATGTGCTTATGTTGAGCTTTATTACAAAACTGAAAACTTAATTCTATTGTTGGTTATTATCAAGCTAAGCTCGAGCCCTATAAGTGCCTAGTCATCAAAATTAGGTTTGCGCGTGTTTCGCTCACCTACCTAATAATTGGGAGAGCGGATGCGCGTGTAGCGCATTCCATTAAGCTAAGAGTGCAATAGGAGTCTCAACTGTAAGTTGACTCCTAATTATGCAGACATTGTTGTCAGCGAATTTATGTTGTGTTAACTCCTCCCTCCGAAATTCCAATCGTCCCCGATTGGTCTTTAAAAGTATGGTATGTCGTTAAGACTTTTATGTCTTGCAACTTTCCTTGTCGGCATATGAATCGCCTCCGCGTTTTGAATAGTGATTTTGGCCATTTTCCGTGTCCGAATTTTAAGAGCTATAATTATGATTATAACAAAGACCAAAATACCGAAATGAGTTAGCATACTAGACTTGACTAGTAAATTTAGAGAGCTAATTTTTTTTATATTTTTAATATTAAGCTCTCTCATAAACTTTAGAGATAGTACTTCTTCGTACTCGGATCGAGAATTTGAATTATGGATTAATGCTGGCAATGGTCTCAGGCTGTGAGCTTCTAAGGCAGTAAAATTTAAATTGTTAATTTTTATTGTTTCATTAAAAAATTTTACAATAAATGATCCATTTAAATGTCTCGTTACATTATTACTTATAGTTAAATTTCCTTGAAAGTTATTTAGTAAAATTAATCCTGCTTTTAGTTCTTCAATGTTTCTTACATGCTCAGTATTTGTAATAATACAATCATAAGGTTTGTTTTCTATCAAATTTTTAATACAATTTTCGTTTTGTAAATCTTCAATTTGGTCTTGTTTGCATATTTTTACATTATTTATTTGTTTACAATCATTTTCTACTCCATAGTAATTTTCATTACATTTCAAAACGTTTTCAAATTTTAATTTCAAAATTGTTCCATTTTTGCTTATTGGTTTTATTAAAATGCAATTACAGATTTCATTCCTTGTTTTTGGTACTTTTATTACATAAATTAGTAAATTTTGTTTCATAGCGATTTGAATATTTGCAAAGTTTAATGATTCTTCTAAAGAATCAAACGGAATGTTTTCTTCAATAAATATGTCATTTATCTTGTGAGCCTCTTCATTCGATATTATGAAACTGTTTATTATGTTTGCTTTGGTAAGATGTATTGCTCTTTCAAAAACGTTTCTATTAATTACTAGCTGTTGATTATTGTTTTCTATGATTTCTTCGAATTTACTATTGATCGCTACAAGGTCATCATGGTCGGGATTTCCCGCAATCCATTTCCATGCTGATCCTATAAAATTTAATGACCTCTTTACTCTATATTTTGGTGTTATATTGTTGATAGTAATATTAGTTATTTTAATTTCTTCTGCAATGAATGGGTGCAAGGGATGGGAAGGTGGGATATTTTTTGTGAAGTGGGTATTTATGTCNTCTAGAATTATGGAGTATTCGGTAAGGTTGATGAAGTGAAGGATCCTGTAAGTTCCAGACTGCACTTTCCCGATGCCATTTTCGATGGTAACGATTTCGGATTGAGAGTAATCGAGTACTTCAACCGAAGCATAGCAAAGGATGTGAAGGGTTACGGAAATTAGTGATCTAAAAGAAGATATGATTATATTAGTTTCTTATACGATCTTTGTGAACTATTTTGCCTGATTCTGTTTTTACAGTGGAATGTCTGTTTTCCTTAACTATTTCCTCTTTGTACCTTTTTGAAAGTTTTGTTCCTAATCTTTTATTCTGTTTAACAAAAATTTTGTCTCCTGTTCTATAGTCAATAGTTTCCTTTCTATATTTGTTATGATATTGCAGGTCCTTTTCCTGTTTCTCTTTTAATTTTTCTAGAACCTTTTTCATAGTTTCTTCTCTGTTAGTGGCATTTGAGGTAGGTCTACCGAAGAATATGTCAACGGGTTTTTTTCCTGTAACAGAATGAACAGAATGGTTGTATTCGTTTACAGACCATTCCATTAATTCGTCGAAATTTTTATCAGAGTGTTCCTGTTTTAAGCATCTCATAATTTCTGATATTGTTGAATGGAACCGTTCTATTTGTCCATTCACCTCGCTTTTATAGGGTGGTGCAGTGAATATTTTTATATTGAATTCGTTTTCTAACATGAATTTTATTGGTTGAACCGAATTCAAAGACGTTTCATTGTCTATAACTACCGTTTCTGGTACGCCAAATTGAAACAGTAGTTCGCGGAGAGGTTCCTTAACGTGTTCTACGGCTCTGGAGCGAAGGATTTTGGTTTGGGCATACTTTGGAAAACTTGTCAATAGAAGTCAGTACTTTATGTTTGTCTGTTGAGAAAATGTCTATGTGAATAGTATGCCCTGAATATTCTGGTAAGGGTGTAGCTCCTAATTTTACATTGTTTGGGTGGCGATCATATTTATTTTCTTTACAAACTTTGCATAATTTTGTTATTTTTTTTATTTTTTTCGACATAGCGGGGAAATAATATTTTTCCAAAATCTGTAGCTTATTTTCTTTTTCGTTTCTGTGAGCACGATTATGTTCCTCGATTATTATTTTATCTTGGGCCTCGGTCTGAGTAATATCTTCAACTACTATTTGTGTGAAACGAATTTTGTAATTTGCGAAATGTAGAGGGTAGATTGATTGGATTTTTAGCATAATGTGCTCTGTGGTTTTTATACCATTGATTACCGATGGATTAAGATATTTCTTTAAAAGCGATTTTAGCGATTCTTCATTGTAGGTTGTTTCAGTTATTAAGTGCCTATGGTACGTAGGGAAAACTATTTCGAATTTGTATGAATTTTTGGAATCTTCTGATATGAAAATTTGGTTCTTGAAGACGTTAATGGGTATTTCTACTGAAGAAATTAAATTTTCGGAAGAGCTGTCACTACTGTGGACAGTCATTGAATTTAAGTTAGCGATTGGTGGTCGTGAGAGTAGGTCTGCGACAATGTTTGTGTTGCCTGGTTTATAAATGATTTCGTGGTTATATTCTTCCAATTGTGCCTTCCATCTCTTCATTTTTGCATTAGTATTTTTGTTGCTGAGAGCAAAGGTCAATGGTTGGTGATCAGTGTAAATCTTAACCTTTTTTGATCCGTATAGGAAGCATTTCAGGGACTGTAGTGCCCAAATTATTGCCAACATTTCTTTTTCATTGGCGGCAAAAGTTTCCTCTGCTCTGCTTAGAGTTCTTGACAGAAAGGATATTGGCCTACCGTCTTGGGAAAGTACGGCTCCTAGAGCGTAATTTGAAGCATCCGTGGTAAGCTCAAAAGCTTTTTCGAAATCTGGATAGTGAAGTATAACATCGTCTGACGTCAAGCAATTTTTCAGGTCTTTAAAAGCTTCTACTTGTTGTGCTGTTAGATTTAAAAGTGTCCTTTTAGACTGATTCTTGGAAACTCTTCCAAGCTCTCCTCTCAGTATTTCAGTCAGGGGTTTTGCAATTTGTGCGTACGCCTTTATAAATCTGCGATAATGTCCGGTCATTCCTAAAAAGGAGCGTAAATCATGGACAGTTTTGGGCCGTGGAAAATTTATTATAGCTTCTATCTTTTTTTGTGTAGTGCGAATTCCATTTTGGCCAACTATTATACCGAGGAACTCGATTTCCTTTTTGATAAATTCACATTTATCGAACTGTACCTTTAAATGCGCTTTTTCGAGAGTGTCAAATATAATTGCAAGATTTCTAAAATGATCTTCTTTACTTTTGCTAAAGATAATTATGTCGTCAACATAAACGTAGCACCGAACGCCGATGTGTTTCCGTAGCACATCATCCAAAGTGCGTTGGAAAATGGCAGGCGCATTTCCCATTGGCAATAGAGAAGGCGGTCTTCTCAATATCTGCTTCGTTGAGAGCAATCTGATGGAATCCACTCTTAAGGTCAATTGTAGAAAAATACTTATTTGATCCTAGATTAGTTAGAATAGTGGTAATATCTGGAATGGGATATTTATCTGGTATGGTATCCTCGTTCAATTTACGATAGTCGATAACCATACGAAATTTTTTTAATCCTGAGGCGTCTTCTTTCTTCGGTACGACCCATACCGGAGAATTGTAGGGAGATTTCGATGGTCGAATAATGCCATCTTTAAGTAATTCCTCAATTTGTTTGTCTATTTCTTTTTTTAAAGGCATTGGATAGGGGTAAGTTTTGGAATATATTGGTCTATCGCTTTTAGTCCTAATTTCGGCTTTAACGTTCGTGACATATGTTAATTTTTTATCTGGTTCAGCAAATAATGTTGGGCAAGAGTTAAAGAGTTGTTTTAATCTTTCGTTATCTTCGGGACTCAAATGATTTAGCCTTAATGTAGTGTGTGCAATAGAGTTAATTGCTAACTGGTGGATGGGGTATTCATATTTATTGAGTAATGTAAAATAGTTCTCCTTAGTAAAAATTACTGCGTTTAAACTTTTCATTGTATCGTTACCTATTATGGCATCAAAGGTTCTTAACTTGGGCAAAATGTGAAATTTTAATTTACCTACTTCATCACCGAAAACACTGATATTAGCATGATGAGTAATTTTGACGGGTCCACCAACAGAATTTGCAATGAAGGGTTTGTTCGGAATGGGGTTCCTAATATAAGTTGGACTAATATAATTTTTGTTCGATCCGGTATCTAATAAAAATTTTAGTTCATTGCCATTTTTCATTTTGAAACGATAGTATGGCAATGAGGAGTTTAGTCTAAAAAATTGATATTGTCACTAGGGGTTGCTTCGATCTCTTCTTGTTCTTCGTTGTATTCGGGTTGGTAGTCCTGTGAAGACTCGTAGTACCCTTCAGGATATTCTAATATTTCATTATTGTATTCTATTGGGTCAGTTTCTGTAACTTCAGTATTGTAGATCCGTTGCATTTTATTAGGAGTCTGTATTATCTGTGATGGTGGTCTCTTACTAGCTTGATTATTATTAGCCTGCGCTGGTCCATTACTAAATGCTTGTGGTTTGTTACTGAATGTTTGCTGCCCCGGATTATGAAGTCCTTGGGGTTGTGGCCTATTCTGATAATTTACCTGTTTGGTTTGTATGGACTTGTCAACGTCCATTGGGACTGGGGGTTTGGGTGGTAGTTGTGGTCTGTAACCCTGTTGAGAATTTCCGTAAGGCGTATTGTACGATGTGACGTATCGATTTGCTTGTGGTGTGCCGTACTGTTGATACGAATTTGGTCTAAGGGATTGGTTGTGAACATTTGTAAGTTCTGGGTAAAAGGGTCTTACTTGGGGTTTGAATGAGTATGACCTTGGTTGATTGTGGCTAATAGGTTTGGTATTGGTCGGCAAGCGACTGATACTATTTGTATGGTTCGTCCTGTAATTCATGTTGCCTAATTTGAGACACTTAACAGACTGGGTAAGTTACCGTTCAGACCTCTTATAAATGTGTCGAGTGCCTTTTCTCTATATGTCGTTGTCATGGCATACAAAGCTTCTTCACCTAACTCCAGGCAATCGATTTTGTCTAGGATAAGCGAGAGATATTGATAAACTTGTTGATAAAATTCTTCAACAGTGCTACCCCGTTGTGCAAGTGTTGTCATTTGATATTCCAACGTGCTAACGTCTCTCTTGTCTGAGTAATGTAACATCAGACATTTTTTTATCTTACCCCAATTTAATGGGGTGCGGTAAGACTCAAGGGCCGTGTCAGCTTCGCCAACTATCTTGTGTCTTATTGTATGCAGGATAGCAAAATATTTTGGGGTGTTCTTATTCTGTCCGTACATTAATAATATTCTATCTACCGATTTCCGCCATGAACTGAATTCTCCGTTTTTACCCGAAAATTCTCTTAAACATTTTACCACATCCGGAACTCTTTCGGATGCGTCCACGTTAATGCTAAAATCTACAGGATGTTCAACTTCTTCTTCGATTTGTGGGTGTAATATGGTCGAAACTGTTTCCGATATTAACGAAACGAGTTCTCTTTTTTGATTAGGAGTGAAAGTGGAAGGGATGTTAGGAGGGGAAACGTTAGTGCTGGATGAGTGCAGATTTAATCTCGACAATGTCGATGCTATTTGTTCTTCCTGACTCATTTTTGATTCACTACCTTTCTTTTACGTTTGAAAGATGAAAAATATGAACCTTTTCTTTATAATTTATTAATATTATTTCTTCTAAAGTTTATCACTTGTTTACGAACTTAAATAAGCCTTATTATATAAAAATTTTATTTTTAATAATTTTTTTCTTATTTTATCTATTTAGTAAAAAATCATTTTATTGAACACTGACTTACATTAATCCTAGCCGCTGCATTATGTCCTTAAATTGCTGAGGTTGATTGCTGGTTAACAATGCTGTTGAGGCTGATAAGTAGTAAATTTGTTGTCAGCACTGGGTCGATGAATGTTGATTGGTGGTTAACAACGCTGCTGGGGCTGATAAGTAGTAAATGGATTGATGATGTTGTTGTTTATTCGTTGCTCTTTAGTTTTATATTTTGATTTTTTTTCTTTTGAACTTATTTGCCTTATTTGCTCTTGGCACTTATTTGTTGATTCCTTTAGCACTGTTTGGTAGTCTATTTAAATTGTTAGTCCGACATTTGCAGTCTTAGAAATATTTTATTATTCTTTTATTTTTATTTTATGGTCTAATTTCATTGTAATTTTATTTGTTGCTCTTATTTATTTAAAAACTTTTTTTTTCACACGATTTTGATGTAGAATTTTTAATTTGTTGTTATTTTCCACTTTAAACTTTTTAAGTCCATTTAATTCTTATTGATTCTGTTAGTATTCTGGTTGGCGGGTCCGTACGGACCGTCCTTTTCGTAAACAAGGATTATGTTATTACTAAGGGGCCAACTACCAGAAGTTGTTTCGTCCGCGAATAGAAAACGCGAATTTATTAGTTAATTCAAACTAACTTTCGCGGTCCCTGCTCGGGCGCCACATAAAATAAAACAATGCGTTTTTATAAGCAGCACTATGTGCTTCTGTTGAGCTTTATTACAAAACTGAAAACTTAATTCTATTGTTAGTTATTATNAAGCTAAGCTCGAGCCCTATAAGTGCCTAGTCATCAAAATTAGGTTTGCGCGTGTTTCGCTCAACTACCTAATAATTGGGAGAGCGGATGCGCGTGTAGCGCATTCCATTAAGCTAAGAGTGCAATAGGAGTCTCAATTGTAAGTTGACTCCTAATTATGCAGACATTGTTGTCAGCGAATTTATGTTGTGTTAACTTATATATTA

>Btry_MarQ#TcMarMariner

GTCTTTTTCAGTATATGTCTTTTGAAGCTTAAACGACAATATTTTTTTTTACTCAAATATNTCGAAATTTGTGCCTAAAAAGGTTTTGGGGTCTTCTTCATTACTTTAATATGAAGAAAACCTCAGCCGAAAGTCATCGTATTTTGGTGGAAGTTTATGGTGAACATGCTCTAGCTGAGCGAATGTGCCAAAAGTGGTTTGCACGATTTAAAAGTGGTGATTTTGGCTTGGAAGACGAAGAACGTCCTGGGCGGCCAAAAAAGTTTGAAGATGAGGAACTGGAAGCATTACTCGATGAAGATTGTTGCCAAACACAAGAAGAGCTCGCAGAATCTTTGGGAGTCACTCAAGCAGCCATTTCAAAACGTTTAAAAGCAGCCGGATACATTCAAAAGCAAGGAAATCGGCGTTCGAACGGACTGAAGCCAAGAGACGTTGAAAGACGATTTTGCATGTCAGAAATGCTGCTTGAACGCTCAAAAGAAGTCGTTTTTGCATCGGATTGTGACTGGTGATGAAAAATGGATCCATTACGACAACCCTAAACGCAAAAAATCATATGTGAAACCTGGCCAACCAGCCAAATCAACGGCAAAGCCGAATATCCATGACGCCAAGGTAATGCTCTGTATTTGGTGGGATCAGAAGGGCGTGCTGTATTATGAGCTTCTAAAACCGGGTGAAACCATTAATGGGGAACGCTACCGACAACAACTCATCAAATTGAAGCGAGCGATTGCCGAAAAACGCCCGGAATTTGCGACTAGACACGAGGCAATAATTTTCCACGTCGCAAGACCGGTTAAAAACTATTTGGAAAACAGCGGCTGGGAAGTTTTGCCTCACCCGCCTTATAGCCCAGACCTTGCCCCTTCTGACTACCATTTGTTCCGGTCGATGCAGAACGCTCTCACTGGAATACGGTTCACAT

>Btry_547sTIR#DNA

ACTTATGCCCGGTTTCACAGTCCATACTTAAGTTGTGCCTAAATAAAATCTTAGCTAAGTATTGTTGCAAACTGAGTGGTCGTTTGCGTTTCACAGTCAATGCTCAATTTCTATAAAATTTTATTATGATATATGGCAACGTTATGGGCAACATATGTTTTACAAATGTCATTCATAAAAATATGTTTGAAATATTTAAATTATAACAGACAATACATAAATTTGCGAGGAAAATTATATCAAAAATTGATGAAAACAACAAAAGAACCCCAAATTTCATCACTAGCGAGTCGGAGCACCTATGGGAACTGGTGGAAATCCCAATCCGATTCCATATTTTTTCACAAAAAATAATTTCTTATTTCTTATTATTCTTCTTTTGACAAATAACTGAAAATTCAAAACAAAAATCAGCTGTTATTTAAGCACTGCTTAAGTCTCCCATTTTCAGTACTTAAGCATAACTTAAGTATGAACTGTAAAACAATATTTTCCTGTTTTATCTTAAGTACAACATAAGTATGGACTGTGAAACCGGGCATTAAAG

>Btry_726sTIR#DNA

CTTAAGGGGGGAGATACCTCTAGAATTTTCAAAAAATTAAAAAAAAATTTTTCGCTCTAATCAATTGAAAAACTATTCAAAAATATAGTCCCGAAGTTTCATTGGTTTACTCCGAATATTTTCGAAGAAATAGGCCCCAGAACCACTGCACTTCATGCAGCGGCTGTATAGGTACCAAAACTTTGAGGCTCGTTTCCTCGAAACTAACTTTTTTTATCCGGCCGTCAAAATTGCGAGCGAATTATTTAACCGATTTACTTGAACTAAAGCGTATTTTATAGACAATTTTATACTCAAGTGCGTGATAGAGCGCTTTTCCGAGAAAAATGGCATTTTGACAGTTGTTTAACTATTTTGATGTCGAAAAACACAGGTAAAAATGGCACTTTTTACAAAATCCAGGCCTACATCCCCAATTTTTCGAAATTCTCAAAACCCCTCTACTATGCACTTGATAAAAGCTTCCAGATTAAAAAAAAATTTACTTTTTTCGTTTCAGATAAGAAGCCTAGCCAGGAAGTTGACGGCCGCAAAAAATGTCAATCAACGGGATCGTTGATATTTCCGCCATTTTGTTTTTTTTGACATAAAATAATTTTTATTTTGTAGTTCAATAATACCTCTTAATATATATGCAAAAAATCGGATGTGATCTGTTTTTATTCTCCCATAATAAAAGTGGAAAAAATTACCTTTTTTGACGGTACTAGAGGTATCCCCCCCTTAAG

>Btry_1322#unknown

AATTCTGTGAGGCCAAACGTTGAGAACGTTCAGCGCTTGATTCCCGATCACGGCTAGCCAAAGTTCGGGAATTCTGTGAGGCCAAACGTTGAGATCGTTCAGCGCTTGATTCCCGTTGTCGGTTCGCCAAAGTTCGGAAATTTTGTGAGGCGAGACGCTGCGAACGTTCAACGCTCGATTCCCTAGCACGGTTTTGTTCACTGATTGCTCGTTGAGTACCCAACCTATGCATATTCTCATCCTCAGTTTCATTGCTGCGCAAAACTCGCAGTCGCTTTGCAGTAGCAGTCTGCCGAGAAAGCTGAGTTCTTTTTCTAGGCATTATAGTGACAAAGAAAGAACGTAGAAAATGAATAAAAAGAATGTAGAAAAAAGAAATGTAAGATTAATAATCAGGAACCTAATTATTTTTACCTGCACTTGGTATTTACGTAAACTGAATATTATGTCAAAAGAATTACCATAAAACGAAAATAAGGTAAACTAACCTATTTATTTAATTAATAATCTACATGAAAACAAAAAAAGGATTAATTACTATGAAACTATTAGAAATAAAAGTAATATACTAAAACAAAAAAATATCTACTATATAGAAAACAACTACAAAATGAAAATGTGTTTAAAAAGTATTATTACGATGATAATATATCGCAAAAACAAATTAGCGCTGCGTTCGACCGTTCAGTACGGAGCCGACCGCGCGAGTGAGCCGCGCGGCATGGTATCTCGTAGAGCTACACCCCCCTCCGGCGCGCCCGCAAACACCGCTTCTCGCCCGCCGGCCAGCGCAGCGCAGAGTGCGAGTTGATTTTGTTTCGGAGTTATTTATTTGTAATATTATCTTTTAAGTTGTTCGTTGAAATTAAGTGTCGTCAAAGACAATTTTATTCAGAATTAAATACTCTTAACCAACAACACATTTATTTGGATAAAGATTAATATTATTATGTTAATACAACTCTAACAAATAATTTCGACCAAATTAGTTATCACAAAAACGGTACTCACGGACTTTTTTTTAGATCATTAAAAGAGAAATAATTCTTTCATACATAACTTTTGTGTATCTTGAACCGCTTTCGCAGCGCAAGGAACGGAAGCCCTCAAAGATAAATAATTTCCCCCGTTTTTTACACATTTACCACTGTTTCTTCGCTCCTATTGGTCGCAGCGTGATGCTATATAGCCTATAGCCTTCCTCGATAAATGGACTATCCAACACAAAAAGAATTATTCAATTCGAACCAGTAGTTTCGGAGATTAGCGCGTTCAAACAAACAAACAAACTCTTCAGCTTTATAATATTAGTATAGATTT

>Btry_908#DNA/TcMar-Tc1

TATAAAGGGTCTACCAACAAGAACGACGTGATGTTAAAATGAAATAAAACACTCAAATTTGGTCAAAATGGGTTCTGTTTTTTGTTATGCAGATAATTTTATGCCATTTATGATTTGAACAAAATGTCGGCCAAATGGCCACCACGGCTCCGTTTGCATATCTCCACCCGTTTACGCCAATTTTCGATGACCCGGTGCAGCATTTCGGCTATAGTGCGCTGAATGTTGTCTTCGAGCTCCTCGAGCGTTGCTGGTTGATTGGCGTAAACCTTTGATTTAACATACCCCCAGAGAAAATAATCTAGAGGCGTTAAATCGCATGATCTTGGCGGCCATTTGACTGGGCCATTTCTCGAAATTAACGAGTCGCCAAACTTTGCACGCAATGTGTCCATGTTTAGACGTGAAACATGAGGCGGCGCACCGTCTTGTTGGAAATAGAGATCGTCCGCGTCGATTTCATCAAATTCCGGGAAAAAAGTCGGTCAACATCGTGTTATAACGCTCGCTATTGATGGTAACGGCATTTCCTGCCTCATTTCGAAAGAAATAAGGCCCGATGATGCCGCCATACCACAATCCGCACCAAACGGTAAATTTTTGGGGATGCAATTGCGTTTCCTGAACCACACGAGGATTTGACTCACCCAATAGCGACAATTTTGTTTGTTGACGAAGCCATTAAGCCAGAAATGGGCCTCATCTGAGAAGATGATTTTTCTTCGGAGAACGTGAATTTGCGTAATAATCTTGGACAATTTCCAAGCGTTGTACCAAAGTGAAACGTGACATGATGAAATGGCAAACCTTACTGAACACACTGACAAAATTTGACACAAATCCGTAAACAAACATGGCCGCCACGAGCTGTCAAAATCACGTCATCCCTATTGGTAGACGCTGTATATA

>Btry_1858#RC/Helitron

TATCTTATATATAAAAATAAGTTGGGTTTTTGTCTGTTCGCTATAGCTGACTAAACCGTTGCATGAAATGAAAAATGGCCTGAACCACTTTGAAGGTAATCCAATGAGGATGGTTTGTAAAAAAAAATTCAGAAAATGTTCATTTTTAACCGACTTACGAGCGTAAATTAAAAAAAAAATCATTACTTACTTATAACAGAGAATGTTAAATTTAGACAGAGAATGTTATTTAACATTTAGCTAAGCTGCAACACACTGATAAGGGCGAGTTATAGCGTGAATAATAACAAAAACAACATTTTTTTTTGGATGCGCCAGGGCACTGGCAAGACGTTTCTTGCAAATTTAATACTTGCTAAAATAAGACAATCAGGGAAGATAGCGATAGCAGTTGCTTCGTCAGGAATTGCTGCAACTCTTCTAAAGGATGGAAAAACTGCTCATTCATCTTTTAAACTTCCGTTATCCATAAATCTTGAACAACAATCAATATGTTCTATCCGCAAAAATGGGCCTCTTGGTTAACTATTGCAAGACGCCTCATTGATTATGTGGGATGAATGCACCATGAGCCATAAAGCACATATAGAGGCCGTGGACAGAACATTAAAAGACCTTAGAAACTCCTTTGCTCTCATGGGCGGGATAACTTTTGTATTTGCTGGAGATTTCCGACAGACTCTTCCTGTCATTAACAGAGGTACGCGAGCAGATATCATCAAAGCATGCTTAATATCGTCGCCCTTATGGACTTCGATTGAAAATATAAAATTACGGACGAATATGAGAGCTCATCTTCATGGAATCACTGATGGTGATTTTCCACAGCAACTGCTTAAACTTGGGGAAGGAATTTTTCCCACTCCAAATTTAAGCAGTAATTGTAATATTCTTCTGGATGAGTCTCTAGGCCAAATAGTCCATAGTTTAGAAATTTTAATAGATGCAGTATACCCTGACATCGAGAATTTGCACGAAAAGGAATTTCATTGGTTGTGTTCTAGGGCAATAGTGTCACCCAGGAATGATAGTGTCAACGAAATAAACGATCTAATTATTCAAAAAGTTCCCGGAGAAATAAAAAAATACAAATCGATAGACACCGTGACTAATATCGAAGACGCAGTCCATTATCCCCAGGAGTTTTTAAATTCATTAAACCCTTCTGGACTGCCACCACATGAATTGGCTTTAAAAATTGGAATTCCAATTATGCTTTTGAGGAATTTAAGTCCTCCAAGCATGTGCAACGGGACGAGGCTGCTTATTAAGGAATTGAAGGACAATTTAATTGTAGCAACCATTATCACCGGCCCAGCAGCTGGTCAACTAGCTCATATACCGAGGATTCCGATGATCCCAAGTGATCTGCCAATCCCATTTAAACGGCTTCAATTTCCGGTGAAAACATCTTTCGCTCTAACTATAAACAAGTCCCAAGGCCAAACTTTCGAATTAGTAGGGATTGACCTACGAAAAGAATGTTTTACTCATGGCCAACTGTATGTTGGTCTATCGCAGGTCGGAGGTCCTGAAAATCAATTTATATTGCTGCCACAAAATAAATTAACATCAAATATCGTTTACAGGGAAGCTCTGACTCAATAAATTATTTTTCAGGAGCAGAGAAAGTAAAATATATAGTAAAGGCCTTTTTACAAAAATTAAAAAAAAATAATAATAATACAAAAAAATATACTTTCATGTTCGAATTTCCTTCATTTAACTAATTTTTAATGACCCCACGCAAAAACGAGAAAGTATGTGTGGGAGAGTGTGTACAAAATTATAACTTTTGAAATGATTGTTTAATACCCGTGTGAAGCCGGGGCGGGCTGCTAGTTATTATATAAATATA

>Btry_5673#LINE/I

AGTCACCGACTAAACCTCGTACTGAGCAGTTGAGTTAGCGTCTTTCTCTGAGTTTATAGCTATTCATATAGCTCTCTTTTGGTGTTCTTTTTTTTTGCACTACGGTCTGCTTACTTAAGTAACGCGATTAATACTTATTTTAAGTGAGAAATATAATCCAATACTTATATCAGTGTATTATAATTTTTCCACTATGCCCCCGGCGGGCTTCCAGTTCGGGGACAACAGGTTTGCTGCGCTGGCCCAAGGTCCCCAACCAAAACGTAAAAAATCGTATCAAAAACTGCAAGATGCTTTTCCTGACTTACCGCCAATAAAAAGTGATGATCCAAAGTATATTGTGATTAAATCCGACGAGAATTCTACACCCCTTTCGAAGGTTTCTTGTTTTCGTGTTTACAATGCCTTACTTACCGTGAGCAAAGATATAAACAAAATTAGTGAATTACGCGACGGCAGCCTATTACTGCTAGTGAAAAATAAGCAAGTAGCAGAAAAGTTTATAAAAACGAAATGTCTTTTCAACATTGGCGCTGTTAGCGCTAGCTATCATCAACATCTTAATTGTAACAAAGGCACCATTTATGCACCGTTTTTAAACGATGTGCCTGAAAGCGAAATAATAGAAGGACTGAGTTCTTACGAAGTTTCCGCGGTTTATAAGTTCACTCGCAAAGTTGAAGGTGTTATAAAGCCTACTGGTGTTATGCTAATATCATTCAACAGTTATTCCCTTCCAAACAAAATAAACATCGCATGGAGGATGACCTCTGTGCGACCATACGTGCCTAATCCAATGCGCTGCAGGTCATGCCAAAAATTGGGTCATACGCAAAAACATTGTAAGGGTTCCCCAACATGCGAAATTTGCAATTTCCCTTCTCCACACGATAATGACTGCATCCGAGTTATGTGTGCGAATTGTTCTGGCGAGCATCGTTCTTCGGACTATAAGTGTCCAAAGTATATGCAAACCAAAGAAATTTTAAAAATAAAGACACTAGAAAAGTGCAGTATGCACGAAGCCCTTAAAAAGTATAGAGAAAATACTTCCCTCCCTTCCCTCACTGCTAGCTTTGCACATGTGCTTCAACCTACAAAAGAGGTATCCTCTGTATCCAACACACCAACAAAAATTTCAGCCATCAACAATTCCACAAAATCTAAAAATACTCATAGAATTGCCGACAATGACTTGCCTAGTACATCAAAACAAAACGCAGAGGTCACCTCTGCTCCCAACTCATCAATAAACTCTTTAACTAGAAAAAATCACTCATCAAACGCCAACAACAAACATATTTCCATCAACAAGCCTGCATCGATCAGTTCAGACTACAACAACGTCTCACATTCAGCTAAAAATATCAGCGACAACTGTACCCAACAAGCCTCTTTCTTAAATCCCAAACTTTTCAGTCCAACAACAGCCTCTCTCATTTCTAACTTAAACAACTCCTTAAACTCGCTTAATAAATATACTGACTTCAACAACACTACTACCAACACTACCGTGCAGTCTAATCCACCTAATCACGAAAATCTCTTTCCAACAACAAGCAATTCCAACGATAATCAATACTCTACTCAACATACTGAACAAATAGAAATTGACTCTGAATAAATACGCTTAGCCTTTACTCTCTTTTGAACTAGTACTCAATTGGTGACACTCAGCTTCAACTGACTGCTCATACGAGTTGATATTCTAGCTCTCTTCTGTTTCCCTTTTTCTTTTTTTCCTACATAGATCTTATGATTCCATTATTGCAATGGAATTTAAACGGCTACACTAATAATTACAATGAGCTTCAATTATTAATACAAGATCATGCCCCAGCTATTATACTCTTAAATGAAACTCACATCTCTTTCAACTTGTCGGCTTTTACTCCTAAGCAGTACATTGGCATGTTTCACAATCTTCCACATATTAGCACAGGTAAAAGAGGTATTGCGATTCTCATAAGAAGAGATATTCCACACAAAATTAATGCCATTCAATCCAACTTGCTGGCTATGTCGATCGAAATTATCTTAGTTAAAAAAATCACCATTATCTGCACCTATATTGCACCAGATGAACATTTTACCAGTACAGACATCCTGCAATTAATCAACCAAGCTTCTACTCCTTTAATTTTCGCTGGTGATTTTAATGCATGGAGTCCACTATGGGGCTCTCCAATAGCCAACAAGAGGGGCAAATGCATTGAAGACGCTTTACTTTCCTCTAACTTAATATGTTTGAATAACGGATCCGCGACACACTTTTCCACTCACTCCACTTTTTCCCATGTAGATCTTACAATGTGCACCGACACACTTGCCACAGAGTGCGAATGGAGTATACTCGATCACCTGCATGGAAGCGATCACTTCCCCATTGTACTAAAATTGCACCTAGGTTCAACAACTAAAAACCACAAAATAAATAAGGTATTCAAAACTGATTACGCTGATTGGGAGAAGTTTCAGACACATTGCGAGATAAATGGCAATAATACCCCAATATCTGACAACCCTAACCAAGAAAGTGCGAGGTTAACAAAAATTATCCGTTCAGCAGCGAATGTTAGCATTCCTCATACAAAGCCAACAGCAAGTTCAAAGAGTGTTCCTTGGTGGAATAAAGAAATCGCTGAATTGCGGGCAAAAAAACAGACAGCTTGGTATGAATACAAACGCGCTCGATCACTAGTCAACTTAATATCTTTCAGGAAAGCCAATGCTCTTTTCCGTCGTTCCGCGAAACAGGCCAAGCGTAAATGTTTTCAGGATTTCACAAGCAAAATAAATCCTTCGTCTAGTCCTAAGTTAATATGGAACGCTTTAAAAAAACTTTCTGGAGTGCCTAGAAATCTAACCATTCAATGCGTAAAGGGGCCAACAGGTTTGGTAACCAATCCATCCGATATTTCCGAGTTATTTGCAAACCACTATTCTGAAACAAGCTCAGATATTTCATTCAGCACACAGTTTCAAACCTCTAAAACCACCACACTGTCCGACACTTCCTACTCTGTCTCCCCTCTTTCTTTTTCTGCTAAACAAATAGAAACCAGCATTTCACTGTCTGAATTCGAGTTCGTTGCATCTACCGTTAAGGGTAAGTCCCCGGGGCAAGATAAAATTTCTTATCCAATTATCAGACATATGCCAAAAAGTCTCAAGCTCCGATTGGTAAAGCTTTATAACAAAATTTTCAATACTGGTGTCTACCCCCAATTTTGGAAAACGTCCTGTGTCATACCAATACTTAAACCACACAAATCCTCCGATGAACTTGCTAACTATAGGCCGATTTCCCTCCTTCCATGTATGGGCAAAATTTTGGAAAAGATCGTGGCTAACCGCTTGATGTGGTATGCTCAGCGAAACAAGTTCATATCACCGAATCAAGTCGCCTACAAAAAAGGTCAAGGCACGTTAGATGCTTTAATCCAACTAGACTACTTCATTACTAACGCTTTGTCCAGCAAAAACCACGTGTCCGTACTATCTCTGGACTTTCACAGAGCTTTCGACAAAATTGGAGCCCATATTATTATTCGACAACTTAGAAAATGGAAAATAGGCCAGAATATGATACGTTTTATTACTAACTTTCTCACAAACAGAAAACTCAAAGTCAACGTAAATGGTTTTCTTTCTTCAACACTCCCGCTTTCTAATGGTACGCCGCAAGGCTCACCTCTTTCAGCCCTCCTTTTTATCATTGCTTTCGATGATATTAGTAGGATGATAAAAAATTACAAAGGAGTTGAGCACCAGATCTATGCTGACGATGTCCTAATCTACACAAAAGTCTCTGACGTTAATTTAGCTCAATCCTTATTTACTAATATACTCGCCAGAATTGAGACTTGGTCACTGGAGTCTGGTGCTTCACTTTCCTTAGACAAAACACATATCCTTCATGTATGTAGGAAGCAAAATTGCAACGGTATTTCACTAACCCACAACCAAACTAACATCGAATGTAAAACACAGCTGAAAATACTAGGATTAATTTTTGACTCTAATTATAATTTTAATGCTCACTGTAAATATGTCAGAAACTCGTTAATGTCACGATTAAATATTGTTAAATATCTCTCGTCTAAGCATTCATTTATTCATCCGAACACACTAGTCAATGTTGTCAGAGCTTTGCTAACTTCAAAAATAGATTATGGGCTCCCCATCTATGGCAATTGCTCCAAAAGCAGTATCAACCTTTTAAATGCACCTTACCATTGCGCAATACGGCGAAGTCTCCGGGCCTTTCCAACCTCGCCAATAAAAACGATAATGGCTGAATCTGGTTTACCAACTATTCAAAATAAAATTATAGATTCTTCGCTTCAAATTCTTGCGAAAACGGCTGAGAACACCAACCCATTACTAAATACAACTATGATTCTTCGCTTCAAATTCTTGCGAAAACGGCTGAGAACACCAACCCATTACTAAATACAACTATCACGCATGCCACGAAAAAAAGAAAAATTCCAAGAATACAATCGGCTATTTCGAGATGCTTAAGTTTCGCTGCAGAAAATAATATTTTACGTAACGCAACACGCAAATTCACCACTCGACATCCTCCCTGTCTGATCAATGATAAAATACTACAGAATAAGCTTATGTTTTTGTCCAAGCAAAACACACCAAACGAAGTCTTTCAACAAACCTTTGCTGAACTGGAATCTAATTACACAAATAATGGCTGGAAGTTATTGTTTACGGATGGCTCCAAGTCCATCGATTCCACTTCGTTAGCAGTTGTCACTGCCACAGGAGAAATTATTTGCAATTGGCTTCTTCCCTCAACGAGCTCTGTATTTACCGCTGAAGGATCTGCTATCCTTCATGCAGTTAATTACGCAAAAAAGACTAAAGGAAAGTTCCTCATCTGTACAGACAGCAAATCGTGTATGTCTGCAATAACGTCTCCTTCCAATCGCAATCCCATAATAGAAGTTATCAGGGACGCGGTCATTGGTGCCCCTCAGAAAATCCAAGTAATGTGGATCCCTGGCCATGCTGGTATTACAGGCAACCACTTTGCAGACCTCGCTGCGAAAAATGTAGCCAGGACTCCTGCCTTAACATACTACGTCTCATCCAAGCAAGACATTCTTAACCTTATTAAGCACAAAAGGCATCAAAAAAACGCAAGCGAATGGAAAACATTTAAACATCACTACGCGGTCATAAACCCAGCCAGAAATCGTATAATCTACTCGTCAACAGTTCCAACTAGCGCAATGAAGACATATACTCGATTAAGGATTGGACACACTATCATAACACACGCCCACTTACTATCGGGTAAAAGCCCATCCATTTGCCCCCTTTGCGATGACACCGCTTCTATCAAACACTTACTCACACTCTGTCCGATGCTTCACCCAACAGCCCACAACTCTGGCAACATTGATCTCATAAAACTACTAAGTGAACCTTCAGAAAAAAACGTGATGATTGTATATAGATTCTTAAAAACCAACGATTTGTTAAAATATATTTAAGCAACTTACATCTTTACTAACCCTTAGCAAATAGAATTTTTCACCTAGTTATAATTACAAGACGGCTGAAGGCCTCGTAGCCAGTGCTGCGTTTATGTATTTATATAATAAAACTCTTTTTGTTATATGAATTATTATAAATAAATAAATAAATAAATAAATT

>Btry_758#unknown

TATACACTCAGTCCTTTTTTTACGCGATTTTTGGTTCTGCCACTAATCGCGTAAAAAAAAATCGCGTAAAAATGGTTAGTTTTCCAATATTAACTGACGAATTGGTTCCGAATATAAAAAATATCGCGTATAACAAAATATACGCGCAAAAAAATATTCAAAAACAATACAATAAGTTTCAAATTTCAGACTTATGATTTATTCATTCATAACAAAATAAAAAATACAAAACAAAAACCATATATAAAAGAGAAGAAAATAGAAAATAGGTAGATTTATTGAAAAAACCGTTGAATGCTGTTTAATCACTGTCATTATCCGTAGTGGAAATTATTCTTAGCCGCTTATTTTGATGGCCGGCACTGATATCACTAGAATTTGTATCAGAATCAATAGTAAGAACTATGGATTGATGTTCTGATTGACTTAGCATCTCCGACTGAGTTTGCACCATAAATTCAGTTAATTTTGTTTGAATGCTTCTTTTTGGACCCAATAAATTCCGATGTAGTTCTTTATATCTTAACATGCAGAGACTCAATTCTTTTTGAAAAATTCGTGCACGTTCGGCATCAGTATCATTTGCTACAAAATAATTTTCCAATTCTGCTGCAAGTTTAAGACCAAGCAAAAAACGAAAAAAAGCAATCGCGTTAAAGTGAGAAATTCGCGTAAAAAAAGGAATTTGCGCTGCAAAAATAACCGCGTTAAAGTGAAATAGCGTAAAAAGAGATCGCGTAAAAAAAGGACTGAGTGTA

>Btry_4663#LINE/Jockey

TGTTAGAGTGTACGGTCAACAGTCAAGTGTGCTCCTCAATTTCTTTTAATATACGTGAAATCCAGGCTGTGGAAATTCGCAAGCAGATTCACCGGAGGACCTTAACATCGTTGGCACCACGCGGTATATCAATTTACTCGAAATTATCTACAATAATATTTAAAAACCATAAATAATTTATCACATAAACATTTGGAAATTTTAAACAATAACAAAAAATAATACCAAATTAAAAACACTCAATTAATTTTCGCGCAAAAATTCTCCTCCGAACATCTAAAGTGGTTATCAGCTGTTTCTGTCGCACGGCAAACCAGCTGTCTACGCAGAGTTGTAGGTGGCCAGGGACACTACATTATCTGCACGTGCAGTCGGTAAAGCAGTTGATAAGGCAGTTTCTTTTACTTAACGTAACCACAATTTAATAAGATCGTGATGGACATACAAACTCCGCCTCCCGGTGGGGGTACACAATGTAACCAAATAGGTATGTTGCTCGAACGCATTAAAAAAATAGAAACATTGAACAATGAATTGAAAAATGAAAATGCGACACTTAAACTTCAAAATGAAAACTTAAAGAAATCGCTAACCAAATATACAAATGAAACGTCTTCTTCTCCATCAAAATCGACCTTTGTAGCCGAAACAGACGAAGAAGAGCTTGAAAAAGAAACAAATTGGCTATTAAAAAAGAAAAAAAGTAGGGAAAGTAAGAAACGCAAAGCAGATTCTTCACCGGATGTAACACTGAAGTCTCCGATTCCGGATGGAACAGCAGAAGATAAAANAGATACAAAAGTAAGTCATCGCCCTCCGCCAATTATAATATCAGAAAATTTTAACCAACCCTTGATGCGTTCCTTGATAAACGCAGATGTAAAAAAATCTTACACACTCAAGCTCACTGGCCCAAGTTCCTGTAAAATAAATCTTTCAGATAGTTCNGACTACAGAATACTTACAAGGAAATTAAACGAATCGAAAATTCCTTGGTACTCGTACGAGGACAAGCAATCGCGTGATATACGCGTAATGGTAAAAGACCTTCATCACTCATGTGAGCCTGAAAGTATTGTCACAGATCTTCAAAAGCAAGGGTTTAAAGTTACGAAAGTCTTAAACAAACTTCAATATAAAACAAAAAATCCGTTAAATATGTTTATTGTATGCTTCGAATCTACAGAGAACATTAAGAAAATTTACGAAATCAAACATATTTTAAATTCTGTCGTTAAACTCGAGCCCATCAAGCCATCGAATCTTGTGCCGCAGTGTAAATCCTGTCAGGCGTTCGGTCACACTAGAAACTATTGTTGTAAACCACCGAGGTGTGTAAAGTGCGCCGGAAATCATACAACTCTAAGCTGCACAAAACCTGCCGAACTACCGCCAAAATGCTGTAACTGCAATCAAAATCACCCTGCGAACTATAGGGGATGTGAAGTGGCAAAACAACTTCAAAAAATTAAAAACGCGACTGAGAAAAATAAAGAGCATAACCAACAACCAAAACGCTTAAGTACAACCGCTTCTACTGCTCCTCAGAAGGAAGTTACTAATGTAAGTCAGCAATCTGGAAAACCCTCATTTGCCAGCATAACTAAAGGTGCTAATAAAGCTCAACAACATCTGTCTGAAAATCAAACAAATACGAATGCCACTGGTGATATTACTGGTAATTTTTCGTTAATTCTCAACAAGCTTAATAAGCTAGAAGAGCAAAATAAACTTACCCACGAACGTCTTACTAAACTAGAGCGACAGCAAAGCGAAAGTAAGGCGCAAAAATGAAACCGATAAAAATTATGACCTGGAATGCAAATGGCTTGCTCAAACGTAGTAAAGAATTAGAGACATTACTCCATGTTGAAAAAATTGATATTTGTCTTATATCAGAAACCCATTTTACAAATGAGACTTTCGTTAAATTCAAAAACTACAAGACGTATTGCTCGAACCACCCAAATAACAATGCGAGAGGGGGGAGTGCCATAATTATCAGAGATAATATTCCCCACTACGAAGAAATTAATATAAGTGTCCCAGAATTCCAAACCACGACCATATCGGTTGAATCTAGTTTCGGAAAAATAAGTATTACCGCAATATATAGCCCACCTAGACACAATATCAAAATGGACTTGTACACAGAACTTATACTAAAACACCATGGGAAATTCATCATGGGTGGTGATTTTAACGCAAAACACTCCCAATGGGGTTCAAGAGTTACGACACCGAAGGGGAGAGAGCTATTTAAAGCTGTTATGGCTACTGGATGCGACTTCATGTCTACTGGCAAGCCGACTTACTGGCCTTCCGACACAAGAAAATTACCTGACCTGATCGACTTTTTCATCACTCGACAAATATCCAAGACATTCATTACCATGGAAAATGGATTGGACTTAAATTCGGACCACTCGCCAGTATATCTAATCCTAAATGGCCATCTAAAAGAAATCGACATACCGCCTTATCTTTCTAATAGACATACGGACTGGGAGTATTTGGGAGTATTTCAAAGTTATGCTCGACAAAAATATTGATTTTAAAGTAAATATAACAACTCAAGATATNTTAGAAGATGAAGTTCATACATTTACGAAAACTATTCAAGAATCGGCTTGGAAAAGTACTCCTGTTCCTAAACAAAAAAACTTTTATACTAAATATCCATCCGACATTTTAGATCTCATAAAGAAAAAGAGAAAACTACGTAAAAAATGGCAAATCACGCGATTTCCTCAATATAAAACAGAACTAAACAATTTAACTAAGATTCTCAGCACCAAAATAAAGTTATTTACAAATCAAAATATTTCTAATTATATTGAGAAATTAACGCCAAACAAACATACGGATTATTCCCTTTGGAAAGCTGTAAAAAATGCTAGGAAACCAATATTGTCGAATGCACCCCTCAGAAAGCCAAACGGATCCTGGGCCAAAAGCGACGAAGAAAAAGCGGAAGTTTTTTCAGAGAAACTAACTAAAACGTTTAGTCCTTTTGCGTTCAATGGTACTCTACCGCAGCTGGAAGAATCGCAATACGTAGGTGAAATCCAACCAGCAACAAATAAAGAAGTGGAAAATATTATTAAAAGTAGATTTAAACCAAAAAANGCTCCCGGCTTCGACTTGGTGACTGCTGATGTACTAAAAAATCTAACGCGAAAAGCATTAGNAAAACTTACATCGATAATAAACGCTTGCTTAAATCTCAGATACGTTCCATTGTCATGGAAGGTATCTGAAATCATTATGGTACAAAAACCCGGGAAAAGCGCACAGGAAGCCTCGTCATATCGTCCAATCTCACTGTTGCCTATTTTGTCCAAACTACTTGAAGCTGTAATTATAAGAAGACTTCAGAACATAATTGAAGAAAAGGACCTTATACCAATTCATCAATTTGGATTTAGATCGCAACATTCCACAATCGATCAAGTTCATCGAATAACGAGCATAATTGAAGATGCGATGGAAAAAAAGCAAAATTGCACAGCTGTTTTTCTTGACGTATCGCAGGCGTTCGATAGAGTATGGCACCATGGCTTACTTCATAAATTAAGACTTCTCTTTCCACAGTATCTAACCGATCTAGTTGACTCTTACCTGAGCAACCGCTACTTCAGAATCAAGCAGGGACAATCATATACTACGTTACAGCCAATAAAAACAGGTGTACCCCAAGGAAGTGTCCTGGGACCACTCTTATACATTTTGTTTACTAGCGACATGCCTACGCCGCCAAACTGCACAATCGCCACATTTGCAGATGATACCTGCATCATTACTACTGGTAAAAATGTAGTAGAGTCGTCGAACAGAATGCAGTCTTCAATCAACCTAATTGTAGAATGGACACAAAAATGGAACATTACTCTCAACGAGTTGAAGTCTATACATATAAACTTCACTAACAAAACTGCTACGTATATCCCCTTATATATTGGGAATGAAGTAATTCCGTACTCCACCTCGGCGAAATATCTTGGTTTGACGCTGGATGCAAAGTTAAAGTGGAAGGAGCATATACAAAGAAAAGTTGCAGAACTAAAACTAAAATACAACAAAATAAATTGGTTAATCGGCAAGAAATCACCCCTAACAACCTCAAATAAAATCCTAGTCTACAATCAAACGTTAAAGCCCATTTGGACATACGGAATTCAACTATGGGGATGCGCTGCACCGTTATACATCGAAGCGGTGCAAAGATTCCAAAATAAAGTTCTGCGAAAAATTGTAAATGCCCCGTGGTACATACGNAATTCAGATCTACATCGTGATCTTCACATAAAAATGGTTCGGGAAGTCATTCGCGAAACGGCATCGAAACATGCCACAAGGTTGCAAAATCACGTAAACGCTGAAGCCCGACAACTAGGAGAGACTAGAAATAGCAGGCGCAGATTGAAAAGAACAACGTTTCATGACCTTCTAAGCAAATAACAAATATTATAATATTTAGTTTTTAATACAAAGTTTAGTTTTTGAAAAAAGCTTCTTAATAGAGCTTTTATTGTTTTACCTTATAAATTATGAAAAATATTGCTTGTTAGTACATTTGTCACTAGTTGCAATTCGAATGAAATGTAAAACCATATCTTGTTACGTTTCAATAAAAAAAAAA

>Btry_2998#LINE/Jockey

ATTACCTATGACAGTTTTTTGCGGAACGGCCCTGAGGTNCAGTCATGTAACCCTTCTCAACCCATGTCCTCGTCAGTCGTTAATGAGGAATCAATATTGAAAAAAGTGGAAGTGCTTATTCAGCAAATGGTAATGCAGATGACNGCTCAATTTACACAAATGATTACTCAACTCATGACATCAATTAATCAATGCAAGTAAGACTTAAAATTACCATATGGAATGCCAACGGCCTATGCAAAAGCAAAACTGAAGTCGAACACTATGTCAAAACTAACAACATCGATGTTCTACTTGTAGCAGAAACTCATTTCAATGATCGGTCATATTTTAAAATAGCAGGGTACGATCTTATAAAATCAAATCACCCTAGTGGTAATGCAAGAGGCAGAGTTCTTATAAAATCTGATATTTCTCACACGGAAATTAAATCCTCACAATATCCTTGGGTTCAGGTAGCAATGGTAATGTTGCCCAGCCCTCTTGAACTTTCTCCCACGACACAATCTATCGCAGGACATGTATGAGCAAATTATGAACCAATTTGGTAATAAATTCATTGCTGGTGGTGACTATAATGCCAAAAACACGTGGTGGGGTTCATCGCTCACAAATACCAAAGGTAGAGCACTTTTGAATTGCATTTGGAACAAAGGTTTGATGTGTCATTCAACTGGTGAACCAACTTACTGGCCATCCGACCTAACGAAAATTCCAGACGTTCTTGATTTTGCTATTTCGAAAGGTTTTAATTCAAGGTACATACGCTCTACCACCTGTTCGGATCTCCTATCTGATCACAGCGCAGTTCTGCTCGAAGTAAATACGCCGGTACTCGCAATGAATAAAAACAAATTGGCAAACATTTTAAATTATCGCAAATGGCTAGAAGAAAATGTGGACCCCAACATCGCCATTTCCTCTGAGGCAGATATCGATTATGCTGCGGAAGTCTTGACCAGAAGCATACACAATGNTGCCCATATAAGTACGCCAGAAGCTAGAACCACACAGAGCGTCCACAAGCGGGACAGACATCTCTGGTCCAATGAGGTAATGACGAAGCTCAGAGAGAAGAGACGTCTTCGGCATGCATGGCAAGTCAGTAGAAATCCTAATGACAAAACACTTCTCAATCGTGCCACAAAGGAGCTGAAGCATGTTCTCTGGAAATTGCGCAACAAGTCCCTAGAGGCGTTTCTGGTTGCTGCCAAACCAGGAGATCCAGTAAACAATCTTTGGAATGCGACTAGACAAAACAATAAGCCGATATCTAGATGCCCACCAATTTTAAACAGCAGAGGCCATTGGTGCAGGACCAATGCAGAGAAAGCCGAGGTGTTTGCCACACACCTCCAAAACACGTTTACTCCTAACGAGTTTAGTGATGGATCTAGCAATGCGGCAGTCAGCAATTTTTTGGATGTTGCTTGTCAAATGGAATTTCCGATTGCTGAAATAGATGCTGTAGAAGTGCAGGAGGAGATCAAAGCATTGGCAAACAAAAAGTCACCAGGTTATGACAGAATTAATGCGTTTGCACTTAAAGCGCTACCCGAAAACGTAATCAGCATGATTGTCAAGCTTTTTAACGGAATGCTCAGACTTTGTCATTTCCCATCGCAATGGAAATGTGCTGAGGTTATTTTAATTCTTAAACCCGGAAAACCTGAACATCTGGTTAATTCTTATCGACCAATTAGTCTACTGGTAATTATCTCGAAAGTATTCGAAAGAATATTTTTACGCAGGCTTTTGCCGGTGTTGGAAGACAAACAGCTTATTCCGAATCATCAGTTTGGCTTCAGAAAATTTCATGGCACAATTGAACAATGTAATAGGGTAGAACACTTTATAACTGATGCTATGGAAAATAATAAATACTGCTCTGCTGTGTTCCTTGATGTCCGCCAAGCATTTGACAAAGTCTGGCATCTAGGCCTATTATTCAAATTAAAGGAAAAACTACCAATGCCTTATTATTTGCTGCTAAAGTCTTACTTGCACAATCGGACGTTCTACGTTAGATGCCGTGATGCTGAGTCGGATTTGAGACCTGTTCGTGCTGGCGTGCCCCAAGGCAGTGTACTCGCCCCTGTGCTCTACATTCTTTATACAGCAGACCTTCCAGTGATCAACTCTGACTCCACAATGACGGCAACCTATGCTGATGATACTGCACTGCTAGCATCAAGTGGCAACCCTAATACTGCTTCCAGAAAACTGCAGATGCAATTGAATGCTCTAGAACCGTGGCTCACCAAATGGAACATTGGCATCAACACCGAAAAGTCCGTACAAGTAGTATATACCCTCAAGCACAAAAATTGCCCTCAAGTGGCTTTATATGGTCAGTTGTTACCAACAAACACATGCGCAAAATATTTGGGGATTACAATTGATAAGAGGCGCACTTGGAAAGATCACGTAAAAATAAAAAGAACTCAGCTAAGATTAAAGTTGGCGCAGCTATACTGGCTCCTTAGGCCAAGATCCCGCCTCAGTCTGAAGAATAAGTTGCTAATTTATAAGGTAATACTTAGGCCGATCTGGACGTATGGCATTCAAATCTGGGGTAGTGCAGCGAATTCAAATTTAAAAGTAATTCAAGCTTTTCAAAATAAAGTTCTGCGCACCATAGTGGGGGCTCCTTTCTATGTGAGAAACGATGTCCTCCACCGGGATCTAAAGTTGTTTACTGTAAAGGAAACAATCGAAGAATTTAGCAGACGTTACCTCTTCCGTTTGGAGCATCATGAAAATATATTTGCGATTCAACTGCTGGACAATAGTTTACAACGTAGAAGATTGAAAAGATACCACTCGCTAGACCTCCCCTTTCGATTCAATTAATGTATAAAATTTTATTTTATTTTTTTTTATTTATTTAATGTCATACTACCATAATTCATCTCATTGTGTTTTCATGAAATTTAGTTATTGTTTTTTATATTTAAACAGATTCTAAATATATTGAAATTTACAAAAAAAAAAAATAAT

>Btry_3051_LTIR#DNA/hAT-hATm

ATATATATATATATTAGGCCGGGTCGATTTGTGGGGAGGCAAAAAAATCGCCCATTGCTCTATGAAAATCATATTCTAGGGATCAAAATAAGAAACTTTGCCGAAGGAACCATACCTCTGAAACGAATTCTGATGTCCCCCAATTTGGGTCGAACTTTTGGGTAGGGGCAAATTTTGAAATAGAATTGAAATTACCATTTCATTCTTATTACCTCTGAAAGTGAGGAGAACTAAAGCAATAACCACTATGGTATTTCAAAAAAAATAATAAGTGAAGTGACCATTCCAAATCAAAAAGTTTACAATGAATCCACCATCCTCTACACCGCAAGATGAAGCAACTACATCAACAACTATCGAAAACCCAATGAGTCATATACCCAAGCATGATGTTACTGTTTTTGGTGTGTCTACTGATTTGACTGATCTTAATTTACCAACCCATCTGGATATCTTGAGATATTATTTTTACTTAAGCGAACGTGCTAAAACAGAACAAAAAAAGTTTTCCCATAAATCATTCACTATTCAAGTACAAGATAAGTTGATTGGAATTTGGGAAAAACTTGGTATGGAAATAATGCTGAAAAAAAGTGTATGTAATAAATTGAATAAATTGCTTGACAAGTATCAAGAGCAAATCAAGAGAAGAAACAACACCCAACAATTTACAGAGTATGTAAAATCTCTGGAAACAATTTTTTACATTGGAACATGTAAATGCGATTTGAAGGCAGCTCCATGTGCATGCGGCTGGGTTCCCGAACGCCTCAAAGAGTTCATGCACGATCAACATAATCAGAGAAGACTAACAATGAATGCATTTATGATGGAAACTGAAGAGCAAGGAGCAACGTCTATGTCATCAATGCCAACATACCAAGATCCCGATGATTCAACATACGCACCGCCACCGGTTCAAGAAGATATGGATAGAAGCACAAGTTCACAATACACAGAGAGATACGATTGTTTTAACTTTGCCTTGGTATGTGACAGATTTGGTGTGCCTGACAGAGTAGCATCAGCATTGGGAACCGCTCTTTTGCAAGATTTTAAAATTAAAGATAAGCATGGGAAACCTCTCATCATGGATAAATCGAAAGTTCGCAGAGAAAAAGAGAAATGCAGACAAGAAGTGCTTCGCAAACGGTTAGATGATACCAATTTGTTAGCGTTTTCATTCGATGGCAGAAAAGATGATACTTTAACGATAGATAAAATTGATGAGAAGTATCATACTAGGATGGTAAAAGAACCTCATCTTGTTATTTTGAGAGAACCCAATTCTGAATTGATTGGTTACGTAAGACTGGAACATGAAACCGCTGAATACAAAACAACCAAATTAAATGGTTTTTTCAACGATAAAAATATATCACTGGATACATTAATTGGAATATGCACTGACGGTGAGCCAACAAACACTGGCCCACACGGTGGAATTATACGACGATTCGAATTGCTGTTAAAAAGACCATTGCATTGGTTTGTTTGCCTTCTACACTTCAACGAACTTCCGTTTCGGCATTTGTTTGAAGCTTTGGATAAATCAACCAGCACTGGACCAAGATCGGCAACCGGAAAACTGAGCCGTCAAATCGAAACTTGTGAAACTCTTCCGGTAAGTTATTGCTATCACTCATTTATTATAATTGTCAACCATTTTTAATTATTTTATTATTATATATTTTTAGGTGGTGGACGGCTTCCAGAAAATTGAGTTGCAAAATATGCCCCCTGCTCCAGAAAAAAAAGAATTTTCCACCGATTCGAAGTACTTATACGACATGGCCCATGCAATTTCTAGCGGCGTGGTTCCGGTGGATCTGGCTAACATCAAACCAGGGAAAATTGTACATTCTCGGTGGCTCACCAAGGCCGCTAGATTATTGCGATTATATGTGACAACGGAAAATCCAGATGCAAATTTAAGAATTCTGGTTGAATTTATAATTAAATGTTATGTGCCAATGTACTTCAATATCAAGTATTACAGCTCTGTCGTGTACGGCAGTGCATTATTTTTCAAGTTCATTGGTTGGTCACGATTTCTAGAACCTCGTTTACGCAAAATTGTCAATCAAGTAATTAAAGATAATTCATATTATGCGCATTCGGAAAATATCTTGTTATCAATGTTGTTTGATGATAGGAAAGAAAAGCGCGACTGTGCCATCAAGAAAATTCTACGCTATCGAACCGATGTTGACGAGCCAATGGAACTTAGAGTTTATAAAAAACCAGATATAAACTTTAATTGCACAAGTTATACGGAAATGATCAATTTGAATGATATAAATATCGTATTTGAACCACCATTCACGCGAAGCATTCCGTACGATACATTGAAAGAATATTTAAATCAAGATGATCCACCGTTTAATGATCCAAAAATTCCATCACACATACAAGGAACGGAACGACATGTTCAATTGCTAGCTAGCGTTTCCAAACGGGTTATACCGGAAAATGTAGAGGCTGTTATGGCGACGACATTAGAGAGCCGTGCGAAATTGCCCAGACTTGAAAGTAAAAAAGACTTCAAACAATAATTTTTTTTAGTTTCTTTTCTATAACTCACTTAAATTAATTTTTTCATTTACATATGTTCTGACTAAATAAATTTCTTAAGAGAAAAAATAGATATTATTATAAATAAATAAATAAAAATAAAGAAAAAACATAGCCATTTAGCTGATTTTTTCATGTAAAGGCCAAAAATGGTGATTTTTTGAAATGATTGTATGGGGAACCCCCCAGGGGAGTTCCAGGGGGTGTGCCACTGGCATGGGTGGATCGGCCGTCCAAAGTTAGTGGGGGTCGGTCATACATTTGGACTCGATTGGAGCACTCTAAATGGGTCAAAGTGGGATTTTTCAAAATTTGCCCCTACCCAAAAGTTCGACCCAAATTGGGGGACATCAAAATTCGTTTTAGAGGTATGGTTCCTTCGGCAAAGTATCTTATTTTGATCCCTAGAATATGATTTTCATAGAGCAATGGGCGATTTTTTTGCCTCCCCACAAATCGACCCGGCCTAATGTACATA

>Btry_MarR#DNA/TcMar-Tc1

ATATACACATATGGTCAAAATAATAAGTACATTCCTTTAGGCTGCAGTCATGATTGAATTTTGAATGTTTGGTGAAGTACACGAATGCAACTATGGGTTGATTTTATTAAATTCGATATGGTAGTACTTTTTATAGTGAAAAGAAAATGCTGAATCCACTCTTTGTAGTGGGCGTGGCAATTGATTGTTGATGTTCGTTTTCTAAGTGGTCAAAATAATAAGTACATTGTTAATTATTTTCAGATTTTCATCAAATCGAGTGATTTATTTTAATGGTTTGCTCTTTAGGTTGTATAGCTCCATGTTTAAGACTCATTAAAAACTAAAAAAATGTATATATGGGTAGAAAAAAGCATTGCAGGGCGGAGGAAAAGGACATCGTCTTAATCCTCATAAAAGAGGGCCAAAGTCTTAGGAATATCGCTAAAACATTAGGCCGTTCATTGTATTTCGTGCAGAATGCCCTAAGGATGAAAAAGAATGTTGAAACTCGCGGTCGACCAAAAAAGACATCAACAACAACGGACAACCGTATCGTCACTCTTGGTAAAAAGGACCCATTGGTATCTTCAAGGATAATATCAGCCGATATTGGTAGCATAATATCTTCCCGAATGGCCCGACGAATACTATATCGAGCTAATTTACCTGGCAGGATAGCCAGAAGAATACCATTATTGCAAAAAAAAAATTTAAGATAAGATTACAATTCGCGAAAAATCATGCAAATTGGTCCGGCCCAAGTGGTGAGAAAAAGTGGGCGCAATATCTTGTGGAGCGACGAAACAAAAATTAATTTGTTCGGAAATGATGCCTGACGGAATGTTCGTCGTCCGAAAGGCAAAGAATTGCATTCACAGTCTACAAAAAAGACCGTTAAACACGGTGGCGGCAACATAATGGTCTGGGGCTGCTTTTCGTGGTATGGTGTAGGACCAATACATCGTATTTCAGGCACCATGAATAGGTTCCAATATAAAGATATCTTGGAAAACACGATGCTGCCATATGCTGAGGAAAGTATGCCACTAATATGGAAATTTCAGCAGGATAATGATCCTAATCATACTTCGACGTTGGTAAAAGATTGGTTCCAGGACAATGGTGTGAGTTCTCTTAAATGGCCATGTCAATCTCCCGACTTGAATCCAATTGAAAACCTTTGGGGGGAGCTAAAGCAACGGATTGGGAAGCAATCGTTCCAAAATAAGGATCAGTTAGGGAATTTTGTTGAAAAAACCTGGTATGAGATTCCAATTGATACGTGCCGCAAACTTATCTCCAGTATGCCAAAACGAATCTCAAAAGGAATTGAAAATAATGGCGGATATACTAGATACTGATGAAAGAAGTAAATAAAAATCAAATAGGAGACAGAAAACTCAAACTAATACATTTTTTCATTCACAGATACCTGATGTACTTATTATTTTGTCCAGCCATTTTTTTAGTCTTTTTATTATTTGCTAGAAATTTAAGTTCCTATTTCAACTATTATTACAAATATCTTTATTTTTATTATTTAAGTCATATATGAAATGAAATATGATATTATTTCTCTTTAAACATGAATAAAAGAGAATTTATTAGTCATTGAATGCTACACGGGCGTATGTACTTATTATTTTGACCATGTGTGTATAT

>Btry_MarS#DNA/TcMar-Tc1

ACATACAGTTACGGACAATAAAATAGAATCAAAGGTTTTGCTTTGAAACAATGGTATAATTTTTTTGAAATTTTTCATTTATGTAGTAACTTTTCTGCATAATAACAAAGGCAATAATATGTACTTTTAAAAAAGAGGAAAAGATAGCATTGTCGAGTTTTACAGTTAACTGTAATTTAAAAAGTGTAAATAGCGTGCCTCGTGCTTAGTGACAAAATAATAGAATCAATAGCGAAATGGTTTCATAAACGAAAAATATTGAATTTAAAAAGCGTAATAAGTAGTTATATTAACTTAATTAGATCTGTGGAATGAATAATTGTATCAATTATGAATTTATTTAAAAAAATGGGTCGAGCAAAACATTGTTCAGAGGAGGAAAGATTGTGCGTCCAAAATTTGCAAGGAATGGGTCACGCGTAAAGGCAAATTGCAAACATATGAAATATGTCTCAAATATTTGTGTTTGATGCCTTAAAATTTCCGAAAAANGGTTGAAAAAATAGGCCGACCACAAAAAAATGACTGCGCAAGTCGTTCGAAATATTGCATGATTGCCGATGCGCGTACCGTTTAAGGCCTCTGCAGCCATTCAATAAGAATTATGTGCAAAAATTTCTTCAAGAACAGTAAGAAGACGACTTGTGGAGAAAAATTGCTTTGGCACATGGTCCCGAAAGGTGCCAATGCTGAGAAAACGGCATAGATGCAATCGTATTCAGTTTGCAAAACGACACATTAATTAGGGCGGAGCTGAAAATTAAAAAAAAAATGGTTCGACATTTTATGGTCTCAATGCAAAATTAATATGATAGGAAATGATAGTTGGGCGTGAGTCCGACGTCCAAAAAACAAAGAAATGTGCGTCCGCTACACGACTAGGTCCTTCAAACCCCGTGGCGGCAATATAATGGATTAGGATTGTTTCTCATATTGTGNTGCGGGACCTATTTTTTGGATACAAGAAAACATAGACCGCCATATATACGTTAAAGTTTTAGATAAGGTCATACTCCCATATGCTGAATGGAATTCGCCTTGCATTTTGGCAATTCCAACAAGAGAGCGACCCGAAACATACTTCGGGTCTTGCAAAAAATGGTTTTCAGATCATGGTGCTAGCGTTATGGCTTGGCCAGAGTAATCAGCAGATCTGAATCTCATCAAAAACTTATAAAAATTTTGTTAAGGAAAATATTGCACCCCTTAAACCGAAAAATAAGGCAGAATTCTGAGAGAAGGTACAAAGGTCTGGTATTCTGTTCCACAGAACACTTTCGGTAGCCTAATTAGGTCTATGCCTAGAAGATGTGCATCTGTATTAGACG

>Btry_4535#LINE/CR1

CGTAATCGGTCGTCACTGTGAACGGACGTATTTTTTCTCTGCTCCGCGTTCTTTTATTTTATCTATATTAATGCCGTGTGTTTGCGGGCATAGAGTTGACCGTGCTCAGCCCAAGATTAGTTGCCATAAATGCAATCAAATATTTCACTTGTCGTGCGTGAATTTATCACAGGCTGATGTAGATTTCTTGGTGAAATCTAAAAATGCTTTTTATTGTAAAGTGTGCGCGGTAGTTCGGAGAAATTCCATTCGCTCGCCGCCTGCGTCGCCATCAGTGCGCAATGCTCAATCAAACAAGGAAGTAACTGAACCTTTGTTGGAGCATAACAATCATAATAAAGAAAATGTAGTGCTTAATTCTCTGGTTTTGGAAGTAAGTTCACTGAGAAGTGAACAAACGAAGGCGCTCGCGTTAATACAGCAATTATGTGATGATCGNAAATCGTTAATCGCTATGCTTAACGAACTCAAATCAGAGATCTGCACTGTGCATAAGAAATTGTCCGATAACGGCAAGTTCTCTGCACCAGCTTCGAATGCTCTCTCTGCTGCCAACCCCACTCTCACTGCTGCGACTGCTGTTGTTACCAGTGACTCCACAATCGCTGCTCCGTCTAACACCACTGTTCCTGCTGATAACGTGTGTGAGAACTATCTCGCTCCCGCTACACTGAAGGTACTCTCTGCAGCTTCAACTACTGCAAACTCTATTCTCACTTCTACGACTATTGCGAGTACAAATGCTCCGTCGCCGAAAGTTGTTGTTTCTGCTCCCACTGATTGTGTGAGTATGGATTATCTTGCTCCCGCTACTTCGAAAGCACTCTCGGCTGCTCCGTCTGCTGCTTGCTCCGCTCGCTCTATTGCTGCTGTTAATGGTACAAATACAGCNCCGTCCAACGTTGCTGTTTCAGTTTCTTCTGGTANTGTCAGCGTGGATTACTGTGCTCCTGCGGCTAGACCTNCTGCTGCTNCCAATGCTGTTAATTATGCCAATACATATGCTGACGCTGTTGGTAGGAACATCAACACTCACTCGAAAGCCAATAACAATCCAACAGTTGCTGCTAAACCAGATCGCCAACCTGTCGCTGTCAATGCTGCTCACTCTGTCGCCGTTGATGATTCTGTTAATCTGAACTCTTCTACTTCAATTACTGCTAACAGAAATTTGAAGTTAAAAGTGAGTACTAGGAACAAAAAATCCCCAGCCACTTCAACTGTTGTTGGTGTTAATGCCAATGTTGATCTCGATGTCGTCATACCCAAGAAATGGATTCACTTATCTGCCTTTAAGAACTCAGTCACGGAGGAAGATATTATATCTTTTGTTGTTAAACATGCTAATGTTGATAGAGAGCATCTTCTATGTTATAAATTAATTAAGAAAGATACCGATGTTAATAATCTTAAGAGGATAAACTTTAAGCTAGGTGTGTCCCCATCTTTTTTTAATGCTGTTTTCTCCTCTTCAGTGTGGCCTTCTGGCATTAAGCTGCGTCCCTTTAAGTTTTTTCCAAAGGGAGGGGAAAACAGCGCTGCAGATTAGTTGATGCGCCTGTTAATCGCAATTCTACTCATGCGTTTTCTGCACCTTCCTCAACTGGCTCCTGTCTTAAGGTCTACTACCAAAATTTATCGGGTATTAGGTGTAAATCAAATATTATTCGCAATTTTTCTTCACGTTTGGATTTCGATATTATCGTTATTGTTGAGACTTGGCTGAATTCTAGTTATTTCGATAGCGAATACTTTGACTCAAATCTCTATTATGTCTTCCGCAAGGACAGAGATCACGAAAAAACTGGCTGTCTAAGAGGGGGTGGAGTTCTCTTAGCAGTTCATCGCAAATATCGTCCTCGCTTAATTATGCTTGAGAATGAAGATTCTATCCTGGACCAACTATGTATCTGTGTCAATGATGCTCTGTTCATTGCAGTCTCATACATCCCTCCTAATAGTCCGCTAGAGTTGTACAAGTTGCACGCAAATAATATTTCCTCGCTGGTATTAAACAAGGTGAAAGAGAATAATATTTGTGTTTTGGGTGACTTTAATCTAAGCAATATCGTCTGGTCTGGTTGTTCCTATAGTCCTGCTCTTATTCCTTCAAATTTTTCGTCTTCTCAGGAGTTATTTCTCATTGATAGCTTGCTTGGTCTCAATCTGATTCAAATCAACTCATATTCAAATAGTCTCGATCGATTTCTTGACTTAGTCTTTCTCAGTGATAATTTAAAATTTAGGTTTCTTGATGGATTCTCTTCAATTACTCCAGCCACCATCCATCATTCACCGCTAGCGTTTGAGGTGGATACTTATCTTTTCGCTCCTGTTAAACCAATTGATGGGCTTGGTTTTAATTTTGCATTTTGTGACTTTGAACGACTCAACAATCAGCTCTTTGAAATAAAATGGGATGATTTACTATCGGGACTTGATACAGCTAATAGTTTCTCCATTTTTAAGAAATCAATCCAGGAATTTTGTCATTATAATATGTAACAATACACATTTAGCACGTTTAAAAAATCTTAGAAATAAATATTATAGAAAATTCTCTTCAACTAAATCTCATGTTTCCTTTGTTCAATATCAGCACTACACAAAACTGTTTAATGAATTGGATAAGTTCCTCTATAAAAGGTTTATTAATAATACGGAGTCTACAATTAAGTCGAACCCTAAGTTCTTTTGGAACTTTGTAAAATCTAAAAGAGCGTGTTCGGCCATTCCCTCTGCTTTACGCTGGGGTGATAAGTCGGCATGCACTCCGGTCGAAATCTCCAATTTGTTCGCTGAATTTTTCAAATCGAACTATGTTCAAGATGATCCTGGTACTAGTTCCACTTTTTCAGCTGATAACTTCCCATCCATAAATTTTGGCTCACTGTGTCTTTCACAGGACGATATTGCCAAGGCTATTTGTGACATTAAGTCTTCGTCCAAATTGGATTTGGACGGGCTTCCGCCCGTATTTATAAAAAATTGTACTGCCTTATTATATCCTCTCATGCTTATCTTCAATAAGTCTCTCGCCACCGGGGATTTTATTTCTGACTGGAAATTGACATGCATTACCCCTATTCATAAAGGTGGTAGAAAGGATGATGTCTCCAATTACAGGCCTATTTCTAAACTTTCTACTATTTCGAAAATTTTTGAGTGTTCTGTTAAAAATAAATTATTTTTTGCAGTAAAATCAATAATTAATGTCAATCAGCATGGGTTTGTCCCTGGGCGCTCCACTGTGTCAAACTTAGCAGTTTTTAGCGATTATTGTATGTCTGCATTCTCCGCTGGATTTCAGGTAGATTGTGTCTACACAGATTTCTCTAAAGCCTTTGATAAAGTATCACACAATATTCTAATTAAAAAATTATCATCTCTAGGATTTCACTCGGCCTTCTTGAAGTGGATTAAATCATATTTGCAAAACAGACGATGCGTAGTCANTGTCGATGGAGTGTCATCTGATTCATTCATTGCGTCTTCGGGAGTCCCACAAGGAAGCGTCTTGGGTCCACTTCTCTTTGTGCTGTTTATCAATGACATCTCCTGTTGCTTCTCTTTCGCCAACTTTCTGTTATATGCAGATGACTTAAAAATTTTTGCATTAATCAATAACACACAAGATGTANTCAAACTTCAATGTGATATTGATACTTTTCATAATTGGTGCCTGAAATCGAAACTTTTTCTGAATNTAGATAAATGCTCACAGATCTCCTATGGTAGGGGACGCAACATCTTATCTTCTAGCTACAGTATTTCTAATTGCGTCCTAAAATGCGTTACTGAAATCAAAGATCTTGGGGTAATCTTTGACAACAGATTTTCTTTCAGTAATCACATCAACTATATCACTTCAAAATCTTTTTCTGTACTTGGCTTTGTCCGTCGAAATTCTACAAATTTTTCCGACCCCTACACGTTAAAATTACTGTACACATGTTTTGTGCGGTCCATTTTGGAGTATGCAGTGTTTATTTGGAGACCATATTGCATATCGTCAATTAACAGGATCGAGCGTGTTCAAAAGATCTTTCTTAAATATGCTCTCCGCTCCTTAAAGTTTGTTGACCCAATACCATCGTATACATCGCGTTTATTGCTTTTACATCTTAAATCACTGGAAAATCGAAGGTCCGTTCTCTCACTTTCATTTGTCTACAATGTAATTAACGGGGAAATTGACTGTCCCTACTTATTAGGGAAACTTAACTTCAATACTCCCCAGCGTTGTCTCAGATCCATTTCGCCATTTTATTACAAAAAGGAAAGTACTAATTTTGCTGAATATGCTCCCCTCAAGAGGGCTATGCGGGAGTTTAACTCGATTTCCGAGAAAGTTCTACTTGATTTTTCTCACTCTAGGGTATCCTTCATTAATACCCTCAATTCTGTTTTTTAGTTTAAGTTATCTACTCATGTTATTGTAATTTAATTATTTTAGAATTCTAATATGCATCCTAGTTTTTCATTAGTCTGTAAGAATCATTGTTCATAGACTTTACTAAATAAATAAATAAATAAA

>Btry_6505#LTR/Gypsy

ATATTTTTACTTCAAATATGGCCAAATCATTGCCTTTATTGATATATTTACAAATGTACTTAATTGATTTTACAGAACTGCAAAGCTCGACATTGATATGAGCCTCATGAGTTTTTGAAAGTAATGGTGAATATGGTACCACCCACTGATTATCAAATTCTACTGGATTTGTAAAATTCGGCCATTGCAATGTAAATGTGTGGCCACCATTCTCAGTATTTCTGCGCCGGTACATTGGATAATTGTCAATATCTGTGATAGTGGCATTCGTAAGCAGCTTTAAGAAATTTTTCTTACACTTTCCATTTTCCATGCAAGTTAAAGCCACCAATAGCTAACGGCACTTATGCCTGAAATAAACAATAAAGTTAACGCCAACAATAGCTAAGGGCACTTTTGCCTAAAGTAAACAATATTCATATAAATTCATTATTAAACGAATGTGTCAAATATGTTATTGACACATCGCCAATAAGGAAGCGGCCATAAACTATAACTTATGGCACGCTTTAAGTAGAATTAGACAGCACATCATCTAACACCTAAGTCTGCACTTATTAGAATGTAAGATGAAACTCTGTATCTAGGCTTAAGATATCAATTGTATAAATATAAGTGAAATAAAGATAATTTAGTTTTGTTTTTGAACATAACTCACTAGATTCACATCTTTTTAAAAACCACCCCGCGTTCGCGCAAGCTTATCAATTACAAAAGTAACATTGTTATTGTAAAGCAACAATCCCTTGCATATACCACGCAACGTGACGATTTGCGAAGGCAAACTAAACAACAAGTGCAACAAAATTAAAAGTTAAGCGGCGAACGATCCACAAAGACAACAGTGTAATCAGCAAAAGCATAAAAATCAAATTAGAAAGAAAAAACTAAAAGTGCAAACAAAGCGATGAATCTACTCAATCAAACTAAACCTATAGTTTGACTAAAGAAATATAATAAAAAAAATAAACAAAAACCAAAAATAAATATAATTTCAAAAATATTTAGAACACAGAGTTCTTTAAAAACAACAAAATAAACAGTGCCAGTGAAAACTCCGCATCAAAAGCAATAAAGCAGCGACCAAGCATAAAGGAAAAAGGCAACTAGTTTCCAAAACAACAGCAGCAGCAAAGGCAACAAGAGCAACAGCAATAGCAGCAAAGGCAAGAACTAACAGCAACAGTAGCAACAGCAACAGTAACAACAGCAGCAGTAGCAACAACATAAAATTAGTTGTAAGTAAATCCTATATTTATTAATTAAATGGGAAATAATCCCACTAATATTAAGTATGAAAAATTGTTTCACTGTGAGTATTGCAAAACCAATAATCATGACACTCACAAGTGCCCTTATTTAAGAAAAAAGAAAACAAAATAAATTCTTATACATCTATTAAATATATATAAATAAAAAAGAAATTTAACCATATTTTTGTGAGTATTACTATATATGCTATTTCATGAAACTCACAAATATATAAAAAAAAAAAAAACCGAAAAATAACGATTATAAAAAAGAGGCGATAGTTCAATATCAAGAAATAAAGAATTTTTAAGCGAACAAGATAATTTTACAATTTTTCAAAATGGCAGTGAATAATTTTACTGTAGACGATATAGCTAGATTAATAGCTACACAAACCGCAAACTTAAGAACTGAGATAGCTCAGTTGAGTAATAGAATTAAAATTCTAACAGATGCGTCACATGTAACTGAGTATGAAACTCAAAGAATTAACGATTCCATAACATGTGACGAAAATTTAGACATAATAAAGTCTTTGCCAGAATTTTCCGGTAAATCTTATGTCAGCTGGAGAGAAGCTGCAAAGAATTCGATGAGCCTATATATTGAAGGTAGTAGAAGGTATTTTGGAGCATTAACTATCCTCCGAAATAAGATTGTAGGTAATGCCAATGATATGTTAACTAATCATGGCACAGTTTTGAATTTAGAAGCAATATTTGCAAGATTAGATTTTGCTTACGCCGACAGACGACCAATTCACGTAATTGATCAAGAGATGAGCGTTATGAGACAAGGATCGACATCCTTAATTGATTATTATAATGAAGTCAATCAGAAATTGACATTACTAATAAATAAAACTATAATGACGCACGGCACCAATTCAGATATTACAAAACAATTAAATATCAAGAATCGAGAAACCGCGTTAAGAACTTTTATAACTGGTTTAAATTACCCTCTAAACCAAATACTATTTACATTAACACCTACCGATTTGCCAAATGCTCTTGCTAAAGCACAAGAACTTCATTCAAATCAAATACGATCGCAATTTGCTTTGCAATTTACTAAAAACAACCATGAAATTGGTTCACGAAACCAGCCTCAAGCTAACCGAAGATATTCGAATTTTCAGAATAACTTAAGGTACCCCCAAACAAATAATAATTCATACCAAATGTATGCCAATCACCCAGAACCTATGGAAGTGGATCAATCAATCCAATTAACAAATAAACCCAGTCATGTGCAAAGAAACCAACCACATGTTAGGAGTAACAGGTATAATTGGCAGACCAATTATCAGCTAACAAATATACCAAAAAGGTCAATTGAAAGTGCACAAATAAGCACACAACCTCAACCAAAAATTCAACGAATTAATAATATTGGTGAGAACCATTTTTTAGGGTAAACTCCGATTTGCCGTATGTTATTAAAACCGATACAAAAACTGGGCAAATCTTTAGAATTCTCATAGATACGGGGGCAACCAGCTCATATATACGAGCTGGAATTTATAATAATAAAAACATTTTAGTAATTAAGAAGAAGGTTAGAACCATTCATGGTTCGACCATAATAAAATATTTTCATAATATCAATCTGTTTGGTATACAGGAACGTTTTTATGAAATTAACAATTTAGAGAGCGATCTTTTAATTGGAATTAATTTCCTAAATAAAATCGGAGCGACAATTGATATTCCGAATAGAAAATTGATTTATGGGAATAATAAAACAGAGAAAATAAATTTTCTCAATGATTTAATTATACTAAACCCCTTGGGACAAAAAAGTCCTGAGGCACCCGTCAGTAATAATATTAGCAAAAATGATACTAAGGATAGGAAAATTAATGAAGCAAATGCTCACAACATTTTAAAAATAAATAATGTTGAACATTCTAACGAAGTTGCTGATACGTACAAATGTAATAATAATGAAACGAAAGATATCAACATTTCATTGCTAAGAGAGGATGTTATTAAAATAATAAATAAAGCTCATCAAAACGTGAACATGGATTTACCTTTTAGAACAGATATTAAAGCAGAAATTAGAACTGAGGATGACAAACCTATATGGTCCAAACAATATCCTTATTCTATATCTGCAAATAATTTTGTTAATAAGGAAATCGAAAGTTTACTCGCAAAAGGGATTATACGACATAGTAAAAGCCCATATAATTCTCCCGTTTGGGTCGTTCCAAAAAAAGGAATAAATGAAGATGGTAGTCCGAAATTAAGACTGGTCATCGACTTTAAAAAAATTAATGAAAAAACAGTAACCGACAGGTACCCAATGCCTAATCCCGAGGTGATACTTTTTAATTTGGGAGAATCAAAATACTTTTCCACAATAGATTTAGAGTCTGGATTTCATCAGATATTGATGGAAGAAAACGATATAGAAAAAACAGCATTTTCTGTAAATAACGGAAAATATGAATACCTTAGAATGCCATTTGGACTTAAAAATGCTCCAAGTATATTTCAGAGAGCAATGAATAATATTTTACATGAATTTATAGGAAAATTTTGTCACGTATATGTTGACGACATAATAGTATATTCAAAAACTCTAAAGGAACATATATCTCATTTGAAATATATAGTACAAACCCTCGAAGAAGCCCATATGAAAATATCTATAGAAAAATCTAAATTTATAGAAACCGAGGTTGAATTTTTAGGATACGTTGTATCCCATAAAGTTATAAAAACTGATCCCAAAAAAGTAGAAACAATAAAAAATTACCCATATCCCAAGACGTTACGACAACTTAGAGGATTTTTAGGATTAACGGGATACTATAGGAAATTTATAGGAAATTATGCAACAATAGCTAAACCACTAACAAAACACTTAAAGGGAGAAAATGGTAAAATATCGCAGTATATGTCCAAAAAAACAATTATTAATCTTGATCAAGAAGGAATTGTTGCTTGTGAAACCTTAAAAAGAGCGTTACAAGAACAAGTTGAATTAGCTCAACCAAACTTTGAGAAAAAATTAATTTTAACTACTGACGCATCTAATGTTGCTGTAGGTGCAGTTTTATCCCAAGAAGGAAGACCAATAACATTTATTTCAAAGACATTAAACTCTACCGAACAAAATTACGCAACAAACGAAAAAGAATTATTCGCCATAGTTTGGGCATTAAAGACATTACGCCATTATCTTTATGCAGTAAAAAATTTAGAAATTCATACGGATCATCAACCATTAGCATTTGCAATGTCCGAAAAAAATCCAAATGTTAAAATGAAAAGATGGCGAGCTTTTATTGAGGAGTTTTCTCCAAAAATAATTTATAAACCGGGAACAACAAATGTAGTTGCTGACGCATTATCAAGACAAGTATTAAATAATTTAACTAGTTCATGTGAGTCGAGTGACTCAAGTGATTCACTAATAGAAACCCAACATTCCGCAGAAAGTAGCGATGAGTACCAAATTCATGAAACAAAAAAACCTTTGAATCATTTTAAGCAACAGATAGTAATCGATAATGGAGCAAGAATTACTGTCTTAGAGACTATTTCTTCTTTTAATAATAAACGTTTTTTAATTGAATATGACAGACCAGAAAATATAATTGGTATTTTAAAAGAATATATGAATCCAAAAGTAGTTACAGGAATATTTTGCACACCTGAAGTGCTATATGCAATAAAAAACATTTTGAAGGAGACATTTCCCACGATAAAATTTCTATATACTAAACTATTCCCTAATGATATATGCAATAAAGAAGATCAAGGANCGATAATCGAGCAAACACATAATAGAGCGCATAGAAATTATAGGGAAAATTTGGCACAAATTAGGCAGCAATATTATTGGCCTTTAATGATAAAACAATTTAAACAGTATGCAAAAAATTGTAGCATATGTAATAGTAATAAATATGAAAGACATCCGAAACTAATTCCAATAGGGGAAGCACCAATACCAAACAAAGAGGGAGAACAACTCCATATAGATATTTTTTTCGCACAAAAGCTTAAATTTCTTACATGTATAGATTCATATTCCAAATTTCTAATCGTAAAATATATAGAAGACAAATCAAATTTAGAAGAAAAAGTTTTGGAACTGTTACAAACATTTCCAAATGTTAAAAGTATAGTTATTGATAATGAACCAGGCTTAAGTACGATACAGTTCAAGTCTTTAATGGAAAGGATAAATGTACAAATTTATTATTGTACACCACGCCACAGTACAAGTAATGGCCAAATAGAAAGAGTTCATTCAACACTGATTGAAATCTCCAGATGTTTAAAGCAAGAACACAGCTTAATTAGTAATTTTGAATCAATTATGAGAGCAGTTCAACAGTATAATAAAACAATTCATTCTGTAACAGGTAAACAACCTTGCAACATTTTATTTAATAAAGAGCCTCACGATAATATGAAAAATGTGTTGCAAAATGCACAAGAGAAAATGCTAGAGCATCATAACAAGAAAAGGCTTCAGAAAAGTTATCANAAAGGAGATATTATATATGAAAAAATATATGGGGAAAGAAATAAATTAAACCCCAAGTACAAAAAACAAGTAGTATTAGAAGATTTAGGAAATACAATTAAAATACAAAACAGAAACAGAATAATTCATAAAGATAATATCAAATCTTAAATCTTTTACCTTCAATATACAAGAAAAAGAAATTTTTTGTCTAAATGATGTCACATAAAAAGACTTCCATCAAAAAATAATTAGAAGTCAAAAATAATATAAAATGTATCGAAAGTATATTTAAGAGTCAAATTTAAATAAATTGTAAATATAAATTAAGATTTCAAAATAGTACTGTTCAAATTGATAAAATAGTCTATAGCCTCACGTACTACTATATATCCATTTGAATCGGGACGCTTCAATTTAGAGGTGGGGGAGTTAATACCACCAGTTCACATTACATGAACATATTCTTTATCTGTCATAAATATGTAACATGACACTTACGACCCATATGGTCCGTCATAAGTGGATTGGATATGTAGTTAGAATGTAAAATGAAACATTGTACTTTGCTAATACTGTCCGCCTTAGGTGGTCCAAAAATGTAACTCATAGATTGTAAGATGAATCTTTCCACTTGTAACATATGTCCTATCTCAAGTGGATTGTAGATATACTTCCTAGAATGTAAGATTAATATTTGTATCTAGGCTTAAGCAAAATATTGTATTTAAGAAAATGTAATAAAGAAAACG

>Btry_475_sTIR#unknown

ATAACACCGGTCAACAAAAAAAATATTTTTTTTGGGGTTTCTGTTCTCATGCTTGAATTCTGATTCTAGACATTGAAAAACACTTAAATATTAATCTTTAGTTCTTTAAGTTTGCTTTGGGCTGATCTACATCGATAAGTATATTTGGGGTTTAATACGAGAGAGCTCATATGAGCATAAAAGAAATACAAAAAATATTCACTTTTAGACAGTAATTTCTTGTTATTTTACATTTATATATTATTGTTTATCACACATCTTGAATAAAAACGACTCTTGTATCTAAAAAAAAGTTTTCTAATTATAATCTATAGTGAAATTGTAGAAAAAATATAAAAATCATCGAAAAATTGCTCTAATTTCTACGAAAGCAAACTTTAACCGGAAAAAAAATTTTTTCCTTATTTTTGTCATAGGAGAAACTAAAATTTAACATGTAGATGTCAGACCCAAAAATCGTGTTGACCGGTGTAAT

>Btry_MarT#DNA/TcMar-Tc1

ATACACTTTGTTTCACCGCTATAAAGACGTTATATTTTCGTGAAATACTGAAAAAATGTGTTCTCATTCCAAGAGTGAATGATGCTATTTTAAAGCTCTATGGTAACTCTAACAATTCTATAAAAACAATTTTTTTCTAACGGTACTTTTCCTACTATTGTAGGTAGTAAGCGGAATGCTCTGAAAACGGTGTTTCATTGCTATAAAGACACTCATACCGATCAGTGCCATTTAGAGTTATAAATTAATTTTATTAAATTGTGTTAGCTAATCGGAGAATGCCGCGCAAAAGCATACTTAACGATAAAGAGAAAGGAGCTATAGACGCTTTGAATGGTGAGGGTTTGACCAATCGCGCCATTGCTCGGAATTTGGGAAGGTCGGTAACAGTGGTGTGTAATTACCTGAATAACAAAGAAAATTATGGCCAGAGAACTTACTCTGGTCGAAAAAAAAATATTAACACGCGACAGGAAAGAGAAATTTGTCGACAGTTATCAAATAAAACGACGTCAATACGGAAAGTGAAAAGAGAGAACCAAATTTCTGCATCTGTTTCAACAATTTGGCGTGCGGTGAAACGAAATCCTAACATTGTACGTGAGCAAATGCAGAAAGCGCCTCTCTTGAAACCACACCATAAAGTCTCTCGTCTCAGTTTTGCCCGAAATCACATGAATAAAGACTGGCATCAGGTAAGATTAAGAAAAATAAAATTTTGAATAATACGGTTGCGGCAAAGTAGACAATGCGCTTACAAAAAACTTTACTATTGGTCCTAGGTGATTTTTTCCGATGAGAAAAAGTTCAACCTTGATGGTCCAGACGGCTTTTTGGGATACTGGAGAGATTTACGAAAAGAGCCGCGATATTTTTCAAAGCGCAATTTCGGCGGAGGCTCACTTATGGTATGGGGCGCATTTTCAAATTCTGGGACAGTACCGTTGGCATTCCCTTCTTGCCGAATGAATAGTTCCGAGTACCAGGAAGTATTAGAAGCAAATCTGCTACCCTATATACACAGTCATCCGCAAAAATCTTTCTCTTTTCAACAAGACAACGCAAGAATCCATACGAGCAGGTCAACGAAAACGTGGCTTCAACGTAAAAATATCGATCTTTTGGAATGGCCAGCTTGTTCTCCTGATGTAAGTCCTATTGAGAACATCTGGGGATGGCTGGTAAGAAAGGTTTACGAGGATTATCGTCAATTTAATACGATTTCTGAGCTGAAAACAGCTATTTTGGATACATGGGCTTTATTAACCCCAGAAATGCTTTCTCATTACACCAACTCAATGCCAAATCGAATATTCGCACTTATTAATAATAATGGAAACACAATAAATTATTAGGAAAAAAGAGAAATTTGTTTCTTTCCTTTTTTCGTGGTGGAAGGGCTGTCTTTATAGCAATGAAACACCGTTTTCAGTGCATTCCGCTTACTACCTACAATAGTAGGAAAAGTACCGTTAGAAAAAAATTGTTTTTATAGAATTGTTAGAGTTACCATAGAGCTTTAAAATAACATCATTCGTTTTTGGAATGGGAACACATTTTTTCAGTATTTCACAGAAATATAATGTCTTTATAGCAATGAAACAAAGTGTATATA

>Btry_2822_Harbinger#unknown

TATATATATTTACACCCCTATTCTGTGCACTTGACAAATTCAACATATTTCTAATTTGTCAAAATTTTAAATTTATCAAGCATTTGGTATTCTGTGTCTGAAATAAAAAGCTCTTTTCTTTATAAGTTGTTAAGATGTCAAATGCTCTTGACAGTGCTCGTTATGAATTAAATATCTGATTGTGCATCTTGTTGTGAAAATGGAAGATATTTTACTTTTGTTATTATTGTTAAACGAAGATGAAAATTGTGAGAATAGGAATCGCGCGCGGCATTTAAAATGTTTACGCGACGATAGCAATCCATTTTCTTTGAGCGAGAATACCTTTGTGCAAAATTTTCGATTAACTCGTGAAATTTGCCGCCGCCTAATTGATGAACTGGCCCCACATGATAATCAGAAAACGTCACTACCTTTAACTGTAAGGGTTCTAGCAGCTTTGAACTTCTTTGGACACGGCTCATATCAGAAATGTGTAGGTAACAACGTCAACTTGCCCATGAGTCAGTCGTCCCTTTCGAGAAGTGTGCGAGCTGTAGCAAAACTTATTGTGAAGGTGAAGGGAGGAGAAATAAAGTTTCCCAGTTCAAAAGAGGAAGAAACTTTTATAAAAACGGGGCAAGTATTATTGTAATACAAATAATAATATACTAATTTAAGCTAATTTATTTGAAATATATTCCAGATTTTTTAGAAAATTTGGAATAAAAAGCACCATAGGAGCAATTGACTGCACGCATATTGCTATCATTGCTCCACCATCAAATAATGTCGAACGTCCTCTTAATTTATATTTAAATAGAAAAGGATTCTACAGTATCAATGTTGAAGCAGTAAGTTTTGTTTTTGCATGCACATACATATATGCCAATGCAATTTTTGAATGTATCGTTTTTTAGGTTTGCGATCACCGATTATGTTTTACATTTGTAAATGCTAAATTTCCGGGTGCAACACATGACTCTGGAATTTGGGCAACTTCTGACCTACGAGAACATCTCATTCGTCAGCATACTAATACGTCTGAGCAACAACGGAGAGAATCTTGGCTTCTTGGCGATCAAGGTTATCCTCTAGAACCGTGGCTTTTAACACCAGTGGGAACACCTAATACCCATAAAGAGCAAAAATACAACAAATTACATGGTTCTGCAAGAAACTGCATAGAGAGAGCATTTGGTGTTTTAAAATCTCGCTTCAGATGTTTGTTGAAGCACCGAGTTCTACATTATTCACACGAAACTTCCGCTTTGTTTGTTAGTACTTGCGTTATATTGCATAATATAATGACGAAAGCAGGCATTACGTTCAACGAAATAGATGACGGGGTTGCAGAAGACTTTGATTCTCCAATTAATGATTATAACTCTTCACAGTACATGCGCGAAGGTGAAAGAACAAGATCAAGATATATATCAACTCTTTAGTTATATATATATACAGAAGCCAATCCATATACTTATGTACATATATAAATGTTCTCACAATTCGACTTTCGAAATACACTACTTACTTCCATTATTTGTTTATTTATTTATCCTTTTTGTTAAAAGAAAACAAATTGAAATTTCCATTCCATTTTTTTATTCTATTATAAAACAAAAAACCAAACTTAATTAATTCAATAAATATTAATTAAATAAAAATATTACACATACGTTATTTCAATTTTAATTAAACTTTAGATATTATTTTTCATTACTAGTAAACGATTTTATAAGTTCTGTTATAGAACTAGTTAGGCCTTGAATGGCCTGCATCATTTCACGATGCTCCCGTCGCCTCTCTTCCTCAGCAGCATTCCTTTTTGGTATATCAGCTATCAAAGTGCTGATAAGCTTATTTCGTGCACTTCGTGATAGCACCGAAGTTGAAGCATCCTTAAAAGAAAAAACGTAATAAGAAATTGCAAAAAAAATGTATATATTTTGTAATTATATCATAATGAAAACTTACCTGGTTTTCCAAGCTAAGATACGTAGATGGTGTCGATGGACTTGCTGTATGGTGTTCGGGATTTGGGCTTGCATCTACCTGACTGCATAATTGCTGAGGACTACCCGTGGGTGATGAAATTGCGCATTCATCCGTCAAAGGCTCAATTGGCGTTAAACCTAGGCTTTGNACATTTTGCATCCCATCTACAGCGGCCGAGCCAAATGTAGACAAAGCCATTTCTTCGAAGTCCGTCATCGGCTTGGAACTTGGACCTCCACCTGTGAGCCTTTGAGTCATTTTCAACCGCCTTGCTCGAGTGCGCAGTTGGCTTTTCCAAACGCCTAACGTCTATAAATTAACACAATATAATAAATAAACGAAAAATAAACTAAAATGTTACCTCTTTCCATTTTGCAGCACTTCGTATTGGTCCTCTGCACGAATTAAGTTGATCTGCCAGCTGTTGCCACAACTCCTTCATCCGTTGAGGCGTTTTTGGGCAATTTTTCCCCGTACCTATTTCTGGGTGTGCTTGGCAAAACAANACATATGCCTCCAGCTGCTCGTGCGTAGCTCTATCGTGCTTGGGTTTACTATAAAATAAAAGTTAAATTATAAATTTTGTTAATAATTAAAATTTGGAAACTTACGTTGCAGAACAGTCCATTTTAACGAATAATTTGAATAGAAATCAGCTGTTTTCTATTTACATTATTTTGTTCACCACACGTGTGGGGAAAAGCTTTACGAATTGAAGCTTTTACTTTTATCAAGATTGCCACAGAATACCAAAATATTTCTCGCTTGATAAATATTTGAGTTAATTTTCAATATTTTATTAATTTGTCAAGTGCACAGAATAGGGGTGTAAATGTGCGCG

>Btry_MarU#DNA/TcMar-Tc1

TACAGGGTGGCGCACGAATCGTGCTACAAAAACAAACCGTAATAACTTTTTTCTGTGTGAGTAATCGACTTACTTTTTTATTTGGCAGGTTATTATTTAAATTTTTTAAAAATGAATGCTTGGGATACCGAAAAGAGGGTTTGGATAGTGCAGCGGTACCATGCTTTGCAGTCCGTCATTTCCGTCCAAAGGGAATTCAGACGGGATTTTGGCGGCACTCCCCCGAGTAGATGGACCATTATGAGGTTGGTAAACATGTTTGCCGAATCTGGAAGTATCGCAAGAAGACCGTACCATCGAGATCCGTATGTTCGTGTTCAAGAAACAATCGCGGCTGTTGCATCGTCAATTCAGGCAAATCCAAGAGTTTCGACTCGCAACTTATCGGCGCAACTTGGAGTTAGCAGACGGTCATTACAACGGATAATTCATGATGACTTAAACCTGTTTCCGTACAAAGTTCAAATAACAAGCCGGTTAAACCCTCTAGATTTGCCTATTCGCCTGGAATTTTGCCAAAAAATGATTGAAATGGCCGAAGAAGACAATAACTTGATAAATTGCCTGTTTATGTCTGATGAGGCCCATTTTGATCTAAACGGAAATGTAAACAAGCAAAATTGCCGTATATGGAGTGAATCAAATCCAGAAATAATCCACGAGACGGAACTTCACCCAGAACGAGTCACTGTGTGGTGCGCAGTTTCATCAAGGTGTATTGTCGGGCCATACTTCTTTGAAGAAAACGGTGTAACAGTAACTGTCAATGGGCACCGTTATTTAAAGATGTTGAATGAGTTTTTTTACCCAGAACTGCGCCGAAGACGTATCCCTTTCAATAGCGTTTGGTTCCAGCAAGATGGAGCAACTGCACACATAGCCAGACCGGTTATTGCGGAGCTCCAACGAAAATTTGAAAACAAATTAATATCAAGAAACTCCACTTTCCGCTGGCCCCCAAGGTCACCTGACCTGACTGCACCAGACTTTTTTATGGGGTTATGTCAAGCAAGAGGTGTACAAAACAAAACCCAGAAATCTGACTGAATTGAAGCAGTCCATTAAATCGATAATTGAGGCAATCCCGACTTCAACCCTTCAGGCCGCAATGAATAATTTTCTAATTAGGTGTCGCATATGCGTGGCTGAGCATGGAGGTCATTTACGGTCCATAATTTTTAAAAATAATTAATTATATAAAATAAAATAAAATTTGCTATACAATAAAAAATAAATGTAATGCATTGATTAAAAAAAAGTTATTACGGTTTGTTTTTGTAGCACGATTCGTGCGCCACCCTGTATAAAAAT

>Btry_5224#RC/Helitron

ATCATACTAATATTATAAATGCGAATGTAAGTTTGTTTGTTACGCTTTCACGCAAAAACTACTTAACCGATCATCATGAAACTTTGTACATATACTCTTGAAGGTATTAGAAGTAACATAGGGTACTTTATATTAAAAAAAAAATTTACGAAAGATAAAAAAATTGTTTGTCAAAAAATCGTCAAATTTAGCTACTTCAACGCCATCTAGCATTACAGTAATGAAGTTTCAACCATGAATACTGTAATACCGTCCCACTGTATTACCGGCGGTGACGACAATATCTAATTCAATTGAATATAGTTTCAACTGTTTCTAATTTTTCTATATTTATTGTTATTTTCTTCTGTCGTTACATGCAGTATAAAACTATTCAAATTTTTGAGGTAATCCGTAATTTTTGTGGTTAGCTGTCACTTTATTATTTTAGATCTTACTCGCTGATGTTAATTCTTACTCTGCTTTTCTTTGAAAATGCCTCGGAAGCGGAAATCTGGTCTATCCAAAAATTCATCGCGGGCCCGTGCCGCAAAAGTTGCACGAAATCAAGAAACTTCGGCTCACGCAGAGCTGAGAAGACAACAACAAGCAGAGCGACAAAGAGTTTTGAGAGCAGCTGAAACGCCTCTTCAGGCACGAGTTCGTTTAGAAAGACAAGCTGCACTGCAGACAACTAGAAGAGCGATAGAAACACCAGAACAATCGCAGGCGAGAAGAATTCATAATGCAGACATGCAGACCACGCGCCGCCGAAATTTCATGCGTAATGATTGGGCTGTGTTTAATGGTACTGGGTTTCAATATGATCCTTCAATTGAATATCATAATCATCCATTGATTGTTATTGGCTCAATGAATAAAAAATGTCAGTACTGCGATGCTTTTAAATGGAAAGATGAAACTGCTGGAATGTGCTGTTCCGGTGGTAAAGTTTCACTTCCATTACTTGGTGAACCAAAAGAGCCTTTGAAAACTTTATTATTAAATGTTACAGATGAGTCAAAGCAATTTCTAAGCAAAATAAGAAAGTATAACTCTTGCTTTCAAATGACATCCTTTGGAGTTGATAAAGTGATAAGAATGCCCGGGTTTTCACCAACTTTTACTGTACAAGGACAGATATATCATCAAATTGGATCACTATTTCCAGAAGATGATGATCAGCATAAATTTCTGCAGGTATACTTTATAGGTGATGAACAAAATGAAGTAAATCGTCGTTGCCAATATATTGAAGGAGTAGAAAGAGAAACGGTATTGAAAATTCAACGAATATTACACAGTCACAATGTCTTGGTAAAAATCTTTAAAAGTGCGATAGACAACTGGCCTTCAGACAACTACAAAGTTATCATTCATGCTGATCGAACTCCACGTGGCGAACATGAAAGGCGTTATAATGCGCCGATGGTCAATGAAGTTGCTGTTTTGGTTACTGGTGACCCGAGCTCTCCACGAGACATAGTACTACGGGCACATGACAATACGCTAAAGCGTGTAGCCGATACTCATAGATTCTATGATGCTTTGCAGTACCCATTAATTTTCAGTAAAGGAGAATCTGGATATCATTTTAATATTCCAGTCATTAATCCAACAACGGGACAACCAATTCAAAATAAAAAAGTTTCCTGCATGGACTTTTATGCGTATTACATGATGCTTCGAGAGCATGACTTCAACCTTCTTCTGCGTACAAGTCAACTTTTTCATCAATTCTTGGTAGATATGTATATTAAAGTAGAGAGCGAACGTCTTCGTTACATTTCTCTAAACCAGAAAAAATTACGAGCTGAAAATTACATACATTTACAAGATGCGATTAGCGCAGACAATAATATCAGACCTAACGACATTGGCAAAATGGTTATTTTGCCGTCCACTTTTGTGAACAGTCCCCGTTATCTCCATGAGTACACGCAAGACGCATTTTCTTATGTACGAACTTACGGAAGGCCTGATCTCTTCGTAACTTTTACCTGTAATCCTTCGTGGCAGGATATAACTCAAGAGCTTATGTCTGGGCAAAANGCCACTGATCGACATGACATAGTTGCCCGCGTGTTTCGATTAAAGGTCCAAAAGTTAATGAACGTTGTAACAAAAGGAAAAATATTTGGAGACGTCCAGTGCCATATGTACTCTATTGAATGGCAGAAACGAGGTCTACCACATGTGCACATATTAATTTGGCTAAAGCAGAATATACTTCCTAATCAAATTGACAACATAATTTGCGCTGAAATTCCAGATACAGAAGAAGATAAAAATCTGTATGACACGGTTATTAAAAATATGATACACGGTCCCTGTGGAGCTCGCAATCCAGCATCACCTTGCATGCAAAATGGCAAGTGTACAAAAAAGTATCCTCGAGAAATGATTAAAGAAACAGTTCACAGTGATAAGGGTTACCCGTTATACAGACGTAGGGCGCCTGCTGATGGTGGTAAACAAGTTAACATTCGTACTCGCAGTGGGGAAATAAAGAGCTTTGATAATAGCTGGGTAGTGCCCTATTCCCCGATTTTGTGTAAATTATTCAACGCCCACATTAATGTTGAAGCTTGCAATTCTGTGTGTGCAATTAAATATATTTGCAAGTATATAAATAAAGGAAGCGATCAGGCGATCTTCAATCTGCGTAATGAAGGTGCTGTACAGGCCCTCAATGAAATACAAACTTATCAAGCAGGAAGATATGTCAGTAGCAATGAAGCAGCATGGCGGCTTCTTGGCTTTCCATTACATGAAAGATATCCCACTGTTACACATCTTGCAGTACACTTAGAAAACGGACAGCGTGTCTTTTTTGATGAAAATACCTTTCAAGACAGGATTTTATCTCCTCCTAAGACCACGCTGACTGCCTTTTTTCACCTTTGTCAGAATGATACTTTTGCAAAGACATTATTGTACTCTGAGGTTCCCCGTTATTATACATGGAACAGTTCAAAAAAAGAATGGCGTCGTCGTATTCATGGTACACCTGTTCAAAACCAACCTGGGGTTAAAGCATCGGATGCTCTTGGTCGTGTCTATACCGTACATGTTACGAATTTTGAATGCTTTTGTCTGCGCATGTTGTTGCATCATGTTAGAGGTCCAATTAGCTTTACAGAACTTAAAATTGTCAATGGTCAGGAATGTCAAACATACCGAGAAGCATGTGAAGCTCGGCGACTGTTAGAAAATGACAATCATTGGGACGAAACTATGGAGGAAGCTGTACAATGCCGATCGCCAGATAAAATCAGGGAACTTTACGCTACGCTTTTATATTCTTGCGGTCTTTCTAACCCTCAAACTCTTTGGAATAAGTACAAAGAATACATGGCTGAAGATATTTTACATCAACTGCAGCAAGTACACACAGATATTACTTTCAATGAAAATGTCTACAATAAAACGCTAATTATAATCGAAAACAAGGTTTTTACGATGGTTGGAAAAAAATTAGATGATTTTGGAATGTCGAGCCCTCGACGAGACAATATGGATGATTTTGATAATGAAATTGCACGAGAGCTAGATTATGATTTCATAGCACTGCAACATCAAGTGACAGAATTAGTCCCTCAATTACTTCCAGAGCAAAATCATGTTTTCCATCAAGTTTTACGTAAAATAGACTCCGGCAGTGGTGGACTCTTCTTTCTCGACGCACCAGGAGGAACTGGAAAAACGTTTTTGCTTAACTTATTATTAATGTCCGTCAGAAAAGACCAAAAAATAGCAGTTGCTGTAGCTTCGTCAGGTATTGCCGCGACGCTATTAAACGGCGGTCGTACGGCACATTCCGTTTTAAAATTACCATTGAATTTGGCACAGGAAGATTCGCCCATCTGCAATTTTAGTAAAAATAGTTCACGAGGTAGGATGCTGCGAGAATGCAAGCTTTTAGTGTGGGATGAAAGTACAATGTCTCACAAGAAGGCTATAGAAGCTTTAAACCGGACTCTCCAGGACTTGCGAGATAGTACAGATATAATGGGAGGNATGGTAGTTTTATTGGCTGGTGATTTCCGTCAAACCCTCCCAGTGATTCAGAGGGGGACACCAGCAGATGAAATTCAAGCGTGCATTAAATCATCAAGCTTATGGTCGACAGTTGAAAAACTCCGCCTGAAAACGAATATGAGAGTGCATCTTCATAATGACGTGGATTCAGGACTTTACGCAGAAATGTTGTTGAAAATTGGTGATGGTTGTTTAGATGTTGACGCTGAAGGCTACATATTACTGTCAAGAGAATTTTGTAATTTAGTAGAAAGTGATGTGGATCTCATTGCTAATGTTTTTCCGGAATTGCAACAAAATTTGTGTAGTGATCAGTGGTTGTGTGCAAGAGCAATATTAGCACCAAGAAATGAAATTGTTAATAGGATCAACACTGACATTTTAAACGAGGTTCAAGGAGAAATGAAGGAATATTTGTCAATGGATACAATTATAGATACGGAACTAAGTACTTCATATCCTGTGGAGTTTTTAAATTCACTTGAACTATCGGGTGTACCGTCACATAAACTTCAATTGAAACTAAATGTACCAGTAATGCTTATGCGAAATCTAGATGCTCCTCGGCTATGTAATGGAACAAAGCTTCGGATAACAAAATTGGGACAGAACATACTTGGTGCTACTATTTTAACAGGTGTCGGTAAGGGAAATAGTGTTATAATACCTCGGATACCAATTATTCCCACTGACCTTCCATTCCAATTTAAAAGGGTTCAGTTTCCCGTCAAGCTTAGCTTTGCTGTTACTATAAACAAGGCACAAGGACAAACATTACAGGTAGCAGGAGTGCATTTAGAAAACCCATGCTTCTCTCATGGTCAACTTTATGTAGCCTGTTCACGTGTATCTAATGCCCGGAATTTACACATATTTGCACCCGACGGGAAAACTTATAACATTGTCTACAAAAATATCCTAGATTAATACTTTGTATTTTATTTTTTTAAATTGTTAGAATTCAGAATTATTTATTTTTGTTACGGTGAAATATATTTTAACATTTGTTGTTGCATTTCAATACGCATATTTTAAGGTGTTGAAACTTCATTGTCTGTAGTAATTTTTAAAAATTGTTGATCTCAGCCAAGCAATAAAATTTAGTTATCATTTTGACTCATTTCTATTATTATACCCTTACCCTTTATCTCTCATACTTATTTAATGGCTCCACTGCGGGCGAAGCCGCGGGTAAAAAGCTAGT

>Btry_MarV#DNA/TcMarTc1

TATATACAGTTGTCCACAAAATAATAGGAGTGCCAATATGAAAAATGTGTTTGGCTGCATAACTTTTTTAAATCACATGTAAATGACGCGAAACAATAACGGTACACATAACGGGATACTAGCATTAAAATACAAAACAAATTTTTATTGTGATGATTGCTATTCTACTTCTGCTTTGCTAGTTCTCGGAGACACTTGTTATTGCTTTCCACAAAATAATAGGAGTGTTAGATATTTCGTTTTCAAAATAGTCCTTAACATAAAAAATATTCACAAAATTAGAAAAGTAAGTATAGTTCCATAATCTAGCAGCAATTTAAATTAGTTATTATTTTATTTAATCATTGGGTTTTTCTCCAGTGGGTAGAGGAAATCATTGTACAGAGGAAAAGCGGAAAACGATAAAAAAATTGAGAAAACTAGGAAAAACTTATAAAGAGATCCAAGAAATCGTAAAATGCTCTCCTCAAATGATAGCAAATGCCTTAAATTATAGAAACAAAAATGAAACTCGTAGACGAAAGCGCAAAACATCCACTGCAGACGATCGCAATATAGTACGCTACTGCAAAATTAATCCTTTTGCTTCAGCCAGGAGCATCCATGACGAACTAAATTTGTCAGTTAATACTGAAACTGTTCGTAGGCATTTGGAGCAGAACAAATTATTTGCCAGAAGTCCACGAAAAGTACCGCTACTCAAAAAGAAACACAAGGAAGCCCGCATACAATTTGCAAAAGAGCATGGCTCCTGACCCGCGTCTAAATGGCGAAACATATTGTGGTCAGACGAATCCAAAGTTGAGTTATTTGGTAGTACAGGCTCTAGGAACTTTGTCCGACGTCCCCCTAACTCAGAATATAATCCCCGGTTTACCACAAAAACCGTTATACATAACGATGCCAAATCATGGTACGCGGTCGTTTTTCACATTGTGGTGTTGGTCCAATTCACTTAATTGAAGGCATCATGGACCAAAGGGCATATGTCAAAATATTAGATGAGGTAATGTTGACACATGCCTCATGGAATATGCCATTGATATGGGTATATCAACAGGATAATGACCCTAAACATACTAGCAAGTGCGCAAAAAAATGGTTTAGGGCCAACGGAGTTGAGGTTATGTGGTGACCTGCTCAGTCTCCTGACCTCAACCCCATTGAAAACCTGTGGACAGATATCAAGAAGAAAGTAGCTGAAGAGAAACCAACCAATTCGAGAGATTTATGGCAAGTAGTGAAAGAGGCTTGGAAAGGAATACCAATAAAACGTTGCCAGGATCTCATAGACTCCATGCCTCGGAGATGTGATGCTGTGTTAAAAAAATCGAGGTTATTCAACGAAATATTAATTATTTTAATATTCTGTGAAACTTTGGAAGCATTATATATTAAAATTATAGAACGGTCTCGTATTACATAAAATACTCCTATTTTTTTGTGGAACTAGTTTTTTAGTTTACTTGATATAAACTGCGATATAAAATTTTGTTTATGTTTAATTGAAATAGATGAAATCTGGAAATATTAATAGATCTTTAGCAAATTACATTGTGCAAAAATTTTGAAAAAAAAAAACATTTTTGTTAATGAAAATTAACAATTCGTTTTTCTCATGTGGTCACTCCTATTTTTTTGTGGACAACTGTATATAC

>Btry_4580#LINE/Jockey

TACAAAGTTGGCAACCCTGGAGCGAAGTCAAGAGAATTGAAGTGTGATAGAATAATATTCAATAAATTCAAAATTTTAAATATTTACAACAATTAAATTAAAAAACTTGTTTCTACACGGAAGTGCTTTTTACGGAGATTGCCTGGAAGTTGAAGTGCGGTGAGATGATCGAGTGGATAGGCTGTTGCACCTGCTAAAAGTTTCTCAGTGGATGTAAGGCAACCTATTACCATTTTTTCTTATTGGACTTTGACCAGCAAAAGGGAATCTCAAAACTCACTACCTAGTATAAGCTTTATTTATCTAATACAAGAACCAATTATATTTGCGTTATTTATAGTTAAGCTTTTACCTGTATTAGATCAGCCTAAAAGTATACACCGAAATTGGTAAATGTTTTATCTAATAGTTGCCACATAGTTATTTTTACTAAATATTGGCTCATTATGAAAAATGTCCGAACGCCTCCACCTGGTGGTGGCAAAACATCTGAAAACCCAGAAACTACGGCAAATAAACTAAATCATTATGAAACAATTATAAACCAACAATCCAGGCAGTTGGAAACAATAGAGCAATTATGCGCCGAATTAAAGAAAGAGATAGCATTTCTCAAAGCCGAAAATCAGAACCTAAAAACAAAGAATTTAAGTCCTACAAATAACGAAATAAATGCTGAATTGTATGCAACAGATGAGGAAGAGCTTGCCCGCGAAACAGATTGGATTTTGAAAAAAAGGCCCAGTAAAAAAAGGAAAGCTGAACTATCTCCTCAGCTTGTGGTTCCAGCTACATCAAATACCATAAAAGAAGTCGGAAGAAATCAAACAGAAACGAAAATCTCACGACCTCCTCCAATAATGATATCGGGTGACTGTGAATACCAAAAAATATATAAAATTACGAATGATGAGGCAAAAAAAGCATTTACTATTAAGCTTCTAAATAATAATACATACAAAATCAATACATCAGACTCTGATGATTATAGAAAGTTAACCAAAACTCTGTCTACACAGAATTTACCGTGGTTTTCTTATGAAAATAAGCAATCTCGACCAATTAAGGTAATNGTTAAAAATTTACATCACTCAAACGAGCCAGAGGCTATAATATCAGACCTCCACAAGAAGGGATTCAAAGCTATAAACGCAGTCAACAAACTGAAGTGGAAAACGAAAGAGCCGTTAGATATGTTTCTTCTTACTTTCGATGCAACAGAAAATATAAATAACATATATGCAATAAAAACCATATTGCATTCAGTTGTTGCCATTGAGGCCGTAAAGCAGTCAAAGCTCATACCACAATGCAAATTGTGTCAATCATATGGACATACTCAAAACTATTGTGGTAAGCAAGCAAGATGTGTGAAGTGTGCGGGAAAGCATAAGACCATAGAGTGTCAAAAACCTAATCACAATCAACCAAAATGTTGTAACTGTGGTGAGGCTCATCCTGCTAACTACAGAGGATGCGTAGTTGCGAAAGAACTGCAAAAGATTCGTGACCAAAGAACTGGTACAAAGAAAAACGATCCACGTCCTAAAAGCTCGGTTGTAAAAGATACCAAAAGCCTCCCAAAAAACGCAATACGCCAACCAATGGCTGAAAATAAAGTTTCAACAGCAAATGAGCTAACTTATTCGCAAGTTGTAAAAGAGAACACTTGTTCAAATGATGCTAGCATTCTTCAACAAATCTTAGAAAAGCTTACAAAACAAAATGAGTTTACCATTGCACTTGAGAAGAGGCTTCAGAAGCTTGAAAAAGCAGTACAAAAAAATAAATAAATATGACAAAATTCTTAAAAATTATGCTATGGAACGCAAACGGCCTTACTAAACACCAAAATGAGCTAGAAACAATACTCACTACTGAAAAAATTGACGTATGCTTAATATCTGAGACCCATTTTAGCAAGCAAACTTACATTAAATTCAGAAACTTCGAAATGTATCATGCAATACATCCGAACAATTGTGCACGCGGAGGAGCTGCTATTATAATAAGAAGCAACATAAAACACCACGAAGAAAACAGCATATCTACAGATGAATTTCAAGCAATAACGGTCACGATAGATAGTGACAACAACTTGTCCCTTACAGCAGTTTACAGTCCCCCAAGACATCAAATAAAATGTCATAGCTATCTAGAACTGATCAATAGCCATAAAAACCGATTTATAATGGGTGGTGATTATAATGCGAAACATACTCACTGGGGCTCGAGGCTTATAACCACAAAGGGCAGAGAGTTATTGAAAGCTCTTCAATTGACTGGGTGCAGTATCATGTCTACTGGAACACCAACTTATTGGCCAACAGATCCACTTAAAACACCAGATCTTATTGATTTTTTTATTACAAAACATATCTCCTCAAATTATATGTGCATAGAAAGCGGTTTAGATATGACCTCTGACCATTCACCAGTATATTTAACACTACACGAAAGTGTTGTTATTAAGGAACCTCCACTTTCGCTAACAAATAAAAATACTGACTGGGAATACTTTAAGCAAATGCTATCCAATGTTGATAGTCACGTAAAAATAAGTTGTAATTCCGAGCTAGATAACGAAATCATGAATTTGACAAATACAATTCAATATGCAGCCTGGAGTAGCACACCAACTAAAAGAAACGTCACAGCAGGTCATAAGTATACCAAAGAAATTAAAGAAATGGTAGCAGAAAAACGCCGATTACGCCGAAAGTGGCAACAAACAAGATCTCCATCCGACAAAAGTTGTCTTAACAAATTGTGTAAAAAACTTACCAATAAGATAAAGGCATTTAAAAATATAACCCTAAGCAATTATCTGAAAAGTTTATCTAACGACCATAGGAATGATTATTCACTTTGGAAGTGTACAAAAAAATTTTCTCGACCCGTAACTCAGCATCATCCAATCAGAAAAGCAGACCAGACATGGGCCAAGAGCGATATAGAGAAAGCAAATGTTTTTTCGGCATACCTTGAAGGTGTCTTCAAACCATATGACACATCCAATGCAGCAGAAATGGAAACTCATACTTACGAGAATTATACCGAAATTCCTCTAATAACTAACGATGAGGTATGTGAAGAAATAAAAAAACTGAAGTCTAAGAAATCGCCTGGGTTTGATCTTATTACCAGTGAAATATTAAAACAGCTTCCTCACAACGTTATATGTAAAATTACTGCCATAATGAATGCTACCATAAATCTTCAATACGTTTCCATTTATTGGAAAACTGCAGAGGTGATTATGTTCAATAAACCTGGTAAGCCAGCGCATGAAATTACTTCGTATCGGCCCATATCTCTCCTGCCAATACTCTCAAAACTTTTGGAAAAACTGCTTGCTGTGCGTATTAACAACATAATTGAAGAGAGGAAGCTGATACCGGCGCATCAGTTTGGCTTTCGAAAAAATCACTCCACTATTGACCAAGTACATAGAATAACTCGAGTCATTGAAAATGCCTTCGAAGACAAAAAAGTTTGCTCGGCCGTTTTTCTCGATGTGTCTAAAGCCTTTGATAAAGTTTGCCATGGCGGGCTCCTAAACAAAATTAAAATGCTTTTACCAAAAAAATATTACAATATTTTAAAATCATACCTTTCGGACCGTTTTTTTCGAATTAAACATGGAGATATGTACTCTGATTTAAAGGAAATAAAGGCTGGTGTTCCTCAGGGTAGTGTGTTAGGACCCATCCTTTACCTACTCTTTACGTGCGATCTACCACATGACGACAATTGTATTACAGCAACATTTGCAGATGATACTGCGGTTCTGTCTGTCGGCAAAACACAAACTGCATCAATTGCAAGATTGCAGGAACATTTAAACAAAATATTTGAATGGACAGCTAAATGGCGAATAAAATTAAACGAGACTAAATCAGTACACATTGATTTCACATACAACAATACTAGATATATGCCTCTGTACTTAAACGGTGTGCAAATACCATATCACAATACCGCGAAATACCTAGGCATGACCCTGGACACAAAACTTAGATGGAGTGCACATGTCAAAAAGAAAAAAGAAGAATTAGAAATAAAATTCAGAAACATGTATTGGCTATTGGGTAGAAACTCTGCATTATCCACATATAACAAAATTCTAGTCTACAAGCAGGTGTTCAGACCAGTTTGGACTTATGGGATACAACTTTGGGGCTGCTCAAGCCCTAGCATTGTTGCACAAATCCAGCGATTTCAAAACAAAGTTTTAAGATGCGCCGTCAATGCGCCTTGGTATGTTCGCAGTCACGATCTTGAACGCGATCTCGGAATTGACTCAGTTGCTGCATCAATCAACAAATGTGCAAAGTCTCATATGGAAAGACTTTCTGACCATGTGAACACTGAAGCCAGGGCTCTTGTCACTCGAAGTGAATGGAAAGAAAGGCTTAAGCGCAAAAAACCGTATCACCTTATACAGTAACCAGATTCTCTGCAATTTATACGCAGAGCACCATTAATTATTTTGTATTATACAATAAGCAAAACAAATTGTTCGTTAGTTTATTTTATGTTAACTAGTTACAATGGCAATGAAATAAAAAAAAAAA

>Btry_1745#DNA/P

CAAGAATGATAGAGAAAGAGATTACGCAATGCGCATGTTTGCTTTCAGTCAGCTGTTCTTGCTGACGTCACGGTTGACTTATTATTCGCCCGCGCTTTTGAAAGTGAATTCGAATTGTGTTTTCAAGTGTTGTATTAATTGTGAAATTTTATATTTTTTAAAATGCGTTGCGCAGTGTTCGGATGCAATAATAACAATGGTAAAAATAGTGCTACGAAATGGAGATTTTTCCATTTCCCTAAGGATAAAACAGTACTAAAACAGTGGATTCATTTTTGTGCACTTCGCGCCAGAAGCCTTCGAAAGAAATTTGCAGTTTGAAATGGGTAATTATAGCATTTTGATTACAATAATTTTACTTTTATAACCAAACACTCTTGCAGGTTTAAGTTCGCGGAGTCTAACGAAGTTGCTACCTAGCGCTTTTCTCACGTTAACCAGATTAACTCCTGCAGAGGCTAATGAACGGCAGAAAAGGATGGAGTCTCGTTTAAACCAGGACGTTTTAGAAAATTTGTTTGGCGTAATCAGGTTAAAAGGCGGTCTTCACGATCACCCTGACAGGCAGGAGTTCAAGTATAGGCTGCGTTCCTATATACTTGGTCACAACGAAGGACCGATTACAGATGAAGGAAATGTTGAAGTCGACAACACCCCAGATTTGGAAGGGAATTACTTATCTCTAACCGGTAATTATCAAAATATTAAACGTACATAGTATTATAAACCTAAAACTACAGAAAACATTCTTATATAAAAATGTATTGATGTTAACAAATGCAAATATGTACTTACATACAAACTAAGTTTTTGCATACTAACCACTGTTTCTTATCCTAATTATAATAATAATCTTAACTAACAATTTATTCCATTTATTTATACGCATGTATGTATGTTTATTATATTTTTGCAGGTAATATTTTTCAACGTGTGTCAGTTGATCTAAATTCCACTGACCCAATTGAAAAAAATGATGACTTGGAGATTCTCAACAACGACGGACTTGAGAATTTGGCAGGGTACATCTGCCACAAACTCGGTAAAGACAACCCCGAAATTTGTGCCAATTCAGAAAATTGTTCGTCTTATACTTGGGTGGATCACCTTTCTGAAGGAGGACTCTCAAAACCAACAGATATGTTGATGGAGCATATGAGAGCCTTGCAAGCAGTATTTGACGACNTAAATGGTACTGATTTGCATATATGTACAAATTACGTCCAGAAGCACTTAGATTTAAGTAATTTTGTAAATTGTAGCTCGAAAGTAAAATAATTGTTTTTCAGAGCTAGAATGTATTTTAGGATTCGTTCCTTAAATAAAAACTTAATTGAACATATGTATAGCAAAAAAAGAAAATTAAATAAGACAATAAATTGATACTTGGTCACAAAATATTCAATNTTTTTATTTGCATAAATGTATATGGTTGAAACAAACACAGCAAAGAGACAAAATTAAATAATACAATGAAAATACAATTAAATGAATATGTAGGTAAATGTTCACTATAAAATATTCACTTATATTGATATCTTTTATTAATATTCACTTATATTGATGTTAACTTTTGATGATATTCATGTTTTTGTATGCCTGCAATACAAAATTGCTTCTTATTGCAAAGAAAAACAAGCAACTGAAAATCAGCTGATTGTACGTTGGTCAACCGTGACGTAGATGCGCAGAACGAACAAAATTTGAACTCGTCATTGCGCAATCTTGTTTCTATCATTCTTGCT

>Btry_1397#DNA/Chapaev-Chap3

ATCAGAAAATGATGCTGATTTTGAAGGTATGTCTAATGAGCCAAAACGCTTTTCCCAAAATGAGTTGAATGATCTTGTAAGAGATTTAAATCTTTCTAAACAAGCTTCAGAATTGTTAGCATCAAGACTAAAAGAAAAAAACTTGCTATCCTCGGATACTAAAATAAGTTTTTATCGTGAAAGAGATGCAGAATTACGGCGTTATTTTTCCGAAGAAAACGGCATTACCTTTTGTTCAGATATCAAAAACTTGTTGATAAAAATGGGCTTGAAGCGATACGAACCAAACGACTGGCGTTTATTCATTGACAGCTCAAAACGTAGCCTTAAATGTATTCTATTACATAATGGAAATAAATACGCTGGGGTACCTATTGCCCACAGTACAAAGTTAAAGGAGGAATATTGTAACATTTCGCTAGTCTTGAACAAAATAAAATACAATGACCATGAGTGGCAAATTTGTGTCGATTTAAAAATGGTCAACATTCTTCTCGGTCAACAAAGTGGTTATACCAAGTTTCCCTGTTTTATATGTCTTTGGGACAGTAGAGCTAAACAAGAGCATTGGGTGAGAAAAAACTGGCCCTTGAGAGAAAAAATGGAGCCTGGAAAGCACAATATAGTCCACAATTCATTAGTAGCTCGTAACAAAATTATCCTTCCACCATTACATATAAAACTGGGCATCATGAAACAGTTTGTAAAATCACTAGATAAAGATGGGAACTGCTTTTCTTACATTTGCCAAAAATTTCCTCAGCTCACTATGGAAAAGATTAAAGCCGGAATTTTTGATGGCCCCCAAATTAGACAACTTACGAAAGATACTCAATTTAGAAATTCTATGACTGAGTCAGAACTAAAGGCTTGGACAGCATTCGTATCTGTGATGCAGAATTTTCTCGGAAATAAAAAATCTGAAAATTATATTGAACTTGTAGAAGACCTTCTGCTACAATTAAAAAATATGGGATGCAATATGAGTATAAAACTCCACTTCCTCCATAGTCACCTAGAGAGGTTTCCGGAAAATTTAGGAGATATGAGCGAAGAGCAGGGAGAGCGTATGCATCAAGACTTACGCGTCATGGAAGAGCGCTATCAGGGTTTCTGGGATACAAATATGATGTCTGACTATTGTTGGTCTCTAATACGTCATTTTCCAAAGTCTACACATCGGAGAAGAGCTTTAAAACGAAGTTTTTTAGAACTTAATGATTAATTATTTTTCTTAATAAAATTTTTCTTAATAAATACATGTTTTTTTCTGTGTAATACCTACATAGCTGAAACTCTGAAACTAGAGCTGATAAAAATATTTAAATTATATTTTTGGAAATAGCATATTATATGTAGTCAGCAACAGCTAAAATTTCTTCGGCAACAAATGTACTGTAAACTAGTGTAATCAA

>Btry_MarW#DNA/TcMar-Mariner

ATATACACCCTGTCAAGAAAGTATCGGGAATTCTTCATTTCCCCCTCTCATCTCCCGCTTATATGGAAGACAGGTAAATTTTTTTTCCTAGGTTGGTACAACTGTCCTTAATTATGATGCCAAAAATTAGCGCGATCTGTCAACCAGTGTGTTTACAGCAGCTGTTTATGTCAGTCGACCTCAGTAGTGTTTGTCGAATTTTACAATGACCGAAAATATTGATCAACGTATTTGTGTTAAATTTTGTGTTGCGAACGAATTTTCGTGTGCCGATACATTGAAAATGTTGCAGAAGGCTTTTGGTGAATCTGCTCTATCAAAAACGCGCACTTACGAGTGGTACAGAGCATTCAAGGAGGGTCGGGAGACCGTCGAAGACATGCCTCGTTCTGGACGGCCTTCAACGAGTGTGACCGATGATAACGTTGAAAAAGTGAAGGAAATTGTTGCCGAAAATAGCCATGTGAGCTTGAGAGAGATTGCCCATAAATTGGAGATGTCACACGAGAGCGTACGTACGATTATGCATGAAAAATTGGGCATGCAACGCGTTGCTGCAAGACTTGTTCCGATAGAGCTGAATTTCCTTCAAAAAAGGTATCGAGAGCAGGTAGCTGCAGACATGCTGGATCGAGCCAATTCGGATCCCACCTTCATGGAACGCATAATTACTGGTGACGAGACATGGGTCTACGAATATGACACACTTACGAACCAGCAATCATCCGAATGGCGGCCCAAAGGCGCGCCAAAGCCGAAAAAACCACGCCAAAGCCGATCGAAAGTGAAGGTAATGCTCATTGTTTTCTTCGATATTCGTGGTGTCGTCCACTATGAATACGTTCCAACTGGTCAAACCGTCAACAAAGAGTATTATCTTGGTGTTATGAGGCGCTTACGAGACGCTATCCGTCGTAAACGTCCGAGTTTGTGGGCCAACAATTCGTGGATTCTTCATCATGATAACGCACCATCGCACAAAGCCATCATTGTGAATGAATTTTTGGCCAAACACTCAACAAATATCATTGAGCAAGCACCGTATTCACCTGATATGGCCCCCTGTGACTTTTTCTTATTCTCCACACTGAAATATCCACTTCGGGGAACACGCTTCGACTCAATTGAAGACATCAAAACAAATTCGACGCGAGAGCTGAAGGCCATCCCGGAAAGTGCCTATAAAAAATGTTTTGACGATTGGAAGAAGCGTTGGCACATGTGCATCGCTTCAAATGGATGTTACTTTGAAGGCGATAAAATAAATTTGGATGATGATTGAAAAATTTTCGTTTTATTTATAAATTCCCGATACTTTCTTGACAG

>Btry_4004#LTR/Pao

TATAAATAAAAATAAAATTGAATCATCAACGTCAACAAAAATCAACGCAACCAAAAGAAGCAAATATGAAGAATTTGCTAACACGACAGAAGGAGTTGGGAGATCTAGCTGCGTTGGAGGAGCAGAAAATCCTGAGCGGAAAAATAAGAAAGGCTTCATGTCTGGAATATATGGATCATATTCAGACTGTTATGAAAGAGTTTACTGCAAATCATAATAAAATAATGGCTAAGAAGAGTCAAGAAGCTGAGGAATACATTGCGAAGAACTACTTCGAGCATGTAATGCAAACAATGGAGAATTCTATTCAGAATTTAAGAATTCAAAGTACTGCCGAAGGTGTTTCAGCAGTGAGCAGCATGTGGCTGATTGGATCTTTGCAACAAGCTAATCAGTTCGAACCAACTGATGCTGTTTCTAACGTTGAAAAAAGATATCAACGGCGTGCAAAAAAGCTCAAAAATACATTTGACGAAATTGAGCAAAACGAACTGGATTCTCTCGAGCAAATGAGAGAATTCAAAACCAAGCTTCAAATACAATTTGAAAATGTTGAAGAATCATATTTTGAAACTCAAAATATCCAACTAAAAGAAATAACGGAAAACTCTATTGAGGAACAAATAGAGGAATTTCGAGAAAAATATACAATTACCCTCGAAAAACTTACAGAAAAATTAAAGAGGAACAATACAACACAAACAATGTATTCCAATATAAAACTGCAGGAAATTAAAATTCCGATGTTTTACGGTGAAATGAAACAATGGTCGTCATTCCACGATCTTTTTAAAAGTTTGGTGCATGATTCGAAGCACTTTACAGAAGTGGAAAAGATGTTCCGATTGAAGACATCATTAGGTGGAGAAGCTGGTAGATTAATTCAACATCTACCAGTAACAGCAACAAATTATGAAGCTGCTTGGCAAATTCTCAAGGAAAGGTATGAGAATAGAAGGCTACAATTTACAGCACAAGTAGACAGACTACTTGATCAACCGACAGCAAATGGAGAAAGTGCACAAAGCATGAAGCAGCTGTTAGATACCACCAAAGAATGCATTTATGCTTTAAAAGGGCTAAATCTAAATTTAAATGACGAACAAGCCATCATAGCTCGGATAGTGGTTCGAAAACTAGACAAAGAAAGTCTACGTTTATACGAGCAAAACGTAAAAAAAAGTAGGGATATACAAAGCCTAGATGACGTCTTCAGTTTTCTGGAACAGCAATATCAAGCTCTAGAAGCAATACGCGACAGAAAACCAACTAAATGGAACAATCAAAAGCAGCCACAGCGAACACCTACATTTTATACAGCGGCACAAAATAATTGTGTATACTGCAAGGTTCCTGGGCATCAAATTGGTGAATGCAGAAAGTTTCAGACATTGACTACGATGAATCGAAGAAGATTCATAACAAACAACAAGTTATGCTACATTTGCCTTGATCACGTATATGAAGGAAAATGTAATAATAAAAAGAAGTGCAATAAGTGTGAAAGAAATCATCATAATTTATTGCACTTCAATGAATTCCAAAAAGGAAAAGGAGGAAGTGAAGAAAATGTCACAAAAACATCTTCCACAATGATGACGAAACATTCGGAAATGGCGATACTCCTAGCAACAGCACAGATAAGGGTGAAGGCAGCAAATGGAGAGTATGTGACATTGCGAGCTCTAATCGATCAAGGATCCCAAAAAACTACTATTTCCGAAGAAGCATCTCAAATACTTCATTTACCAAGAAGAAGAGAAGTAACTGAACTACAAGGTTTAGGAAATACCACAGTGGGAGTGTCCAAATTTAAAATCAACATTAAAATCAAGCCGAGATTCCTAAGCAACGAGGCGTACAATGTCGAAGCACTAATTTTGCCGAAATTAGCGAGTGCTCAACCAGACAAAACGTTTAAATGGGATATAGAACAATTGAAAAACTACACTTTAGCTGATCCCAATTTCAATAAATCAGATCGAATCGATATTGTCATTGGAAGTGACATATATGCCGACATACTAGAAGAAGGTATATTTAAAAAGGATCGAATACTAGGTCAAGCTACGAAATTGGGCTGGATATTGTCGGGAGTTTTACAACAACCGAGAAAAAACAATACAATTTTAGCAGCGGTTACTACAACATTGGAGAAGTTTTGGGAAATAGAAGACACTACAACTCCAGCAGATACAGCAGATGATGATGAATGCCTAAGAATTTTTGAAAAAACAACAGTAAGAAATGGAAATAATCGATTTGTCGTCAATCTACCTTTTAAACAAAAAAAAGAATTAGGCGATTCTCGCAAACAAGCTATGGCGAGGTTTCTTAATCTTGAGAAAAGGTTCGCTACAAATAACGAGTTAAAACAACAATATGTGCAATTTATGAAAGAATATTCAGAAATGGGCCACATGGAAAAAGCAAGTGAAAATAAATGCGGAAAATACTATTTGCCACATCAAGCAGTGATTCGAGAAGACAGTAGAACGACTAAACTACGAGTAGTTTTCGATGCGTCTGCAAAAACTACAAATGGAAGTAGTTTAAATGACATCCTCTTCATAGGACCGAGACTTCAAAGAGATATATTTGACATTATAATCAAATGGAGACTTTGGCAATATGTATTAACTAGTGATATTGAAAAGATGTTTCGACAAATTAAAATAACAGATAGTGATCAAGATTACCAACGTATATTGTGGAGAGAAACAAAAGGAGAGCCGATAGAAGAATACAAACTACAAACAGTTACATATGGGACAGCATCAGCTCCTTTTTTGGCAACGAGAACTTTACAAGAAATTGCCAAACTATGCGAGCCCCACAACTATTTGCTTTCAAACATCATTAAAAACGACTTCTACATGGACGATTTAATGACAGGAGCAGATTCAATAGATGAATGTAAGGCCATTCAGTATGAAATATCGAAACAACTACAAAAATATGGATTTCACTTAAGGAAGTGGATGTCAAACAACAGTGAAATCATAGCAACAATTCCGGTAAATAACGCGAATGAAGTAATAAAAATAGCAGAAGACGAAACAATAAAGACATTGGGTATTCAATGGGATCCTACCAAAGACATGTTTGGATTTAATACGAATCTTTCAACGCAAAAGCTGGTTACAAAAAGACAAGCTTTGTCTGATTTAGCGCGAATTTTCGACCCAATGGGATGGCTTTCACCAACAACCGTTATAGCAAAGTTATTTATACAGAAACTTTGGTTGATGAAACTCAACTGGGACGAGTTACTTGACGAGAATTTAACAAAAGAATGGCAAGAGTTTATGCAGCATATACCAGAGATAAAACGAATAAAAATTCCTAGATGGTTTGAAACTAAAAACAACTTTAAATTTGAACTACATGGATTCGCAGATGCTTCAGAGAAAGCTTATGCAGCAGTTGTATATACTAAAGTTGGATCAGTTATAACAATTGTTGCTGGTAAAACTAAAGTGAATCCAATTAAAAATAAGAAAACGTTACCAAAACTGGAACTATGCGCGGCTCACCTACTTGCTAAATTGCTGCAAAGGATAAAAACAGTCATCAACAGAGAAATGAAAATATTTGCTTGGAGCGATTCAACTATAACGTTAGCATGGATAAATAATTGCCAAAGTAAAGACAGATTTATTAGAACACGAATAGAAGAAATCAAATTACTTGTTTCCAATGCGAAATGGCAACACGTTGGAACAAAGGACAATCCAGCTGATGTAGCAACCAGAGGGGTACTGGCGAACAAACTAATAGAACACGATCTATGGTGGAAGGGACCAAAATGGTTGCGAGAAGAGGAACATCATTGGCCAATAAAGAAGATTATTCAAATCGATGCAACGTATGTGACAACCTCAGAGGAATTACATTTCTTGGATAAGGTAATACTTAAATATTCTAGTTTAAGCAAATTAATAAGGGTAGTAGCTTATATTTTGCGATTTGTAAAAAGAATAAGAGGGAAAAAATGCCCTGAATACTTAG

>Btry_535_sTIR#unknown

ATTACACTGGCCTACAAAAAAAATTTTTTGGTTTGGTGCAGCTCTGTGCGCATCAGTAGTCGGTATAGTAGGTAGGATGAACCACATTCCCGCTACTAGATGTCAGCTGACAGCTCTAGTTTACCTACAATTCTCATTTTAATTTCCCCGTATACTATGTATGCAAGCACTTGCCAGCCACCGCATCGCGCATCACTCATACGCCCTGGCATACACTAAGTAATGTAAGCTTGTATGGTACTGTCCTGCATGAGGTCAGCCGTTTCAAATATTGGCTTCTGGCAATTTATGTGAATAAACCAATTACACTGGCCTACAAAAAAAATTTTTTTGGTTTGGTGCAGCTCTGTGCGCATCAGTAGTCGGTATAGTAGGTAGGATGAACCACATTCCCGCTACTAGATGTTTTTTCTTCTTTCTTATGAACAAGAGCTGATATGGAAAATGTGACTTGTTTTCTCGTATCAACTCACAAAATACATCCAAAATCAGGTATAAAATCTTAGGCACCAAAATGAGTGTAGGCCAGTGTTAT

>Btry_435_sTIR#unknown

TTATAGCCTTTTCGCACAGGAGTTAATTGATCAATTAACCGGCCTTTGCTCGATTAAAGCGCTAATTTAGATCGATTTACCATACAAAATAAAAATCTTATTTTACTACATTAATTCTTAATCGAATTGAAATCGAGTTGAAACTGGAATGCAACTCTGCGGGTTGTTGTCCACACTGTAAATTTGGCTGTGTTTAACTCTGCCGGTTGTTGTCCAGGCTGTAAATTTGGTTGTGTGCATTGATAAAATAGCAATCAGCTGATGAATTTTAATCATTAGAAAAAATACAAAATGAAGAACACAAGCAAGTATAACAGCTGATCGTTAATCGAGCCTATCGTTAATCGAGTAGCCGGCTGTGCGAAAGGCAAAAACTGGTTTCTCAATTATAATCTTAATGTACTAAATTAATTTGGCTGTGCGAAAAGGCTATAA

>Btry_465_LTIR#unknown

TATATATATGTACATTAGAGCGGGTCGATTTTTTTCTATAAAATCGCGTATGCGGGAAATGTTCTACGGATCATTCTGAACAACTTTGCCTAAGAGACTATGGGTCTAAAACCGGTTTCGAGCCAGCGATTTTTAAGGCGAAGTTTAAAAAATCTGAATAATCGGTTGTATGGGAGATATGTGATATAGTTGTCCGATCTGGCCGATTCCGACAAATGATCAATAGAATATCAAAATGCACCTACGTATTAAATTTCATTCGGATATCTCAAAAACTGACGAACAAATTTTTATAATCCCTAAAAAACTTCGCCTTAAAAATCGCTGGCTCGAAACCGGTTTTAGACCCATAGTCTCTTAGGCAAAGTTGTTCAGAATGATCCGTAGAACATTTCCCGCATACGCGATTTTTCCNTTTTTTTGGCTCCCTGTAAATCGACCCGCTCTAATATATATATACATATA

>Btry_456_LTIR#unknown

AATAACACTGTGGAACATTTTTGGGATTATTGTCTGGGGGGTGAGAAATTCCAAGTCAGGGTGATGGTACTAGGTCAGTATAGTAAAACCCTCAAAGGGTTTGGCGTTCGTATGTGTCAGTTTTTTTTATGACCTTTTAAATTTTTTCCTTATCTTGATGTTTTTTCGCTTAGTTGTAACATTTTGGCAAAAACGTAGTTTAACATAACTCGCGAACCACTATTATCTATTTTTCCAATCCATTAGACGGTTGTAGCAGCTTTTACACACTATATTTGGTACCCAATTTTCGTTCACTATAACATAAGAAAAAATTTAAAAGGTCATAAAAAAAAACTGACACATACGAACGCCAAACCCTTTGAGGGTTTTACTATACTGACCTAGTACCATCACCCTGACTTGGAATTTCTCACCCCCCAGACAATAATCCCAAAAATGTTCCACAGTGTTATT

>Btry_2538#unknown

CAGAGAACGTATATTATAGAGAAGGTTCAACTACGGACAAGCGTGTGAACTGTCAAGAGCTAACAAAATCCTATTAGTGGATTTCACCACTTTTGGCTCGGCAGGAGAAATTTTAACAGAAATGAGTGAACAGAGCTGAGCATTCAATGAAAATTATGCTCAGAAATATACATTGCTATTACAGACGTATGTTGGCCTTTTGCTTATATTTTTATACGTTTACATGCAATCATATCAATTGATATGAATATCATTTTGCGAATTTTTGTGAATTCATGCAAAATTGATAACATTATTATTAAATTATGCTATTTTCATGGTAATATTTGTAGTTAATGTCCAAATAAAAGCTACTTTTTAAATATTTCTTATCTTTGATAATATTATATAATTTCGTATGTATGTATATATGTATGTATATGACATACTATCATAATATCCTAAAAAACTTAATATATTACAATAATATTGTATGTAAATTACATTTCTTCATTTAAATTTTCATACGCATCTACATATTAGCACATAGTTACATACATACGTATGTAAGTACAATTCAAATTCAAATCAAGATATTATCATTTATCAGTAATATAAAAAGCGCGCGCTTCTCTATATTTACGTACGTACGTACGTATGCATNCGCGGAGACGAATAATGCGTACGCAAACATACACACGTACGTATAAATATATGCGTACGCATGTAAATATCCCAAAAAATACAAACATTCTTCTGGAAAAACAACAATAGTCTCAAAAATCATCATACATACTTACAATATGAGTACACATGTAAATATGTGCGTATGACATTCCCACACAAACAATGCATACACAACATACATACTTATATGCGTTCACATGTAGATATGTCACCCGCAAAAATTACAAATATTGTTCTACAAAAACAACAATCGTTTGAAAAAGCATCAGAAACCAATATGGCAGCCAAATTGCCGACGTCAAAATTTATAGTAAATTACCCAACAACAAAATTTTCATTCATGAACGCAAACGTACTTTCAAAAAGCATATTCGATTCATTCATAAAAGCAGACGCCATTACATCTCCCCGAGCATGCCTTGCCTACAAGCGTCTTTTCGCGACGATTTCAAAAGCTAAAAATCATAAATTGAATATATTTCTGAAATTTGTATGAGAAGGAAAAAGTGACAACCCCGCAAATATAAGTATTAAAATATATTAGAATAAAATTGACAACATAGTTTATTTGTCGATTTTCAAGTCAAGAAATGTTTCAAAGAAAATATTCAATATTCCTCTGATTTNTGTGTACAGTCAATCCCGGTTAAGTAGTACTCCGCTTAAATGAAAATCAAATCAAAATCGGTTTGTTTTTTGAAATACATAGAAATTATTGTTTTAAGTACCACTCGCTTAAGTATCCGCACTCGCTTATGTGCCATATTATATTTTTATTTTTAACAAACCACAAAAATCTGTATCTTTGATTGTGGCACATAACCGGGATTGACTGTATTTGTCAAGGAATGTAAAAAATATTTTGTAATTCTGAAATATTTTTACGCAGCTGCGTCGATATGTATGCAAGCTCACACACAAACATACATATTTACGCTGGTTTGTAGGCGAAGCTGTACAGCGGCAAGGGAAGCGGTGACAACGGCGGCCATTCGTTTATTTTTTTCGGCACAGCTCGGATTTTCTACCAACAACACAATGTTGTGACGCTTTGTCGTCGCGTCAGTCCTTCGACACATTGACTTTTTGACAAAACGTGAAGTTCGGCCATTGGTTGTCCGTGACAACGGAGTTTTTGTATGAAACAATGGGTGTATATATTTACATACAAAGTTGTGTGTAGACAAATTTACAAACATTATGCGAATGATTCTTTCAAATTTGTTTACATGAACACAAAATTACATACATACATATGTGAATATGTGAACTTTATGCGTACGGTTTTTTAAAATCTGTTTACATGTACACATAATTATATGCATATGACATGGTCTTTTAAGATGTTATCAAAACTTCTAATGCCCAAAGCAAATAAATACATATATATGTATATAAATATATAACTTAATAAACCTATATTAATTATAATTATATTAATTAAATATTTAAGTAATAATACAAATATGACTTTATTATACATTTCAACAGATTTAATGGAATTTATCATTAAATAGGTCAAATTGCACATACATACATACATACATACTAATGAGCTTTGTTATCAAATATATGGAGAATATCTATAAATGATATCCATAGNCGTTTGCGCATTGTAAATATGCGAATCTGTAAGCGTACAAGCGTACATTGAAATTAGCATCATTTTTCTCATTTAACACTGAAAATGCTCAATTCTGCTATTTTCGACGCTCCTGTTAAATATTGGTGAAATCCGCTAATAATATTTTTGTGGTGAAATCCACTAATAGAAAAGAGTACCCCTCCAAAAAATGAAATATCGTAAATTTGGCAACGCGGTGAACCTTCTCTATATTATACGTTCTCTG

>Btry_687#unknown

TTAAGGTTTTAAAGGCAAAAAAAGGATATATTTGTGTTTTTTTTGCGGCGACATGAGTAATATAATTTTATTAAATTTAATGACATATTATTGTACAACTTTTGAAGTATATTAGAAAAAATTTTCAATGAAAAATATTAATAAATAAGCCTGTGACGGTGAATCTCCAGAAGCGCCTTGAAAAAAAGTGGTTTAGCGGTGAACATTATAACTCGTTACCGGATCATCTGAAACAAAAAAATTTATATGCGTTTTAAAGGTGATGTATTTCTCGAGGTAATCAGAAAGAGTTTTTTTCTATTTTTTGCAGCACTTGGAAGTAAAAAGCGCGATTTTAGCTCAAAAAATCGCGGTTAATTTGTTAATAAAAAATAGAAAAAAAGGAAAAAAACTCCATCTGATTACCTGTTGATTTATGTGAAGAAGTAATGTTCAAATTTTCAAAAGGATCGGTTCAGTAGATTTTGAGTTCTAATGTTCACGAACTTCAAAAATAACGTTTTCGGGAAAATCGATTTGAAGTTTTTTATACATCAAAGTCAGGTATCACGGCCGGTCGTTTAAAACGCTGTAACTCCGTTAGTTTGACTATAATTGACTCAAAATTTGTACACAATATTCTCGACATATTGGTCGTTTAGAAAAAAAAAATAAAAAAAAAATGCAAAAAAAAAATTTAAAACCTTA

>Btry_959#unknown

TAATAAATATAGTTTAAAAAAACTAAAAAACACGCTTTTAAAGCATATCAAACTAAAAAGTGAAAAATAATTCTTTGTATAAACTAACAATAATAATGAAGGAAAAATTCCTGCATTATTGTGAGAAAAGCGTGGAATGCTCGATATATGAAAAATATGTCACAAAGCACCCCACGCTTTTCTCACAATAATGCAGGAATTTTTCCTTCATTATTATTGTTAGTTTATACAAAGAATTATTTTTCACTTTTTAGTTTGATATGTTTTAAAAGCGTGTTTTTTAGTTTTTTTAAACTATATTTATTTTTAATTTTTTAGTTTGATGTGAGAAACCCCAAATTTGTTAAAATATGAACTATGACATGTAGTATTGGTTAAGTAAATCGATAATTAGGCAGCATTTAATATCTACTCGTAACCTTTCATTGACTAATTGTTATATTATTTGCGACCAAGTTCTATTGACGACTTTACGAATTATTACTTCTTAATCGAATCATTTCTTCTAAAGCTTCACTGTTACATGGGGTTTTACCTGTCAACTTTGAACAGTCTAGTTCTTCAATTCGAATACCGTCCAAAGATCTACAACGACTAAGTGCTACATAAGCTTGTCCAGCAGCAAACAGTCGAGAGCCCAGATAAATTACTGCATGATCTACTGTACAACCAGAGAACGTATATTTATAAATTTATAAATATACGTTCTCTGGTACAACCTTGAATCTTATGAACGGTCGACGCCCAACTCAGTATGCTATTCAAATGTCACCAAAACTAGTTTTATATAGATGTTGCATACTTTTAAGCGCATCTTTTTGGTGAGCTGCTTAAACTTACTATCGCCTTCCTACAGTGTCGCTCAAATATTCTAGGTTTCAGTACCACTTAAAAAAGTTACAAACAAACATACATACATACATACATACAAGTGAAGCTAATAAAAGCGTATTAAAAAA

>Btry_117#Unknown

GATGTGATGTGATTTAAAAGTCGCGAGGTGACTTCGCGGGAAGCTGGCTGCCAGCTGGCTCTATATAAGAAAAGCAGGCACCGTGCCTGGCCAGTGCTTAGGTGACTCTCGCGCCGC

>Btry_106#Unknown

TTGTTGTTGTTGTTGTAGCGGCAGAATTCTGCCGAGTTGACAGTCCTTGGCCGGATAAAAATCCGGGTCCGTTCCGGTTACGTAGACCCGACTGTCGTGGGAACGG

>Btry_671#Unknown

ACAACAATCGTTGTGATTTACGTGAGTACCTGCCGACGACGGCCTTACCTCACGGTGCTTTCTAGAAGCACATAGATCAATACAATGCGTTGATCAGCGCCCCAAAGTATAAATGCATTTTGCACGTATATTTTGGTTCCAATTGGTTCAGGTGGATAGGGACACCCTAGCCGCCTTGGTCGGGTTCGAGGCCGATCTAAAACCTCTGCCGTACTCTGGGTCCGGCACCCGGCCGCAGTGCGACTCCACATTGGATTGAATTTTTCAACCCTGCCTGCATTTAGGTTTAAACCACAGCCACCACGAATCTCCACAAAGGGCCAAACCCAATGAGCAGATTGGAGTAAACTCCAAGGGCTAACTGCTCCCCGTGGTACCAGGTTAAAGTCTCTGGTAAGACCTTGTAGCTTTTGCTACTACCAGTTGAGCCCCCAAAACTCAATGACTGGTGACATTTGTCGCTTGAACAACAAATGTCTTTATATTAGGAATTAATATAAAGCCTATAATATCATTTCAAGCTGAGTATTATAGCACTATTTCTGACATAGACTATGCTGACATTTCAAGGCAAGCCCAGAAAGAAAGACATAGTGCATATGGATTTTAGTCGCCTCTTACGACAGGCATGCCTTACCGCGGGTATATTCTAACCCCTACCCGCAGGGGGA

>Btry_703#Unknown

CAGTGGCGTAGCTACAACTTCGGGCGCCCGGGGCCAAGGATGTTCTGCCGCCCCCCTTTATCTACCTACCTACAAGTTTCATGAGGTGGTTCGAACAAAAGTGAATTTTTACAAAATATCTTCGATATTAAGTATATAAAATTTAGGAACTAGAAGCCACGCAAATTCTGAAGTTCCAAAATTCTCACAATACGGTTTTTGAGGAAATTGATAGTAAATTTACAAGACATCTTATTAAAAGCCATAGAAAAAACCCATTTTCGCCCTATATCTTGTACACTTTTGACACAATTTGCAGGAATTTTTGCCCGAATTGGAGTTTTTAAATACCATTTTTGAAAATTTGGAGAAATAAAAGTATTTGTTACATTCAAAGCATAGGGTAACTGTGAGAGTGACACGTCGCTAGACAAAGCTAGAAAAGTGCATCTTTAAGTTCTATCACTATTTTTAAGAATTAAGCCAAAAGATATAAGACAAATTCAAAGACTGAAGAGTATTCGAGTAAAAATTAATATTTTTCATTGTTATTCAGATTATTACATTGTGAGTGTACAACTCTTTATCTTTTATAATAAATTTTGTAAAAAAATTTGTTGAAGTATTTTTCTGAATATTAAAAAACAGCGAAGATCGTTTTGCCGCCCCCAAGACGTTGCGCCCGGGGCGACGGCCCCCTTGCCCCCCCCTAGCTACGCCACTG

>Btry_1667LITR#unknown

TATATACAGTGGACCAATAAAGTTTACATACACCTAGTTCTATTAAATTACGACACGTTTATTTGTTATAAAATGAATAAATTTTGTGGTTTTTTTCTATCCATTTCAAAAGATGTATATTTAACATTAAATTTAGCATATCAAAATATTTTTTTTTACACAAATAAAAAAAATTAATGCTAAAGCTTAAAATGNGCATAATTTATATCAAAAAAGTTTACGTACACTAATGATTATTTATATAAATCAAAAGTAATTACAATAGTTTTAGCGGTTTTTGGCCTACATTTTGTTGCTATTATTGTCCCTAGACATCGTTGTATGCTCCCCACTCAATTTCTAGTATTATTTCCGGATATTTTGACCCACTAAGTCCATGATCCAGCTTTTTGGCCTTATTTTGATGAAATTCGATGTTTTCGAATACGGTTTTCCAAAAAGTTCCAGATATTTTGAATAGGATTAATGTCTAGTGATTGCGGGACCTATGGAGTTATTTTTATTTCCATAACAACCATTCATGTACAAGATAAGACATGTATTTTGGGTCGGTATCCTGTTGGAAATTGGTTTTGCTGCTATTAACAGCCGATTTAAGCACACTTAAAATTTAAATTTTTTTCCTAAAATGTTCAGATACATATGCTTATCCATGTTTTCACTAATAAAGTCCAAATGTCCAACTCTAGATGCAACCATACACCCGCAAACCATAACATTACCCCCACAGTGCTCTACCATTGGCAATACATTTTGTCCTTGCGACTCTTCATTGATCTTTCTCCACACTTTTCGTGATCTATTCGAACCAAATTTATTAAATTTTGAATCGTCGGTGAACACAATGTTTTCCCAAAACCAATACGGTTTGTTTAAATATTGTTTAGCAAACGCCGACATTTTCTTTTTATTAACCTTATATATGTATGGCTTACGACGCGCGACTCGTCTATGATAACCAGCACTCTTCAACAAATTCCTTACTGTTTGGGGATTTATTTTTTTGTGTACATTTCAAAAATGTATAAGCATATGTATCTGAGCATTTTAAGAAAAAGATTTAAATTTTAAGTGTGCTTAAATCGGCTGTTAATAGCAGCCATTTCTATTTACCACAGGATAACGACCCAAAACACACAACTTATCTTGTACGTGAATGGTTGTTATGGAAATAAAAATAACTCCATAGGTCCCGCAATCGCTAGACATTAATCCTATTCAAAATATCTGGAACTTTTTGGAAAACCGTATTCGAAAACATCGAATTTCATCAAAATAAGGCCAAAAAGCTGGATCATGGACTTAATGGGTCAAAATATCCGGAAATAATACTAGAAATTGAGTGGGGAGCATACAACGATGTCTAGGGACACTAATAGCAACAAAATGTAGGCCAAAACCCGTTAAAAGTATTGTAATTACTTTTGATTTATATAAACAATCATAAGTGTACGTAAACTTTTTTGATATAAATTATGCTCATTTTAAGCTTTAACTTTAATTTTTTTTATTTGTGTAAAAAAAAAATATTTTGATATGCTATATTTAATGTTAAATATACATATCTTGAAATGGATAGAAAAAAACCACAAAATTTATTCATTTTGAAACAAATAAACGTGTCGAAATTTAATAGAACTAGGTGTATGTAAACTTTATTGGTCCACTGTATATATA

>Btry_sTIR#unknown

ACAGTGAAATTCCCCATTACGGACAGTCCTCATAGCCGGACATCTCCCATAACTGGACAAATTTCATGAACACAACGTTTTCATTATTATTTTCATACAAAAGCAATTCCTATAACCGGACATGAGTACGTTCCAATGACCGGACACGGACACTATTTTGGTAATATTTCGTTCAAATTATCTCTGATAAACGGACAAGATTTCATAAAATTTGTCAAGAAAATTGTGTACAATTTATAGTTCCCGCTTTTTTAAATTCTTATTAATTTTTTGTTGTTTTGAGACAGTTTTTTTAAGAACAATACGCAAAATGATCACAGCAAACAAATTGACTTGCGCTGGCGCCGTCTGGTTCGCGGTGGCTCAACGGAAACTAATGGGATAATTGTCATTCATTCACTCCTGGCTGACATTGGCTCGCATTTATTAGTTCAATAGAAATATAGTTCTGAGTTGCAGCTTCTGACTCTAAGAATACATTTTAAACACTCGTTTTTGCCACGCCCCCATATATGGACAGCTCCCTTAGCCGGACAAAAACACTGCGACGGTGGGTGTCCGGTTATGAGGAATTTCACTGTATAT

>Btry_766#Unknown

ACATTAAAGGAAAAATAATTTACGGGCGATCTGAGGTCGGGAAAAGGAAGAGGAGGGGCGTGGTTGAATTTTTAAGGGTTAAGAATGGTTTATCTCGATTTCCGGCAAAACTACGAGTCCTATAGAAAAAAGTTATATGGCAATGTTGTAGGTAATGAAAAGATCTATAACTTTTGTATACAGACTTTTTTCACATAACCTCAAAATTTATGTGAAAAATTCAAATAACCAAGTTTTCGGTTTTTTTATTTTTATCTTTTACAAAAAAAATTTTCACGAAATTTGGTGCAAACTTACCTTCTTATGTCCCAAACACACTGTATTTTTTATTTGAAATATTTATTATTTTTCCTTATATCGACTTAATATCCGAAAAAGCCGAACTTTTGTATATAGACTTTTTTCACATAACCTCAAAATTTATGTGAAAAATTCAAATAACCAAGTTTTTGAGTTTTTTATTTTTATCTTGTACAAAAAAACGATTCTTTCACGAAATTTGGTGTAAACTTACCTTCTTATGTCCCAAACACACTGTATTTTTTTATTTAAAATATTTATTATTTCTCCTTATATTGATCTTATAACCGAAAAAGCCCTAATTTTCAATCAAAAANTCTCAAGTCAAAATATAATTTTTTTTAAGTAGTAGGCTTTATGTTTGTTGAAATCTCTACTTTCTGATGGTATAAAAAATATATACATTACTATGGTAAATTTTCTCAGAAAACGCAACAAAAGACACCACTATCCAGCAAAGGTCTTCA

>Btry_603#Unknown

TTAAGGGGTCAGTAGGGTAATCTTTTTTAATTTTTTTTTTTTGCATTTTCTGTTCCCTTATACCCTTAGAATTAATGTAGAAACCCACTTTACCATTGGAAGGTTTCGAAAAAGGCCCAAAAATAATAATACCGCTCGACGTTCGGAGCGCTCGGAGTGCACACCTCAAACTTTAAACGCGTTTTTCTCAAAACACACTTTTTTAAACTGGCGGACATGATTCCGGTCGAACTACTCAACCGATTTGCTTAATTTTTTTTTTAATGTTCACAAAACGCCTGGCTATCGTCCCTACTAGATTCATAATTTTTTATAATTTATTGACAATGTTATGACAAAATTTATGTAAAAAAACATGGGGAAAAATTAAAAAAAAACGTTAATTACTTTTTTATTTATCAACATTTTTTCATGATTCTAGTAGGGACGATAACCATTCACGTACTTTTTAAGAATAAATTGGGTTTTTGTGTTTCAGATGATCCAAACATGAGAAATCGTGTCCGCCAGTGAAAAAACGCATCTCATTCACCCAGCCATTTCTCCGACAGATGTCATCAAAAAATTCCGAAAAAATTTGTTTATACACTCCACGATATACCTCATGATATGTGAAAAAAGTTCTATTGTAGAATAAAAATTTCTATGAAAAAAAAATGTCAAAAAAACAGGCCAGATTACCCTACTGACCCCTTAA

>Btry_723bp#unknown

TAAGGGGTTATATACAGTTAGAAGGCCGAAAAAAGCGAATTTTCCAGAATTTTTTCTGAGAAAACTTTTAAATTTATTGATCCAAAAATTTGTACACATATTATGGTATCTTTTAACTGTATTTTAAGACTTAGTATTAGTAAAAATATTTATTTGGAAAGGAACTACAGCTGATCTCCGGGAGCTCCTCTCAAAAAAGACGTTTTGCGGTGACCACTATATCTCTGAACTGGATCCTCTGAAATTAAAAAACCAAACAGATTTCGTTAAAGTAATGTTAAATCTAGTAAATTAATCGAAGGAATAAGCAAAATAAAATTTTTTGACAAAATGGCGGTTTCTAAAAAAAAATCGATTTTTGACCAAAATTTCGGCCTTAAATTGTTTATAAAAAAATATTTATCGGTGAGAAAAAATCTTCGATTAATTACTAAAAAATATATTTAAGAAGCTCGTGTTAAAATTTGAGACTAATCGGTTTAGCCGTTTTCGAGTAATGTTGGTCACCGACTTTGAAAACACCATTTTGAGAAAAACGCGTTTAAAGTTTGGATTGTACTTCCAAGCACTCTGAAACGCCTTTTCAAATTTGCGTGTAACTTCGAAAATATTCACCGGAACGATATGAAATTTTCTGTGTGTATTCTTAAATATATGTACATTAAGAAAATAAAATAAAAAAAATCGATTTTTTGAAAATTCTAACTGTATATAACCCCTTAA

>Btry_1663#unknown_includesHel3_fragment

CGCACATAGGAGCATTTGGTCTCAAAAACCGGACAAAATTATTTAAAGTGATTTTATGAATGAAAATGAATAAAAATGCATGATTGAATATAATAATGCAATATATTAAAATAAAACAAGTAAGGAAGGACTAAGTTCGGGTGCAACCGAACATTTTATATTGAATATATTTGAGTAGGGGGTAGTGCCGACCAGATTTTATCTATTTTTGCTCATCCCACATACTACTAATATGCCACATACTCAAAAAAATGTTCCCTCGGTTTCATTTAGGTACCTTACATACAATCACCAATCATATGGAGCAAGGTCAAGTGGATGTCCGAAAATCTTGGTAATAGTTATATGGGGGGGGTTGTGTTGGAGGGGTCAAGTTTTCGCGCAATTGTATCTATTTTAGGCGCAAAGATACACTGTTATGAATAAAACATGTTCTGCAATTTTCATTGAGATTGACTCAAATATTGGCGGATATATCCAGCATAAAGTGACCTAGAAGTTCTAAAATCTTTATATTAGGTATATGGGGGCTCAGGAAAGTATTGACCCGATTCAACCCATTTTTGACACAATGAAATACTATTGTCAGAAAAGGATTCTGTGTGAATTTCAATTTCATATCTCACACATTGAACGATATTTTCGGTCAAAAGTCAACTATTGGTATCGGGTCCACATATTCGGTACCTAGGGGCTTGAACAGTTTTGGTTGGATTTNGACAATTATTGGTCAAAAGGTGGCTTACTTTAAGGGAATTATTACCGCAAAATTTTATTCCGTTATATTAATTAATTCTTTATTTGTGTACTGGAAAGTGAAAGAATCAAATGGAATTTTAAATTGTGTTATATGGGAAGTAGGTGTTGTAGTCCGATTTCGCTCATTTTCACAGAGTNACATAGGAATATGAAAANAATGTTACGTTCCAAATTTTGTCGAAATCGGTCGTCGTCCGAGATATGGGATTTCACCAGAAAGTGGGCGGTGCCACGCCCATTGTCCAATTTTCACACCGGCTCCGATAGAGCCCCCTTATACCATCTATATACCACCCGTATAGAATAATTTAGCAGGATTTCAGTATTTAATAAAAAAAATAAATAAAGCAGTACTCATTTTATGCAAGTGACCAACNTAATTCAACGCGCTTTTTTTAAGGGGGGGAAAGACGGGGAAAATGTTTAAGCTTATATGTTAAGTAGAAACCCTGTAGTTTACTAAGAAAAATAATTCATAGCTCGACCTCGCCCGACACTATAACAAAAACACTAATTTCTAAAAAATTAAAAAAATTAGTTTTTTTAATGATTCATTGTTTTCCCGAAAAAAACAAAATAATACATTAACTAAATTAATCTTTATATCATTAATATTAAATACATACCATGTTGTCCAGAATCCATAAAAAATATTTTCCAGAATTCACTTCACGCAACACAAAAAACCACTTGCAATTTCGAGAACAATAGAGAGTTTCGCGAATAAGCACGTGCGTTCTGAACGAAGTCTAGGTTCCGACTACTTGTGTGGCAATATAGCGGNCTGAAGGTCATTCATTTAATCGGTTCATTGTAGAACGGTTACTTAGGTACNAAAAAAAATCAATTTTGAGTAGCATCGCTGAAGTTTGTAATTTTCAATTTTGTCCGGTTTTTGAAGGAAAATGCTCCTATGTGCGTCGTT

>Btry_605STIR#Unknown

GAAGTGCTGAACACATAGAGCGCGACAGACTGCAAACTACATCTGTCAAATATTAAAATACACACGTTCTTATGGACACTGTTATTTTGACAGCTGCACGACTACACGACTGCAGGCCGACAGTTGTCGTTCTCGCTAACTTCAATATCCTCCTATTTTTGCCAACGACAGTAGGACGACAGTAGAGTTGACAGTAGAGAGATGAGGTAGAGTGGTGATGCCACTAATATGTAAAATGTAAAATTATTCACAGCTCTAACAAACTTGACGTTATTTTACAAATAAAAGCATAAAATAGCTCTATTATATCATTTGTAATAACAATTGATAATTTAAAGCAAAAATATTTACCTATTTACTTATTTTTTGCATAAAAAATTTCACTTTGAACAAAATATTAAAATTTCATTGAAGTGAAAGGTATTAGTATGGCCACAACGAAATTGTGTACATACAAAATGGACAACTGCGTTGTTTTGTGTACATGCCGGCCATTGTTATGTCGCTGCCTTCTTACAAATGTTCATGTAGTAGGATTCACGACGCCATTTTCAGTTCACTGTCGTGCTGCTATGTCGTGTCGCGCTCAATGTGTTCAGCACTTC

>Btry_746STIR#Unknown

TTAAGGGGGTATTCTCATGTAATCACTTCGAAAAATCGATTTTTTTTTATATTTTGACAGTAAAACCTATTGAAAACATGCTGTGAAAAATTCAGACCGAAATTCATAGTATTTGATAAGTTACAGCTCATAGTCGCCGACGTGTCGTAAGCGACTGTCTACCAGGCGCCATGACAAGTCTGCAGCTGCTACGTTTCTAAACGCATTTTTCTCGAATCATCATTTTCTGAAACTGTCATGGTCCTAAAAAAAAAGTATTCAACCGATTGCTTTAAAATTTACATATGTTCTTCTACTCATTTATAGTTATCGTCCCTACCAGAATTGTTTATTTTCGAAAATATTTTCATATTTTTTTAAACGATTTTTAACGAAAAAAAACAAGATTTTCGTGACTTTTTTTTGAAGTTCGACATTTTTTTTTTTAATGAAATTGATTATATTTGGTAGGGACGATAGCCGCAGAACTACTGAATAATAATCCATTCGATTTTTTTATTTCAGACAACATGAACAGCCGCTATCACCATGACCAGTGAACTACTTTTTTTTGTTGGGGACTACTTTGATTTAAAAAAAAAATATATTAAAAATGTAAAAAACGAAAAAAAAATTTTTTTTGTACATTTCAAAATACAAATAAAAATATATTAAAAAATTAAAATGATTGAATTTTGTTGTCGCATAAAAAAAATTTTCCTAAAAAGTGGTAAAATGTATGCGTTCACATGAGAGTACCCCCTTAA

>Btry_585sTIR#Unknown

CACTCGTGGCCACGCAAACCTTACCACTCAAAATTTTCAAGTATTGTGCATATTTGACTACATTTTATAGCGGTACTTTTTGCGGTATAGAAATTTTTTATTTAGAAATAAGTTAATGACATGTTATTAATCAAAAATATTAAAAATTAAAAACAAAACACATTCAGCTATAGATAAATCAATTGAAAGATAGTGAGCTTTACAAAATGAGCTCGAAATGTAAGAGACTGACATGCGATGAAAAAATTATATTTTTTATTTTTTTAATTTAATTTCATTTACACTATATTTGAAATTAATATTTTATTTTTTTTTTTTTAATTGAAACACGAAGTTTAGTAGGTGTATCACTTTAAAAACAATAAAATCGGCCGCTTTTGTATAAGCACAGAAAAGCTAATTTGTGTTTTACTCATTAAACGTAATAAATTAAAAACTATAATTATAATTAGATAATATAATTTAGTTTACTTATGCTTACTAAAAACATAAAAAAAACAAAATTGAAGAAAATCCCGAACGAACGAAAAAGTCCAAAAAAAATTTAAGAAAGTATAGTGGTAAGGTTTGCGTGGCCACGAGTG

>Btry_1100LTIR#Unknown

GGCTGCGATTTAGAATAGCATCCCCGATTTCGGGGTTAAAATGGGTATGTACGGATAGAGGAGGTCCTCCAGATCAAGAATATATATANATATAGTTGCCGCTGACTTATGGTTTTTGAGATATTTGCATTTAAAGTTCAAAAAATCAACTATTTAAAATGCGTGTTTCTCCGATTTATAATGCATTTTACACTTATATTTTTAACTTTTATATCCACTAACACTTCACTAATTCGTTTCTAATCATTTATTTTATATTAAATAGTGCAAAAATAACTTTAATTCCATATCAAACTTTAGTTAAAATCCATTTATTCATAAAATAAGAAAATGGTTGTAAACAAGCAAAAAATTAGTGTTACCATATTTTGTAATGGAATAAAAATAAGAAAAAAGAAAGATTATGACGTCATAACTAAATTCATTATCGTGTGATGAGTAATGTAAAATCTTTCAGAATTCTCATTATCTTGATAAATCCCGGTGTTGGATCGGCCAGGGTTATTTTTTTTGAAGCGCGGCCGAAGGCCGCCAACGCAAAAAGGAGTTCAACGCAAAAGAACTGAGCACGCCCAGGTGTTGGTCCGACCGAGGGTTATTTTTTTGAAGCGCGGCCGAAGGCCGCCAATGCAGAAAGGAATTCTATGCGGAAAACTAATGTGTTATATATTTCCTGTCATTGGGGTATTATTTGTTTTCTTTATTTCTTTTGGTTCTTCTGTAAAATATGAACGTAAGGTAGCACTGATTATTAGTTAGAATGCAACCCTTTTGTTATTGTGTGAACAAAAATAATTGAACAAAAGTTAAATGTTGAATAAAAGTTATTTTTGCACTATTTAATATAAAATAAATGGTTAGAAACGAATTAGTGAAGTGTTAGTGGATATAAAAGTTAAAAATATAACTATAAAATTCATTATAAATGGGAGAAACACGCATTTTAAATAGTTGAATTTTTGAACTTTAAATGCAAATATCTCAAAAACCATAAGTCAGCGGCAACTATATATATATATTTTCTTAATCTGGAGGACCTCCTCTATCCGTACATACCCATTTTAACCCCGAAATCGGGGGATGCTATTCTAAAATTTACCC

>Btry_1290sTIR#Unknown

GCTTAAAGCGCCCATACACGAGCACACAAGTGCGTTCGATCGGTATGTGTGCGCTTGCGGTTTATCGTGTATGGGCTTGTTCGCGTACCGATCCATGTTCGCGAAAATGTTTGATTTTTTACGATCGGTGCGCGAACATGTTTATCGTGTATGGCGTTGTCGGCGCACAAAATCTCATTTCTCTCGTGTCGCCGAACAGTGAAGATCGCGGACAATGGCAAGTGTTAAGGGGAGAGCCTGCTTTCGAGGCTTCAAAAAATCGATTTTTTTGAGGATTTTTTTGGGAGGGAAAGAAATAATTGATTAAAGCGAAATTTCTAGGGTTTATAGTTATATATTTAAACATCATTCACAAATTTTTTGGATACGAAATCTTTGATATTCTGCCTTTGGGAAGCAATTGCCCGATGCATCTCGCATGTGCATTTGTCGGACGGCGGGCAGGATGCAGGTCGCAATTTCTATCGGAAACAAAAAATTCAAAGAGATTCATATTTTTTAATAATTTATCTTCTAGTTAAACTAAGAAAATTCCAAAAAAAAGTGTAAAATTTTACAAATTTTTTAAAAATTGAAAAAAAGTGGTTTTTGACCAAAAAAATTTTACTTTATGTTGTTTAAAAATATGTTCAAACTTGACTTTTCTTAGTTTTTTTAGTTTAAATAGAAGATAATTTAATGCCAAAGATAATAAACTTTGCCCCAATTCCATACGACGTTTAGTTTTTTTTCTATCCTGCCCGCCAATTAAGAAAAATCGTAAAAATGAAAAACCGAGAAATCGCGCGTCAAAGTTTTCGCTTTCTGCCCAAGCGCTCATATATCTGCTAGACGCTCGGTCACTATTTCCCTTCTAGCTTCGAAAATATTTCGAATTCCCATCTATAACTTTGGGGACATATTCTTAGATAATTTTTAAAGAGATTTAAGCAAAAAAAAAATCGATTTTTTGAAGCTTCTAAAGCAGGCTCCCCCCTTAATAAAGATTTCGTATCAGAATTTATTGATCTTATAAAAAATGAATTGGCAATTTGGCAAACGAAAAGTGATAAATATAAAAATAAAATTGAGAAAAATAAAAGTTGGAATAACTTATTAACGAAATACAAAGAAATAGATAAAAGTGCTACATTAGAAATAAATTCCAAGAAATGTTCCGCTTGCCTGTCGCACATGTTAACTCTGTGAAGAACGAACGCAAAATGGATTTGTGTGTCGTGTATGGGCAAACGAATTCATACCGATCGAACGCACTTGTGTGCTCGTGTATGGGCACTTTTAG

>Btry_500sTIR#Unknown

CTAATACCATGTTCAGACCGAAAATTTAAAAGATTAGATTATATTTAAGCATTTCATAACCCAACAGTGTTCTCACTGCCGTTAGAAAGATCAAAAAACAGCTGTTATTGTCATTCAAGAATTCACATCGCTTAATATTTATCAAAAGAAAAGATTGTCATATAGTATTTTGAAATAAAAGCCGTATTCTTTTAAATATTTTCCATATAATAGAAATAATGGATGCATTTTTTCATTTGTTAAGTCGATTTTCAGNTGAAATTAGATTCATTGCTTTAAAAAAAATTTGTTTGTTTACATTTTTTTCCCTTTGTAGTGTCTAAATCAGCTGTGATAATGTAACAGATTTCCAGTGCTGACAAGTAGATTGCGCAAAATCAGAGTCGCACAGAGAGATTTAGCGTGGATCAGTTTCAAATCATTTCAATGAAGTGATTTGGAACTGTCATTTTTGTATGGCGAAATCTTGATAAATTTTTAAGATTAGTGGGATAATCCATTCAACAGCCGAAATCGTGTGATTAAGTGTCGGTCTGAACATGGTATAA

>Btry_722sTIR#Unknown

TTAAGGTAGTAGTCTGGTAACTTATGCCTATTTTCATGACTTCTTTGGAGGGTGGTTTTTTATAGGAAAATAAAAATGAATTATCTTGAATATTTAGAGTGTTTTTATTTACATATTGAAAATATAAAAAAAAATTATTTGAAAAAATTTAATACTTTATTACTATTATTATTGTCACTCGAAGTTGGAACCTCAAAAAAAATGCACGTGGGTGGCCCGGGTGATTTTGGCTCTTGATGTTATCTGAAATCAAATATTCTTGATTATTCTTAATCTATAGACATGTCATTCTGGTCGGAACTACTGAAGTTAAATTTCAAGGGTAAATAACAAAATGGCGGACTTTGAAAAAAAGTCCTTTTATTTTAGCAAAGAAAGGGTTGTAAAATAAAAAGTTAGGGGTGAAAAAAATTCTCGTTAGTACCGACGAGAACTATAAGTGTGTAGAACAGCCTGTTAAAATTTGAAAGCAATCGGTTCAGTAGAAAAAAAGTTAGGACCCGGGCCGACCAGAAAAACAATGTTTTGAGAAAAACGCGTTTAAAGTTCAACAACTCCGAGAGAGAGGACTCCGAGGCGCTCTAGGAAACTCGTTATAACTCCCGAAATATTTCGAATTTCTGTCTGAAATTTTCACAGTATATTCTCAAGACATTGTACTTTCAAATTAAGCAAAAAAAATTTCGATTTTTTGAACCCAAGTTACCAGACTACTACCTTAA

>Btry_711sTIR#Unknown

TATACACCCAACTCTTTTTTTACACGGTAGATACGTTCCTCAGAAATCCGTGTAAAAAAACCGTGTAAAAAAAGAGGCGTTTCCTATAGTAAAATGGGGGATACGTTCCATGTCATCTGAAAACCGTGTAAGATGAAATAAAGAACTATATTTACTTCGAAAAACCGTGTAAAAAATGTTGAAAACTATAAAATTTCAGATATGCATACAAATTGTATGTTCATATGGCATTTTATTGAGCATTAAAACTTAAAATACATAATTCATACAGGTGAAAAATTGGTAGATTAAGCAAAAAACAAAAGAAATCTGTTAATCCAGAATTAAGAACAGAAAAATTAGAATAGAAATAAGGAAAAAATATTTACATAGCTACATAATTATTCGTCGCTACTTGATAACAAGCGCAATTGTTTGCGACGAACTGGACTAAAGTCGGTATCATCGCTGGAGATATCCATTTCAGCAATGTAAATATCATGCGAACAAAATGTGTAGGTAAGAGTTATCGAATAAGGGGATTTCCCAGTTACTACTATGTATGTTCATTTCAGCAATGGTGCTAAGTGTGGTAAAAACAAACATAATTACAAGACAGTGATTTTGAAACCGTACTAGATTAAAAATTTTTTACAAAAAAAATTTTTTTTCAACCGTGTAACTTCAAATCCGTGTAAAAAGAAACCGTGTAAAAAAAGAGTTGGGTGTATA

>Btry_717sTIR#Unknown

TTAAGGGATCGTCTCAGTGTGACCCTCCGAAAAATCACTGATTTTCGCGATTTTTTTTAATGTTGAAATTAACTAATCGGTCCGNTTTTTTTATAAATAAATACATTCATGACGAATACAAAATGATTTTTTTATGTTTATTTATTGGGTGTATAATAGTTAAATAAATTGTTGAAATGACACGTAAAAAAAGGTCCTGTACGATGTCCACGATTGCGGCCGCAATTTTGATCTAAAACAAAAATTTCAAAAAGATTATTAATCTGTAGGAAAGTCGCTATCGCGCTATGGAAGCTTTTTTTTTTTTTTGAAATTTGACAAAATGGCGGCGGTTTGAACAAAAAGTATCGAATTTGGCCCTTTTTTCGACAGTTTTTCGTAGAAAAAATAGGAAGATTTCAAAAATAAAAGCTTCCATAGCGCGATAGCGAATGACGTTAAAAATGTTCTCTCCAAATTGGAACTAGATATCTTCAAAACTCTTCCTTTGAGAGTGGAAACCGTTTTGAAAAACACCATTCTGAGAAAAACGCGTTTAAAGATTGGAGTCGCTATGTTCCCCACTCTGTTCCCTTTCTACAAGAAACGCTGTAGCGACCGTAATAATTTGAATTTCGATTTCAAAATTTGAGAGAATGTTTATTACAGTGTATACTATCCGATAATGCAACAAAAAAATCGATTTTTCGAAAATTTCACACTGAGACGATCCCTTAA

>Btry_1327#Unknown

TCGGCACAATTCCGATTTCTTTGGCGCCGCGATTTGAAGGTACCGTTGGAAAGAGGAAGTCCTCCTGATTAAAAAATATATAGGGTTATAGGTACCGCAAACGCTTAGTTTACGAGATATTCGACCTTAAAGTTCAAAAATTCCCAAAATTCGAACATTTCAACAAGCTATTTTACCACATTAAACACACTTTACTCACATTTTTCACTTCAAATTAATTAAATTAAACTATAAACTTTTAAATAAATCCACATTTTACACTTTTAACAGTTTTTTTACCGAAGAAATCTTTATTTTAAAAATTACATTTTACACTCGTAGTGAACATCATGTGTGTGCTAAAAATTACGACGAAATGGAGCGCTGCCATGTGATAATAAGGAAATTCGCTGAAATGCCTTTTTTCCCAAAAATTCCTCTATCCATTCATATGGATATGTTCTTTATTTAATTTAATAAACAAATAAGTAATATTATATAGTAATATTATATAATAATATTATATATATATTGTCACCTAATATACAAAACAACGTATTATCAAAAATAAATGGAAAGCGCTGACAGATATAATAACCAAAATTTAACCATTTTATAACGAAAACTCAAATTTTGTTTCAATATCAGTGCAAGATAACCAAAAAATATAACCACTTTATAACGAAACTCAATATATTTTTTTTATTTTAACGCAGAAAGGAGTACTATGCGAAAGTACTTCAGTCACCCCGGGTATTCGCTCGACCAGGGTTATTTTTTTAAAGCGCGGCCGAAGGCCGCCAACGCAGAAAGGAGTACTACGCGAAAGTACTTCAGTCACATAAATCACATATTACACATATACATATGTAGAAAGTATTTTTTTATAGTTAATATAGAAAAAAGAATCACGCGAAAAAACAAACAAAGAAAAATTGTTAAAAATCCTACATATTTGCTGTTTAAGATGTGGCAAGGCTCGTTTTCCTCATAATTGTAAACAATCTAACCTTTTCAACGATGAGTGTGCTAAGTGATTTTTAAATTAATAAATTTAGGTAAAATATAGCAAAATAAAAGTGAAATGTGAACTTATTTCAATGTTTAGAGTGTATATTTAAATATACAGAGTGAAAAACGTGAAAAAATTTAGTGAAACATAGAGAAATACCATGTGTTATTAGTGCAAATTTTTGAACTTTTAATCGTGAATATTTTAAAAAATAAAAGTTATTTTTATATTATTTTTGGATATTCCTCCTCTGGAGAAGCTCCCCTATCCGCACATACCCCATTTATACGGAAATTCGGTTTTTTTACCCCTCCGCCAAAGTAATCGGAATCGTGCC

>Btry_Mar_fragment#Unknown

AGTTGCCCAAAATTGGACTTTCCGAATGGACCACCTAAGACGCAGCCGCGGTCAACATTTAAATGAAATTATCTTCAAAAAGTAAATGTCATGAACCAATCTAACGTTTCAAATAAAGAACCGATGAGATTTTGCAAATTTTATGCGTTTTTTTTTAAAAAAAGTTATCAAGCTCTTAAAAAATCACCCGATATAT

>Btry18429#Unknown

TTAAGGGGTTACATGGGTTTACGGGTTTCAAAAAATCGATTTTTTTATTGTCTTATTAAATTCTACAACACCTCTAGAATATTGTCCTAAATTTTCAAGTTGATCCGAGTAATAGTTTCGGAGATACAGCCTTGAGAACTTGTGCGCTCGAGGCTAGCTAGGCTAAGTGCGCCGTCTTTAAACGCGTTTTTCTCGAAACTGTGTTTTTGAAGTCGGTTGGCAAGATTTCTCGAGAACTACTCAACCGATCTTCATGAAATTTTACACAGGTCTTTGAGATACAATTCTTAAAGACTTGGACGAAGGATTTTTTTTCGATTACAACTATTTGAAAAAAAATGTCGCGAAATTTTCACTGAAATTTTAATTTTTTTGTAAAAATGTCTGCCAAAAATCCAATTTTCAGTTTTTTTCCTTCGTCCAAGTTCTAAGTTAAGGTTTTAACTAAAACACGTATTTTTTCACTTTAGATGATCCTGTAAGGAGTTATCCTGCCAACGCGGGCGCATCTTTTTTCCGAGGGGTCACCGGAAATGGCGTCGCAATGGCCGAGTTTAAAATATTTTTTTCCAAAAATTTCAGAATTTCTTTGTTAATAGTGTATGTTTGTAACAATAAAAAATTCTAATAAAATATTTAATTTTTTATATGAGAAAAAAATTGTTGAAAAAGGCTGTTTTTTACCCGAGGAAACCCATGTAACCCCTTAA

>Btry_159#Unknown

AATATATGTGACCTGGTCTACGGAAAGGGGGCTTAGGTGTCAAAAAAAAGGAGAACAATTAATGAGATAACGAGAAACTGACGATTATTTTTAACAGCTTTTCCCAGAAAACTAGTTTTCGCACGTTAGCTCCCTTTTCGTAGACCAGGTCACATATAT

>Btry_602#unknown

ATAGGGGATGTTCGAGCAAGCAAATAACTGCGGCAATATTGCAACCCTGATGTTTTTTTGCAGATACTCAGCTGATACTTAAACAAATATTTTGCAAATATTTTGCGCGGCGTGCAGGTGCATTGAAGAAGTGAAGTTTAAGAAGTCTTAAAAATGAATTTTTAATCAATTTAACAGTGGATAACTTGGTGGATAGTGAGTGTAGTTTGCTTCTTAAATACAAAAAAGCAAGGAAACAAAGTTTTTTGCGTAAAAAAGTGGCTTATGACCGAAATTTTAAAGAAGAGGGCAATAGTAAAATCGCAAAACTTCTTTAAAACAATTCGATTTTATCCTCCAGATATATATTTATCACATTTTCGGATGTCTCGTGAAACACAATTAATGATAATAATTAGTTATAAACAAAAATAAAGAGCGATTTCGCTTGCCTCATTTTTTCCATCATAAATTTTGTTGATGCCGATTTTGTCACTTTTGCGCGCAAGAAATGTCAAAATTTGAGTTGCTCGAACGTTTGACATTTGCAAAAGTTGCTACATTTTTGCGTCGAATATGATGCTTTGCAATATTGCCGCAGTGATTGAGACTCGAACATCCCCATA

>Btry_1006#unknown

TTACAGCCCTATTCTCATGCCCTCACAAAAGTCACCAACCTCACTACAGTGATGTTTCTCACTATAGTGAGGGTTACCTTATTCTGGCGAAAATTCAAAAGCTCAAATGCTCACTATTATCACAAATATCTGTGACGTGTGAAAGCAGCCATCATAATACAAAACATCTGTGTTTGTTTTGTTTTACATCAATATTTGACATTTTTTAAAAAATATTTGAGTGTTATAACTAGCATGGAGGAACTGTTCTTTGTGTATGCGGCAATTGTTGAGGAGGAGGAATCGGGGGGTAGGAGGAAAATTTTGAAGGGTTTACGAGACAAAAGCGATCCTTTTACTATGCAAGACACAACATTTATAAATACATTTCGATTCCCAAAATCGCTATGTAAAGTACTTATAGGTGAATTGGTGCCGCATGATGTGCAGAAGTCGAGTATATCTTTTGATCTGCGTTTCCTGGCGTGTTTGTATTTTTACGGGCATGGCTCTTACCAAAAATGCGTTGGTAATAGTTATATGCTCTCTATGAGCCAGTCCTCTGTGTCTAGAGCTCTGCACTTTATTTCGAAATTAATTATGGATGTTAAGGGCAGTGAAATTTATTTCCCTTCAAGCGCGCAGGATGTTTCAGATACAAAGAAGGGGTAAGATAAATAAATAAAAATAAATATAAATAAATAAATAAATTTTCTCTTAAAGATTTTACACGAAATTTGGGATCAAGGGCACAATCGGCGCAATCGATTGTACCCATATTGGGATTGTTTCACCCTCAAGCACAGACGCATACATATCTTTATTATCCATTTGTGTTTTTGTTTGTTTATTTTTATTAAAACAGCTGTTTGACAGCAAAAAGCTTTTTACCTCACGTGGTGACTTTTTTAAGCTTTTTTCTTATGAGGTTTGAGCAAGAATAGCCTCACAAAATATGCCTCACAAGAAATCTCTCAAATTTTTCCTCTCAACTCAGTTGAGAGGTTTTTTCAGAATAGGGCTGTTA

>Btry_681_sTIR#unknown

TTTCTAAGTTTGCGATTACATGAAGCAACTTTAGTTGCTAATTCTCGGGCGATTCTCTTTTGCTGTCGCCCATTACCTTCACACGAGCAACTTGTGTTGCGCAACTCTGCTCGAGTTTTTTTCTCATTCTCTTTCACTTTACTTTAACCCATTGTCTACTCTTTACTTTAACCCATTGTCGTTTCTTTTTCCTTATAGGTATTTGGGCCACTCTATCACATATATTAATCAGCTCTGTCAATTAAGAAATACATTTTATTTATTAAAACAAAGATATATCACCCTTTTTACTATCGTCTGAATCAATTTGTATGGCTTCCTCCATACATTTCACAATATCTTTAATTAATTCGATTTTTTCGTCATACTCCATTTTCTAAAATATTTATATTATATTATGTATATTTGTATACATATTTGCAAATTACTTACCAAAAGATGAAATTAAATTTTTTAAATATATTCAAAATATTAATTTCAAAAAAATATACGCAAATGCAACTATGTACTACGAAAGAAAGAACACGAATGCCGAAAAACGTCGCAGCGAACTGTTACGAATAAGAACACAAAGCGAGTTGCTGAACACTGGAAACTCGGCGCGATTCCCCTCTCCTCGTCGCCCCCTCGCCTATAAACCAAGAGTTGCCGCAACAAAAGTTGCCTCGTGTAATAGCGCTC

>Btry_759#unknown

AATTTACAGCCCTATTCTGAGAAAACCTCACAACTGATTTGTGAGGTTTATTTTGTGAGGTTATTCTTGCTCAAACCTCACAAGAAAAAAGCACAAAACACTCACCACATGAGGTGAAAAGCACTTTGCTGTGAACCAGCTGTTTTACAAAAACAAACAAGACAAAATAAAGAAATGGACAACCAAGAAAAGTGAGTAATTTTGCAAATCTATTAAATTTGGTGCATATATAATAATTTCATTTATTTTTAGGATTTCAAAATGTGAAAGAACAACTCAGGAGCAGCTGCAGCATTACAGCATATTATTTAAACATAAATATCTTTTTATTCGCACATGTATTAAATACATACATACATATTTACAAATAAAATCTTGTCCGAATTATTTAAACATAAATATGTATATGTCTTTTTATTCATACTTAGAAGGAAGTAAAATGTACAAAGACCGTAAGTTACATATTTATTGTAATAGACAAATACTTATATTTATAATAGATAACGCCTTTGGAAGAATAGTTCCTCCATTCTATTTCAATAAAATAATTGCAAATTTCTTTAAAAATATTGAAATTAACAAAACAAACACAACCGATTTTGCATTGTGAGGTTTGCTTTCAGACATCACAAATTTGTGAGGTAATTGTGAGCATTTGTGCTTTTGAAATCACCTCAGCATACGGAAAACCTCACAANTAACAAAAACCTCACAATAGTGAGGGTGGTGATTTTTGTGAGGGCATGAGAATAGGGCTGT

>Btry32060#Unknown

TTATTATATCTGTCAGCGATTTCCATTTATTTTTTATGATACATTGGTTTGTATTTTAGGTGACGATATATATATTATTATATTTAAAATATGAGTTTTGGATATAAAGTGGTTATATTTTACGGTTATCATGCACTCATATTGAAACAAAATTTGAGTTTTGGATATAAAGTAGTTATATTTTTTGGTTATTATATC

>Btry32545#Unknown

CTATTTATACAAACATATATATACATTAGAGTGATTCAAAAAAAAAATTTTTTTTTCGTTTGGTACTCGGAAAAATAGGTTCCTAGACACCTCTAAGAAAGCCTCTCCAAACATGAGTTTTTAATTGTAACGGGAAGGTCCTCCTACATACAGTTTTCTATTTTTTCTTATTATCAGATAGAAAAATTTATATCTCGCTTCCAACTACTTGAAAAAATATCTTGTTCCTTAGATTTTGTAGGAAATTGAATGCTCTACAAAAAAGGTCTATTATGATTTTTTCGTAAACCCAACCGTTTAAAAGATATTAACAGTTAAAGTTTGATTATTTTTGGGAAAATTTTTTATTTCTTATGAATTTTATAACTCAAT

>Btry89050aTIR#Unknown

ATATCGTCACCTAAAATGCAAAATAACGTAACATCACTTTTCATAAGTTTACAGGCGAAGGAAAAACGATATAATAGAAACCACTCATATTGAAACAAAATTTGAGTTTTGGTTATAAAATGGTTATATATTGGTTATATCTGACAGCGGAAGCTGAAAATTTTATGCTACATATATGTAATATGATGTAGTGAATCGTAAGCAATAAAAAACTCAATTTCGATTTTTTTGATGATACGTTGTTTTGCATTTTAGGTGACGATAT

>Btry89050bTIR#Unknown

ATATCGTCACCTGAAATGCAAAATAACGTAACAACATTTTCGGAAATTTACAGTGGCATGAATGAAACACGAAATAAAATGGAACATGAGTGAAAAAAAATTTTAATATTGGTTAAAACTGGTTTATATCTGAGCGCAGAACCTGAAAATGTTGAACATATGTAATATTATGTATGTAATTTGAAGTAGTGAAGCGTAATCAATAAGACACTCAATTTCGAATTTTTTGATGATACGTTATTTTGCATTTCAGGTGACGATAT

>Btry_725#unknown

TTAAGGGGTTAGGGGTAGTCAGAATTTTCAAAAAATTATTTTTTTTTGCATTTTCGTTAAGTGTAATATCTTAAAAATATTCTCTGAAAATTTCACGTTGATCCGATAATTAGTTTTTGAGTTATTCGACAAATAACAAAGAGAGCTCGGGCACTTCAAAGCGCTAGTGTGAAACTTTAAACGCGTTTTTCTCAAAACTATGTTTTTTGAACTGGTGACAACTGTAACTCGAAAACCGCTCAGTAGATTTTAATGAAATTTATACAGCTTTTAGAATACATAATAAACTCGGGCCTGATCGAAGGATTTTTATTTTTTCGAAAATTTCGATTTTTTAAGACCAATTAACTGTTGATTTTTTCGCAAAAATTTAGAAAAAAATTTCCTGAGGCCGCCATTTTGTTAATTTTTGAAAAAAAAAAAATCCTTCGATCAGGCCCGGGATTATCTATTTATAAAACTAATTTTTTTTGTCCGATTGATTTTAGATGAATCTCCAAGGACTTATGGTTGTCACCGCAAGTACCTTTTTTTGGAACAGGGTCAACACAAACAACTATAACTTTGGAAATTAATTTTTTTTTTTGAAATTTTCGTGACTTCAAGTCAACACATTCTATAATAATGCCATATTACTACTTTTGTAAAATAACACGATTTAATAGCAAAAAAAAATACTGAAAATTCTCATTTTTTCGTGCCTCTGACTACCCCTAACCCCTTAA

>Btry91612#Unknown

AATAATATTATTATATTATTTTTTATATAATATTTTTTATATTATATAAAAT

>Btry_971#unknown

TTAACCCTGGTCGAGACCAACGGTCTGATACACTTAGTCGAGACCAATGGTGCTTGGCTAGGCCAAGTGGTGTTTACATACATTTGTATGGAAATACGTGTTGTATTTACGCTCATATATGTACAATATAGATACATTTTTGAAATTTCTTACCTCTCGATTTGGTTGAAATCAAATTGACTGCCTTGTTCACGCTTGGCGTGGCAATAGCGTTCACAAAAAATGTAACATTTTGAGAAAAATTACATTTTTTCTTAACCTTTTAAAACGCTCTAGCGGCTAAAGTATTTGACCTAGGTGGGTCAAATAAATTGCGCCGGTTAGGTAATTTCAAGAGCTTTCAGGAAATGTATAATTCGCCCCCAAGGCCCCACCGGGGGCGTGGCAATAGGCGAAAATGGGTTTTCACCATGTGCCCCCCTTTTGTTTCACTACAGTTTGGGGGGTATGGGGCCGGGGGGGATCAAAACCATTTTGCGAGTTAGTTCACCCCTGGGGCTTTCAGGAAAGCCCTCAATCTCCCCTCTATCCCTACCAGGGGCGTGGCGATAACGTTTACAAAAAATGTAACAATTTGAGAAAAATTACATTTTTCTTAACCTTTAAAACGCTCTAGCGGCTAAAGTATTTGACCTAGATAAACATACAAACCAATTTGGACATGTAACGAACATGAAAACAGATAAATCAACCGTGCGAAGTGATAGAAACATACCAAAGTTCATATATTTTCATACAAAATATATGGGGTCTAGCCAAGCACCATTGGTGCACTAAGGTGTAATATTATAATATCCTTTTTTCGTCCTTTTCGATACGATATCCTTGAATTTTTTTTCTGAATTCTATAGACAATAAAATTCTAGGAAGCTACCTGGAAGCGCAAGTCGTTTGCTACACTCTAAATATATTTAAATAACATTTAAAAACAAAATATGCCTGGCCAGACCCGAGGGTCTCGATGAGGGTTAAA

>Btry_945#unknown

ACTAGTCTAAACCCGCGGCTCCGCTCGCTCTTTGTTGTCAAGGATATCATTTATCGTGATAACAATATAATGGAATAGTTAAATTGGCAATCTACATTTTTTATTGACTTCGTAAATCACTTAAGGAATAGTACATACATAAATATGTATGATGTAACAACATCTTCACAGTCTCTGCATCTGCATCGTATATCGTTACACATATACGTACATATATGCTTCTTGTTATAGTGAATAACAGTGCAGGAGTAGAGCAGAAGAGGGCGCGTCTCACGCCCTATATCTAATCATATGGTAATGTTTTGTTCAACATTCATCGAATGTAATTGCAAAATCGACGGCTAAAACCCAGATACTACACGTAAGAAAACACAGACTTGATCGTTCCTTTTAAATATTGCATTGTTCTGTGCAATGCTTCAGGAATATTTGTCAGCTATTATGCACTCATCTCAAATTATGATTCAACCTTTTCTTATTCAGTTACAACTATCACTATAATTCATATATANATATGTGTATGTAAACAAAAACCACTGTACCCTGCCAGTAGCTGTGTGATTTTATTTTGTATTTTTGAAGTAAAAAAATGTTCAAATGATTCTTACATGAATATTGAACACATGCTTATGTTTTGAAATATAATTTTTTTTATTTATGAATTGTTGTGACTTTTAACATAAAACGATAGAAAGTAGCCTATGCCCGTCCCGAGGTTCCAAGCTACCTCATCACCAATTTTCAGCCAAATCGGTTCAGCCGTTCTTGAGTTATAAATATAGACATAAATTGCGAAATCGAGGGTGATTTTGCAAAAAAGGCCGCCGTTAGGACCTTCTCCAGGACTAAAGGAATACCCGTGCAAATTTTCACGTCTTTCCGTGCTGTGGTTCGGGCGTAATGCGCGGACATACAAAAAAGGCGTTTCATTTTTATATATAAGAT

>Btry_1338#Unknown

CCCTGCCAACCATGAGCGGGTAAAAAACCAGATAATCAGTGGGTAAGACATAGGGTAATCATGTGTACTTTGCCATAGGCCGAGCGACAATTTTACCCACTCACGTACGTATACATGTGATCATACATCTTTGTTGCGCTCATTCAGCAGTGTCATATAAAAAAGTATATCTGTCCTGCTCTAATATCCCCAATCGACGGCTGATGCAGGGTTGCATTTTCTCTTTTTAAATAGCTGATGCAGGGTTGCATTTTTTGATTCCTACTTTTAAAATTTCTTAAAATTTCAAACAAAACTTTTTAATTTTTAAAATTTTTATTTTATTTTATTTTTTACATATTTACATAAAATTTATTATAATTTATATAAATAAAAATATATTTAATTTAAAAAAATAATTAATATCTGAAAAAAGATTAATTAAAAAAATAAAAGAGTGGAAAAATATTATAAAAGACAAATACCTGAAATAAAATAAAGATTATTAATACCTGAAAATAAATAATAATTACATTGAATTTGTAATATCTAAAAGAAAAAGAAAGATTAATTGTATAAAATGATAATAATATCTGAAAAAAAGATTAATATTATCTGAAAGAATAAAATATATTAAATACAAATAGAATTTACTCTGAAAGCAAAGATTAATTAAATAAAAATAGTGATAAAATCTGAAAAGAAAGAATAATTAAATAAAAATCATAATAATATCTGAAATAAAAGAATAATATTATCTGATTATTTAAAATATAAAGCAAACATTTGTTTTCACATATTATATTGGATTGTGCAGGCCGCCTTAAACGCATAAAATACATACTTATGTACATATGTATGTAATTATTTTTGCGTATTATATTGGACTGCGCAGTCCAATTTCAAAGTACACACACTTTTTCTCACATACTTACTTAACAATTGATATTTGCTGCTTCTGATGATATTGCTGCTGCTGCTATTTCCAATTCAGTTCTGATTTCCAATGCTATAAAAACAAATGAAGTAATTATTTGCAAATTATTGTTAAAAAATCACATAAAATTCATTTACTTACCTTCTTGTTGTACGAGCCTCCTTTACGATGGTACGATCACGAATGACATGCGTCTTTCAATCGTTTTTTTATTACCCTTTTTGGGATTTTCTTTTGTCACTATTGACAATTATGTTTTCTCCTTCGCACTCATTCAAATAAATCAAGAAATGAAAGAAACTTTTTTTCATCGCATTTAGGTAAACAAAAAATAACCCGAAAATGTGCGTTGGTGTTAGTGTATAGAGAAAAAATGTGTAGTTGTATTGGAATATTTGTCTCAAGCGGGTAGCCGTGGTTGGCAGGG

>Btry_259#Unknown

CTGGGGCCCTATTCTGTATCTCGATTCGAAAATGTATTCTATTCGAAATTCGGATCTACTTTATCATTATGCGAAAGTTGTACCACCCTGTGCCAGAATAAATACGTACAATTTTCGTTTTTGCTTTCTACAACTCAAAAGCTAACGAATATTTTATTCGAAAATTGGCTTGAAAATCCGAAAATCGAGTTACAGAATATAAAAAATCCGAATTCGAGTAAATTGTTTTTCGAATCGAGTTACAGAATAGGCCCCCTG

>Btry_425#Unknown

GCGCGAGTTAATTTTTACATTAAGTAAAAATGAGTCAAAAGAATATATTTTGAGCTGCACCGAAATGTGTACTCTTTATTTATACTCTTAAGACTAACTTATTACATGGTTCTTACTTCTATTTATACTAACTAGTATGTTTACTTATTACTAGTTGATAGTTTGTTAAGATACAGATTATATTATTTGTTTAGGTTTGTTTACATATATATTGTTTGCTTAGATACATTTGCTTTGTTTTAAGATAAGGTTGTTGTGGTATTCAAACTCAATGTTGTTTTGATACTTGTTTGTTTAGGATTGTTTGTTGTAGTGATAGTGCATTTCAATTGTTCCAACTCAAGGTTGTTTCTGCATTCTGTTTACTGAAACAAAAGGTTGTTTTGATGTTTGTTACTATTATAAGGAATACATTCCTTAACTCG

>Btry_639#Unknown

TCAGGGGTGTTGTGGAATCTGATAGTTGTCGGAATCAGAATTGAAACCGATCAAAAAAAGTAGTAGGTGTCATGAATTCAGATATTATTGGTTTGATATTCGAAACAGAATTTGTATCGGTTTTGGGTGTCACGGAAACCAATTTTATCGGTTTTGCTATCAGAAACAAAGCAAGAAGATAGTCGCTCACAGATTTGAAATGTAAGTTAAGAAATCAAGTTATTTTATTATTTCGAATTAATTTATCTTTATTTCTATAGGGGAAAACATAAGTATAAAACACGAAATGTCTTAATTATTGAGTTTTTGGAAAAAAAATCCGGATATTTAAAGATATTAGATTTACAAAACGTAATAAACGTAATTTAACACCCAAATGCGTCTTTATTCATTCGATAAATGTTATCTCTTAAAATCTTTCTTTAATTCGGAACTCATTAAAAACTTTAATAGGATTGCTTCCAAATGTATTCATTTGCAAGTTTTGTCACATTAGTAAACAGCTGATTCGAATATTGGTTTTGCGTATGTTCGAAAAGACGAAAATCCAATCAGATTCTGTGACACCATTGAAAATCAGAATTGGTTTGCAATTCTGACAGCTAAGCAATTCTGTCTTGGATTCCACAACACCCCTGA

>Btry_526#Unknown

TTAAGCTTAGTACGCAGCTCTCAAGAAACGTAAAAACTTCATATAAAAAAACGCTTAAGAAACGGTCAAGAAACTTTCCTGCGATAAATTTACTTGTGCTAGTACGCAGCTCTCAAGAAATCTAGTACTAATTTTTGATACTTTTTTTCAGTAAAATGTGAATATGGCAAACCTGGTTTTGCGAAGAAACATCAAATCAACAATTGTTTCTTGAAGGAAATTCGAAGTAATCGTCAAATTCATCCTAAATTAGTTGAAATCATTATTATGAAGTATATTCCATTTTGAAATGTTGTATAAAACCTACCAATCATCAACAACATTTCTTAAATCTCAATGAAAATATTTATGTTACCATACACATACACACATACATATTACGCACAAAGTTTGTTTTCTTTGTTTATTCTCTCTTGCTCTTCTAATGTAGATCATTCACAATGCGTAACCAGCTGTCAAAATTACTTGAGAAATAATGTATTTTTTTTCTTGAGCAATTACTTGAGAGCTGGATACGAACCTTTA

>Btry_260#Unknown

AGGGGTATTCTCTTGCTCTTGCAAAACCCTTGCACGGTGCACGAATTGCAGATTTTTCCTCTTGGTGCAACGGCACAACAACAAACACACGCAGAAAATTATTTGCAATGGCTGTAAAATATGTCAGACCAAAACCCATGTTTATTTTCGTGCAATGTCACGCAAAAATATAATTGCAACCCTGTTTTAGCTGTCATTTTACATAAAGACATATTACGTCGTTAAATTGTTGAGGAAGGTAAGTTTTAAATAGATTTTAT

>Btry_562#Unknown

ATAGCGGGATTCTGTAACCAGTCTCTAGTCTCTATTTGAGACATTTTGAGGTAAAGTCTCAATTTGTGACTAGTTACTATTCACTAAACAGTCTCTAGCGTTAGTCTCAAAATATTGCCTCAAATTTGAGACCTGGTAGTTAGCAGGTCACAAAACTAAAAAGAACTCTTGTCTGCCATTTTGAATATACTGAATAGAGATCATCATAGATATTAATCGATTGTCGTTTTTTTATATTTTGTAAGCAACTCAAACACGAAAAGGTTAAAAATAAATCAATTTTACATAAATTTCGTAAATATTATGAAACAAAGTCAACATATATTTTTATTTTTATTGATAATTATGTTTTTTGACTATGTTATAGAAAATTTAATATTTGTTATCGACATATTTATTTTCATTCAAGTGTAAAAACATCTCTGAATAATGAAATGTAATAGATTTTGTGACTGTCACTTACAGAATAGCAAAATCGTCTCAAGTCTCAAAAATTGTCTCTAACTTAAAATCTCAAATTTAGAGACTTGAGACTGTATCACAGTACAGAATCGCACGGTAA

>Btry_724#unknown

TTAAGGGGGTATTCTGGTCTAGACGCATGAATTTTAGGCATTTTTTAAACTAAGATAAAAAAAAATGAAAAACATTTTTACTATCCATTTTTTTATATGTTTTTTATTGACATTTTAATAATATAAAAAAAATTGCAAGAAAAAAAAACAAAATTCGTCGAGATACGGGCCGGTGGAGTGGGGGCTTCAAAAAAAACGGTGCGTCCTGGTTGACGTGATTTCAACCCTTGTAGAGATCTAAAACACAAAAAACAAGAAGATTCTTCATTAGTAAGGATGTCGTTATCGGGTTGACCATTTTCAAAAAAAAAAAAAAATAACAAAATGGCGTCGACTTGAAAAAAAAAAATTTTTTTTGACCCGATTTTTTCGACCTTTTCGAATTTTTTAAAAATACTTCAAATCAAAATTTTTGGAAATCCAAGGCAGACACGATAGAGCATTGTATAAAGAAGATATATACCAAATTTCAAAGGAATCGGTTCAGTAGAACTTGAGATATCATGTCAACCACCTCAAAAAAAGTAGTTTCGAGAAAAACGCGTTTAAAGTTTAGGAGACAATTCTAGTGAACACGGACGCCACGTCACAAAATCGGCTGTATCTCCGAAAATAAGTCGAATTTTGAAAAATCCTTCCAAGGTCATATTTTTGAAAGTCTAAACTTTCAAAATATGCAAAAAAAATCGATTTTTTCAAAATCCTAGACCAGAATACCCCCTTAA

>Btry_1998#unknown

CAGGGGTGGGCATATTAGCCCTCAGTGGCATTTTGCCCGTTCGGGCACGAGTGCACATTTTGAGGGCTAGATGAGGGGATCATCGCGGGCAAACATGAAGAGGGAAGTGAGAGCAACGTATTTTACCACTCCATATTTACAAATGAGAGCAACGTATTTTGCCACACCATGTTTACAAATGAGAGCGCGCGCATATATGGGAAGTTGCACTGTGGGTAATAACTGTAAAATTGCCTAACAATTTTAAGTTTCTTTGTAACTACTATAAATTTATAAAATGTAAGACAAATAACAAAAAGTATAATAAATATTTTATTTTAGTAATAATTTTTTTAATATGTCTTTATAACAGCATTTAATTTAGTTTATTTCAAATAATAAATTCTATGTTTGGTTTAATCTCATTTGCTGTAGCTACACGCATTATTGTACCAAAATTTAAATCAGATAACCTACTTCTTATTTTATGTTTGTTTAATTTCATTATTGAAAAAAATTATTCACAAGTGTAGGTACTTCCAAACATAGATATAATTTTCGAATCAAAAAGTTTCAAACCAGGTTATTTCTTTAAACTTCCTAATAAAAAGTAATTTTAATAATAACTTCAGTTTATGAACCACTGTTTCTTCAAACACATATGCCCAAATGAATCTTATGAGAGCGCAATGTAAATGATCAGCCAACACCGCTTCTACCGCCCGCTTTACCGTAGGATACCGTAGATTTCCACGGCATTTGGATTTTTGCCGTAGAAACGTGTCAGCTGATTGCTCTCTCACAACGCAGGGTTGCCAATCTCGATATACTGTAGTAATTATAAAAATTGTCTTCAATATGTTTGAAATTTTCTTAAACTAACTCAAAATCAGAGTCGAATGCTATTATTTATAAATATATAAACTATTTTTAATAGTTTGATTTCAGTTTTGATATTTTATATTATGAAAAATTGTAATTGAAAAGTTAGATGTGTACAAAACTATATATGCGTATTGAGCAATGGCAACATTGTTCAAATGAAAACGAGAACAAAACGCTTGCCGCTCGTAGCGAAGGGAGAAGCGNAATTCGATGTTTCTGCCATTAGCGTTTGTATTCTATTTGTGTGCTTAGCCACTCTCAATCAATAATGCCATACCGCGCAAATATATTTTTATTTGATGATTTCTTATATTTTTGAAAATATGTGTTCCCTTTCTATAGCGCGTATTATTATTTTAATACATTTCCAGAGGCAACTTAGATTTTTAAAATTTAACAGAACAATTATAGAAAAAAACCTCAATTCTTTGAATATTTTGATAAAACAACCAAACAAAACAACCAAACTGAAAATATCACAAATTATATATTTATATAAGCATTTTCATTAAATGGAACTGCAATATGTTTAAACAAAATATATATTATGGTCACAAGTATCTTTTCTTTTTTATATATGCAGATATATAAAACTGCCTTGCATATGTACGTATAAAAGCCCACAACTTGAGAAAAGTTTTCCCAATTGTAGCTAAAATGAATTTTTACAACTGGCATCACCACCATTAACTGTTCTATCACATGTACAGTGGTGTGAACAAAATAGGAACAACCATAAGGTGTTGTGAAGTTTTAAAATGTGCTGTTTTCTTATTAAATAAAATTGTTTTTAGATACAAGTATAACTCATATAATTTTTTCCTAACAGTGATTAGAATTTCCCACCATGCAAAGGTAAATAAAAACAAATATTAATGCGAAACTCTAAAACCTGCAAATTATGTTTACCTCTTTTTGTTCACACAACTGTACAATTCCGATATGAAGTAAACTTCCGCTCTTTAATTTGTGTACAATGTCAGTATTGCCGCAACCATTCTGCGATGAACATTAAGTTACGGAGGAATGTAATTGATTTTTTTCGCAACTTTTTATGCCCTTCCCTCCGTAAACTGATATTCCCCTCATCTAGCCCTCGTGCGCACTTTGCTAACTTTGCGGGCTAAAAATGTGCCCACCCTG

>Btry_727#unknown

TTAAGGGGCTATACCAGTGTGACGCATGAAAAATTAGGCGATTTTCGTGAATTTTTTTAAAAGAAAGTACTAGATTGAATGTTTCGACGTTCTATGGACGTAATAAAGTATATTTTTAGCTATATTTTAAGATTTTTTTTTACAAAAATATTGAAAAATAACGGAGTTATGTGCTGTCTGCGGAAGTGCCAAAAAAAAGTGCCTCAACTGCTGGCATGATTCCGGTCGAATGAGTAGCTGAAACAAAAAAATTCAAAATGTTTATTAAACTTAAATATATTTTCTATACAATGAACTACGATCTTTGAAAAATATCAAAAATTAAGAAAATGGCGTAATTTTGAAAAAAAAATCGTTTTTTTAGGTAAAAAATGGTCGTTTTTTTAATGCCAAATTGACAATTTTCAACCAATCAAAAAGATCGTAGTCTATTGTATAGCAAATGTATTCAACTATACCCAGTTTTAATTTGAAGTCGATCGGTCAATTTCTCGTTGAGTTATGATGTCAGCAATTTTGAAAAATGTCGTTTCGAGAAAAACGCGTTTAAAGTTTCACTTATATATGTTTGCACCTCTGAGCGGTCGCTCTTTAGAACGCTGCCATTCAAAAACTATTCAAGATACGACCTTGCCGATTTCACAGGATATTTTTGAATATATAAGCTATCGAAAAAGCAAATAAAAATTTTTTTTTTGAAAGTGTCACACTGGTATAGCCCCTTAAA

>Btry_2442sTIR#unknown

AGTTCAACCAGAGAACTTAAAACATAGAGAAGGTGCAAATTGAATTCTGTATGATGTTCGATTGGACGGCTAACAGAGCTTATAAGATTGCGACACGGAATTCACCATTCTCGCTGCTATTTAGCAGAATTGAGCACGTGCAATGGCAGAAATATATGTAAAACGACTGAATTCATGCGAGAATGAATAAAGAGAAATGCTTTGAAGAAAAATATGCAAAGTCACACAAAGAATGTAAAGAAGCATTCTCTGAGACACATATGCGTGATTATTTTGTATCATATGAACCATTTTTTTCCGAAAAATGGTAAAAATTTTAATTTTTTTACAAAATCGGGAAAAATTAGGTAAAAATAATTTTAATTTTATTTTTCTTTAATTTTATTTTTTAATTTTTAAATAAATTAATATTTCAAAAAAATTATATTTATTTCGTCTATTACAAAGCGAAGATGTGTCAAATGAACTTCATTTCTTCAAAGAAAATTGATGACCTTCATGAAATATGCATATTCGCATTATTTCGTTATCATTTCCATATTTGTACCAATAGAATTTCTATGGCATATACATATATACATATATTATTTCTTTGGCACATTTGTCTGTATGTTTACGAAAGCAAATGCGAGCCACATACATACATATGTACATACATTCATAATAACAAGTGTCGCACGCTTCTTTCGCATCTCCGTTGTCACGGTCAACCAATGGCCGAAACTTGCGTTTTGTCAAAGAGTCAAGTGCTGAACAAATTGAGCACGACAGACTGCAAGCGACATCTGTCAAATATTAAAATACACACGTTCTTATGGACACAGTTATGTAGTTGTGCAGCTGGCTCAATAACTGTGTCCCTAAGAACGTGTGTATTCTAATATATTCGTTTGCAGTCGGTCGCGCTCATTGTGTTCAGTACTTCTATGTGTCGAAACACTGACGCGACGGTAAAGCGCCACAACATTGTGTTGTTGGTAGAAAATCCGAGCTGTGCCGAAAAAAATAAACGAATGGCCGCCGATTGTCACCGCTTCCCTTGCCGCTGTAACAGCTTCGCCTACAAACCAGCGTAAATATGTATGTNTATGTATGAGCGTGCATACATATCGACGCAGATTTTTGCGAGTCACGGAAAAATATTTTAGACAAATATTGTAAATCGACAACGGCTATGTTGCCACTTTTGTCACTTCTTTCTGTGAACAAACATCAGAAAGTTGTTCAATTTATGATTTTTAGCTTTTGCCATCGTCGCGAAAAGACGCTTGTTGGCAAAGGCATGCTCGGGGAGATGTTACGGCGTCTGTTTTTATGAATGAAGCGAGGTCGCTTGTGGAATTTGCGCCTGCGTTTATGAATGAAATCGTTTTTGTTGTAAAATTTACTACATTTGGGCTTCGCCAATTCGGCTGCCATATTGACTTGTGATGCTTCTGCTATTTAGACAATTGTTGTTTTTGTAGAACAATGCTTCAATTTTTTGTTGGTGACATATTCACATGTGTACGCATATGTATGTTTGTATGCTTGTGCATTATTTGTTCGGAAGTGTATACAAATATATGTACATATAAGTATATATGAATGTATGAACATGCAGATCAATGCGCGCACATGTAATATATTGATAAGTGATAAAATCATGATTTGAATTTCTGAATGCGCACATGCGTATACATACATTCATATGAGCAGATAATAAGATTAATATGATAGACGATATATTTATATATTGTTGTGATAAATTCAATTTCTATTACTAAGAAACATAATACAAAAATATTTATTAGTATAGTATATTATGTAAAAATCAAATAAAATTATTAAATATGCAAGTCCAAATTGACTGTAAATATTACTATGCATGCATTCATACAAAATTATACTCATATCATAATTATGTTTACATGCCAATATGGAAATGCCGACGGCAAAACGCAAAAGCATACATATTTGCATACATTGCGATTCCCAAGTTGAAAGCAAAAATTGAAGCATGAAAATATCACAATGCTGTTGTTGGGAGTATCATAAGGAGCATGCATACATATATTTATGTCTCTTCATATTCTTGGGCATGTGAGACAAGAACATAAAAAATTTGTTTATGTTCTCTGTGTGAGAGGTGTGTTTTGTACACATTTTTCGCTGTTAAAAATGGAATATCAAAGAACTTATATTTCTGTCTCTTCATATTCTCTGCTTGGGCATATATGCCATGTCATCATATGCCGTAAATACATACATATATTTATTTCTCTTCATATTCTCTGTTTGAATATGTGGAGAACTGTTAAAAATGTTAACATTTCACAGCGTGAATTCTGTGGTTGTGAGGTGAATTCCTTACTATAAATCTAACAAATGTTCGCTGTCGAACCTGCACCTTCTCTATGTTTTAAGTTCTCTGGTT

>Btry_489sTIR#unknown

TAATAGCCTTGACAGACGGCAGCGTTAATCCGGATTAACTGCGCACTTAATCAGATCCAAATTTGTAGGTGACAGACGGCAGCTATGCGAGTTTGAAACTCTCATAAACAGCTCATTGTCCTTTTTATAATATTTTCAATGAAAATTGATAGAAGATAAACATTACGGTTGTTAGCCGACCAATTTTTACCAAACCATTTTTTATTACAAAAGCATATCAACACATTATTTTTAAAACTGCATCATAACATAGAAGTTTTATTATTGAGAATTTGATAAACAGCTGTCAGGGTTGCTTGTATTTCATTCACAATAGATGAAAATTGGCCATTTTTAACAATGTTGCCAACGAAAATGCAGCTAACTCTCTAAAATTTTGAGGATTAAATGGGAGTTAGCAACGGAGTTATCTTCACTAACTCCTGAATTAATCCCGAAAAATGCAATTAATCTGGTTTAACGCTGCCGTCTGTCAATGCTATA

>Btry_359sTIR#unknown

TACAGTAGAGGCCCGCTAATCCGGATCAATTAAAACCGGCCCCTATCCGGAATATAAGGAAATCCGGATTACCAAAGACATATCAGAACTATGTATTATGAAAAAATATTTATAAATTTCTGTTTGTTTATTATAACAAATAATACACATGTTCCAAAAAAACAAAAAAAACTTAAACCAATAAAGCATAAAAGCGAATGTAGTGAATAATAAACATGTGATCCGGATTAGAGAATACGGATAGAGAATGTCGGTCCGGATTACAAGGGACATAATAGAAATTTTGAGTTTTTTAACCCTGTCCGGATTACAGTGGAATCCGGATTAGGCCATGTCCGGATTAGCGGGCCTCTACTGTA

>Btry_1530#unknown

CAGGGGCGGATCCAGAGTAGAATTTTGGGGGGGGAACTTTATGATTTTGTAATTGATTTAAGAAATGTTTAACTTTAAACTATAATTTTATTTTTTATACATATTTCAACATTTAATTTACAGCATAAAATCAATACGGTGTTTGCTCATTTTACTAAATCTTTCTATTATTTATTCTGGTTCCACAGCAATTTCGTAGTTTATGTTGAGGAGTGCTAAGCCGTTCAATCTTTTCTGGAGCATCGTTGTTCTGAGCCAAGTCTTGATTCGGCGCAAAGAAGAAAAGGATCGTTCAGCGCTAGCATTTGTGGCTGGCAAAGTGCAGAGTACGTGAAGGAGAATATGAATCGACGGATATATATCCTTATCGCAAGTGCTGAGGCTTTCTAAGGCAAAAAGCACTTGTGTTCTCGTAGTCATTTTCATTTTCCATGGTTGTTGAAAAAGCGCTACTTCTGCTTTGAGCTTCGTTTTTTGCAGTTCTTTGTTTCCGGCAAGATTTTCCCAAAACGTCTAATTAGGCATTCAATGAGTACAGTTAAATCCTTTTCTTCCAAATTTAGGTTAAATTTTGGTAACAGCTGACTCAAATTGAAACTTTCAAGAACTTCATCGGAAAACCGCGTTTTTAAATCTTGCATGACCGTGTCAATGAGTGGAATGTATGCAGAAACCCTGTAAAAGTCTTCACAGCTTTGAGATGGGTGATTGTTTCGATGACTCTGACGACCACATGTTCCGGGTTTTTCCACTTCAACATCTAATTTTCAACCATCTGTGTAGCATTTGAAAAAATTTCATTAAAGTGATGATCAGCATNCATTCTTCGCGTGTTCAGTGTTGCAACCAATGTTGTTATGACGTTTGATGCTTTCGCAAGGTCGATTGATTCGTTCTGCAGAATCACACTAAGTGGCTGCGTCAGTGACAAAACATCACTGAGACAGAAAATTCCAATAATAAATTCGAAGTTGCACAGTACTGTGACTAAATTCGACGCTTTTACTGCAGTCGTTCTATTCTTCCATTCGCTTATTTTTGTCAGTACTTGAACGATTTCTGGAAGGGTGACTGTGAATTGATGAACGGCATCATGTCTTTGTACCCACCTTGTCTCACAAAGCTGCACAATTTTTCTGCCCAAGAATTGAGCTAAAACAGTGTTACGTTTCGGTCTTGAAACTTGGAAAAATGTTGTAACTTCTCTGAATATTTCAGATATCTTTCCAATCAAAGCAATTTGTTCAATTTATGGGAAAAACATGGTATCATCTCAGCGTTTGTAGCTTCTTTTTGTATTTCCTTTACAGCTCCGCGATCTCCCAACATAACAATACAACCATCAGTACCTATTGCAACGCATTTCTGAACATCCAGCCCCATATCTTTCATGTCTTTCAAAACCAACTGTCCTAACGCAGCTCCCGTGAGTGAAATCTCCTCGTGGTTGTTTTCTTCAATACTCAATACTTATATATACATATACTTNATATATACATATATATACTTCAACACTTATATACTTCAATACTT

>Btry_196#unknown

AGCGAATGTTGATGTGCGAATTGTATGTAACGAATTGAGTCTGCTTTCCCGTTGTCCATCCTTTTTGTTGAGTGGGTTGGCGTACAGATGTGGTGAGTTGTGTTGTAGTGTCATCAGCTGTGGTGAATTGCGCTGTAGCATTAGGATGCGCCAGTTTTACCGGGTAGAGGGGGTGGATGCTTAACTCATTTAAACA

>Btry_InvRpt#Unknown

TGGGTCAAACCACGTCAAGTGGTCCATGAAAATTTCGAAAAATCACCGATTTTGAAAATTAGCAGTTCATTAGGAAGGGGACCAGACGCATACCCCGTGATATAAATATTTCGTCAAAATTCTTTTTTTTGTTAAAGTTATTGAAGGTTGAAATTTAGAGCACGATAAAATTGTAAAATTATTTTCTCATAAACTACTGGAGATAAATCGATGAAATTTTGTGAAGTCAAAGAAAAATGTTCGTGCTATATTATAAATATTTTTTCACGATCCATTTGTTTAAATAATAATATTTTACATAATTTTTTGTTAAACAATTGAAATTTTTNAAGTGTTCTTCACGCTAAAAAAACTGAAAAGTATGTGACA

>Btry_TIR1#unknown

TTAAGGGGGTATTCTGGTCTAGAAGCATGAATTTCAGGTAATTTTTAAAGTGTCGTAAAAAAAGGCAATTAATATTTTTACTATCCATTTTTTTATTAGTTATTTGTTAATTTTAGAAGAGTACAGAAAAAAATTAAAAACTAAAAAAAGTAAAAAATTTCAAAAATTATGAGCTGACGAAGTGGGGGGTCTCTAAAAATTTCCACGTGACCATACCCATGATTTCAACCCTCCTAGTTATCTGAAACCAAAAAAAAAAGATTATTAATCTAGAATAATGTCGCAATGGCCGGAACTACGGAAAAGTGGGAAAAAATTTTTTTCACAAAATGGCGACTGCCTGAAAAAAAAAGTTTTTTTGGATCACTTTTTCTGACTATTTCGAATTTTTTAAAAATAATAAAAATAAAAATTTGGGGATGGGGCTAGTTCCAACCATAGACAAGCCTATAAAGAAGACTCTATAAAAATTTCAAGTAAATCGGTCCAGTAGAACCTGAGAAATCGTGGGTATCGTTCCGAAAAAGTCAGTTTTGAGAAAAACGCGTTTAAAGTTTCGCGTAAAGTCTTTTGTTCGATTAGTTCCGGCCGAACCAGTTTGGATGCCGGGTCAGAAAAATGCCTATATCTCCGAAAATAATTTGAATTTTGAAAAATCCTCTTGTACACATATTCTTGAATAGTTAAACTTTGAAAATATAAAAAAAAATTCGATTTTTTTAAATTTCTAGACCAGAATACCCCCTTAA

>Btry_TR545#Unknown

GGCGTGGCAATAGGCGAAAATGGGTTTTCACCATGTCCCCCCTTTTGTTTCACTACAATTGGGGGGGTATGGGGCCGGGGGGGATCAAAACCATTTTGCGAGTTAGTTCACCCCTGGGGCTTTCAGGAAAGCCCTCAATCTCCCCTCTATCCCCACCAGGGGCGTGGCGATAACGTTTACAAAAAATGTAACAATTTGAGAAAAATTACATTTTTTTCTTAACCTTTAAAACGCTCTAGCGGCTAAAGTATTTGACCTAGGTGGGTCAAATAAATTGCACCGGTTAGGTAATTTTAAGAGCTTTCAGAAAATGTATGATTCGCCCCTAAGGCCCCACCGGGGGCGTGGCAATAAGCGAAAAAGAGGTTGCGAGAATATTGGTAAATTTATATGTAATTTGCACAAAAACGGGAATCAAATAATAACGGTAACTATGTGATTCATGTAACTGCAGGTGTGTGCGTTACATACATATATAAATCACTGATAAGAATTGAACAGGTGAGTAGTTATCACAAAAACAAAGATAAGTACAGTTTTCAAAATA

>Btry_pallindrome1#Unknown

ATATATATATATATATATATATTAGGGTGTTTTTTTTTTGAACTATTAATTTTTTTCAGTCCCGTCACGAAATTTCCTTGGAAATACCCTAAAAAAATTCCCTGAAAATTTTAGCCCTTAATATTAACATTAAGAACTGGACCAAGGCCTGTAAAAATTTTCCATAGAAAATACACTACAATCGTGAGTTTTTATCTTTAAATTCCTACAGATCAGAGGTATTTTATGGCATCGCTTTGACTTTAGACGCATATTTCTCAGAATTGTTCACTCTACAAAAGTGCCCAGTGCAACAAAGTCGTAACTCACACCGGCGAGGTAGTACGGGGCTCTAAACCCGATTTTTCCAAGAATTTCGCATTTTTTTGTATATATCTCGTAAATGACAGGAGCTATAGAAAAAATGTACATGACAAATTTGTAGGAAATTTTATTTGCTATAAAAAAGGTCCGAGGTCAAAATCGCTATCATTAATACTTCTCGAGATATTCGGCTTTTTAAGTAAGGCTGTATGGAATTTTCATAGATTTTTTATTACATACCATCTATGAAAATTCTATAAAGCCTTACTTAAAAAGCCGAATATCTCGAGAAGTATTAATGATAGCGATTTTGACCTCGGACCTTTTTTGTAGCAAATAAAATTTCCTACAAGTTTGTCATGTACATTTTTTCTATAGCTCTTGTCATTTACGAGATATATACAAAAAAATGCGAATTTCNTGGAAAAATCAGGTTTAGAGCCCCGTACTACCTCGCCGGTGTGAGTTTCGACTTTGTCGCAATGGGCACTTTTGTAGAGTGTTCAATTCTGAGAAATATGCGTCTAAAGTCAAAGCGATGCCATAAAATACCTCTGATCTGTAGGAATTTAAAGATAAAAAATCACGATTGTAGTGTATTTTCTATGGAAAATTTTTACATGCCTTGGTCCAGTTCTTAATGTTAATATTAAGGGCTAAAATTTTCAGGGAATTTTTTTTAGGGTATTTCCAAGGAAATTTCGTGACGGGACTGAAAAAAATTAATAGTTCAAAAAAAAACACCCTAATATATATATATATAT

>Btry_pallindrome2#Unknown

TATATATATATATTAGGGTGCTTCAAAAAAAATTTTTGAATTTTTTTTTCAACTCTTACCCCCTCAAATGTTAGTGATTGACAAACAAAAAATCCTCCCTCAAGCCAGGCGCTGTAGCTTAACTCTAAGGGGTCGCTATTTTTTTTTTAGGTTCCCATACATTTAACATAGGAATTTTCGTTTTTTCATTCAAACTAAAATTTCACCAACTAATTTTCGTTTCTCAAAAATAACCATCACATATTCATAAAGTTGGCTTTTCAAACTTTAAAAATTCCCGCGACAAAATTATTATTCTCAGAAACTTTTTTTATTTTTATTGAAAANATTCCAAATTTCAAATCACTATGTTTAATCGTCTAATTGAGATAATCAAACTTTGTCGCAGAAATTTTTAAAGTTTGAAAAGTGAACTTTCTGAATATGTGATGGTTATTTTTGAGAAACGAAAATTAGTTGGTGAAATTTTAGTTTGAATGAAAAAACGAAAATTCCTATGTTAAATGTATGGGAACCTAAAAAAAAAATAGCGACCCCTTAGAGTTAAGCTACAGCGCCTGGCTTGAGGGAGGATTTTTTGTTTGTCAATCACTAACATTTGAGGGGGTAAGAGTTGAAAAAAAAATTCAAAAATTTTTTTTGAAGCACCCTAATATATATATATAT

>Btry_pallindrome3#Unknown

TATATATATATTAGGGTGTGTCATTCTGAGGCAACCTTTTTTTTCAACTGAAAAACAGGCTAAAAACTTTCGAAAATGTGAAAAAGAAAGTCACTCAAAAGATGAGCTCTTAATATTAATATTAAGAGGTGCCTGTTTCAAATTTTCTGTTTTCCATATAAATTACATGGAAAAAAAATCATTTTTTTTGTGGTGGTTATTGTGCGGAGGTTATTATATGTGCACGCGATGGCTGCCAGTAGGGATGGTAAACTCGATTCCGATTCTACTCGAAAATCGAGTTTTCTCGTAAAATAATCGATTTTTCGAATTTGTCAGTAATCGAAATCGATCGTCTTGAAATTTGAAAGAGGCACCTCTTAATATTAATATTAAGAGCTCATCTTTTGAGTGACTTTTTTTTACACATTTCGAAAGTTTTGAGCCTGTTTTTCAGTTGAAAAAAAAGGTTGCCTCAAAATGGCACACCCTAATATATAT

>Btry_dispersedRpt#Unknown

TAATAGCCCTGACAGACGACAGCGCTAATATGCATTAGCGGGCTTAATTTACATTTACTTGCGCATTTGACCCATGTTAATGCAAGTTTGTAATGCACAAGAATTTCGTGCATTTAGCTGAATTTACCAAATTTGAGTAGGCAACACTGCTCAAAAATGGCCGATTTTTGCTATTTTTTGACAGATGTCGCTTGAAATCTGCCGCCTTGTTTGTGTTTACAGCTTCAGAGGTAAACAGTTTTTATATTGCTAATTAAATTATGTGCAAGTATATTAACAAAAACAAATTTTAATATTTTTAGATGGCAGAAAATAAACAACGCACAGTTTGCTATCCATTTTTCCAATATTCTTAACTTTTTCGCACACTTTTATTTCACTTTTTTTTTTAATAAATAAACAAATTTCACTATCAGCTGTCTAAATGCACAAGTCTGCCGTCTGTCACCATAAAATTTCAATGCGCATTAGCGTTGCTTAAGGCGCATTAATTTTGCTCGTCTGTCAGGGCTATAA

>Btry_90#unknown

GCACGCTCATACATATACATACATATTTACGCTAGCTTGTTGGCGACGCTGTTCGAGCAGCAAGGGAAGCGGTAACAATCGGCGATCGTT
