# Supplementary material for: The draft genome of the pest tephritid fruit fly Bactrocera tryoni: resources for the genomic analysis of hybridising species
Source: BMC Genomics. 2014 Dec 20;15(1):1153. doi: 10.1186/1471-2164-15-1153 (PMC4367827; doi:10.1186/1471-2164-15-1153)
Supplement: Supplementary file 3 — Additional file 3: Alignment of rRNA sequences. The alignments of the three Bactrocera rRNA sequences, along with the D. melanogaster rRNA sequence. The 18S, 5.8S, 2S and 28S regions are indicated on the B. tryoni sequence by blue highlighting. The red highlight indicates the B. neohumeralis insertion identified in an earlier study [8]. (DOC 131 KB) [file 12864_2014_6888_MOESM3_ESM.doc]

Additional File 3

Btry GCACAAATTTGCTCAAATTGACTTATACTGAAATAGTACTTATATGAAAATGATTACTTG

Bneo GCACAAATTTGCTCAAATTGACTTATACTGAAATAGTACTTATATGAAAATGATTACTTG

Bjar GCACAAATTTGCTCAAATTGACATATACTGAAATAGTACTTATATGAAAATGATTACTTG

Dmel TCGCA---TTGTTCGAAAT-----------ATATATTTCGTATA----------------

Btry TATGACTTTATAAGTTAGTACTTATATGAAAATTTTTTATTA---GTGAAAACTAGTTAT

Bneo TATGACTTTATAAGTTAGTACTTATATGAAAATTTTTTATTA---GTGAAAACTAGTTAT

Bjar TATGACTTTATAAGTTAGTACTTATATGAAAATTTTTTATTA---GTGAAAACTAGTTAA

Dmel -ATGATTATATTGGT---TACTTATAATAAAGTATATTATTATCCGTACAAATTTGTT--

Btry TATGTCTTATATCTCTATATATTTCACTATATTTTAAGTA----AATATGCTTTTCTACT

Bneo TATGTCTTATATCTCTATATATTTTACTATATTTTAAGTA----AATATGCTTTTCTACT

Bjar TATATGTTATATCTCTATATATTTCATTATATTTTAATTA----AATATGCTTTTCTACT

Dmel ---------------------TCTCAGTTCTTTTTGAACACGGGACTTGGCTCCGCGGAT

Btry TGTATGAA-ATTTGTCATTTCGTACTTGTATGAGAATTTTTATACTTGTATGACTTTATG

Bneo TGTATGAA-ATTTGTCATTTCGTACTTGTATGAGAATTTTTATACTTGTATGACTTTATG

Bjar AGTATGAA-ATTTTTCATTTAGTACTTGTATGAGAATTTTTATACTTGTATGACTTTATG

Dmel AATAGGAATATACGCTATT--------------------------TTAGATAATATCGTT

Btry GACTTAGTACTTATATGAAAATTTTTATACTTGTATGACTTTATAAATTAAGTATTTATA

Bneo GACTTAGTACTTATATGAAAATTTTTATACTTGTATGACTTTATAAATTAAGTATTTATA

Bjar GACTTAGTACTTATATGAAAATTTTTA---------------------------------

Dmel GAAACAAAAGTC------AAGTTTCTA---------------------------------

Btry TGAAATTTTTTTTTTATTATAAGTATTTGAGTAAATAATTTGTATACGTTAAAAATTTTT

Bneo TGAATTTTTTTTTTTATTATAAGTATTTGAGTAAATAATTTGTATACGTTAAAAATTTTT

Bjar -------------TTTTTATAAGTATTTTAGTAAATAATTTGTATACGTAAA-----TTT

Dmel ----------------TTATACATAGAATAACAAATCGTTTCCATATATTA---------

Btry ATAGGGTTTTTTTTATTCCTGGTACCT-GCTACAGTTGGTTTA---------AATAGACA

Bneo ATAGGGTTTTTTTTATTCCTGGTACCT-GCTACAGTTGGTTTA---------AATAGACA

Bjar GTGGG---TTTTTTATTCCTGGTACCTGGCTACAGTTGGTTTA---------TATAGACA

Dmel --------TCGTTAATTTTTGGTGGCAGGCAAATATTAGTTTATTACCTGCCTGTAAAGT

ETS start

Btry AATTTTGTTATATTTATTATAATATGATGAAATTTAAAATTCTATACTTAACAAGTAGCA

Bneo AATTTTGTTATATTTATTATAATATGATGAAATTTAAAATTCTATACTTAACAAGTAGCA

Bjar AATTTTGTTATA-TTAAAACAATATGACGAAATTTAAAATTCTATACTTAACAAGTAGCA

Dmel TGGATTATTATA-TCGTTACGGTATAATACAAAATGGATTCATAT----------TATTA

Btry TATGAAAGAATATTTTAACTTTCTTTAAACTATTCTTATTACACAT-AAAATAT----AT

Bneo TATGAAAGAATATTTTAACTTTCTTTAAACTATTCTTATTACACAT-AAAATAT----AT

Bjar TATGAAAGAATATTTTAACTTTCTTTAAATTATTCTTATTACACATAAAAATAT----AT

Dmel TATGAAAGAA-ATATAAAATTTATATA------------TAAATTTGGAAGAATTATCAT

Btry GTGCTAATGGAATTGTATTAAATTAAGTGAAATATTTATATTATTACGCTCAAA-AAATA

Bneo GTGCTAATGGAATTGTATTAAATTAAGTGAAATATTTATATTATTACGCTCAAA-AAATA

Bjar GTGCTAATGGAATTGTATTAAATTAAGTGAAATATTTATATTATTACGCTCAAAGAAATA

Dmel GTGCGCTCGGTTTTATGTTATATATTACCAGAGAGTTATATGA--------AAAGAGATA

Btry CTTTCATAATCATATATTTATTATTTAAAAATAAATATAATGGAATTTGAAATTTTTAAT

Bneo CTTTCATAATCATATATTTATTATTTAAAAATAAATATAATGGAATTTGAAATTTTTAAT

Bjar CTTTCAT-ATCATATATTTATTATTGAAAAATAAATATAATGGAATTTGAAATTTTTAAT

Dmel -------AATTTTAAATTTATCATC--AAGATGCAAATGATTTAACTT---ATATTTGGT

Btry TTCAAATCAAAGAAAAAATGTATATATTCTTAAAAAAAGATATATACACATTATGCATTG

Bneo TTCAAATCAAAGAAAAAATGTATATATTCTTAAAAAAAGATATATACACATTATGCATTG

Bjar TTCAAATCAAAGAAAAAATGTATATATTCTTAAAAAATGATCTATACACATTATGCATTG

Dmel T-------AAACAAAAATTGTACA-AGTGTGGATACAAAATTTATGTATGTTG------G

Btry AAATAAAGTAAAATGTATAAAAAATGATAT-TATTTGAAAGTTTAGCTAATATAAGAAGA

Bneo AAATAAAGTAAAATGTATAAAAAATGATAT-TATTTGAAAGTTTAGCTAATATAAGAAGA

Bjar AAATAAAGTAAAATGTATAAAAAATGATAT-TATTTGAAAGTTTAGCTAATATAAGAATA

Dmel AAATAAAATGATATTT---TAGAATGAAATATATGTATATATAAAGACAAAATTATAGAA

Btry AACATCGTTCTTAC-ATAATTAAATAATATTATGTATAGAGAACGAAAATTCTTTTCACA

Bneo AACATCGTTCTTAC-ATAATTAAATAATATTATGTATAGAGAACGAAAATTCTTTTCACA

Bjar AATATCGTTCTTAC-ATAATTAAATAATATTATTTATAGAGAACGAAAATTCTTTTCACG

Dmel AATA----TATTACAATAATTGTATGATCTTCTT--------------GTTATATTGGTA

Btry ATACAAAAGCAAAA--ATTAAAAGCAACCGTATTTGCAACATAACACGCATAACAAACCA

Bneo ATACAAAAGCAAAA--ATTAAAAGCAACCGTATTTGCAACATAACACGCATAACAAAACA

Bjar ATACAAAAGCAAAAACATAAAAAGCAACCGTA-TTGCAACATAACACACATAACAAAC--

Dmel AAAC--AAGTAGAA--TTTAAAAATGGA--------------AATACGAATTACGA----

Btry CAAACGTGTCAGTGCCAATTAATTGGACTGA---AAGAGAATATAAGAAAATATATATAT

Bneo CAAACGTGTCAGTGCCAATTAATTGGACTGA---AAGAGAATATAAGAAAATATATATAT

Bjar CAAACGTCAGTGCGCCAATTAATTGGACTGAAAGAAGAGAATATAAGAAAATCTATA---

Dmel -----------GTGCTATATAA------------AAATGGCCGTATTCGAATGGATTTAT

Btry TTTAATATATATGATGATAAAAATTCT----CAAAGAGAAATAAGCGAATAAATGAATAA

Bneo TTTAATATATATGATGATAAAAATTCT----CAAAGAGAAATAAGCGAATAAATGAATAA

Bjar TTTAATATAT---ATAATAAAAATTTT----CAAAGAGAAATAAGCAAATATATGGAAAA

Dmel TTTTATAAAT---ATATTTAAAATTTTTACCCAAAGGCAAAATATTGAAT----------

Btry AATAATGGTATATGGCGGGTACCAGAAT-ATATAAAGCAAT-------------------

Bneo AATAATGGTATACGGCGGGTACCAGAAT-ATATAAAGCAAT-------------------

Bjar AATA---GTATATGG-GGGTACCAGAATAATATAAAGCAATCAATAAATATTAAAGCTTT

Dmel --------TACATTC----------AATAATATAAAAAAAT-------------------

Btry -TGTAAAAAGGTCAGAAAAAATATATAAAGCTTTAAAAGCTT--TAGAAAAAAGTAAAAA

Bneo -TGTAAAAAGGTCAGAAAAAATATATAAAGCTTTAAAAGCTTTATAGAAAAAAGTAAAAA

Bjar ATAAAAAAAAGTCCGAAAAAATATAT---------------------AAAAAAGT--AAA

Dmel --------------GGAATTATATAT------------------------AAAGTGGAAA

Btry TGTGTTGTCTGTGTTGTATATATGTGTGTTGACTTTCTATATTTTGTGTATTGTGTAATT

Bneo TGTGTTGTCTGTGTTGTATATATGTGTGTTGACTTTCTATATTTTGTGTATTGTGTAATT

Bjar TGTGTTGTCTGTGTTGTATATAAGGATGTC--TTTTTTATATTTTGTGTATTGTGTAATT

Dmel ----------------------------------ATCTATAATATTTATATTGCTTA--T

Btry TTCAAATAAGAAAATATCATTTAAAATTTTATATAATATAAAAAATATTATATAAAAAAT

Bneo TTCAAATAAGAAAATATCATTTAAAATTTTATATAATATAAAAAATATTATATAAAAAAT

Bjar TTCAAGTAAGAAAATATCATTTAAAATTTTATATAATATAAAACATATTATATAAAAAAT

Dmel TTCAATTCAAAAAATATGA--------------------------------ATGAAATAT

Btry AATATAATAATATTATTCTGGTTGATCCTGCCAGTAGTTATATGCTTGTCTCAAAGATTA

Bneo AATATAATAATATTATTCTGGTTGATCCTGCCAGTAGTTATATGCTTGTCTCAAAGATTA

Bjar AATATAATAATATTATTCTGGTTGATCCTGCCAGTAGTTATATGCTTGTCTCAAAGATTA

Dmel GAAAAGAAAACATTATTCTGGTTGATCCTGCCAGTAGTTATATGCTTGTCTCAAAGATTA

**18S start**

Btry AGCCATGCATGTCTAAGTACAAACAAATTAAAAGTGAAACCGCAAAAGGCTCATTATATC

Bneo AGCCATGCATGTCTAAGTACAAACAAATTAAAAGTGAAACCGCAAAAGGCTCATTATATC

Bjar AGCCATGCATGTCTAAGTACAAGCAAATTAAAAGTGAAACCGCAAAAGGCTCATTATATC

Dmel AGCCATGCATGTCTAAGTACACACGAATTAAAAGTGAAACCGCAAAAGGCTCATTATATC

Btry AGTTATGGTTCCATAGATCGTTAACAGTTACTTGGATAACTGTGGTAATTCTAGAGCTAA

Bneo AGTTATGGTTCCATAGATCGTTAACAGTTACTTGGATAACTGTGGTAATTCTAGAGCTAA

Bjar AGTTATGGTTCCATAGATCGTTAACAGTTACTTGGATAACTGTGGTAATTCTGGAGCTAA

Dmel AGTTATGGTTCCTTAGATCGTTAACAGTTACTTGGATAACTGTGGTAATTCTAGAGCTAA

Btry TACATGCAAAATAAACACGGACCTTTTGGAACGTGTGCTTTTATTAGGCTAAAACCAAGC

Bneo TACATGCAAAATAAACACGGACCTTTTGGAACGTGTGCTTTTATTAGGCTAAAACCAAGC

Bjar TACATGCAAAATAAACACGGACCTTTTGGAACGTGTGCTTTTATTAGGCTAAAACCAAGC

Dmel TACATGCAATTAAAACATGAACCTTATGGGACATGTGCTTTTATTAGGCTAAAACCAAGC

Btry GATCGTAAGATCGTTATATTGGTTGAACTCTAGATAACTTGCAGATCGTATGGTCTCGTA

Bneo GATCGTAAGATCGTTATATTGGTTGAACTCTAGATAACTTGCAGATCGTATGGTCTCGTA

Bjar GATCGTAAGATCGTTATATTGGTTGAACTCTAGATAACTTGCAGATCGTATGTTCTCGTA

Dmel GATCGCAAGATCGTTATATTGGTTGAACTCTAGATAACATGCAGATCGTATGGTCTTGTA

Btry CCGACGACAGATCTTTCAAATGTCTGCCCTATCAACTTTTGATGGTAGTATCTAGGACTA

Bneo CCGACGACAGATCTTTCAAATGTCTGCCCTATCAACTTTTGATGGTAGTATCTAGGACTA

Bjar CCGACGACAGATCTTTCAAATGTCTGCCCTATCAACTTTTGATGGTAGTATCTAGGACTA

Dmel CCGACGACAGATCTTTCAAATGTCTGCCCTATCAACTTTTGATGGTAGTATCTAGGACTA

Btry CCATGGTTGCAACGGGTAACGGGGAATCAGGGTTCGATTCCGGAGAGGGAGCCTGAGAAA

Bneo CCATGGTTGCAACGGGTAACGGGGAATCAGGGTTCGATTCCGGAGAGGGAGCCTGAGAAA

Bjar CCATGGTTGCAACGGGTAACGGGGAATCAGGGTTCGATTCCGGAGAGGGAGCCTGAGAAA

Dmel CCATGGTTGCAACGGGTAACGGGGAATCAGGGTTCGATTCCGGAGAGGGAGCCTGAGAAA

Btry CGGCTACCACATCTAAGGAAGGCAGCAGGCGCGTAAATTACCCACTCCCAGTTCGGGGAG

Bneo CGGCTACCACATCTAAGGAAGGCAGCAGGCGCGTAAATTACCCACTCCCAGTTCGGGGAG

Bjar CGGCTACCACATCTAAGGAAGGCAGCAGGCACGTAAATTACCCACTCCCAGTTCGGGGAG

Dmel CGGCTACCACATCTAAGGAAGGCAGCAGGCGCGTAAATTACCCACTCCCAGCTCGGGGAG

Btry GTAGTGACGAAAAATAACAATACAGGACTCATATCCGAGGCCCTGTAATTGGAATGAGTA

Bneo GTAGTGACGAAAAATAACAATACAGGACTCATATCCGAGGCCCTGTAATTGGAATGAGTA

Bjar GTAGTGACGAAAAATAACAATACAGGACTCATATCCGAGGCCCTGTAATTGGAATGAGTA

Dmel GTAGTGACGAAAAATAACAATACAGGACTCATATCCGAGGCCCTGTAATTGGAATGAGTA

Btry CACTTTAAATCCTTTAACAAGGACCTATTGGAGGGCAAGTCTGGTGCCAGCAGCCGCGGT

Bneo CACTTTAAATCCTTTAACAAGGACCTATTGGAGGGCAAGTCTGGTGCCAGCAGCCGCGGT

Bjar TACTTTAAATCCTTTAACTAGGACCTATTGGAGGGCAAGTCTGGTGCCAGCAGCCGCGGT

Dmel CACTTTAAATCCTTTAACAAGGACCAATTGGAGGGCAAGTCTGGTGCCAGCAGCCGCGGT

Btry AATTCCAGCTCCAATAGCGTATATTAAAGTTGTTGCGGTTAAAACGTTCGTAGTTGAATT

Bneo AATTCCAGCTCCAATAGCGTATATTAAAGTTGTTGCGGTTAAAACGTTCGTAGTTGAATT

Bjar AATTCCAGCTCCAATAGCGTATATTAAAGTTGTTGCGGTTAAAACGTTCGTAGTTGAATT

Dmel AATTCCAGCTCCAATAGCGTATATTAAAGTTGTTGCGGTTAAAACGTTCGTAGTTGAACT

Btry TGTGCTTCATACGGGTAGTACAAC-TATAATTGTGGTATGTACATTACC-TTATGTATGT

Bneo TGTGCTTCATACGGGTAGTACAAC-TATAATTGTGGTATGTACATTACC-TTATGTATGT

Bjar TGTGCTTCATACGGGTAGTACAAC-TATAATTGTGGTATGTACATTACC-TTATGTATGT

Dmel TGTGCTTCATACGGGTAGTACAACTTACAATTGTGGTTAGTACTATACCTTTATGTATGT

Btry AAGCGTATTACCGGTGGAGTTCTTATATATAATTAATACAATGTATTTTTT-ATATATTC

Bneo AAGCGTATTACCGGTGGAGTTCTTATATATAATTAATACAATGTATTTTTT-ATATATTC

Bjar AAGCGTATTACCGGTGGAGTTCTTATATATAATTAATACAATGTATTTTTT-ATATATTC

Dmel AAGCGTATTACCGGTGGAGTTCTTATATGTGATTAAATACTTGTATTTTTTCATATGTTC

Btry CTCCTATTT--AAACCTGCTTCAGTGCTCTTCATCGAGTGTTGTTGTGGGCCGGTACAAT

Bneo CTCCTATTT--AAACCTGCTTCAGTGCTCTTCATCGAGTGTTGTTGTGGGCCGGTACAAT

Bjar CTCCTATTT--AAACCTGCTTCAGTGCTCTTCATCGAGTGTTGTTGTGGGCCGGTACAAT

Dmel CTCCTATTTAAAAACCTGCATTAGTGCTCTTAAACGAGTGTTATTGTGGGCCGGTACTAT

Btry TACTTTGAACAAATTAGAGTGCTTAAAGCAGGCTCCAAATGCCTGAATATTTTGTGCATG

Bneo TACTTTGAACAAATTAGAGTGCTTAAAGCAGGCTCCAAATGCCTGAATATTTTGTGCATG

Bjar TACTTTGAACAAATTAGAGTGCTTAAAGCAGGCTCCAAATGCCTGAATATTTTGTGCATG

Dmel TACTTTGAACAAATTAGAGTGCTTAAAGCAGGCTTCAAATGCCTGAATATTCTGTGCATG

Btry GAATAATGAAATAAGACCTCTGTTCTACTTTCATTGGTTTTTAGATCAAGAGGTAATGAT

Bneo GAATAATGAAATAAGACCTCTGTTCTACTTTCATTGGTTTTTAGATCAAGAGGTAATGAT

Bjar GAATAATGAAATAAGACCTCTGTTCTACTTTCATTGGTTTTTAGATCAAGAGGTAATGAT

Dmel GGATAATGAAATAAGACCTCTGTTCTGCTTTCATTGGTTTTCAGATCAAGAGGTAATGAT

Btry TAATAGAAGCAGTTTGGGGGCATTAGTATTACGACGCGAGAGGTGAAATTCTTGGACCGT

Bneo TAATAGAAGCAGTTTGGGGGCATTAGTATTACGACGCGAGAGGTGAAATTCTTGGACCGT

Bjar TAATAGAAGCAGTTTGGGGGCATTAGTATTACGACGCGAGAGGTGAAATTCTTGGACCGT

Dmel TAATAGAAGCAGTTTGGGGGCATTAGTATTACGACGCGAGAGGTGAAATTCTTGGACCGT

Btry CGTAAGACTAACTTAAGCGAAAGCATTTGCCAAAGATGTTTTCATTAATCAAGAACGAAA

Bneo CGTAAGACTAACTTAAGCGAAAGCATTTGCCAAAGATGTTTTCATTAATCAAGAACGAAA

Bjar CGTAAGACTAACTTAAGCGAAAGCATTTGCCAAAGATGTTTTCATTAATCAAGAACGAAA

Dmel CGTAAGACTAACTTAAGCGAAAGCATTTGCCAAAGATGTTTTCATTAATCAAGAACGAAA

Btry GTTAGAGGTTCGAAGGCGATCAGATACCGCCCTAGTTCTAACCATAAACGATGCCAGCTA

Bneo GTTAGAGGTTCGAAGGCGATCAGATACCGCCCTAGTTCTAACCATAAACGATGCCAGCTA

Bjar GTTAGAGGTTCGAAGGCGATCAGATACCGCCCTAGTTCTAACCATAAACGATGCCAGCTA

Dmel GTTAGAGGTTCGAAGGCGATCAGATACCGCCCTAGTTCTAACCATAAACGATGCCAGCTA

Btry GCAATTGGGTGTAGCTACTACTATGGCTCTCTCAGTCGCTTCCCGGGAAACCAAAGCTTT

Bneo GCAATTGGGTGTAGCTACTACTATGGCTCTCTCAGTCGCTTCCCGGGAAACCAAAGCTTT

Bjar GCAATTGGGTGTAGCTACTACTATGGCTCTCTCAGTCGCTTCCCGGGAAACCAAAGCTTT

Dmel GCAATTGGGTGTAGCTACTTTTATGGCTCTCTCAGTCGCTTCCCGGGAAACCAAAGCTTT

Btry TGGGCTCCGGGGGAAGTATGGTTGCAAAGCTGAAACTTAAAGGAATTGACGGAAGGGCAC

Bneo TGGGCTCCGGGGGAAGTATGGTTGCAAAGCTGAAACTTAAAGGAATTGACGGAAGGGCAC

Bjar TGGGCTCCGGGGGAAGTATGGTTGCAAAGCTGAAACTTAAAGGAATTGACGGAAGGGCAC

Dmel TGGGCTCCGGGGGAAGTATGGTTGCAAAGCTGAAACTTAAAGGAATTGACGGAAGGGCAC

Btry CACCAGGAGTGGAGCCTGCGGCTTAATTTGACTCAACACGGGAAAACTTACCAGGTCCGA

Bneo CACCAGGAGTGGAGCCTGCGGCTTAATTTGACTCAACACGGGAAAACTTACCAGGTCCGA

Bjar CACCAGGAGTGGAGCCTGCGGCTTAATTTGACTCAACACGGGAAAACTTACCAGGTCCGA

Dmel CACCAGGAGTGGAGCCTGCGGCTTAATTTGACTCAACACGGGAAAACTTACCAGGTCCGA

Btry ACATAAGCGTGTAAGACAGATTGATAGCTCTTTCTCGAATCTATGGGTGGTGGTGCATGG

Bneo ACATAAGCGTGTAAGACAGATTGATAGCTCTTTCTCGAATCTATGGGTGGTGGTGCATGG

Bjar ACATAAGCGTGTAAGACAGATTGATAGCTCTTTCTCGAATCTATGGGTGGTGGTGCATGG

Dmel ACATAAGTGTGTAAGACAGATTGATAGCTCTTTCTCGAATCTATGGGTGGTGGTGCATGG

Btry CCGTTCTTAGTTCGTGGAGTGATTTGTCTGGTTAATTCCGATAACGAACGAGACTCAAAT

Bneo CCGTTCTTAGTTCGTGGAGTGATTTGTCTGGTTAATTCCGATAACGAACGAGACTCAAAT

Bjar CCGTTCTTAGTTCGTGGAGTGATTTGTCTGGTTAATTCCGATAACGAACGAGACTCAAAT

Dmel CCGTTCTTAGTTCGTGGAGTGATTTGTCTGGTTAATTCCGATAACGAACGAGACTCAAAT

Btry ATATTAAATAGATGCTTTCAGGATTATGGTGTTGAAGCTTATATAGCCTTCATTCATGCG

Bneo ATATTAAATAGATGCTTTCAGGATTATGGTGTTGAAGCTTATATAGCCTTCATTCATGCG

Bjar ATATTAAATAGATGCTTTCAGGATTATGGTGTTGAAGCTTATATAGCCTTCATTCATGCG

Dmel ATATTAAATAGATATCTTCAGGATTATGGTGCTGAAGCTTATGTAGCCTTCATTCATG--

Btry TTCATCTTGAATGGACAA--------GTGTTTGAATGTGTTTATATAAGTGGAGTCGTAC

Bneo TTCATCTTGAATGGACAA--------GTGTTTGAATGTGTTTATATAAGTGGAGTCGTAC

Bjar TTCATCTTGAATGGACAA--------GTGTTTGAATGTGTTTATATAAGTGGAGTCGTAC

Dmel ------TTGGCAGTAAAATGCTTATTGTGTTTGAATGTGTTTATGTAAGTGGAGCCGTAC

Btry CTGTTGGTTTGTCCCATTATAAGGACACTAGCTTCTTAAATGGACAAATTGCGTCTAGCA

Bneo CTGTTGGTTTGTCCCATTATAAGGACACTAGCTTCTTAAATGGACAAATTGCGTCTAGCA

Bjar CTGTTGGTTTGTCCCATTATAAGGACACTAGCTTCTTAAATGGACAAATTGCGTCTAGCA

Dmel CTGTTGGTTTGTCCCATTATAAGGACACTAGCTTCTTAAATGGACAAATTGCGTCTAGCA

Btry GTAACGAGATTGAGCAATAACAGGTCTGTGATGCCCTTAGATGTCCTGGGCTGCACGCGC

Bneo GTAACGAGATTGAGCAATAACAGGTCTGTGATGCCCTTAGATGTCCTGGGCTGCACGCGC

Bjar GTAACGAGATTGAGCAATAACAGGTCTGTGATGCCCTTAGATGTCCTGGGCTGCACGCGC

Dmel ATAATGAGATTGAGCAATAACAGGTCTGTGATGCCCTTAGATGTCCTGGGCTGCACGCGC

Btry GCTACAATGAAAGTATCAACGTGTATTTCCTAGACCGAGAGGTCCGGGTAAACCGCTGAA

Bneo GCTACAATGAAAGTATCAACGTGTATTTCCTAGACCGAGAGGTCCGGGTAAACCGCTGAA

Bjar GCTACAATGAAAGTATCAACGTGTATTTCCTAGACCGAGAGGTCCGGGTAAACCGCTGAA

Dmel GCTACAATGAAAGTATCAACGTGTATTTCCTAGACCGAGAGGTCCGGGTAAACCGCTGAA

Btry CCACTTTCATGCTTGGGATTGTGAACTGAAACTGTTCACATGAACTTGGAATTCCCAGTA

Bneo CCACTTTCATGCTTGGGATTGTGAACTGAAACTGTTCACATGAACTTGGAATTCCCAGTA

Bjar CCACTTTCATGCTTGGGATTGTGAACTGAAACTGTTCACATGAACTTGGAATTCCCAGTA

Dmel CCACTTTCATGCTTGGGATTGTGAACTGAAACTGTTCACATGAACTTGGAATTCCCAGTA

Btry AGTGTGAGTCATTAACTCGCATTGATTACGTCCCTGCCCTTTGTACACACCGCCCGTCGC

Bneo AGTGTGAGTCATTAACTCGCATTGATTACGTCCCTGCCCTTTGTACACACCGCCCGTCGC

Bjar AGTGTGAGTCATTAACTCGCATTGATTACGTCCCTGCCCTTTGTACACACCGCCCGTCGC

Dmel AGTGTGAGTCATTAACTCGCATTGATTACGTCCCTGCCCTTTGTACACACCGCCCGTCGC

Btry TACTACCGATTGAATTATTTAGTGAGGTCTCCGGACGTGATCACTGTGACGCCTTGTGTT

Bneo TACTACCGATTGAATTATTTAGTGAGGTCTCCGGACGTGATCACTGTGACGCCTTGTGTT

Bjar TACTACCGATTGAATTATTTAGTGAGGTCTCCGGACGTGATCACTGTGACGCCTTGTGTT

Dmel TACTACCGATTGAATTATTTAGTGAGGTCTCCGGACGTGATCACTGTGACGCCTTGCGTG

Btry TCACGGTTGTTTCGCAAAAGTTGACCGAACTTGATTATTTAGAGGAAGTAAAAGTCGTAA

Bneo TCACGGTTGTTTCGCAAAAGTTGACCGAACTTGATTATTTAGAGGAAGTAAAAGTCGTAA

Bjar TCACGGTTGTTTCGCAAAAGTTGACCGAACTTGATTATTTAGAGGAAGTAAAAGTCGTAA

Dmel TTACGGTTGTTTCGCAAAAGTTGACCGAACTTGATTATTTAGAGGAAGTAAAAGTCGTAA

Btry CAAGGTTTCCGTAGGTGAACCTGCGGAAGGATCATTATTGTGTTCCTATCCGAAAAATAA

Bneo CAAGGTTTCCGTAGGTGAACCTGCGGAAGGATCATTATTGTGTTCCTATCCGAAAAATAA

Bjar CAAGGTTTCCGTAGGTGAACCTGCGGAAGGATCATTATTGTGTTCCTATCCGAAAAG---

Dmel CAAGGTTTCCGTAGGTGAACCTGCGGAAGGATCATTATTGTA-TAATATCCTTACCGTTA

**ITS1 start**

Btry ATATATAT---TAAAAAAAAAAAAAAAAACAA--AACAAAAAAAAAAAAAGAATAAAA--

Bneo ATATATAT---TAAAAAAAAAAAAAAAAACAA--A--AAAAAAAAAAAAAGAATAAAA--

Bjar --AAATAT---TAAAAAAAAAAAAAAAAACAA--AACAAAAAAAAAAAAAGAATAAAA--

Dmel ATAAATATTTGTAATTATACAAATAAAAACAATTTACCAAAATAAAAATATAACAAAATG

Btry -----AAGAAAAAAAAAAATTTTTTTTTTCTTTTTGTTCTTTTCATTCAATATGTTTTGA

Bneo -----AAGAAAAAAAAAATTTTTTTTTTTCTTTTTGTTCTTTTCATTCAATATGTTTTGA

Bjar -----AAGAAAAAAAAAAATTTTTTTTTTCTTTTTGTTCTTTTCATTCAATATGTTTTGA

Dmel ATTCCATGGAATCAAAAG----------------------TTAAAATCAAAATAAAACGA

Btry ATTATTGATAAATATTATTTATTTAAATCGATGTGGGTATATCAATAATTTGCATAAAAA

Bneo ATTATTGATAAATATTATTTATTTAAATCAATGTGGGTATATCAATAATTTGCATAAAAA

Bjar ATTATTGATAATAATTA-TTATTTAAATCGATATGGGTATATCAATAATTTGTATAAAAC

Dmel A-----GATGGGTTTTATTTATAT-AGTTAGTGTGGG--GCTTGGCAACCT-CATAAAAA

Btry ACATATTTGAGTGTTTTTCTTTTTTTTCTTTTTTTTTTTT--TTACTCCTTGTAATGCAT

Bneo ACATATTTGAGTGTTTTTCTTTTTTTTC-TTTTTTTTTTT--TTACTCCTTGTAATGCAT

Bjar ATATATTTGAATGTTTTTCTTTTTTTTC--TTTTTTTTTT--TTACTCCTTGTAATGCAT

Dmel G---ATTTTAACATTTCTAATGTATGTTGTGCGTATTTGTGGCGAGTACTTACAAC----

Btry TATGAGCAGTATATTTA--TACATATATTGTGAATTTCGCATACATTGTATTTGAACGCT

Bneo TATGAGCAGTATATTTA--TACATATATTGTGAATTTCGCATACATTGTATTTGAACGCT

Bjar TATGAGCAGTATATTTAATTACATATATTGTGAATATCGCATACATTGTATTTGAACGCT

Dmel ----AACGGCGT-------TTCCTATAAAAATAATGTTTCGAACATGAAAATCGAAG---

Btry -AAAAAACCTTTAAACATATATAGCTGTACTTATTATTTATAAAAAATTTTATAAAAAGT

Bneo -AAAAAACCTTTAAACATATATAGCTGTACTTATTATTTATAAAAAATTTTATAAAAAGT

Bjar AAAAAAACCTTTAAACATATATAGCTGTACTTATTATTTATAAAAAA-TTTATAAGAAGT

Dmel -AAACAAAATTCGAAAGT-------------------------GGAAGTCGAATCAAAAT

Btry TAAATGATCTT---TTTATAGTAAATAT----ATAAATGATAAGTTAATTTGTTCACATT

Bneo TAAATGATCTT---TTTATAGTAAATAT----ATAAATGATAAGTTAATTTGTTCACATT

Bjar TAAATGATCTT---TTTATAGAAAATAT----ATAAATGATAAGTTAATTTGTTCACATT

Dmel AAAATAATTTCGAATGTGTGGTAATCATCGAAATAAGTGTTAATATAATTGGTAGATATT

Btry AACGTGTAATTCCT----TTTTTATGGTATTTTCTATTTGTGATTAAGTATATGTTATAT

Bneo AACGTGTAATTCCT----TTTTTATGGTATTTTCTATTTGTGATTAAGTATATGTTATAT

Bjar AACGTGTAATTCCT----TTTTTATGGTATTTTCTATTTGTGATTAAGTATATGTTATAT

Dmel AAC---TAATTTTTAAAATTTGTGTG--------TATTTATTACTATACACGCGTTGCGA

Btry ACATATAAAATACATATATGTTATATAAATATATT-------------------------

Bneo ACATATAAAATACATATATGTTATATAAATATATT-------------------------

Bjar ACATATAATTT---TATATGTTATATACATATATT-------------------------

Dmel ATATGTATTGTTCATCTTAGTTATGGGCATACGTTGGCTAATGCAACAACCTGAAATAAA

Btry ---------------------TTGGTTTATTTTCATTCAAGAAATACTGGATTATGCACA

Bneo ---------------------TTGGTTTATTTTCATTCAAGAAATACTGGATTATGCACA

Bjar ---------------------GTGGATTATTTTTATTCAAGAAATACTGGAAAATGCACA

Dmel CAATGTTGTACCTGGCATCCATCAGGTTAATGTTTTATATAAATTGCAGTATGTGTCACC

Btry AATAAAAGAGA-----ATAGCTACTAAAAATGGTAT------TACTGTTTTTGTTGACCT

Bneo AATAAAAGAGA-----ATAGCTACTAAAAATGGTAT------TACTGTTTTTGTTGACCT

Bjar A--AGAAGAGA-----ATAACTAA-AAAAATGGTAT------TACTGTTTTTGTTGACCT

Dmel CAAAATAGCAAACCCCATAACCAACCAGATTATTATGATACATAATGCTTATATGAAACT

Btry AAGACA-TGCGCAGCTTGCAAATGTTTGGGTTTAAAATTACAATTTATTGAAAGATGTGT

Bneo AAGACA-TGCGCAGCTTGCAAATGTTTGGGTTTAAAATTACAATTTATTGAAAGATGTGT

Bjar AAGACA-TGCGCAGCTTGCAAATGTTTGGGTTTAAAATTACAATTTATTGAAAGATGTGT

Dmel AAGACATTTCGCAACATTTA---TTTTAGGTATATAAATAC-ATTTATTGAAGGA---AT

Btry --TGAAATTTTATTTTAAAAATTTGTTATAAATATTATTATTATGCTTTCAATAAATTAA

Bneo --TGAAATTTTATTTTAAAAATTTGTTATAAATATTATTATTATGCTTTCAATAAATTAA

Bjar TGTGAAATTTTATTTTAA-----------AAATATTATTATTATTCTTTCAATAAATTAA

Dmel --TGATATATGCCAGTAAAATGGTG-------TATTTTTAAT-TTCTTTCAATAA---AA

Btry AAACTCTTGACTTTGAATCAAAAAATACAAAAAAATTTTTACTCTAAGCGGTGGATCACT

Bneo AAACTCTTGACTTTGAATCAAAAAATACAAAAAAATTTTTACTCTAAGCGGTGGATCACT

Bjar AAACTCTTGACTTTGAATCAAAAAATAC-ATAAAATTTTTACTCTAAGCGGTGGATCACT

Dmel ACATAATTGACATTATATAAAAATGAATTATAAA------ACTCTAAGCGGTGGATCACT

**5.8S start**

Btry CGGCTCATGGGTCGATGAAGAACGCAGCAAACTGTGCGTCATCGTGTGAACTGCAGGACA

Bneo CGGCTCATGGGTCGATGAAGAACGCAGCAAACTGTGCGTCATCGTGTGAACTGCAGGACA

Bjar CGGCTCATGGGTCGATGAAGAACGCAGCAAACTGTGCGTCATCGTGTGAACTGCAGGACA

Dmel CGGCTCATGGGTCGATGAAGAACGCAGCAAACTGTGCGTCATCGTGTGAACTGCAGGACA

Btry CATGAACATCGACATTTTGAACGCATATTGCGGTCCATGCTGTTATGTACTTTAATTAAT

Bneo CATGAACATCGACATTTTGAACGCATATTGCGGTCCATGCTGTTATGTACTTTAATTAAT

Bjar CATGAACATCGACATTTTGAACGCATATTGCGGTCCATGCTGTTATGTACTTTAATTAAT

Dmel CATGAACATCGACATTTTGAACGCATATCGCAGTCCATGCTGTTATGTACTTTAATTAAT

Btry TTTAAAGTGCTGCTTGGACTACATATGGTTGAGGGTTGTAAGACTATGCTAAATTAGTTG

Bneo TTTAAAGTGCTGCTTGGACTACATATGGTTGAGGGTTGTAAGACTATGCTAAATTAGTTG

Bjar TTTAAAGTGCTGCTTGGACTACATATGGTTGAGGGTTGTAAGACTATGCAAAATTAGTTG

Dmel TTTATAGTGCTGCTTGGACTACATATGGTTGAGGGTTGTAAGACTATGCTAATTAAGTTG

**2S start** **ITS2 start**

Btry CTTATTCTTTTAGTTAATTAAAAGAATTTAAGCATATGGTATATTATTGGAT---TGTAT

Bneo CTTATTCTTTTAGTTAATTAAAAGAATTTAAGCATATGGTATATTATTGGAT---TGTAT

Bjar CTTATTCTTTTAGTTAATTAAAAGAATTTAAGCATATGGTATATTATTGGAT---TGTAT

Dmel CTTATAAATTT------TTA--------TAAGCATATGGTATATTATTGGATAAATATAA

Btry T--TTTCAATCCATAATATTAATAGCAT-AAAAAGAAATATATACAATATATTCTTGATT

Bneo T--TTTCAATCCATAATATTAATAGCAT-AAAAAGAAATATATACAATATATTCTTGATT

Bjar T--TTTCAATCCATAATATTAATAGCATAAAAAAGAAATATATACAATATATTCTTGAAT

Dmel TAATTTTTATTCATAATATTAA-AAAATAAATGAAAAACAT------------------T

Btry ACCTCATATTTGAACGAAATTTTATAATAAATGAGAATCTTAGTATTCCCAAAATAAAAA

Bneo ACCTCATATTTGAACGAAATTTTATAATAAATGAGAATCTTAGTATTCCCAAAATAAAAA

Bjar ACCTCATTGTTGAACGAAATTTTATAATAAATGAGAATCTTAGTATTCCCAAAATAAAGA

Dmel ATCTCACATTTGAATGTGA-------------------------------AAAACGAAGA

Btry AAATTTCAATATTATTTAAAATATATTTAAATAAAT-ACATT-AAGAGGAAAGTCTAGCA

Bneo AAAATTCAATATTATTTAAAATATATTTAAATAAAT-ACATT-AAGAGGAAAGTCTAGCA

Bjar AAATTTCAATATTATTTAAAATATATTTAAATAAATAACATTAAAGAGGAAAGTCTAGCA

Dmel GA-----AATATTTTCT------TTTTCAATCAAATAATACT---GAGAAATGTCTAGCA

Btry TAAAAATTT----TATTTGTGGTCTAGACCTTATGTCCCTAATGTATTTATTTAAAAAGT

Bneo TAAAAATTT----TATTTGTGGTCTAGACCTTATGTCCCTAATGTATTTATTTAAAAAGT

Bjar TAAAAATTT----TATTTGTGGTCTAGACCTTATGTCCCTAATGTATTTATTTAAAAAGT

Dmel TAAAAAATTGAAATATTTTTCATCTAGAAT---TGTCTCTTAT-TAATGATTCGGAAATA

Btry GGAAGT-GATGATAAA-ATA-TATTT--TAATAATATATGAAAT--AAATATATTAATAA

Bneo GGAAGT-GATGATAAA-ATA-TATTT--TAATAATATATGAAAT--AAATATATTAATAA

Bjar GGAAGTGGATGAAAAATATA-TATTTTATAATAATATATGAAATAAAAATATATTAATAA

Dmel GAAAAATCTTGGTTATGTTATTATTCTTCGTTGGTTCGTTAAAAATGGATAAATAAAAAC

Btry TTTGAATTGGTGGGATGAAAAGATTTTATATTTATTATTAGAGATTTATAAAATGGTATC

Bneo TTTGAATTGGTGGGATGAAAAGATTTTATATTTATTATTAAAGATTTATAAAATGGTATC

Bjar TTTGAAT---TGGGATGAAAAGATTTTATATTTATTATTAGAAATTTATAAAATGGTATC

Dmel TTTGCAT----------ACAAGAATTAATA-------------------AAAATGTTATA

Btry GAAGATATAA-AATATATTTCTTTAAAATAGCGAAAAAATAAAAGAGTT---ATTAATAT

Bneo GAAGATATAA-AATATATTTCTTTAAAATAGCGAAAAAATAAAAGAGTTATTATTAATAT

Bjar G-AGATATAA-AATATGTTTCTTTAAAATAGCGAAAAAATAAAAGAAT-----------T

Dmel ACGAATTTAATTAAATGTTTTATCATTATATATAAAGAATTTATGGC-------------

Btry ATATATAATATAAAAAATTAATTTTTTTTATACAACCTCAACTCATATGGGACTACCCCC

Bneo ATATATAATATAAAAAATTAATTTTTTTTATACAACCTCAACTCATATGGGACTACCCCC

Bjar ATAAAAAAAAGAAAAA--------TTTTTATACAACCTCAACTCATATGGGACTACCCCC

Dmel ------AAGATAAAG----------TTATATACAACCTCAACTCATATGGGACTACCCCC

**28S start**

Btry TGAATTTAAGCATATTAATGAGGGGAGGAAAAGAAACTAACAAGGATTTTCTTAGTAGCG

Bneo TGAATTTAAGCATATTAATGAGGGGAGGAAAAGAAACTAACAAGGATTTTCTTAGTAGCG

Bjar TGAATTTAAGCATATTAATGAGGGGAGGAAAAGAAACTAACAAGGATTTTCTTAGTAGCG

Dmel TGAATTTAAGCATATTAATTAGGGGAGGAAAAGAAACTAACAAGGATTTTCTTAGTAGCG

Btry GCGAGCGAAAAGAAAATAGTTCAGCACTAAGTCACTTTGTCTATATGGCAAATGTGAGAT

Bneo GCGAGCGAAAAGAAAATAGTTCAGCACTAAGTCACTTTGTCTATATGGCAAATGTGAGAT

Bjar GCGAGCGAAAAGAAAATAGTTCAGCACTAAGTCACTTTGTCTATATGGCAAATGTGAGAT

Dmel GCGAGCGAAAAGAAAACAGTTCAGCACTAAGTCACTTTGTCTATATGGCAAATGTGAGAT

Btry GCAGTGTATGGAATATCTTAATA-TCTAGTATGAGAAATTAACGATTTAAGTCCTTCTTA

Bneo GCAGTGTATGGAATATCTTAATA-TCTAGTATGAGAAATTAACGATTTAAGTCCTTCTTA

Bjar GCAGTGTATGGAATATCTTAATA-TCTAGTATGAGAAATTAACGATTTAAGTCCTTCTTA

Dmel GCAGTGTATGGAGCGTC--AATATTCTAGTATGAGAAATTAACGATTTAAGTCCTTCTTA

Btry AATGAGGCCATTTACCCATAGAGGGTGCCAGGCCCGTATAACGTTAATGATTACTAGAAA

Bneo AATGAGGCCATTTACCCATAGAGGGTGCCAGGCCCGTATAACGTTAATGATTACTAGAAA

Bjar AATGAGGCCATTTACCCATAGAGGGTGCCAGGCCCGTATAACGTTAATGATTACTAGAAA

Dmel AATGAGGCCATTTACCCATAGAGGGTGCCAGGCCCGTATAACGTTAATGATTACTAG-AT

Btry GATATTTCCAAAGAGTCGTGTTGCTTGATAGTGCAGCACTAAGTGGGTGGTAAACTCCAT

Bneo GATATTTCCAAAGAGTCGTGTTGCTTGATAGTGCAGCACTAAGTGGGTGGTAAACTCCAT

Bjar GATATTTCCAAAGAGTCGTGTTGCTTGATAGTGCAGCACTAAGTGGGTGGTAAACTCCAT

Dmel GATGTTTCCAAAGAGTCGTGTTGCTTGATAGTGCAGCACTAAGTGGGTGGTAAACTCCAT

Btry CTAAAACTAAATATAACCATGAGACCGATAGTAAACAAGTACCGTGAGGGAAAGTTGAAA

Bneo CTAAAACTAAATATAACCATGAGACCGATAGTAAACAAGTACCGTGAGGGAAAGTTGAAA

Bjar CTAAAACTAAATATAACCATGAGACCGATAGTAAACAAGTACCGTGAGGGAAAGTTGAAA

Dmel CTAAAACTAAATATAACCATGAGACCGATAGTAAACAAGTACCGTGAGGGAAAGTTGAAA

Btry AGAACTCTGAATAGAGAGTTAAATAGTACGTGAAACTGCTTAGAGGTTAAGCCCGATGAA

Bneo AGAACTCTGAATAGAGAGTTAAATAGTACGTGAAACTGCTTAGAGGTTAAGCCCGATGAA

Bjar AGAACTCTGAATAGAGAGTTAAATAGTACGTGAAACTGCTTAGAGGTTAAGCCCGATGAA

Dmel AGAACTCTGAATAGAGAGTTAAACAGTACGTGAAACTGCTTAGAGGTTAAGCCCGATGAA

Btry CCTGAATATCCATTATGAAAAATTCATCATTATAACTGTGGTATTTAATTTTAAATATCA

Bneo CCTGAATATCCATTATGAAAAATTCATCATTATAACTGTGGTATTTAATTTTAAATATCA

Bjar CCTGAATATCCATTATGAAAAATTCATCATTATAACTGTAGTATTTAATTTTAAATATTA

Dmel CCTGAATATCCGTTATGGAAAATTCATCATTAAAATTGTAATATTTAAAT---AATATTA

Btry TAATAATAGTGTGCATTTTTTTCATATAAGGACATTGTAATCTATTAACATAATAAAGTA

Bneo TAATAATAGTGTGCATTTTTTTCATATAAGGACATTGTAATCTATTAACATAATAAAGTA

Bjar TAGTAATAGTGTGCATTTTTTTCATATAAGGACATTGTAATCTATTAACATAATAAAGTA

Dmel TGAGAATAGTGTGCATTTTTTCCATATAAGGACATTGTAATCTATTAGCAT-ATACCAAA

Btry TTTATCAAAAGATCATTGGTTTTAAGTTTATTCAAATTAATTTGCTTTTAGCTTATTAAC

Bneo TTTATCAAAAGATCATTGGTTTTAAGTTTATTCAAATTAATTTGCTTTTAGCTTATTAAC

Bjar TTTATCAAAAGATCATTGGTTTTAAGTTTATTCAAATTAATTTGCTTTTAGCTTATTAAC

Dmel TTTATCATAAAATA--TAACTTATAGTTTATTCCAATTAAATTGCTT---GCATTTTAAC

Btry ATAGAATAAATACTGATGATTTGATAAAGTGTTGATAGATTT-TATTATATATAATGCTA

Bneo ATAGAATAAATACTGATGATTTGATAAAGTGTTGATAGATTT-TATTATATATAATGCTA

Bjar ATAGAATAAATACTGATGATTTGATAAAGTGTTGATAGATTT-TATTATATATAATGCTA

Dmel ACAGAATAAATGTTATTAATTTGATAAAGTGCTGATAGATTTATATGAT-TACAGTGCGT

Btry AAATTCTTTTGAATTTTACAATAATATTATTATCATTGATTTTAATATTAATTGTATGCA

Bneo AAATTCTTTTGAATTTTACAATAATATTATTATCATTGATTTTAATATTAATTGTATGCA

Bjar AAATTCTTTTGAATTTTACAATAATATTATTATCATTGATTTTAATATTAATTGTATGCA

Dmel TAATTTTTCGGAATTATATAATGGCATAATTATCATTGATTTTTGTGTTTATTATATGCA

Btry TTTATATGATTAACAATGCGAAAGATTCAGGATACCTTCGGGACCCGTCTTGAAACACGG

Bneo TTTATATGATTAACAATGCGAAAGATTCAGGATACCTTCGGGACCCGTCTTGAAACACGG

Bjar TTTATATGATTAACAATGCGAAAGATTCAGGATACCTTCGGGACCCGTCTTGAAACACGG

Dmel CTTGTATGATTAACAATGCGAAAGATTCAGGATACCTTCGGGACCCGTCTTGAAACACGG

Btry ACCAAGGAGTCTAACATATGTGCAAGTCATTGGGTTATATTAAACCTAATGGCGTAATTA

Bneo ACCAAGGAGTCTAACATATGTGCAAGTCATTGGGTTATATTAAACCTAATGGCGTAATTA

Bjar ACCAAGGAGTCTAACATATGTGCAAGTCATTGGGTTATATTAAACCTAATGGCGTAATTA

Dmel ACCAAGGAGTCTAACATATGTGCAAGTTATTGGGATA---TAAACCTAATAGCGTAATTA

Btry ACTTAACTG--TATGGGATTAATTTTTAGTGTAT---TACACTATTAATTCAATCCCGGG

Bneo ACTTAACTG--TATGGGATTAATTTTTAGTGTAT---TACACTATTAATTCAATCCCGGG

Bjar ACTTAACTG--TATGGGATTAATTTTTAGTGTAT---TACACTATTAATTCAATCCCGGG

Dmel ACTTGACTAATAATGGGATTAGTTTTTTAGCTATTTATAGCTAATTAACACAATCCCGGG

Btry GCGTTCCATATAGTTATGTATAATGATAATTTATTATTATTTATACCTCTAACTGGAGCG

Bneo GCGTTCCATATAGTTATGTATAATGATAATTTATTATTATTTATACCTCTAACTGGAGCG

Bjar GCGTTCTATATAGTTATGTATAATGATAATTTATTATTATTTATACCTCTAACTGGAGCG

Dmel GCGTTCTATATAGTTATGTATAATGTATATTTA-TATTATTTATGCCTCTAACTGGAACG

Btry TACCTTGAGCATATATGCTGTGACCCGAAAGATGGTGAACTATACTTGATCAGGTTGAAG

Bneo TACCTTGAGCATATATGCTGTGACCCGAAAGATGGTGAACTATACTTGATCAGGTTGAAG

Bjar TACCTTGAGCATATATGCTGTGACCCGAAAGATGGTGAACTATACTTGATCAGGTTGAAG

Dmel TACCTTGAGCATATATGCTGTGACCCGAAAGATGGTGAACTATACTTGATCAGGTTGAAG

Btry TCAGGGGAAACCCTGATGGAAGACCGAAACAGTTCTGACGTGCAAATCGATTGTCAGAAT

Bneo TCAGGGGAAACCCTGATGGAAGACCGAAACAGTTCTGACGTGCAAATCGATTGTCAGAAT

Bjar TCAGGGGAAACCCTGATGGAAGACCGAAACAGTTCTGACGTGCAAATCGATTGTCAGAAT

Dmel TCAGGGGAAACCCTGATGGAAGACCGAAACAGTTCTGACGTGCAAATCGATTGTCAGAAT

Btry TGAGTATAGGGGCGAAAGACCAATCGAACCATCTAGTAGCTGGTTCCCTCCGAAGTTTCC

Bneo TGAGTATAGGGGCGAAAGACCAATCGAACCATCTAGTAGCTGGTTCCCTCCGAAGTTTCC

Bjar TGAGTATAGGGGCGAAAGACCAATCGAACCATCTAGTAGCTGGTTCCCTCCGAAGTTTCC

Dmel TGAGTATAGGGGCGAAAGACCAATCGAACCATCTAGTAGCTGGTTCCTTCCGAAGTTTCC

Btry CTCAGGATAGCTGGTGCATTTAAATATTATGTAAAGTAATCTTATCTGGTAAAGCGAATG

Bneo CTCAGGATAGCTGGTGCATTTAAATATTATGTAAAGTAATCTTATCTGGTAAAGCGAATG

Bjar CTCAGGATAGCTGGTGCATTTAAATATTATGTAAAATAATCTTATCTGGTAAAGCGAATG

Dmel CTCAGGATAGCTGGTGCATTTTAATATTATATAAAATAATCTTATCTGGTAAAGCGAATG

Btry ATTAGAGGCCTTAGGGTCGAAACGACCTTAACCTATTCTCAAACTTTAAATGGGTAAGAA

Bneo ATTAGAGGCCTTAGGGTCGAAACGACCTTAACCTATTCTCAAACTTTAAATGGGTAAGAA

Bjar ATTAGAGGCCTTAGGGTCGAAACGACCTTAACCTATTCTCAAACTTTAAATGGGTAAGAA

Dmel ATTAGAGGCCTTAGGGTCGAAACGATCTTAACCTATTCTCAAACTTTAAATGGGTAAGAA

Btry CCTCACCTTTCTTGATATGAAGGTTGAGGTTATGATATAATGTGCCCAGTGGGCCACTTT

Bneo CCTCACCTTTCTTGATATGAAGGTTGAGGTTATGATATAATGTGCCCAGTGGGCCACTTT

Bjar CCTCACCTTTCTTGATATGAAGGTTGAGGTTATGATATAATGTGCCCAGTGGGCCACTTT

Dmel CCTTAACTTTCTTGATATGAAGTTCAAGGTTATGATATAATGTGCCCAGTGGGCCACTTT

Btry TGGTAAGCAGAACTGGCGCTGTGGGATGAACCAAACGTAATGTTACGGTGCCTAAATTAA

Bneo TGGTAAGCAGAACTGGCGCTGTGGGATGAACCAAACGTAATGTTACGGTGCCTAAATTAA

Bjar TGGTAAGCAGAACTGGCGCTGTGGGATGAACCAAACGTAATGTTACGGTGCCTAAATTAA

Dmel TGGTAAGCAGAACTGGCGCTGTGGGATGAACCAAACGTAATGTTACGGTGCCCAAATTAA

Btry CAACTCATGCAGATACCATGAAAGGCGTTGGTTGCTTAAAACAGCAGGACGGTGGACATG

Bneo CAACTCATGCAGATACCATGAAAGGCGTTGGTTGCTTAAAACAGCAGGACGGTGGACATG

Bjar CAACTCATGCAGATACCATGAAAGGCGTTGGTTGCTTAAAACAGCAGGACGGTGGACATG

Dmel CAACTCATGCAGATACCATGAAAGGCGTTGGTTGCTTAAAACAGCAGGACGGTGATCATG

Btry GAAGTCGTAATCCGCTAAGGAGTGTGTAACAACTCACCTGCCGAAGCAACTAGCCCTTAA

Bneo GAAGTCGTAATCCGCTAAGGAGTGTGTAACAACTCACCTGCCGAAGCAACTAGCCCTTAA

Bjar GAAGTCGTAATCCGCTAAGGAGTGTGTAACAACTCACCTGCCGAAGCAACTAGCCCTTAA

Dmel GAAGTCGAAATCCGCTAAGGAGTGTGTAACAACTCACCTGCCGAAGCAACTAGCCCTTAA

Btry AATGGATGGCGCTTAAGTTGTATACCTATACATTACCGCTAAAGTAGATGATTTATAATA

Bneo AATGGATGGCGCTTAAGTTGTATACCTATACATTACCGCTAAAGTAGATGATTTATAATA

Bjar AATGGATGGCGCTTAAGTTGTATACCTATACATTACCGCTAAAGTAGATGATTTATAATA

Dmel AATGGATGGCGCTTAAGTTGTATACCTATACATTACCGCTAAAGTAGATGATTTATATTA

Btry CAATTTCGGTTGGATTATAAATTTTGAAACTTTAGTGAGTAGGAGGGTACAATGGTGTGC

Bneo CAATTTCGGTTGGATTATAAATTTTGAAACTTTAGTGAGTAGGAGGGTACAATGGTGTGC

Bjar CAATTTCGGTTGGATTATAAATTTTGAAACTTTAGTGAGTAGGAGGGTACAATGGTGTGC

Dmel C--------TTGTGATATAAATTTTGAAACTTTAGTGAGTAGGAAGGTACAATGGTATGC

Btry TTAGAAGTGTTTGGCGTAAGCCTGCATGGAGCCGCTATTGGTACAGATCTTGGTGGTAGT

Bneo TTAGAAGTGTTTGGCGTAAGCCTGCATGGAGCCGCTATTGGTACAGATCTTGGTGGTAGT

Bjar TTAGAAGTGTTTGGCGTAAGCCTGCATGGAGCCGCTATTGGTACAGATCTTGGTGGTAGT

Dmel GTAGAAGTGTTTGGCGTAAGCCTGCATGGAGCTGCCATTGGTACAGATCTTGGTGGTAGT

Btry AGCAAATAATCGAATGAGACCTTGGAGGACTGAAGTGGAGAAGGGTTTCGTGTGAACAGT

Bneo AGCAAATAATCGAATGAGACCTTGGAGGACTGAAGTGGAGAAGGGTTTCGTGTGAACAGT

Bjar AGCAAATAATCGAATGAGACCTTGGAGGACTGAAGTGGAGAAGGGTTTCGTGTGAACAGT

Dmel AGCAAATAATCGAATGAGACCTTGGAGGACTGAAGTGGAGAAGGGTTTCGTGTGAACAGT

Btry GGTTGATCACGAGTTAGTCGGTCCTAAGTTCAAGGCGAAAGCCGAAAATTTTCAAGTTTT

Bneo GGTTGATCACGAGTTAGTCGGTCCTAAGTTCAAGGCGAAAGCCGAAAATTTTCAAGTTTT

Bjar GGTTGATCACGAGTTAGTCGGTCCTAAGTTCAAGGCGAAAGCCGAAAATTTTCAAGTTTT

Dmel GGTTGATCACGAGTTAGTCGGTCCTAAGTTCAAGGCGAAAGCCGAAAATTTTCAAGTAAA

Btry AATGAAATGAAGTGAAA--TTGAATTTTTTTATTTCATAG----TAATTAAACACTTGAA

Bneo AATGAAATGAAGTGAAA--TTGAATTTTTTTATTTCATAG----TAATTAAACACTTGAA

Bjar AATGAAATGAAGTGGAAATTTAATTTTTTTTATTTCATAG----TAATTAAACACTTGAA

Dmel ACAAAAATGC---------CTAACTATATAAA---CAAAGCGAATTATAATACACTTGAA

Btry TAATTTTGAACGAAAGGGAATACGGTTCCAATTCCGTAACCTGTTGAGTATCCGTTTGTT

Bneo TAATTTTGAACGAAAGGGAATACGGTTCCAATTCCGTAACCTGTTGAGTATCCGTTTGTT

Bjar TAATTTTGAACGAAAGGGAATACGGTTCCAATTCCGTAACCTGTTGAGTATCCGTTTGTT

Dmel TAATTTTGAACGAAAGGGAATACGGTTCCAATTCCGTAACCTGTTGAGTATCCGTTTGTT

Btry ATTAAAAATGGGCCTTGTGCTCATCCTGGCAACAGGAACGACCATAAAGAAGCCGTCGAG

Bneo ATTAAAAATGGGCCTTGTGCTCATCCTGGCAACAGGAACGACCATAAAGAAGCCGTCGAG

Bjar ATTAAAAATGGGCCTTGTGCTCATCCTGGCAACAGGAACGACCATAAAGAAGCCGTCGAG

Dmel ATTAAATATGGGCCTCGTGCTCATCCTGGCAACAGGAACGACCATAAAGAAGCCGTCGAG

Btry AGGTATCGGAAGAGTTTTCTTTTCTGTTTTATAGTCGTACTACCATGGAAGTCTTTCGAA

Bneo AGGTATCGGAAGAGTTTTCTTTTCTGTTTTATAGTCGTACTACCATGGAAGTCTTTCGAA

Bjar AGGTATCGGAAGAGTTTTCTTTTCTGTTTTATAGTCGTACTACCATGGAAGTCTTTCGAA

Dmel AGATATCGGAAGAGTTTTCTTTTCTGTTTTATAGCCGTACTACCATGGAAGTCTTTCGCA

Btry GAGAGATATGGTAGATGGACTAGAAGAGCATGACATTTACTGTTGTGTCGATATTTTCTC

Bneo GAGAGATATGGTAGATGGACTAGAAGAGCATGACATTTACTGTTGTGTCGATATTTTCTC

Bjar GAGAGATATGGTAGATGGGCTAGAAGAGCATGACATTTACTGTTGTGTCGATATTTTCTC

Dmel GAGAGATATGGTAGATGGGCTAGAAGAGCATGACATATACTGTTGTGTCGATATTTTCTC

Btry CTCGGACCTTGAAAATTTATGGTGGGGTTACGCAAACTTCTCAACAGGCCGTACCAATAT

Bneo CTCGGACCTTGAAAATTTATGGTGGGGTTACGCAAACTTCTCAACAGGCCGTACCAATAT

Bjar CTCGGACCTTGAAAATTTATGGTGGGGTTACGCAAACTTCTCAACAGGCCGTACCAATAT

Dmel CTCGGACCTTGAAAATTTATGGTGGGGACACGCAAACTTCTCAACAGGCCGTACCAATAT

Btry CCGCAGCTGGTCTCCAAGGTGAAGAGTCTCTAGTCGATAGAATAATGTAGGTAAGGGAAG

Bneo CCGCAGCTGGTCTCCAAGGTGAAGAGTCTCTAGTCGATAGAATAATGTAGGTAAGGGAAG

Bjar CCGCAGCTGGTCTCCAAGGTGAAGAGTCTCTAGTCGATAGAATAATGTAGGTAAGGGAAG

Dmel CCGCAGCTGGTCTCCAAGGTGAAGAGTCTCTAGTCGATAGAATAATGTAGGTAAGGGAAG

Btry TCGGCAAATTAGATCCGTAACTTCGGGATAAGGATTGGCTCTGAAGATTGAGATAGTCGG

Bneo TCGGCAAATTAGATCCGTAACTTCGGGATAAGGATTGGCTCTGAAGATTGAGATAGTCGG

Bjar TCGGCAAATTAGATCCGTAACTTCGGGATAAGGATTGGCTCTGAAGATTGAGATAGTCGG

Dmel TCGGCAAATTAGATCCGTAACTTCGGGATAAGGATTGGCTCTGAAGATTGAGATAGTCGG

Btry GCTTGATTGGGAAGCAATACCATGGTTTATGTACTCGTTCTGGGTAAATAG-------AG

Bneo GCTTGATTGGGAAGCAATACCATGGTTTATGTACTCGTTCTGGGTAAATAG-------AG

Bjar GCTTGATTGGGAAGCAATACCATGGTTTATGTACTCGTTCTGGGTAAATAG-------AG

Dmel GCTTGATTGGGAAACAATAACATGGTTTATGTGCTCGTTCTGGGTAAATAGAGTTTCTAG

Btry AATTTCGGTT-----CTTGTTCCCCGGATAG--TAGTTACGTAGCCAATTGTGGAACTTT

Bneo AATTTCGGTT-----CTTGTTCCCCGGATAG--TAGTTACGTAGCCAATTGTGGAACTTT

Bjar AATTTCGGTT-----CTTGTTCCCCGGATAG--TAGTTACGTAGCCAATTGTGGAACTTT

Dmel CATTTATGTTAGTTACTTGTTCCCCGGATAGTTTAGTTACGTAGCCAATTGTGGAACTTT

Btry CTTGCTAAAATTTTTAA-AGGATTATATCG--TAAGATATATATTCTTTTTAAATTATAA

Bneo CTTGCTAAAATTTTTAA-AGGATTATATCG--TAAGATATATATTCTTTTTAAATTATAA

Bjar CTTGCTAAAATTTTTAA-AGGATTATATCG--TAAGATATGTATTCTTTTTAAATTATAA

Dmel CTTGCTAAAATTTTTAAGAATACTATTTGGGTTAAACCAATTAGTTCTTATTAATTATAA

Btry CGATTATCAATTAACAATCAATTCAGAACTGGCACGGACTTGGGGAATCCGACTGTCTAA

Bneo CGATTATCAATTAACAATCAATTCAGAACTGGCACGGACTTGGGGAATCCGACTGTCTAA

Bjar CGATTATCAATTAACAATCAATTCAGAACTGGCACGGACTTGGGGAATCCGACTGTCTAA

Dmel CGATTATCAATTAACAATCAATTCAGAACTGGCACGGACTTGGGGAATCCGACTGTCTAA

Btry TTAAAACAAAGCATTGTGATGGCCCTAACGGGTGTTGACACAATGTGATTTCTGCCCAGT

Bneo TTAAAACAAAGCATTGTGATGGCCCTAACGGGTGTTGACACAATGTGATTTCTGCCCAGT

Bjar TTAAAACAAAGCATTGTGATGGCCCTAACGGGTGTTGACACAATGTGATTTCTGCCCAGT

Dmel TTAAAACAAAGCATTGTGATGGCCCTAGCGGGTGTTGACACAATGTGATTTCTGCCCAGT

Btry GCTCTGAATGTCAAAGTGAAGAAATTCAAGTAAGCGCGGGTAAACGGCGGGAGTAACTAT

Bneo GCTCTGAATGTCAAAGTGAAGAAATTCAAGTAAGCGCGGGTAAACGGCGGGAGTAACTAT

Bjar GCTCTGAATGTCAAAGTGAAGAAATTCAAGTAAGCGCGGGTAAACGGCGGGAGTAACTAT

Dmel GCTCTGAATGTCAAAGTGAAGAAATTCAAGTAAGCGCGGGTCAACGGCGGGAGTAACTAT

Btry GACTCTCTTAAGGTAGCCAAATGCCTCGTCATCTAATTAGTGACGCGCATGAATGGATTA

Bneo GACTCTCTTAAGGTAGCCAAATGCCTCGTCATCTAATTAGTGACGCGCATGAATGGATTA

Bjar GACTCTCTTAAGGTAGCCAAATGCCTCGTCATCTAATTAGTGACGCGCATGAATGGATTA

Dmel GACTCTCTTAAGGTAGCCAAATGCCTCGTCATCTAATTAGTGACGCGCATGAATGGATTA

Btry ACGAGATTCCCTCTGTCCCTATCTACTATCTAGCGAAACCACAGCCAAGGGAACGGGCTT

Bneo ACGAGATTCCCTCTGTCCCTATCTACTATCTAGCGAAACCACAGCCAAGGGAACGGGCTT

Bjar ACGAGATTCCCTCTGTCCCTATCTACTATCTAGCGAAACCACAGCCAAGGGAACGGGCTT

Dmel ACGAGATTCCTACTGTCCCTATCTACTATCTAGCGAAACCACAGCCAAGGGAACGGGCTT

Btry GGAATAATTAGCGGGGAAAGAAGACCCTGTTGAGCTTGACTCTAGTCTGGCAGTGTAAGG

Bneo GGAATAATTAGCGGGGAAAGAAGACCCTGTTGAGCTTGACTCTAGTCTGGCAGTGTAAGG

Bjar GGAATAATTAGCGGGGAAAGAAGACCCTGTTGAGCTTGACTCTAGTCTGGCAGTGTAAGG

Dmel GGAATAATTAGCGGGGAAAGAAGACCCTTTTGAGCTTGACTCTAATCTGGCAGTGTAAGG

Btry AGACATAAGAGGTGTAGCATAAGTGGGAGATATATAATTTCGGTTATATATCAACAATGA

Bneo AGACATAAGAGGTGTAGCATAAGTGGGAGATATATAATTTCGGTTATATATCAACAATGA

Bjar AGACATAAGAGGTGTAGCATAAGTTGGCGATATATAATTTCGGTTATATATCAACAATGA

Dmel AGACATAAGAGGTGTAGAATAAGTGGGAGATATTAGACCTCGGTTTGGTATCGTCAATGA

Btry AATACCACTACTCTTATTGTTTCCTTACTTACTTGATTAAGTGGAACGTGTATCATTGCT

Bneo AATACCACTACTCTTATTGTTTCCTTACTTACTTGATTAAGTGGAACGTGTATCATTGCT

Bjar AATACCACTACTCTTATTGTTTCCTTACTTACTTGATTAAGTGCAACGTGTATCATTGCT

Dmel AATACCACTACTCTTATTGTTTCCTTACTTACTTGATTAAATGGAACGTGTATCATTTCC

Btry TAGCCATTATA-TGGGTATATTTA-TATATCTTATGGTATTGGGTTTTGATGCAAGCTTC

Bneo TAGCCATTATA-TGGGTATATTTA-TATATCTTATGGTATTGGGTTTTGATGCAAGCTTC

Bjar TAGCCATTATATTGGGTATATTTA-TATATCTTATGGTATTGGGTTTTGATGCAAGCTTC

Dmel TAGCCATTATA-CGGATATATTTATTATATCTTATGGTATTGGGTTTTGATGCAAGCTTC

Btry TTGATCAAAGTACCACGAGTTTGTTATATAATTGTAAACTTAT--TTTAATGAAATGATA

Bneo TTGATCAAAGTACCACGAGTTTGTTATATAATTGTAAACTTAT--TTTAATGAAATGATA

Bjar TTGATCAAAGTACCACGAGTTTGTTATATAATTGTAAACTTAT--GTTAATGAAATGATA

Dmel TTGATCAAAGTATCACGAGTTTGTTATATAATCGCAAACAAATTCTTTAATAAAACGAT-

Btry GCATTTCGGTGTTATTATTATAATTAAAATTTGGTATAACTCCAA-CACTCAGGTATGAT

Bneo GCATTTCGGTGTTATTATTATAATTAAAATTTGGTATAACTCCAA-CACTCAGGTATGAT

Bjar GCATTTCGGTGTTATTATTATAATTAAAATTTGGTATAACTCCAA-CACTCAGGTATGAT

Dmel GCATTTATGTATTTTTGATTTG---AAAATTTGGTATAACTCCAATTACTCAGGTATGAT

Btry CCAATTCAAGGACATTGCCAGGTGGGGAGTTTGACTGGGGCGGTACATCTCTCAAATAAT

Bneo CCAATTCAAGGACATTGCCAGGTGGGGAGTTTGACTGGGGCGGTACATCTCTCAAATAAT

Bjar CCAATTCAAGGACATTGCCAGGTGGGGAGTTTGACTGGGGCGGTACATCTCTCAAATAAT

Dmel CCAATTCAAGGACATTGCCAGGTAGGGAGTTTGACTGGGGCGGTACATCTCTCAAATAAT

Btry AACGGAGGTGTCCCAAGGCCAGCTCAGTGCGGACAGAAACCACACATAGAGCAAAAGGGC

Bneo AACGGAGGTGTCCCAAGGCCAGCTCAGTGCGGACAGAAACCACACATAGAGCAAAAGGGC

Bjar AACGGAGGTGTCCCAAAGCCAGCTCAGTGCGGACAGAAACCACACATAGAGCAAAAGGGC

Dmel AACGGAGGTGTCCCAAGGCCAGCTCAGTGCGGACAGAAACCACACATAGAGCAAAAGGGC

Btry AAATGCTGACTTGATCTCGGTGTTCAGTACACACAGAGACAGCAAAAGCTCGGCCTATCG

Bneo AAATGCTGACTTGATCTCGGTGTTCAGTACACACAGAGACAGCAAAAGCTCGGCCTATCG

Bjar AAATGCTGACTTGATCTCGGTGTTCAGTACACACAGAGACAGCAAAAGCTCGGCCTATCG

Dmel AAATGCTGACTTGATCTCGGTGTTCAGTACACACAGGGACAGCAAAAGCTCGGCCTATCG

Btry ATCCTTTTGGTTTAAAGAGTTTTTAACAAGAGGTGTCAGAAAAGTTACCACAGGGATAAC

Bneo ATCCTTTTGGTTTAAAGAGTTTTTAACAAGAGGTGTCAGAAAAGTTACCACAGGGATAAC

Bjar ATCCTTTTGGTTTAAAGAGTTTTTAACAAGAGGTGTCAGAAAAGTTACCACAGGGATAAC

Dmel ATCCTTTTGGTTTAAAGAGTTTTTAACAAGAGGTGTCAGAAAAGTTACCATAGGGATAAC

Btry TGGCTTGTGGCGGCCAAGCGTTCATAGCGACGTCGCTTTTTGATCCTTCGATGTCGGCTC

Bneo TGGCTTGTGGCGGCCAAGCGTTCATAGCGACGTCGCTTTTTGATCCTTCGATGTCGGCTC

Bjar TGGCTTGTGGCGGCCAAGCGTTCATGGCGACGTCGCTTTTTGATCCTTCGATGTCTGCTC

Dmel TGGCTTGTGGCGGCCAAGCGTTCATAGCGACGTCGCTTTTTGATCCTTCGATGTCGGCTC

Btry TTCCTATCATTGTGAAGCAAAATTCACCAAGCGTTGGATTGTTCACCCATTCAAGGGAAC

Bneo TTCCTATCATTGTGAAGCAAAATTCACCAAGCGTTGGATTGTTCACCCATTCAAGGGAAC

Bjar TTCCTATCATTGTGAAGCAAAATTCACCAAGCGTTGGATTGTTCACCCATTCAA-GGAAC

Dmel TTCCTATCATTGTGAAGCAAAATTCACCAAGCGTTGGATTGTTCACCCATGCAAGGGAAC

Btry GTGAGCTGGGTTTAGACCGTCGTGAGACAGGTTAGTTTTACCCTACTAATGAC-AATTGT

Bneo GTGAGCTGGGTTTAGACCGTCGTGAGACAGGTTAGTTTTACCCTACTAATGAC-AATTGT

Bjar ATGAGCTGGATTTAGACCGTCTTGAGACAGGTTAGTTTTACCCTACTAATGAC-AATTGT

Dmel GTGAGCTGGGTTTAGACCGTCGTGAGACAGGTTAGTTTTACCCTACTAATGACAAAACGT

Btry TATTGCGACAGCATTCCTGCGTAGTACGAGAGGAACCGCAGGTACGGACCAATGGTACAA

Bneo TATTGCGACAGCATTCCTGCGTAGTACGAGAGGAACCGCAGGTACGGACCAATGGTACAA

Bjar TATTGCGACAGCATTCCTGCGTAGTACGAGAGGAACCGCAGGTGCGGACCAATGGTACAA

Dmel TGTTGCGACAGCATTCCTGCGTAGTACGAGAGGAACCGCAGGTACGGACCAATGGCACAA

Btry TACTTGTTCGAGCGAACAGTGGTATGATGCTACGTCCGTTGGATTATGCCTGAACGCCTC

Bneo TACTTGTTCGAGCGAACAGTGGTATGATGCTACGTCCGTTGGATTATGCCTGAACGCCTC

Bjar TACTTGTTCGAGCGAACAGTGGTATGATGCTACGTCCGTTGGATTATGCCTGAACGCCTC

Dmel TACTTGTTCGAGCGAACAGTGGTATGACGCTACGTCCGTTGGATTATGCCTGAACGCCTC

Btry TAAGGTCGTATCCGTGCTGGACTGCAATGATAAATATGGGGCAA-TTGCATTGTATGGCT

Bneo TAAGGTCGTATCCGTGCTGGACTGCAATGATAAATATGGGGCAA-TTGCATTGTATGGCT

Bjar TAAGGACGTATCCGTGCTGGACTGCAATGATAAATATGGGGCAA-TTGCCTTGTATGGCT

Dmel TAAGGTCGTATCCGTGCTGGACTGCAATGATAAATAAGGGGCAATTTGCATTGTATGGCT

Btry TCTCTAAACCATTTAAAGTTTATAAATTTTATTTATAAACGACAATGGATATATGTGATG

Bneo TCTCTAAACCATTTAAAGTTTATAAATTTTATTTATAAACGACAATGGATATATGTGATG

Bjar TCTCTAAACCATTTAAAGTTTATAAATTTTATTTATAAACGACAATGGATATATGTGATG

Dmel --TCTAAACCATTTAAAGTTTAT-AATTTACTTTATAAACGACAATGG----ATGTGATG

Btry CCAATGTTATTTGTAACATAGCAAATGCGGGAGGATTAAATATCACCTGTATGACGCGCT

Bneo CCAATGTTATTTGTAACATAGCAAATGCGGGAGGATTAAATATCACCTGTATGACGCGCT

Bjar CCAATGTTATTTGCAACATAGCAAATGCGGGAGGATTAAATATCACCTGTATGACGCGCT

Dmel CCAATGTAATTTGTAACATAGTAAAT-TGGGAGGATCTTCGATCACCTG-ATGCCGCGCT

Btry AGTTACTTATTAAAACATTATTTAATACAATGTCAATGCCTAGAATCAATTGTAAACGAC

Bneo AGTTACTTATTAAAACATTATTTAATACAATGTCAATGCCTAGAATCAATTGTAAACGAC

Bjar AGTTACTTATTAAAACATTATTTAATACAATGTCAATACCTAGAATCAATTGTAAACGAC

Dmel AGTTACATATAAAAGCATTATTTAATACAATGACAAAGCCTAGAATCAATTGTAAACGAC

Btry TTTGGTAACGGGCAAGGTGTTGTAAGTGGTAGAGCAGCTGCCATACTGCGATCCACTGAA

Bneo TTTGGTAACGGGCAAGGTGTTGTAAGTGGTAGAGCAGCTGCCATACTGCGATCCACTGAA

Bjar TTTGGTAACGGGCAAGGTGTTGTAAGCGGTAGAGCAGCTGAAATACTGCGATCCACTGAA

Dmel TTTTGTAACAGGCAAGGTGTTGTAAGTGGTTGAGCAGCTGCCATACTGCGATCCACTGAA

Btry GCTTATCCTTTGCTTGATGATTCGATCATACTGTTTTATAAATATATATAATATGTATAA

Bneo GCTTATCCTTTGCTTGATGATTCGATCATACTGTTTTATAAATATATATAATATGTATAA

Bjar GCTTATCCTTTGCTTGATGATTCGATCATACTGTTTTATAAATATATAT----------A

Dmel GCTTATCCTTTGCTTGATGATTCGA-----------------------------------

28S end

Btry TATATATATATATATATATAAT-TATTGTATATTATTTATTTATACAATATATTAAAAAA

Bneo TATATATATATATATATATAAT-TATTGTATATTATTTATTTATACAATATATTAAAAAA

Bjar TATATATATATATATATATAATATATTGTATATTATTTATTTATACAAT----------A

Dmel ------------------------------------------------------------

Btry TATTATATATTTATTTATTTATATATAAAATATAAAAGAAAACATCTAATTTTAAATAAT

Bneo TATTATATATTTATTTATTTATATATAAAATATAAAAGAAAACATCTAATTTTAAATAAT

Bjar TATTATATATTTA-----------------------------------------------

Dmel ------------------------------------------------------------

Btry TAATTATTAAAAATTAGATTAAGTATTTTATATATAATATATAAGAATGTATAATAAATT

Bneo TAATTATTAAAAATTAGATTAAGTATTTTATATATAATATATAAGAATGTATAATAAATT

Bjar --------------------------TTTATATATAATAAATAAGATTATATAATAAATT

Dmel ------------------------------------------------------------

Btry ATGTAAATTAAATAAACATATATTTATATATATTATATATTTATAAACAGTGTAAAAAGA

Bneo ATGTAAATTAAATAAACATATATTTATATATATTATATATTTATAAACAGTGTAAAAAGA

Bjar ATGTAAGTTAAATAAACATATAT---------TTATATATTTAT-AACAGTGTAAAAAGA

Dmel ------------------------------------------------------------

Btry ATCATTTCATTTATAATAATAAAAAGAAAAATATTTGAACGAATAATACAATATGATTGT

Bneo ATCATTTCATTTATAATAATAAAAAGAAAAATATTTGAACGAATAATACAATATGATTGT

Bjar ATCATTTCATTTTTAAAAATAAAAATTAAAATATTCAAACAAAT-----AATATGATTGT

Dmel ------------------------------------------------------------

Btry TATTA------AACAACTATTT-TATAATATTATACCCCAAAAGTATAAAATGTATAGAA

Bneo TATTAAAACTCAACTACTATTT-TATAATATTATACCCCAAAAGTATAAAATGTATAGAA

Bjar TATTA------AACAACTATTTATATAATA-TATACCCGAAAA--ATAAAATGTATAGA-

Dmel ------------------------------------------------------------

Btry AATAAATAAATAAATATATATAAATATAAATGGGTATATGTTATATTAAGTGTTTGAGAG

Bneo AATAAATAAATAAATATATATAAATATAAATGGGTATATGTTATATTAAGTGTTTGAGAG

Bjar -------AATTAAATATATATAAATATAAATGGGTATATGTTATATAAAGTGTTTGAGAG

Dmel ------------------------------------------------------------

Btry AATCATAAAATGAAAATAAAAAATATTATGAACGCTAAATAAT---TTGTTTTTAATATT

Bneo AATCATAAAATGAAAATAAAAAATATTATGAACGCTAAATAAT---TTGTTTTTAATATT

Bjar AATCATTAAATGAAAATAAAAAATATTATAAACGCTAAATACTTTGTTGTTTTCAATATT

Dmel ------------------------------------------------------------

Btry TATTATTTAAAAGCAAAGTATAAGTGTTG---TGATTCATATTAAACGCAACTACATATA

Bneo TATTATTTAAAAGCAAAGTATAAGTGTTG---TGATTCATATTAAACGCAACTACATATA

Bjar TATTATTAAAAGGCAAAGTATAAGTGTTGTGATGATTCATATTAAACACAACTACATATA

Dmel ------------------------------------------------------------

Btry ATATACAAAAAAAAAAAAAACATATATATATATATAGTAACACA----AAACGAACGATA

Bneo ATATACAAAAAAAAAAA----ACATATATATATATAGTAACACA----AAACGAACGATA

Bjar ATATACAAAAATCAAAAA---AAAAAAAAAAATATAGTAACACAATACAAATGAACGATA

Dmel ------------------------------------------------------------
