# Supplementary material for: The draft genome of the pest tephritid fruit fly Bactrocera tryoni: resources for the genomic analysis of hybridising species
Source: BMC Genomics. 2014 Dec 20;15(1):1153. doi: 10.1186/1471-2164-15-1153 (PMC4367827; doi:10.1186/1471-2164-15-1153)
Supplement: Supplementary file 5 — Additional file 5: The spacing of satellite DNA 12-mers in the 100bp reads. For the satellite sequence Btry_Sat1 (166 bp in length), the histogram shows the frequency distribution of the spacing between the 12-mer beginning at position 1 of the canonical sequence and other 12-mers from the same satellite sequence that are close enough to co-occur on the same 100 bp read. The 12-mers in the 3’ direction start at positions 13, 25, 37 etc, while the 12-mers in the 5’ direction start at -11, -23 etc. The distance expected from the canonical sequence is shown above each distribution. In each case, the >92% of the 12-mers occur at or near the expected distance and in the expected direction, which is consistent with the existence of large tandem repeat arrays. The 13 separate frequency distributions are non-overlapping at this scale and are therefore presented as one histogram. The frequencies decrease with absolute distance from the 1-position 12-mer due to the decreasing number of 100 bp reads that span both 12-mers. Some decrease is also due to cumulative sequence variation. In all cases, 12-mers were scored as matching if they differed from the canonical sequence by no more than a single 1 bp substitution. (DOC 61 KB) [file 12864_2014_6888_MOESM5_ESM.doc]

13

37

25

49

61

73

-11

-47

-35

-23

-71

-59

Additional File 4.
